# Peptide Conjugates Derived from flg15, Pep13, and PIP1 That Are Active against Plant-Pathogenic Bacteria and Trigger Plant Defense Responses

**DOI:** 10.1128/aem.00574-22

**Published:** 2022-05-31

**Authors:** Àngel Oliveras, Cristina Camó, Pau Caravaca-Fuentes, Luís Moll, Gerard Riesco-Llach, Sergio Gil-Caballero, Esther Badosa, Anna Bonaterra, Emilio Montesinos, Lidia Feliu, Marta Planas

**Affiliations:** a LIPPSO, Department of Chemistry, University of Girona, Girona, Spain; b Laboratory of Plant Pathology, Institute of Food and Agricultural Technology-CIDSAV-XaRTA, University of Girona, Girona, Spain; c Serveis Tècnics de Recerca (NMR), University of Girona, Parc Científic i Tecnològic de la UdG, Girona, Spain; University of Tennessee at Knoxville

**Keywords:** antimicrobial peptide, peptide conjugate, plant defense elicitor, plant disease, secondary structure

## Abstract

Thirty peptide conjugates were designed by combining an antimicrobial peptide (BP16, BP100, BP143, KSL-W, BP387, or BP475) at the N- or C-terminus of a plant defense elicitor peptide (flg15, BP13, Pep13, or PIP1). These conjugates were highly active *in vitro* against six plant-pathogenic bacteria, especially against Xanthomonas arboricola pv. pruni, Xanthomonas fragariae and Xanthomonas axonopodis pv. vesicatoria. The most active peptides were those incorporating Pep13. The order of the conjugation influenced the antibacterial activity and the hemolysis. Regarding the former, peptide conjugates incorporating the elicitor peptide flg15 or Pep13 at the C-terminus were, in general, more active against Pseudomonas syringae pv. actinidiae and P. syringae pv. syringae, whereas those bearing these elicitor peptides at the N-terminus displayed higher activity against Erwinia. amylovora and the *Xanthomonas* species. The best peptide conjugates displayed MIC values between 0.8 and 12.5 μM against all the bacteria tested and also had low levels of hemolysis and low phytotoxicity. Analysis of the structural and physicochemical parameters revealed that a positive charge ranging from +5 to +7 and a moderate hydrophobic moment/amphipathic character is required for an optimal biological profile. Interestingly, flg15-BP475 exhibited a dual activity, causing the upregulation of the same genes as flg15 and reducing the severity of bacterial spot in tomato plants with a similar or even higher efficacy than copper oxychloride. Characterization by nuclear magnetic resonance (NMR) of the secondary structure of flg15-BP475 showed that residues 10 to 25 fold into an α-helix. This study establishes trends to design new bifunctional peptides useful against plant diseases caused by plant-pathogenic bacteria.

**IMPORTANCE** The consequences of plant pathogens on crop production together with the lack of effective and environmentally friendly pesticides evidence the need of new agents to control plant diseases. Antimicrobial and plant defense elicitor peptides have emerged as good candidates to tackle this problem. This study focused on combining these two types of peptides into a single conjugate with the aim to potentiate the activity of the individual fragments. Differences in the biological activity of the resulting peptide conjugates were obtained depending on their charge, amphipathicity, and hydrophobicity, as well as on the order of the conjugation of the monomers. This work provided bifunctional peptide conjugates able to inhibit several plant-pathogenic bacteria, to stimulate plant defense responses, and to reduce the severity of bacterial spot in tomato plants. Thus, this study could serve as the basis for the development of new antibacterial/plant defense elicitor peptides to control bacterial plant pathogens.

## INTRODUCTION

The finding of new agents to control plant diseases represents an important advance in tackling the lack of effective and environmentally friendly pesticides. In this context, antimicrobial peptides highlight as a promising choice (1–4). These peptides, found in the innate immune response of all classes of living organisms, display exceptional biological properties, such as a broad-spectrum antimicrobial activity and a low propensity to induce microbial resistance (5–12). These inherent advantages have prompted the search for new antimicrobial peptides with high antimicrobial activity and low toxicity. In this search, it is crucial to take into account the structural features that govern the mechanism of action of these compounds that mostly target the negatively charged bacterial membrane (5, 13, 14). The cationic character of antimicrobial peptides and their ability to adopt an amphipathic structure allow their interaction with the bacterial membrane and their insertion into the lipid bilayer.

Several strategies can be used to design novel antimicrobial peptides. Among them, the combination of two individual peptides into a single hybrid or chimeric sequence aims to potentiate or maintain the activity of the native fragments or to diminish their toxicity (15–18). The majority of studies of hybrid peptides have focused on combining two fragments of natural peptides, both of them with antimicrobial activity (19–33). Fewer studies have investigated the merging of an antimicrobial peptide with a sequence displaying a different activity to generate bifunctional peptides in which one of the individual sequences potentiates the activity of the other (34–40). In addition, even though the order of the monomers in the sequence of the peptide conjugates has been described to influence the activity of these conjugates, this effect has not been thoroughly analyzed (41–44).

During the past years, we have developed linear undecapeptides with high activity against the Gram-negative plant-pathogenic bacteria Erwinia amylovora, Xanthonomas axonopodis pv. vesicatoria, *Xanthonomas axonopodis* pv. pruni, Xanthomonas fragariae, Pseudomonas syringae pv. actinidiae, and Pseudomonas syringae pv. syringae. These peptides also showed low hemolytic and phytotoxic activities. In particular, from a 125-member library (CECMEL11), we identified the peptides KKLFKKILKKL-NH_2_ (BP16) and KKLFKKILKYL-NH_2_ (BP100) (45). The biological profile of BP100 was optimized by incorporating a d-amino acid and/or a fatty acid chain into its sequence, providing derivatives KKLfKKILKYL-NH_2_ (BP143) (46), Ac-KKLFKKIK(COC_3_H_7_)KYL-NH_2_ (BP387) (47), and Ac-KKLfKKILKK(COC_3_H_7_)L-NH_2_ (BP475) (48). These derivatives displayed improved biological activities in terms of antibacterial activity and hemolysis. More recently, we identified the linear decapeptide KKVVFWVKFK-NH_2_ (KSL-W), which exhibited high antibacterial activity against all the above-mentioned bacteria, low hemolysis, and low phytotoxicity in tobacco leaves (49). In addition, in a study focused on obtaining suitable sequences to be expressed in plants, we designed peptide conjugates derived from BP100 (41). The resulting sequences were more active against the above-mentioned plant pathogens and less hemolytic than the corresponding monomers and were expressed in rice seed endosperm (50).

Plant defense elicitors also constitute an alternative to control plant pathogens (51, 52). In fact, some peptides have been described to trigger plant immune responses by activating defense signaling pathways that enable plants to counteract the attack by pathogens (53–56). Our group found that the linear undecapeptide FKLFKKILKVL-NH_2_ (BP13), which exhibits antimicrobial activity, also induces the overexpression of genes related to plant defense responses on tobacco cells and tomato plants (52). The induction of plant defences by BP13 could also contribute to its efficiency in controlling fire blight infections caused by E. amylovora in pear plants. Another plant defense elicitor peptide is RINSAKDDAAGLQIA-OH (flg15), which corresponds to 15 amino acids of the N-terminal conserved domain of bacterial flagellin and activates the immune system in tomato (57–61). In addition, the sequence YGIHTH-NH_2_ (PIP1), identified through combinatorial chemistry, induces the jasmonic acid signaling pathway and defense-related secondary metabolites in tobacco cells (62–64), and peptide VWNQPVRGFKVYE-OH (Pep13), a pathogen-associated molecular pattern (PAMP) from Phytophthora sojae, triggers multiple defense responses in parsley and potato (65–70).

Recently, we have identified two peptide conjugates able to protect plants against pathogen infection through a double mechanism of action consisting of antimicrobial activity against the pathogen and plant defense elicitation. In particular, flg15-BP16, bearing the antimicrobial undecapeptide BP16 at the C-terminus of the plant defense elicitor peptide flg15, showed antimicrobial and plant defense elicitation activities in an Erwinia amylovora/pear pathosystem (44). Similarly, BP178, derived from BP100 and magainin(1-10), controlled infections in tomato plants caused by two bacteria and a fungus, and it also induced a gene expression pattern comparable to that of flg15 (71).

The above-described initial studies prompted us to perform a thorough search for new bifunctional peptide conjugates useful to control plant diseases. They were designed by conjugating an antimicrobial peptide (a CECMEL11 peptide, a CECMEL11 derivative, or KSL-W) at the N- or C-terminus of a plant defense elicitor peptide (flg15, BP13, Pep13, PIP1). For the resulting 30 peptide conjugates, structural parameters and physicochemical properties as well as their antibacterial activity and toxicity to eukaryotic cells and tobacco leaves were determined. The effect of selected peptide conjugates on defense gene expression of plants and their efficacy in reducing the severity of bacterial spot in tomato plants were also analyzed. Finally, the secondary structure of the best peptide conjugate was characterized by nuclear magnetic resonance (NMR) spectroscopy.

## RESULTS

### Design and physicochemical properties of the peptide conjugates.

Peptide conjugates were designed by combining an antimicrobial peptide with a sequence with the ability to induce plant defense responses (Table 1). The antimicrobial peptide was selected among the peptides identified in previous studies with activity against plant-pathogenic bacteria and with low toxicity, and included peptides BP16, BP100, BP143, and KSL-W, and lipopeptides BP387 and BP475. The plant defense elicitor peptides chosen were flg15, BP13, Pep13, and PIP-1. To analyze the influence of the order of the monomers in the antibacterial activity of the resulting conjugate, the antimicrobial peptide was incorporated at the N- or C-terminus of the corresponding plant defense elicitor peptide.

**TABLE 1:** Sequence and physicochemical properties of peptide conjugates

Peptide	Sequence^*a*^	Length^*b*^	Net charge	HR (%)^*c*^	tr (min)^*d*^	GRAVY^*e*^	<μH>^*f*^
flg15-BP16	RINSAKDDAAGLQIA-KKLFKKILKKL-NH_2_	26	+6	46	5.89	–0.319	0.332
BP16-flg15	KKLFKKILKKL-RINSAKDDAAGLQIA-OH	26	+6	46	4.59	–0.319	0.284
flg15-BP100	RINSAKDDAAGLQIA-KKLFKKILKYL-NH_2_	26	+5	50	6.29	–0.219	0.323
BP100-flg15	KKLFKKILKYL-RINSAKDDAAGLQIA-OH	26	+5	50	4.96	–0.219	0.295
flg15-BP387	Ac-RINSAKDDAAGLQIA-KKLFKKIK(COC_3_H_7_)KYL-NH_2_	26	+5	50	6.35	–0.219^*g*^	0.323^*g*^
BP387-flg15	Ac-KKLFKKIK(COC_3_H_7_)KYL-RINSAKDDAAGLQIA-OH	26	+5	50	5.84	–0.219^*g*^	0.295^*g*^
flg15-BP475	Ac-RINSAKDDAAGLQIA-KKLfKKILKK(COC_3_H_7_)L-NH_2_	26	+5	50	6.79	–0.023^*g*^	0.325^*g*^
BP475-flg15	Ac-KKLfKKILKK(COC_3_H_7_)L-RINSAKDDAAGLQIA-OH	26	+5	50	6.03	–0.023^*g*^	0.304^*g*^
flg15-KSLW	RINSAKDDAAGLQIA-KKVVFWVKFK-NH_2_	25	+4	52	5.40	–0.076	0.079
KSLW-flg15	KKVVFWVKFK-RINSAKDDAAGLQIA-OH	25	+4	52	4.75	–0.076	0.053
BP13-BP16	FKLFKKILKVL-KKLFKKILKKL-NH_2_	22	+10	54	7.85	0.245	0.827
BP16-BP13	KKLFKKILKKL-FKLFKKILKVL-NH_2_	22	+10	54	7.68	0.245	0.818
BP13-BP100	FKLFKKILKVL-KKLFKKILKYL-NH_2_	22	+9	59	8.56	0.364	0.842
BP100-BP13	KKLFKKILKYL-FKLFKKILKVL-NH_2_	22	+9	59	8.23	0.364	0.802
BP13-BP143	FKLFKKILKVL-KKLfKKILKYL-NH_2_	22	+9	59	8.10	0.364^*g*^	0.842^*g*^
BP143-BP13	KKLfKKILKYL-FKLFKKILKVL-NH_2_	22	+9	59	8.34	0.364^*g*^	0.802^*g*^
BP13-KSLW	FKLFKKILKVL-KKVVFWVKFK-NH_2_	21	+8	62	7.02	0.562	0.420
KSLW-BP13	KKVVFWVKFK-FKLFKKILKVL-NH_2_	21	+8	62	6.26	0.562	0.519
Pep13-BP16	VWNQPVRGFKVYE-KKLFKKILKKL-NH_2_	24	+7	50	5.70	–0.517	0.503
BP16-Pep13	KKLFKKILKKL-VWNQPVRGFKVYE-OH	24	+7	50	5.12	–0.517	0.161
Pep13-BP100	VWNQPVRGFKVYE-KKLFKKILKYL-NH_2_	24	+6	54	6.14	–0.408	0.549
BP100-Pep13	KKLFKKILKYL-VWNQPVRGFKVYE-OH	24	+6	54	5.53	–0.408	0.126
Pep13-BP143	VWNQPVRGFKVYE-KKLfKKILKYL-NH_2_	24	+6	54	5.97	–0.408^*g*^	0.549^*g*^
BP143-Pep13	KKLfKKILKYL-VWNQPVRGFKVYE-OH	24	+6	54	5.62	–0.408^*g*^	0.126^*g*^
Pep13-KSLW	VWNQPVRGFKVYE-KKVVFWVKFK-NH_2_	23	+5	56	5.15	–0.261	0.364
KSLW-Pep13	KKVVFWVKFK-VWNQPVRGFKVYE-OH	23	+5	56	5.10	–0.261	0.185
PIP1-BP475	Ac-YGIHTH-KKLfKKILKK(COC_3_H_7_)L-NH_2_	17	+5	47	5.55	–0.076^*g*^	0.563^*g*^
BP475-PIP1	Ac-KKLfKKILKK(COC_3_H_7_)L-YGIHTH-NH_2_	17	+5	47	6.28	–0.076^*g*^	0.600^*g*^
PIP1-KSLW	YGIHTH-KKVVFWVKFK-NH_2_	16	+4	50	4.81	–0.163	0.098
KSLW-PIP1	KKVVFWVKFK-YGIHTH-NH_2_	16	+4	50	4.83	–0.163	0.189

a COC_3_H_7_, butanoyl; lowercase letters correspond to d-amino acids.

b Number of amino acids of the sequence.

c Hydrophobic ratio; percentage of hydrophobic amino acids relative to the total number of amino acids in the peptide sequence.

d HPLC retention time (method B).

e Grand average of hydropathicity, obtained from ProtParam (Expasy Proteomics Server). A higher positive score indicates greater hydrophobicity and *vice versa*.

f Mean hydrophobic moment, obtained from HeliQuest.

g To obtain these values d-Phe has been replaced by l-Phe, and/or side-chain acylated Lys has been replaced by Leu.

The synthesis of the peptide conjugates was performed manually on solid phase following a 9-fluorenylmethoxycarbonyl (Fmoc)/*tert*-butyl (*t*Bu) strategy. They were obtained in excellent purities (96 to >99% high-pressure liquid chromatography [HPLC] purity) and were characterized by mass spectrometry (see Table SD in the supplemental material).

The parameters related to the structure of the peptide conjugates (sequence, charge, hydrophobic ratio [HR], and HPLC retention time), as well as their physicochemical properties (grand average hydropathicity [GRAVY] and hydrophobic moment [<μH>]) obtained from the online programs ProtParam (Expasy server) and HeliQuest are included in Table 1. These conjugates contain 16 to 26 amino acids, a positive charge of 4 to 10, and a hydrophobic content ranging from 46 to 62%. Regarding the HPLC retention time, which is an important indicator of the relative hydrophobicity level of the peptides, longer retention times were observed for conjugates containing BP13 than for the other sequences. Consistent with these data, positive GRAVY values were obtained for the peptide conjugates incorporating BP13, whereas for the other sequences, the GRAVY values were negative. These results suggest that the former are the most hydrophobic, while the latter are moderately hydrophilic. In the case of the hydrophobic moment, defined as the vector sum of the hydrophobicity of individual amino acids, the conjugation of BP13 with BP16, BP100, or BP143 led to sequences with the highest value, around 0.8. This result reveals that these conjugates can adopt a perfect amphipathic structure. It can be noted that the presence of KSL-W in the peptide conjugates of this study leads to a decrease of the hydrophobic moment, suggesting a reduced amphipathicity. In addition, it can be clearly observed that those sequences containing the elicitor peptide flg15, BP13, or Pep13 at the N-terminus showed a longer HPLC retention time and a higher hydrophobic moment than their counterparts with these elicitor peptides at the C-terminus.

### Antibacterial activity.

**FIG 1:**
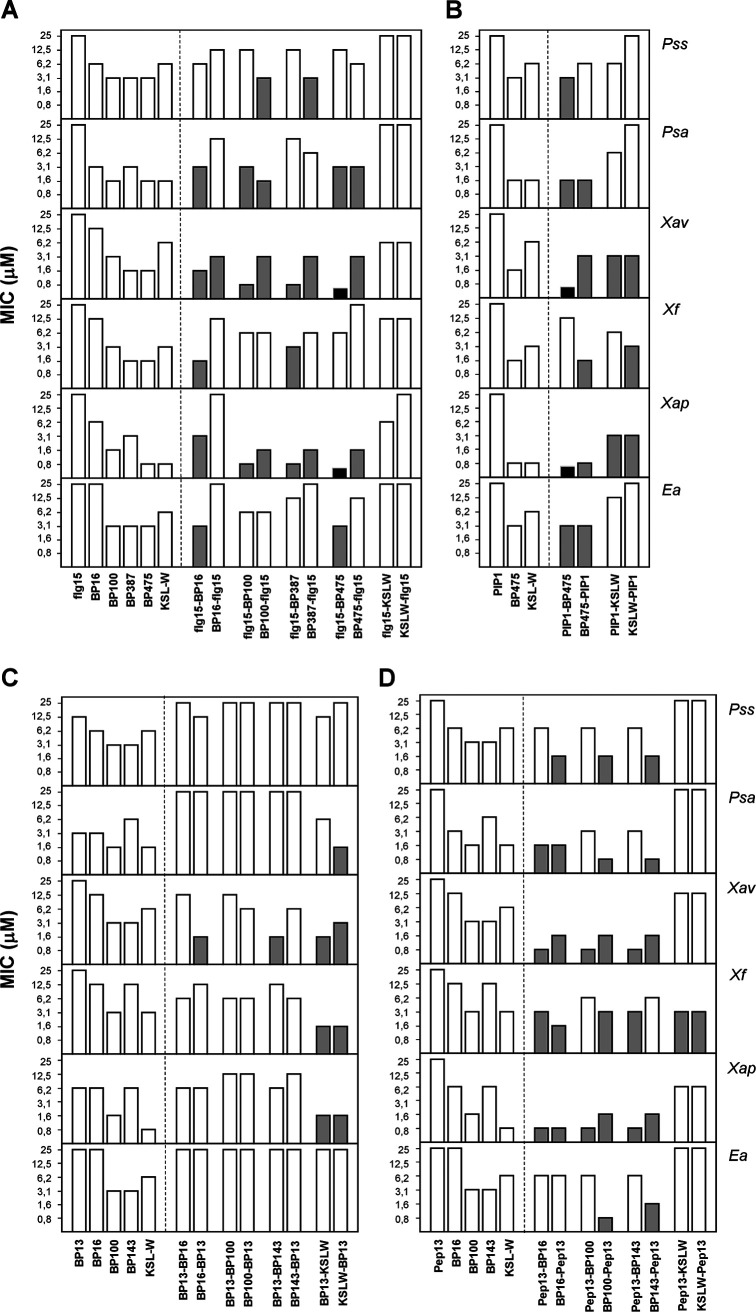
Antibacterial activity of peptide conjugates against E. amylovora (*Ea*), X. arboricola pv. pruni (*Xap*), *X. fragariae* (*Xf*), *X. axonopodis* pv. vesicatoria (*Xav*), P. syringae pv. actinidiae (*Psa*), and P. syringae pv. syringae (*Pss*). Antibacterial activity is given as the minimal concentration that inhibits growth (MIC). The MIC axis is in logarithmic scale, and for each sequence the lowest value of the MIC range is represented. Peptide conjugates with MIC values between 0.8 and 6.2 μM are highlighted in gray. Peptide conjugates with a MIC of <0.8 μM are highlighted in black. (A to D) Peptide conjugates derived from flg15 (A), PIP1 (B), BP13 (C), and Pep13 (D). The corresponding monomers are also included in each panel. Data can be found in Table SA (supplemental material).

Peptide conjugates were tested for *in vitro* growth inhibition of the plant-pathogenic bacteria E. amylovora PMV6076, Xanthomonas arboricola pv. pruni CFBP5563, *X. fragariae* Xf349-9A, *X. axonopodis* pv. vesicatoria 2133-2, P. syringae pv. actinidiae Psa3700.1.1, and P. syringae pv. syringae EPS94 (Fig. 1, Table SA). The corresponding monomers (flg15, BP13, Pep13, PIP1, BP16, BP100, BP143, BP387, BP475, and KSL-W) were included for comparison. As expected, the plant defense elicitor peptides flg15, BP13, Pep13, and PIP1 were not active (MIC > 50 μM).

Out of 30 peptide conjugates, 18 displayed MIC values of <6.2 μM against at least 3 pathogens, and 9 of them, MIC values of <12.5 μM against the 6 bacteria. Interestingly, 11 conjugates exhibited MIC values of <1.6 μM against at least 1 pathogen. The most sensitive bacteria toward these compounds were X. arboricola pv. pruni, *X. fragariae*, and *X. axonopodis* pv. vesicatoria: 25, 23, and 26 peptides showed MIC values of <12.5 μM, respectively.

Analysis of the antibacterial activity of the four families of peptide conjugates, grouped according to the plant defense elicitor peptide, revealed differences between them. BP13 conjugates were the least active. However, BP13-KSLW and KSLW-BP13 showed MIC values between 1.6 and 3.1 μM against three bacteria. Peptide conjugates containing Pep13 were the most active. In fact, except for the sequences incorporating KSL-W, the other six peptides of this set exhibited MIC values of <12.5 μM against all the pathogens tested. These six peptides are particularly relevant for their activity against X. arboricola pv. pruni and *X. axonopodis* pv. vesicatoria, displaying MIC values between 0.8 and 3.1 μM. The most active Pep13 derivative was BP100-Pep13, with MIC values between 0.8 and 6.2 μM against all the bacteria. Peptide conjugates derived from flg15 were less active than those containing Pep13. Among them, the least active were again those incorporating KSL-W. Conjugates of this set displaying the highest activity were the three sequences incorporating BP100, BP387, and BP475 at the C-terminus (MIC values between <0.8 and 1.6 μM against X. arboricola pv. pruni and *X. axonopodis* pv. vesicatoria). In the case of the PIP1 conjugates, similar to the previous families, the KSL-W derivatives were the least active. The two conjugates incorporating BP475 displayed MIC values between <0.8 and 6.2 μM against X. arboricola pv. pruni and *X. axonopodis* pv. vesicatoria.

The antibacterial activity of the peptide conjugates was compared to that of the corresponding monomers BP16, BP100, BP143, BP387, BP475, and KSL-W, which were reported as antimicrobial peptides active against the above-mentioned pathogens. In general, the combination of BP16 (MIC between 3.1 and 50 μM) with flg15 or Pep13 resulted in peptides with higher activity than that of BP16 (flg15-BP16, MIC between 1.6 and 12.5 μM; Pep13-BP16 and BP16-Pep13, MIC between 0.8 and 12.5 μM). Regarding peptide conjugates incorporating BP100 (MIC between 1.6 and 6.2 μM) and flg15 or Pep13, we found sequences with lower MIC values than BP100, especially against X. arboricola pv. pruni and *X. axonopodis* pv. vesicatoria (MIC between 0.8 and 3.1 μM). The improvement of activity was remarkable in the case of BP100-Pep13 (MIC between 0.8 and 3.1 μM against four pathogens). The combination of BP143 (MIC between 3.1 and 25 μM) with Pep13 also gave rise to conjugates more active than BP143 (MIC between 0.8 and 12.5 μM). Regarding the lipopeptide BP387 (MIC between 1.6 and 6.2 μM), its activity was improved against X. arboricola pv. pruni when it was linked either to the C- or N-terminus of flg15 (MIC between 0.8 and 3.1 μM), and also against *X. axonopodis* pv. vesicatoria when it was conjugated at the C-terminus of flg15 (MIC of 0.8 to 1.6 μM). In the case of lipopeptide BP475 (MIC between 0.8 and 6.2 μM), its incorporation at the C-terminus of flg15 or PIP1 provided conjugates with higher activity against X. arboricola pv. pruni and *X. axonopodis* pv. vesicatoria (MIC < 0.8 μM). Finally, in general, the conjugation of KSL-W (MIC between 0.8 and 12.5 μM) with flg15, BP13, or Pep13 led to a loss of the activity. Nevertheless, BP13-KSLW and KSLW-BP13 were more active than KSL-W against *X. axonopodis* pv. vesicatoria and *X. fragariae* (MIC of 1.6 to 6.2 μM) and PIP1-KSLW and KSLW-PIP1 against *X. axonopodis* pv. vesicatoria (MIC of 3.1 to 6.2 μM).

The influence on the activity of the order of monomers in the sequence of peptide conjugates was evaluated. In the case of the flg15 conjugates containing BP16, BP100, BP387, and BP475, it was observed that those incorporating flg15 at the C-terminus were, in general, more active against P. syringae pv. actinidiae and P. syringae pv. syringae, whereas those bearing flg15 at the N-terminus displayed lower MIC values against E. amylovora and the three *Xanthomonas* species. In contrast, KSLW-flg15 and flg15-KSLW were similarly active. The activity of Pep13 conjugates followed a similar trend, except against E. amylovora. Thus, derivatives containing Pep13 at the C-terminus were more active against P. syringae pv. actinidiae, P. syringae pv. syringae, and E. amylovora, and the hybrids incorporating Pep13 at the N-terminus showed higher activity against X. arboricola pv. pruni and *X. axonopodis* pv. vesicatoria. Peptide conjugates derived from BP13 exhibited similar activity irrespective of its position in the sequence. Finally, no general trend could be inferred for conjugates containing PIP1.

From all the above-described results, the best peptide conjugates were Pep13-BP16, BP16-Pep13, Pep13-BP100, BP100-Pep13, Pep13-BP143, BP143-Pep13, and BP475-PIP1 with MIC values between 0.8 and 12.5 μM against all the bacteria tested. Among them is highlighted BP100-Pep13 with MIC values between 0.8 and 6.2 μM. Peptide conjugates flg15-BP475 and PIP1-BP475 also stood out for their activity against X. arboricola pv. pruni and *X. axonopodis* pv. vesicatoria (MIC < 0.8 μM).

### Toxicity.

**FIG 2:**
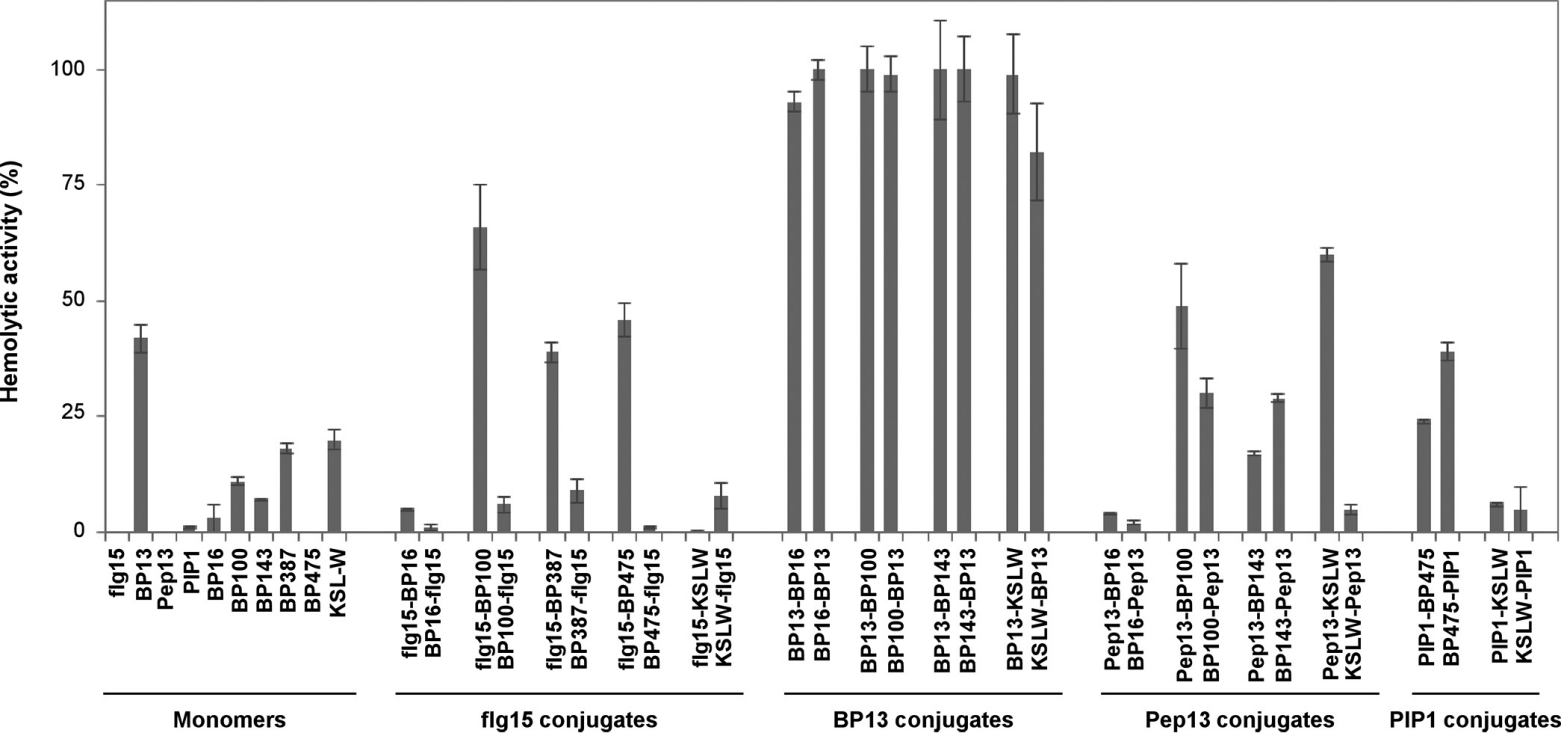
Hemolytic activity of the monomers and the peptide conjugates at 375 μM, expressed as a percentage compared to melittin as a standard. Vertical bars in each column indicate the confidence interval at the mean. Data can be found in Table SB (supplemental material).

The toxicity of peptide conjugates to eukaryotic cells was determined as the ability to lyse erythrocytes (Table SB). Percent hemolysis at 375 μM is shown in Fig. 2. The monomers had very low levels of hemolysis, and except for BP13, all sequences exhibited a percent hemolysis of ≤20% at 375 μM. Regarding the peptide conjugates, those derived from BP13 were the most hemolytic; all the peptides of this set displayed hemolysis of ≥62% at 50 μM. The 22 sequences from the other families had very low hemolysis. In particular, 16 of these 22 sequences exhibited hemolysis of ≤30% at 375 μM, and 12 of them showed hemolysis of ≤10% at this concentration. The evaluation of the influence on the hemolysis of the order of the monomers in the peptide conjugate revealed that in the case of the flg15 and Pep13 derivatives, those sequences containing the plant elicitor peptide at the C-terminus were, in general, less hemolytic than the ones bearing flg15 or Pep13 at the N-terminus.

**FIG 3:**
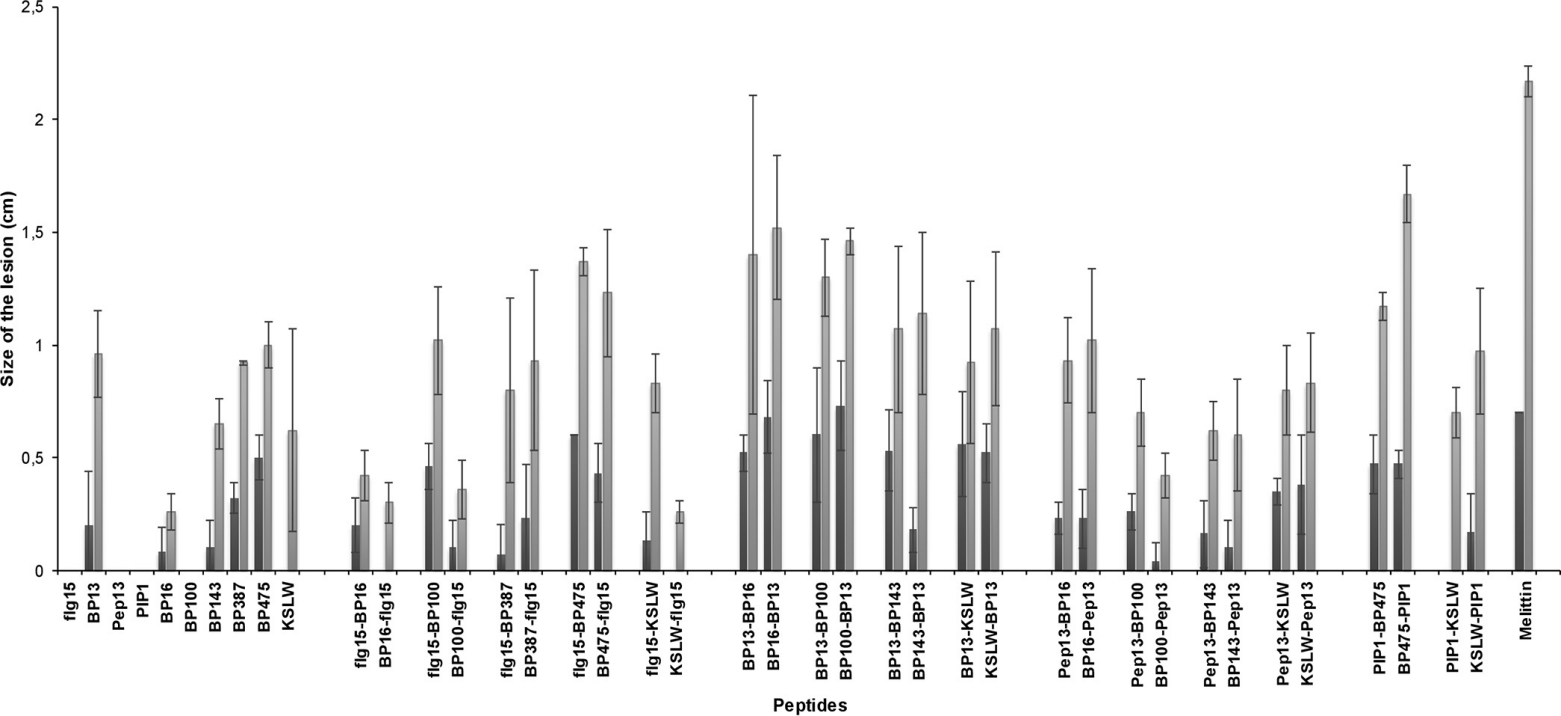
Effect of the monomers and the peptide conjugates on the size of the lesions in infiltrated tobacco leaves at 50 and 250 μM. This effect was compared to melittin. Vertical bars in each column indicate the confidence interval at the mean. Data can be found in Table SC (supplemental material).

Peptide conjugates were also assayed for their toxicity in tobacco leaves (Fig. 3, Table SC). After 48 h of infiltration, melittin caused a necrotic area of 0.70 and 2.17 cm in diameter at 50 and 250 μM, respectively. The monomers were not phytotoxic at those concentrations; all of them led to lesions of ≤1.2 cm at 250 μM. All peptide conjugates were also less phytotoxic than melittin, causing necrotic lesions between 0.26 and 1.67 cm in diameter at 250 μM. In particular, 15 of the 30 conjugates led to a necrotic area of <1 cm at this concentration. In general, sequences incorporating BP13 were among the most phytotoxic.

It should be pointed out that the best peptide conjugates displayed a percent hemolysis of <50% at 375 μM, a concentration 30- to 469-fold higher than their MIC, and were significantly less phytotoxic than melittin. Among the peptide conjugates with high activity against *Xanthomonas* species and low toxicity, flg15-BP387, flg15-BP475, and PIP1-BP475 were selected for further experiments in tomato plants.

### Effect of peptides on defense gene expression of tomato plants.

The capacity to induce the expression of genes related to defense response in tomato plants was evaluated for the peptide conjugates flg15-BP387, flg15-BP475, and PIP1-BP475 and the monomers flg15 and PIP1 (Table 2). Salicylic acid (SA) caused the upregulation of all the analyzed genes except for *PR9*, *GluB*, and *ERF*, whereas flg15 caused the upregulation of all tested genes except for *WRKY3* and *ERF*. PIP1 caused the overexpression of only one of the genes (*PR3*). Concerning the conjugates flg15-BP387 and flg15-BP475, they were able to cause an overexpression similar to that of flg15. Specifically, flg15-BP475 caused the upregulation of the same genes as flg15, and flg15-BP387 induced an overexpression of the same genes as flg15 except for *PR1*. The conjugate PIP1-BP475 induced the overexpression of the same gene as PIP1 and also caused the overexpression of *Harp* and *BCB.*

**TABLE 2:** Expression of genes related to defense response in tomato after treatment with the peptide monomers flg15 and PIP1 and the peptide conjugates flg15-BP387, flg15-BP475, and PIP1-BP475 compared to the reference elicitor salicylic acid (SA)^*a*^

Genes	Property/GO molecular function	Positive control (SA)^*b*^	Monomers^*c*^		Peptide conjugates^*c*^		
			flg15	PIP1	flg15-BP387	flg15-BP475	PIP1-BP475
*PR1*	Antimicrobial, fungicide	**39.56 **± 2.92^*d*^	**8.32 **± 3.35^*d*^	1.00 ± 0.39	1.75 ± 0.37^*d*^	**2.02 **± 0.07^*d*^	0.64 ± 0.36
*PR3*	Endochitinase	**17.20 **± 3.64^*d*^	**17.68 **± 4.18^*d*^	**3.59 **± 1.35^*d*^	**15.39 **± 2.88^*d*^	**10.96 **± 1.85^*d*^	**7.36 **± 1.05^*d*^
*PR7*	Serine-type endopeptidase	**6.48 **± 1.18^*d*^	**9.26 **± 2.06^*d*^	1.13 ± 0.26	**3.31 **± 0.37^*d*^	**5.82 **± 0.78^*d*^	1.29 ± 0.19^*d*^
*PR9*	Peroxidase	1.68 ± 0.05^*d*^	**6.24 **± 2.25^*d*^	1.23 ± 0.18	**3.12 **± 0.55^*d*^	**3.83 **± 0.25^*d*^	1.99 ± 0.11^*d*^
*WRKY3*	Transcription regulatory region DNA binding	**2.01 **± 0.34^*d*^	1.41 ± 0.84	0.94 ± 0.35	1.66 ± 0.51	1.16 ± 0.26	1.46 ± 0.11
*GluB*	β-1,3-Endoglucanase	0.59 ± 0.15^*d*^	**6.05 **± 2.40^*d*^	0.96 ± 0.32	**4.09 **± 0.63^*d*^	**4.31 **± 0.82^*d*^	0.80 ± 0.07
*Harp*	Hairpin-induced protein-like	**4.19 **± 0.20^*d*^	**4.28 **± 1.49^*d*^	1.97 ± 0.45^*d*^	**3.74 **± 0.51^*d*^	**3.68 **± 0.91^*d*^	**2.25 **± 0.12^*d*^
*BCB*	Copper ion binding, electron transfer activity	**2.78 **± 0.29^*d*^	**8.14 **± 2.48^*d*^	1.99 ± 0.40^*d*^	**4.65 **± 1.11^*d*^	**5.84 **± 1.38^*d*^	**2.07 **± 0.11^*d*^
*ERF*	Ethylene-responsive transcription factor	0.57 ± 0.16^*d*^	1.09 ± 0.44	0.58 ± 0.11^*d*^	1.32 ± 0.25^*d*^	0.67 ± 0.15^*d*^	0.81 ± 0.30

a Fold induction above 2 (considered overexpression) is indicated in bold.

b The reference compound SA was tested at 2.5 mM.

c Peptides were tested at 125 μM.

d Significant values different from the nontreated control (*P *< 0.05).

### Effect of peptides on bacterial spot of tomato.

**FIG 4:**
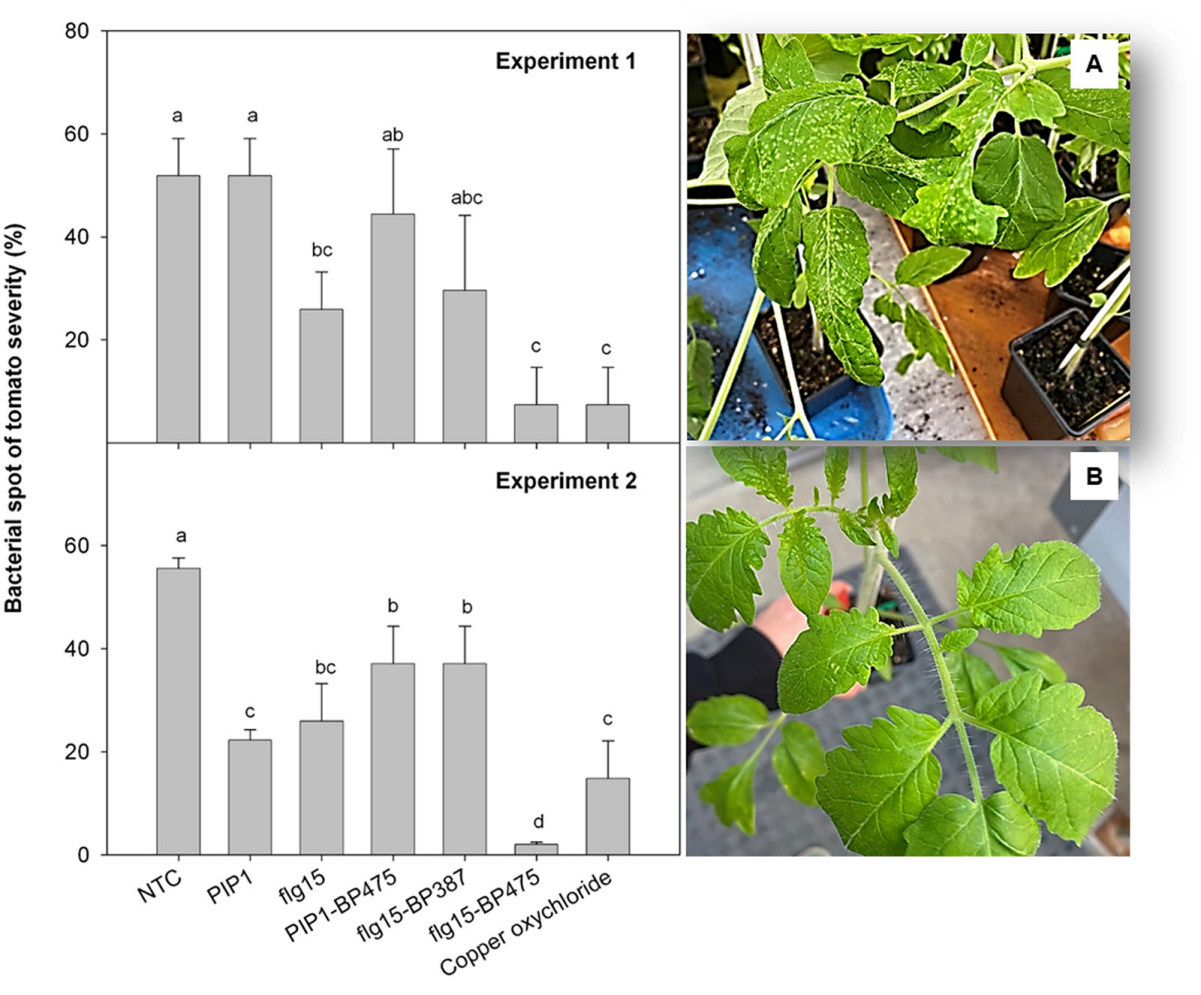
Effect of the peptides on the bacterial spot of tomato plant severity. Two independent experiments were performed. Disease severity was evaluated in tomato plants 13 days after pathogen inoculation. A nontreated control (NTC) and a treatment with copper oxychloride were included as references. Values correspond to the mean disease severity of three replicates of three plants per each treatment, and error bars represent the confidence interval (α = 0.05). Means sharing the same letters are not significantly different (*P < *0.05) according to Tukey’s test. The photographs show the symptoms observed in nontreated (A) and flg15-BP475-treated (B) plants.

The effect of the peptide conjugates flg15-BP387, flg15-BP475, and PIP1-BP475 and the monomers flg15 and PIP1 to reduce bacterial spot infection, caused by *X. axonopodis* pv. vesicatoria, was tested in potted tomato plant assays (Fig. 4). Treatment with flg15 significantly reduced the severity of bacterial spot of tomato compared to the nontreated control in both experiments. In contrast, treatment with PIP1 only reduced the severity of the disease in one of the experiments. Remarkably, the peptide conjugate flg15-BP475 decreased the severity of the disease compared to treatment with flg15, this reduction being significant in one of the experiments. Moreover, treatment with flg15-BP475 showed the same or more efficacy in reducing disease infection than treatment with copper oxychloride. Regarding the treatments with the peptide conjugates flg15-BP387 and PIP1-BP475, they showed the same efficacy in reducing the disease as their respective monomers flg15 and PIP1, except for PIP1-BP475 in one of the experiments.

### Structural characterization.

Since peptide flg15-BP475 showed the best activity, its structure was studied by means of nuclear magnetic resonance (NMR) spectroscopy in the presence of 2,2,2-trifluoroethanol-d_3_. As previously reported, the addition of 2,2,2-trifluoroethanol-d_3_ induces the formation of a secondary structure (72).

**FIG 5:**
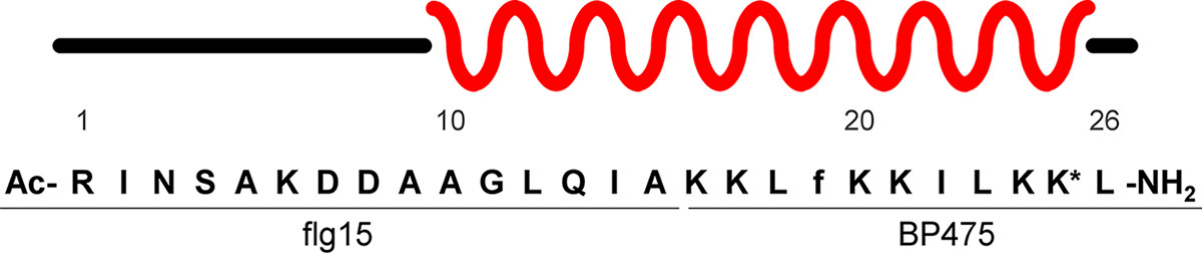
Schematic representation of the secondary structure of flg15-BP475. The red curve marks the helical region, while the black line assigns residues in a random coil. The asterisk refers to the butanoyl-derivatized lysine.

The combination of nuclear Overhauser effect (NOE) correlations, Hα_(i)_-HN_(i+1)_ interresidue correlations, and total correlation spectroscopy (TOCSY) Hα_(i)_-HN_(i)_ intraresidue correlations provided the confirmation of the primary structure and the full assignment of ^1^H, ^13^C, and ^15^N signals (Table SE). Chemical shift index analysis allowed the elucidation of the secondary structure (Fig. 5). It was observed that residues 10 to 25 adopt an α-helical conformation.

## DISCUSSION

Peptides have been described as a promising alternative to traditional treatments to combat plant pathogens. Typically, these peptides either display antimicrobial activity or induce the expression of plant defense genes. In the present study, we designed and evaluated the biological activity of a collection of peptide conjugates resulting from the conjugation of an antimicrobial peptide (BP16, BP100, BP143, KSL-W, BP387, or BP475) and a plant defense elicitor peptide (flg15, BP13, Pep13, or PIP1). The effect of bearing the antimicrobial sequence at the N- or C-terminus of the defense elicitor peptide was analyzed. Structural and physicochemical properties known to be crucial for the activity and selectivity of antimicrobial peptides, such as the net positive charge, the hydrophobicity, and the amphipathicity, were also evaluated.

In general, the peptide conjugates displayed high antibacterial activity against the pathogens tested; 17 out of 30 sequences exhibited MIC values of <12.5 μM against at least 4 pathogens. In agreement with previous results, they were more active against the three *Xanthomonas* species than against E. amylovora and the two Pseudomonas species. These different levels of susceptibility of bacteria to peptides have been attributed to variation in the components of the plasma membranes of the target microorganism, e.g., charge and lipid composition, which would influence electrostatic attraction of cationic peptides to the negatively charged lipids on the outer leaflet of the membranes (45, 47). However, we have not further studied this fact because it deserves biochemical and conformational analysis.

The antibacterial activity clearly depends on the peptide monomers present in the sequence of the peptide conjugates. Sequences incorporating the plant defense elicitor BP13 had low activity, while those derived from flg15, Pep13, and PIP1 exhibited high activity except when containing the antimicrobial peptide KSL-W. Furthermore, the antibacterial activity of the antimicrobial monomers BP16, BP100, and BP143 remarkably increased when conjugated to the elicitor peptide Pep13. The resulting peptide conjugates were, in general, more active than the corresponding antimicrobial peptide monomer, especially against *X. axonopodis* pv. vesicatoria, X. arboricola pv. pruni, and P. syringae pv. actinidiae.

The acylation of a peptide sequence is regarded as a means of increasing membrane affinity and, consequently, antimicrobial activity, since the acyl chain may act as a membrane anchor (47, 48, 73). However, in the present study, the incorporation of a fatty acid chain at the side chain of a Lys residue in BP100 did not lead to an improvement of the antibacterial activity. Thus, flg15-BP100 and BP100-flg15 showed similar activity to that of the corresponding acylated peptide conjugates flg15-BP387 and BP387-flg15. In addition, the substitution of an l-amino acid with its d-isomer has been described to result in peptides that maintain the antimicrobial activity but with increased proteolytic stability (46, 74). Accordingly, the activity of peptides with a d-Phe BP13-BP143, BP143-BP13, Pep13-BP143, and BP143-Pep13 was also similar to that of the corresponding peptide conjugates bearing BP100, with only l-amino acids. Interestingly, the peptide conjugate flg15-BP475, which contains both an acyl chain and a d-amino acid, displayed enhanced antimicrobial activity compared to flg15-BP100 and flg15-BP387.

The order of the monomers in the sequence of the conjugates also influenced the antibacterial activity. In particular, the activity of conjugates containing flg15 and Pep13 was, in general, higher against P. syringae pv. actinidiae and P. syringae pv. syringae when these monomers were at the C-terminus. In contrast, higher activity against *X. axonopodis* pv. vesicatoria and X. arboricola pv. pruni was observed when these peptides reported as defense elicitors were at the N-terminus. The difference in activity between the two conjugates resulting from the combination of two peptides has also been reported in other studies, although this effect has been scarcely analyzed (33, 41–44). In particular, we have observed this distinct activity for peptide conjugates resulting from the combination of BP100 with cecropin A (25–37). In this case, a conjugate containing BP100 at the N-terminus was significantly more active against plant-pathogenic bacteria than that with BP100 at the C-terminus (41).

The monomers present in the sequence of the peptide conjugate, as well as the order of their conjugation, have an important influence on the hemolytic activity. Peptide conjugates containing BP13 were the most hemolytic. In fact, the conjugation of BP13 with BP16, BP100, BP143, or KSLW resulted in significantly higher hemolysis than that of the corresponding monomers. Among the other derivatives, 16 peptide conjugates had low hemolysis (≤30% at 375 μM), some of them exhibiting lower hemolysis than the corresponding monomers. Regarding the order of the monomers in the sequence, in general, peptide conjugates incorporating flg15 or Pep13 at the N-terminus were more hemolytic than those bearing these monomers at the C-terminus. PIP1-BP475 and BP475-PIP1 followed the opposite trend, with the conjugate containing the elicitor peptide PIP1 at the N-terminus being the least hemolytic. In contrast, despite it having been reported that the presence of a d-amino acid decreases the hemolysis (46, 48), in the present work this trend has only been observed for conjugates Pep13-BP143 and BP143-Pep13 that are less hemolytic than their l-counterparts.

The above-described results can be related to the most important factors that govern the activity and selectivity of antimicrobial peptides, such as cationicity, hydrophobicity, and amphipathicity. Concerning cationicity, the best peptide conjugates have a positive charge ranging from +5 to +7 (Pep13-BP16, BP16-Pep13, Pep13-BP100, BP100-Pep13, Pep13-BP143, BP143-Pep13, BP475-PIP1, PIP1-BP475, and flg15-BP475), and those sequences with a charge higher than +7 are, in general, poorly active (BP13 conjugates). These results are in agreement with the general trend reported for antimicrobial peptides, demonstrating that there is a threshold value for optimized cationicity (usually +6 or +7) over which an increase in positive charge leads to a loss of antimicrobial activity (22, 75). Regarding hemolytic activity, no relationship can be inferred between hemolysis and charge for conjugates with a net charge ranging from +4 to +7, and highly cationic conjugates (+8 to +10, BP13 conjugates) are also highly hemolytic. These data do not correlate with previous reports pointing out the contribution of cationicity to cell selectivity due to the higher negative charge of bacterial membranes compared to the eukaryotic cell membrane (22).

A correlation was observed between hydrophobicity and amphipathicity, which could also be related to the antibacterial and hemolytic activities. Hydrophobicity was evaluated with the HPLC retention time and GRAVY values, whereas amphipathicity was quantified by the hydrophobic moment. In particular, an increase of the retention time clearly correlated with an increase of the hydrophobic moment. Analysis of all these parameters pointed out that, in general, the best peptides showed retention times between 5 and 6 min, negative GRAVY values, and a hydrophobic moment within the range of 0.2 to 0.5. Those peptide conjugates with retention times above 7 min, positive GRAVY values, and a hydrophobic moment above 0.8 were poorly active and highly hemolytic (BP13-BP16, BP16-BP13, BP13-BP100, BP100-BP13, BP13-BP143, and BP143-BP13). In addition, conjugates with the lowest amphipathic character also displayed low antibacterial and hemolytic activities (flg15-KSLW, KSLW-flg15, KSLW-PIP1, and PIP1-KSLW). In fact, previous studies have reported that, within a certain range, an increase of the hydrophobicity and amphipathicity of peptides promotes an increase of the antibacterial activity that is also usually associated with an increase of the hemolysis (29, 75). Above this range, antimicrobial peptides tend to aggregate, resulting in a decrease of the antibacterial activity and a further increase of the hemolysis.

It is worth mentioning that the order of the conjugation influenced both the hydrophobicity and amphipathicity of the resulting peptide. Except for conjugates containing PIP1, the sequences bearing the elicitor peptide at the N-terminus had a higher hydrophobic and amphipathic character than those incorporating this peptide at the C-terminus, as shown for the higher retention time and hydrophobic moment. These parameters could explain that peptide conjugates incorporating flg15 and Pep13 at the N-terminus display higher hemolysis. Regarding antibacterial activity, the relationship between both hydrophobicity and amphipathicity, and activity depended on the pathogen. Thus, against *X. axonopodis* pv. vesicatoria and *X. axonopodis* pv. pruni, higher hydrophobicity and amphipathicity are related to higher activity, being peptide conjugates incorporating flg15 and Pep13 at the N-terminus more active. The opposite trend is observed against the two Pseudomonas species; in this case higher activity was obtained for conjugates containing these elicitor peptides at the C-terminus.

Peptide conjugate flg15-BP475, incorporating a plant defense elicitor peptide at the N-terminus and an antimicrobial peptide at the C-terminus, displays a dual activity. This conjugate is one of the sequences with the best *in vitro* biological profile and shows a similar or even higher efficacy than copper oxychloride in reducing bacterial spot infection caused by *X. axonopodis* pv. vesicatoria in potted tomato plants in one of the two experiments. From the overall biological activity results, it can be inferred that this high activity *in planta* is due to the capacity of this peptide conjugate to overexpress plant defense genes and to its high antibacterial activity. Peptide conjugate flg15-BP475 induced the expression of the same genes as flg15 and as the conjugate flg15-BP387. Moreover, the higher activity of flg15-BP475 *in planta* compared to flg15-BP387 could be related to its higher antibacterial activity *in vitro*. Interestingly, flg15-BP475 displayed similar or improved biological properties compared to our previous reported bifunctional peptide conjugates flg15-BP16 and BP178 (44, 71). On the one hand, flg15-BP475 exhibits higher *in vitro* antibacterial activity than flg15-BP16 against *Xanthomonas*. On the other hand, unlike BP178, flg15-BP475 significantly decreased the severity of bacterial spot infection of tomato plants compared to flg15 in one of the experiments. In addition, flg15-BP475 induces overexpression of genes related to plant defense responses that is similar to that of flg15-BP16 and BP178. Thus, flg15-BP475 could be considered a promising bifunctional peptide to control plant diseases.

The characterization by NMR of the secondary structure of flg15-BP475 in the presence of CF_3_CD_2_OD showed that residues 10 to 25 fold into an α-helix. These results are in agreement with previous studies of BP475 and its analog BP100 that pointed out that both peptides adopt an α-helical structure at their C-terminus. This structure has been demonstrated to be crucial for the antibacterial activity of these peptides because it facilitates their insertion into the hydrophobic core of the membrane bilayer (76).

In conclusion, the conjugation of a peptide defense elicitor and an antimicrobial peptide constitutes a good strategy to obtain new peptides with activity against plant-pathogenic bacteria, especially against *Xanthomonas* species. The monomers that are conjugated, as well as the order of their conjugation, have an important influence on the antibacterial and hemolytic activity. The presence of the plant defense elicitors flg15, Pep13, or PIP1 in the sequence of the peptide conjugates leads to high antibacterial activity and low hemolysis. A delicate balance among the structural and physicochemical parameters is required for an optimal biological profile: in particular, a positive charge ranging from +5 to +7 and a moderate hydrophobic moment/amphipatic character. Moreover, the presence of flg15 or Pep13 at the N-terminus of the sequence of the peptide conjugates leads to a high *in vitro* activity against *X. axonopodis* pv. vesicatoria and X. arboricola pv. pruni. Peptide conjugate flg15-BP475 has proven to be a bifunctional peptide able to reduce bacterial spot infection caused by *X. axonopodis* pv. vesicatoria in potted tomato plants through a dual mechanism of action consisting of antimicrobial activity and the induction of the expression of plant defense genes. Its efficacy is similar to or even higher than that of copper oxychloride. In addition, NMR experiments showed that the 16 C-terminal amino acids of flg15-BP475 adopt an α-helix, which could be crucial for its biological activity. Thus, this study provides features that can be useful for the design of new peptide conjugates active against plant pathogens.

## MATERIALS AND METHODS

### General methods.

Manual peptide synthesis was performed in polypropylene syringes (2 or 5 mL) fitted with a polyethylene porous disk. Solvents and soluble reagents were removed by suction. Most chemicals were purchased from the commercial suppliers Merck (Madrid, Spain), Iris Biotech GmbH (Marktredwitz, Germany), Scharlab (Sentmenat, Spain), Carlo Erba Reagents (Sabadell, Spain), or PanReac (Castellar del Vallès, Spain) and used without further purification.

Peptides were analyzed under standard analytical HPLC conditions. Method A: peptides were analyzed with a Dionex liquid chromatography instrument composed of a UV/Vis Dionex UVD170U detector, a P680 Dionex pump, an ASI-100 Dionex automatic injector, and Chromeleon 6.60 software. Detection was performed at a wavelength of 220 nm. Solvent A was 0.1% aqueous trifluoroacetic acid (TFA), and solvent B was 0.1% TFA in CH_3_CN. Analyses were carried out with a Kromasil 100 C_18_ (4.6 mm × 40 mm, 3 μm) column with a linear gradient of 2 to 100% B over 7 min at a flow rate of 1 mL/min. Method B: peptides were analyzed with a 1260 Infinity II liquid chromatography instrument (Agilent Technologies) composed of a diode array detector HS, a quaternary pump VL, a 1260 vial sampler, and OpenLab CDS ChemStation software. Analyses were carried out with a Kromasil 100 C_18_ (4.6 mm × 40 mm, 3 μm) column with a linear gradient of 2 to 100% B over 12 min at a flow rate of 1 mL/min.

All purifications were performed on a CombiFlash Rf200 automated flash chromatography system using a RediSep Rf Gold reversed-phase column packed with high-performance C_18_-derivatized silica.

Electrospray ionization-mass spectrometry (ESI-MS) analyses were performed at the Serveis Tècnics de Recerca of the University of Girona with an Esquire 6000 ESI ion Trap liquid chromatography/mass spectrometry (LC/MS; Bruker Daltonics) instrument equipped with an electrospray ion source. The instrument was operated in the positive ESI(+) ion mode. Samples (5 μL) were introduced into the mass spectrometer ion source directly through an HPLC autosampler. The mobile phase (80:20 CH_3_CN/H_2_O at a flow rate of 100 μL/min) was delivered by a 1200 Series HPLC pump (Agilent). Nitrogen was employed as both the drying and nebulizing gas.

High-resolution mass spectrometry (HRMS) was recorded on a Bruker MicroTof-QII instrument using an ESI ionization source at the Serveis Tècnics de Recerca of the University of Girona. Samples were introduced into the mass spectrometer ion source by direct infusion using a syringe pump and were externally calibrated using sodium formate. The instrument was operated in the positive ion mode.

### Synthesis of peptide monomers.

Peptides flg15, BP13, Pep13, PIP1, BP16, BP100, BP143, and KSL-W and lipopeptides BP387 and BP475 were synthesized following a solid-phase approach as previously described (45–49).

### Synthesis of peptide conjugates and sequence analysis.

The synthesis of peptide conjugates was carried out manually on solid phase in polypropylene syringes equipped with a polyethylene filter. An orthogonal Fmoc/*t*Bu strategy was followed. ChemMatrix resin (0.69 mmol/g) was used as the solid support. Fmoc-Ala-OH, Fmoc-Arg(Pbf)-OH, Fmoc-Asn(Trt)-OH, Fmoc-Asp(O*t*Bu)-OH, Fmoc-Glu(O*t*Bu)-OH, Fmoc-Gln(Trt)-OH, Fmoc-Gly-OH, Fmoc-His(Trt)-OH, Fmoc-Ile-OH, Fmoc-Leu-OH, Fmoc-Lys(Boc)-OH, Fmoc-Lys(ivDde)-OH, Fmoc-Phe-OH, Fmoc-d-Phe-OH, Fmoc-Pro-OH, Fmoc-Ser(*t*Bu)-OH, Fmoc-Thr(*t*Bu)-OH, Fmoc-Trp(Boc)-OH, Fmoc-Tyr(*t*Bu)-OH, and Fmoc-Val-OH were used as amino acid derivatives. An Fmoc-Rink-amide linker was employed to prepare peptide conjugates with a C-terminal amide and a linker 3-(4-hydroxymethylphenoxy)propionic acid (PAC) for peptide conjugates with a C-terminal carboxylic acid.

The ChemMatrix resin was swollen with CH_3_OH (2 × 1 min), *N*,*N*-dimethylformamide (DMF) (2 × 1 min), CH_2_Cl_2_ (2 × 1 min), CH_2_Cl_2_/TFA (99:1; 3 × 1 min), CH_2_Cl_2_/*N*,*N′*-diisopropylethylamine (DIEA) (19:1; 3 × 1 min), CH_2_Cl_2_ (3 × 1 min), and DMF (6 × 1 min). Then, the resin was treated with the corresponding linker (4 equivalent [equiv]), ethyl 2-ciano-2-(hydroxyimino)acetate (Oxyma) (4 equiv), and *N*,*N*-diisopropylcarbodiimide (DIPCDI) (4 equiv) in DMF overnight. After this time, the resin was washed with DMF (6 × 1 min) and CH_2_Cl_2_ (3 × 1 min), and the completion of the reaction was checked with the Kaiser test (77).

The peptide sequence was synthesized through sequential Fmoc removal and coupling steps of the corresponding protected amino acids. The Fmoc removal step was carried out with piperidine/DMF (3:7; 1 × 2 min and 2 × 10 min). The coupling of the first amino acid (5 equiv) onto a PAC-ChemMatrix resin was performed in the presence of DIPCDI (5 equiv), 4-dimethylaminopyridine (DMAP) (0.5 equiv), and DIEA (1 equiv) in DMF at room temperature for 2 h with stirring. This treatment was repeated twice, and then the resin was washed with DMF (6 × 1 min) and CH_2_Cl_2_ (3 × 1 min) and dried with diethyl ether (3 × 2 min). The reaction was monitored using an Fmoc test. Then, the resin was acetylated with acetic anhydride/pyridine/CH_2_Cl_2_ (1.35:1.35:18; 2 × 30 min) and washed with DMF (3 × 1 min) and CH_2_Cl_2_ (3 × 1 min). Coupling of the other amino acids was carried out by treating the resin with the corresponding protected amino acid (4 equiv), Oxyma (4 equiv), and DIPCDI (4 equiv) in DMF with stirring for 3 h at room temperature. After each coupling and Fmoc removal step, the resin was washed with DMF (6 × 1 min) and CH_2_Cl_2_ (3 × 1 min), and the reactions were monitored with the Kaiser or chloranil test (77, 78). After the coupling of the fifth amino acid, *N*-methyl-2-pyrrolidinone (NMP) was employed instead of DMF. Once the peptide elongation was completed, the peptidyl resin was treated with piperidine/NMP (3:7; 2 + 10 min), washed with NMP (6 × 1 min) and CH_2_Cl_2_ (2 × 1 min), and air dried.

In the case of the peptide conjugates incorporating a side chain acylated lysine, this residue was incorporated as Fmoc-Lys(ivDde)-OH [ivDde = 1-(4,4-dimethyl-2,6-dioxocyclohex-1-ylidine)-3-methylbutyl]. After peptide elongation as described above, the N-terminal deprotected resin was acetylated with acetic anhydride/pyridine/CH_2_Cl_2_ (1:1:1; 2 × 30 min), washed with NMP (6 × 1 min) and CH_2_Cl_2_ (6 × 1 min), and air dried. Completion of the reaction was checked with the Kaiser test (77). The resulting resin was treated with NH_2_NH_2_ · H_2_O/NMP (2:98; 10 × 20 min) with stirring and washed with NMP (2 × 1 min), CH_2_Cl_2_ (2 × 1 min), CH_3_OH (2 × 1 min), and NMP (2 × 1 min). Then, the resin was acylated by treatment with butyric acid (3 equiv), DIPCDI (3 equiv), and Oxyma (3 equiv) in NMP with stirring overnight. The resin was then washed with NMP (6 × 1 min) and CH_2_Cl_2_ (6 × 1 min) and air dried. Completion of the reaction was checked with the Kaiser test (77).

Finally, each resulting peptidyl resin was treated with TFA/H_2_O/triisopropylsilane (TIS) (95:2.5:2.5) for 2 h at room temperature. Peptides incorporating a tryptophan residue were cleaved with TFA/phenol/H_2_O/TIS (92.5:2.5:2.5:2.5). Following TFA evaporation and diethyl ether extraction, the crude peptide conjugate was purified by reverse-phase column chromatography with a CombiFlash Rf system, lyophilized, analyzed by HPLC, and characterized by mass spectrometry.

GRAVY was calculated using the program ProtParam (Expasy Proteomics Server) (https://web.expasy.org/protparam/) (79). Charge, hydrophobic ratio (HR), and hydrophobic moment (<μH>) were obtained from HeliQuest (https://heliquest.ipmc.cnrs.fr/cgi-bin/ComputParams.py). α-Helical wheel projections of peptide conjugates are included in the supplemental material (section 4).

### Bacterial strains and growth conditions.

The following plant-pathogenic bacterial strains were used: Erwinia amylovora PMV6076 (Institut National de la Recherche Agronomique, Angers, France), Pseudomonas syringae pv. syringae EPS94 (Institut de Tecnologia Agroalimentària, University of Girona, Spain), Xanthomonas axonopodis pv. vesicatoria 2133-2, *X. axonopodis* pv. vesicatoria Xav206 (Xav) (D. F. Ritchie, Department of Plant Pathology, North Carolina State University), Pseudomonas syringae pv. actinidiae Psa3700.1.1, Xanthomonas fragariae Xf349-9A (Instituto Valenciano de Investigaciones Agrarias, Valencia, Spain), and Xanthomonas arboricola pv. pruni CFBP5563 (Collection Française de Bactéries Associées aux Plantes, Angers, France). All bacteria except for *X. fragariae* were stored in Luria Bertani (LB) broth supplemented with glycerol (20%) and maintained at −80°C. For *X. fragariae*, medium B (80) was used instead of LB. E. amylovora, X. arboricola pv. pruni, P. syringae pv. syringae, and P. syringae pv. actinidiae were scraped from the agar medium after growing for 24 h at 25°C, and *X. axonopodis* pv. vesicatoria and *X. fragariae*, after growing for 48 h at 25°C. The cell material was suspended in sterile water to obtain a suspension of 10^8^ CFU mL^−1^.

### Antibacterial activity.

Lyophilized peptides were solubilized in sterile Milli-Q water to a final concentration of 1 mM and filter-sterilized through a 0.22-μm pore filter. For MIC assessment, dilutions of the compounds were made to obtain a stock concentration of 250, 125, 62, 31, 16, and 8 μM. Then, 20 μL of each dilution were mixed in a microtiter plate well with 20 μL of the corresponding suspension of the bacterial indicator, 160 μL of Trypticase soy broth (TSB) (bioMérieux, France) to a total volume of 200 μL. Three replicates for each combination of strain, compound, and concentration were used. Microbial growth was determined by optical density measurement at 600 nm (Bioscreen C; Labsystem, Helsinki, Finland). Microplates were incubated at 25°C with 20 s of shaking before hourly absorbance measurement for 48 h. The experiment was repeated twice. The MIC was taken as the lowest compound concentration with no growth at the end of the experiment.

### Hemolytic activity.

The hemolytic activity of the compounds was evaluated by determining hemoglobin release from erythrocyte suspensions of horse blood (5% vol/vol) (Oxoid) as previously described (45). Blood was centrifuged at 6000g for 5 min, washed three times with TRIS buffer (10 mM TRIS, 150 mM NaCl, pH 7.2) and diluted 10 times. Compounds were solubilized in TRIS buffer at 750, 500, 300, and 100 μM and mixed with horse erythrocytes (1:1 vol/vol). The mixture was incubated with continuous shaking for 1 h at 37°C. Then, the tubes were centrifuged at 3500g for 10 min, 80-μL aliquots of the supernatant were transferred to 100-well microplates (Bioscreen) and diluted with 80 μL water, and the absorbance measured at 540 nm (Bioscreen). Complete hemolysis was obtained by the addition of melittin at 100 μM (Sigma-Aldrich Corporation, Madrid, Spain). The percentage of hemolysis (*H*) was calculated using the equation *H *= 100 × ([*Op* − *Ob*]/[*Om* − *Ob*]), where *Op* is the density for a given compound concentration, *Ob* is that for the buffer, and *Om* is that for the melittin positive control.

### Effect of peptide infiltration on tobacco leaves.

Peptide conjugates were evaluated for their effect on infiltration on tobacco leaves as described previously (41). Peptide solutions of 50, 150, and 250 μM were infiltrated (100 μL) into the mesophylls of fully expanded tobacco leaves. Infiltrations were carried out in a single leaf, and for each peptide and dose, at least three leaves randomly distributed in different plants were inoculated. Control infiltrations with water (negative control) or melittin (positive control) at the same molar concentration were performed. The appearance of symptoms on the leaves was followed for 48 h after infiltration and measured as lesion diameter.

### Plant host and greenhouse conditions.

Seeds of tomato cv. Rio Grande plants were sown in hydroponic seed plugs (rockwool) and germinated and grown in an environmentally controlled greenhouse at 25 ± 2°C (day) and 18 ± 2°C (night), with a minimum relative humidity of 60% and with a photoperiod of 16 h light and 8 h dark. Then, 2-week-old seedlings (two cotyledons) were transplanted into rockwool plugs (7.5 × 7.5 × 6.5 cm; Gordan Iberica, Spain). Tomato plants were maintained until use under the same environmental conditions.

### Effect of peptide treatment on induction of defense gene expression of tomato plants.

The 2-week-old tomato plants cv. Rio Grande were sprayed with aqueous solutions of the peptides flg15-BP387, flg15-BP475, PIP1-BP475, flg15, and PIP1 at 125 μM until the runoff point. Salicylic acid (SA) was applied at 2.5 mM as a positive control. Water-sprayed plants were used as nontreated controls (NTC). The experimental design consisted of three replicates of three plants per treatment. Leaf samples were collected 24 h after product application and processed to extract RNA for reverse transcriptase quantitative PCR (RT-qPCR) assays. Plant material was roughly ground in liquid nitrogen and was transferred into tubes with two glass beads. Afterward, the frozen samples were finely ground using the TissueLyser II system (Qiagen, Hilden, Germany) at a frequency of 30 Hz for 1 min. Total RNA was extracted from leaves (100 mg) using PureLink plant RNA reagent (Invitrogen, Life Technologies) according to the manufacturer’s manual. The RNA was solubilized in RNase-free water (Ambion, Thermo Fisher Scientific, USA) and was routinely subjected to DNase treatment (Ambion Turbo DNA-free; Invitrogen Life Technologies, Carlsbad, CA, USA) to remove any contaminant DNA. In each step, the RNA was quantified using a NanoDrop ND-1000 spectrophotometer (Nanodrop Technologies, Wilmington, DE, USA). First-strand cDNA was generated from leaf RNA using reverse transcriptase (high-capacity cDNA reverse transcription kit; Applied Biosystems, Foster City, CA, USA) according to the manufacturer’s manual.

To test gene defense induction in the treated tomato plants, a quantitative PCR (qPCR) assay was performed. qPCR was carried out in a fluorometric thermal cycler (7500 Fast real-time PCR system; Applied Biosystems, USA) by using a Mix SYBR green PCR master mix (Applied Biosystems, Foster City, CA, USA) as previously described (49). Melting curve analysis was performed after each amplification to verify amplification specificity. A constitutive gene (actin) was used as a reference control (52), and the following genes implicated in plant defense response were analyzed using the previously described primers: pathogenesis-related protein-1 (*PR1*) and chitinase A (*PR3*) (81), subtilisin-like protease P69G (*PR7*), peroxidase 1 (*PR9*), WRKY3 transcription factor (*WRKY3*), hairpin-induced protein-like (*Harp*), blue-copper-binding protein gene (*BCB*) and ethylene responsive transcription factor (*ERF*) (71), and basic β-1,3-endoglucanase (*GluB*) (52). The primer concentration was 100 nM for all the genes except for the actin gene, for which the concentration was 300 nM. A calibration curve was prepared by cloning the corresponding target sequence in the pSpark cloning vector (Canvax, Córdoba, Spain), which was used to transform Escherichia coli DH5α. Plasmids were purified (Qiagen Iberia, S.L., Madrid, Spain), plasmid copies were quantified, and decimal dilutions were prepared. The efficiency for each standard curve was calculated to check that the efficiency within amplifications was similar. Relative quantification of gene expression was done using the ΔΔ*C_T_* method (82) as previously described (49, 52). Threshold cycle (*C_T_*) values obtained for each repetition treatment were used to estimate the fold change value of the endogenous reference gene (actin) and the target plant defense genes. These results were used to calculate the ratios of the plant defense genes (relative to the actin gene and for all treatments analyzed, including the control plants). The statistical significance of the results for the selected peptides was determined using REST2009 software (Qiagen Iberia, S.L.) (83).

### *In planta* assays.

The efficacy of peptides in bacterial spot of tomato suppression was determined using whole-plant infection assays with tomato plants (cv. Rio grande) inoculated with *X. axonopodis* pv. vesicatoria strain 206. Tomato plants (4 weeks old) were sprayed with a hand sprayer (Herkules; Nuair, Robassomero, Italy) with 6 mL of an aqueous suspension of flg15-BP387, flg15-BP475, PIP1-BP475, flg15, and PIP1 at 125 μM. An NTC was included by spraying plants with water. A treatment with 70% copper oxychloride at 2 g/L (Cuprocol; Syngenta, Spain) was also included as a positive control. After 24 h, treated plants were spray-inoculated until the runoff point (6 mL) with a suspension of *X. axonopodis* pv. vesicatoria at 10^8^ CFU/mL mixed with diatomaceous earth at 1 mg/mL. Plants were incubated under controlled greenhouse conditions (25 ± 2°C, 16 h light; 15 ± 2°C, 8 h dark; 60% relative humidity). The experimental design consisted of three replicates of three plants per treatment. Two independent experiments were performed. Severity of infections was determined per each replicate after 13 days of pathogen inoculation (71). A severity index, ranging from 0 to 4 (0, no symptoms; 1, necrosis/lesions up to 25% of the leaf surface; 2, necrosis/lesions on 25 to 50% of the leaf surface; 3, severe necrosis/lesions on 50 to 75% of the leaf surface; and 4, severe necrosis/lesions on >75% of the leaf surface), was determined for each of the leaves (with 4 to 5 leaflets) of a plant, and it was used to calculate the disease severity index per plant according to the formula


$$S=\sum_{i=1}^{n}[\mathrm{Si}/(n\times 4)]\times 100,$$

where *S* is the severity of the infections per plant, *Si* is the severity index for each leaf, and *n* is the number of leaves measured, which is multiplied by the maximum severity index (i.e., 4). Then, the mean of the severity index of the three plants of each replicate was calculated. The effect of peptide treatments on plant material infection was determined using analysis of variance (ANOVA). Means were separated according to Tukey’s test (*P < *0.05) (SPSS Statistics for Windows version 25.0, 2017 released; IBM Corp., Armonk, NY, USA).

### Structural characterization by NMR spectroscopy.

The structure of conjugated lipopeptide flg15-BP475 was determined by NMR spectroscopy. NMR spectra were acquired at the Serveis Tècnics de Recerca of the University of Girona with an Ultrashield 400 MHz spectrometer equipped with an RT BBI. The following experiments were used to characterize the peptide: 1D ^1^H-NMR, 2D ^1^H-^1^H TOCSY (mixing time, 80 ms), 2D ^1^H-^1^H nuclear Overhauser effect spectroscopy (NOESY) (mixing time, 400 ms), 2D ^1^H-^13^C multiplicity-edited HSQC, 2D ^1^H-^15^N HSQC, and 2D ^1^H-^13^C HSQC-TOCSY. Water suppression was accomplished with excitation sculpting or the Watergate scheme. NMR spectra were processed and analyzed using TopSpin version 3.6.2. All experiments were conducted at 313 K using a Shigemi tube calibrated for D_2_O. To acquire the spectra, 8 mg of peptide were dissolved in 400 μL of 20 mM phosphate buffer at pH 6.5 in H_2_O/D_2_O (90:10) containing 30% 2,2,2-trifluoroethanol-d_3_ to induce the formation of the secondary structure. From NMR assignments, the structural analysis was deduced with the Chemical Shift Index version 3.0 web server (84, 85). ^1^H, ^13^C and ^15^N chemical shifts of flg15-BP475 as well as the NMR spectra are included in the supplemental material (Table SE, section 5).
