# Supplementary material for: Peptide Conjugates Derived from flg15, Pep13, and PIP1 That Are Active against Plant-Pathogenic Bacteria and Trigger Plant Defense Responses
Source: Appl Environ Microbiol. 2022 May 31;88(12):e00574-22. doi: 10.1128/aem.00574-22 (PMC9238401; doi:10.1128/aem.00574-22)

Supporting Information

Peptide conjugates derived from flg15, Pep13 and PIP1 that are active against plant pathogenic bacteria and trigger plant defense responses

Àngel Oliveras, Cristina Camó, Pau Caravaca-Fuentes, Luís Moll, Gerard Riesco-Llach, Sergio Gil-Caballero, Esther Badosa, Anna Bonaterra, Emilio Montesinos, Lidia Feliu,* Marta Planas*

Table of contents:

1. Antibacterial activity, hemolysis and phytotoxicity of peptide conjugates............................2
2. Synthesis of peptide conjugates...5
3. Characterization of peptide conjugates: HPLC, ESI-MS and HRMS..................................17
4. α-Helical wheel projections of peptide conjugates 117
5. Structural characterization of flg15-BP475 by NMR..122

1. Antibacterial activity, hemolysis and phytotoxicity of peptide conjugates

Table A. Antibacterial activity (MIC) of the peptide conjugates and of their corresponding monomers

Peptide	Structure[a]	*Ea*[b]	*Xap*[b]	*Xf*[b]	*Xav*[b]	*Psa*[b]	*Pss*[b]
**flg15**	RINSAKDDAAGLQIA-OH	>100	>100	>100	>100	>100	>100
**BP13**	FKLFKKILKVL-NH$_2$	25-50	6.2-12.5	25-50	50-100	3.1-6.2	12.5-25
**Pep13**	VWNQPVRGFKVYE-OH	>50	>50	>50	>50	>50	>50
**PIP1**	YGIHTH-NH$_2$	>50	>50	>50	>50	>50	>50
**BP16**	KKLFKKILKKL-NH$_2$	25-50	6.2-12.5	12.5-25	12.5-25	3.1-6.2	6.2-12.5
**BP100**	KKLFKKILKYL-NH$_2$	3.1-6.2	1.6-3.1	3.1-6.2	3.1-6.2	1.6-3.1	3.1-6.2
**BP143**	KKLfKKILKYL-NH$_2$	3.1-6.2	6.2-12.5	12.5-25	3.1-6.2	6.2-12.5	3.1-6.2
**BP387**	Ac-KKLFKKIK(COC$_3$H$_7$)KYL-NH$_2$	3.1-6.2	3.1-6.2	1.6-3.1	1.6-3.1	3.1-6.2	3.1-6.2
**BP475**	Ac-KKLfKKILKK(COC$_3$H$_7$)L-NH$_2$	3.1-6.2	0.8-1.6	1.6-3.1	1.6-3.1	1.6-3.1	3.1-6.2
**KSL-W**	KKVVFWVKFK-NH$_2$	6.2-12.5	0.8-1.6	3.1-6.2	6.2-12.5	1.6-3.1	6.2-12.5
**flg15-BP16**	RINSAKDDAAGLQIA-KKLFKKILKKL-NH$_2$	3.1-6.2	3.1-6.2	1.6-3.1	1.6-3.1	3.1-6.2	6.2-12.5
**BP16-flg15**	KKLFKKILKKL-RINSAKDDAAGLQIA-OH	>50	25-50	12.5-25	3.1-6.2	12.5-25	12.5-25
**flg15-BP100**	RINSAKDDAAGLQIA-KKLFKKILKYL-NH$_2$	6.2-12.5	0.8-1.6	6.2-12.5	0.8-1.6	3.1-1.6	12.5-25
**BP100-flg15**	KKLFKKILKYL-RINSAKDDAAGLQIA-OH	6.2-12.5	1.6-3.1	6.2-12.5	3.1-6.2	1.6-3.1	3.1-6.2
**flg15-BP387**	Ac-RINSAKDDAAGLQIA-KKLFKKIK(COC$_3$H$_7$)KYL-NH$_2$	12.5-25	0.8-1.6	3.1-6.2	0.8-1.6	12.5-25	12.5-25
**BP387-flg15**	Ac-KKLFKKIK(COC$_3$H$_7$)KYL-RINSAKDDAAGLQIA-OH	25-50	1.6-3.1	6.2-12.5	3.1-6.2	6.2-12.5	3.1-6.2
**flg15-BP475**	Ac-RINSAKDDAAGLQIA-KKLfKKILKK(COC$_3$H$_7$)L-NH$_2$	3.1-6.2	<0.8	6.2-12.5	<0.8	3.1-6.2	12.5-25
**BP475-flg15**	Ac-KKLfKKILKK(COC$_3$H$_7$)L-RINSAKDDAAGLQIA-OH	12.5-25	1.6-3.1	25-50	3.1-6.2	3.1-6.2	6.2-12.5
**flg15-KSLW**	RINSAKDDAAGLQIA-KKVVFWVKFK-NH$_2$	>50	6.2-12.5	12.5-25	6.2-12.5	>50	>50
**KSLW-flg15**	KKVVFWVKFK-RINSAKDDAAGLQIA-OH	>50	>50	12.5-25	6.2-12.5	>50	>50
**BP13-BP16**	FKLFKKILKVL-KKLFKKILKKL-NH$_2$	>50	6.2-12.5	6.2-12.5	12.5-25	>50	>50
**BP16-BP13**	KKLFKKILKKL-FKLFKKILKVL-NH$_2$	>50	6.2-12.5	12.5-25	1.6-3.1	25-50	12.5-25
**BP13-BP100**	FKLFKKILKVL-KKLFKKILKYL-NH$_2$	>50	12.5-25	6.2-12.5	12.5-25	25-50	>50
**BP100-BP13**	KKLFKKILKYL-FKLFKKILKVL-NH$_2$	>50	12.5-25	6.2-12.5	6.2-12.5	25-50	>50

**BP13-BP143**	FKLFKKILKVL-KKLfKKILKYL-NH_2	>50	6.2-12.5	12.5-25	1.6-3.1	>50	>50
**BP143-BP13**	KKLfKKILKYL-FKLFKKILKVL-NH_2	>50	12.5-25	6.2-12.5	6.2-12.5	25-50	>50
**BP13-KSLW**	FKLFKKILKVL-KKVVFWVKFK-NH_2	>50	1.6-3.1	1.6-3.1	1.6-3.1	6.2-12.5	12.5-25
**KSLW-BP13**	KKVVFWVKFK-FKLFKKILKVL-NH_2	25-50	1.6-3.1	1.6-3.1	3.1-6.2	1.6-3.1	25-50
**Pep13-BP16**	VWNQPVRGFKVYE-KKLFKKILKKL-NH_2	6.2-12.5	0.8-1.6	3.1-6.2	0.8-1.6	1.6-3.1	6.2-12.5
**BP16-Pep13**	KKLFKKILKKL-VWNQPVRGFKVYE-OH	6.2-12.5	0.8-1.6	1.6-3.1	1.6-3.1	1.6-3.1	1.6-3.1
**Pep13-BP100**	VWNQPVRGFKVYE-KKLFKKILKYL-NH_2	6.2-12.5	0.8-1.6	6.2-12.5	0.8-1.6	3.1-6.2	6.2-12.5
**BP100-Pep13**	KKLFKKILKYL-VWNQPVRGFKVYE-OH	0.8-1.6	1.6-3.1	3.1-6.2	1.6-3.1	0.8-1.6	1.6-3.1
**Pep13-BP143**	VWNQPVRGFKVYE-KKLfKKILKYL-NH_2	6.2-12.5	0.8-1.6	3.1-6.2	0.8-1.6	3.1-6.2	6.2-12.5
**BP143-Pep13**	KKLfKKILKYL-VWNQPVRGFKVYE-OH	1.6-3.1	1.6-3.1	6.2-12.5	1.6-3.1	0.8-1.6	1.6-3.1
**Pep13-KSLW**	VWNQPVRGFKVYE-KKVVFWVKFK-NH_2	>50	6.2-12.5	3.1-6.2	12.5-25	>50	>50
**KSLW-Pep13**	KKVVFWVKFK-VWNQPVRGFKVYE-OH	>50	6.2-12.5	3.1-6.2	12.5-25	>50	>50
**PIP1-BP475**	Ac-YGIHTH-KKLfKKILKK(COC_3H_7)L-NH_2	3.1-6.2	<0.8	12.5-25	<0.8	1.6-3.1	3.1-6.2
**BP475-PIP1**	Ac-KKLfKKILKK(COC_3H_7)L-YGIHTH-NH_2	3.1-6.2	0.8-1.6	1.6-3.1	3.1-6.2	1.6-3.1	6.2-12.5
**PIP1-KSLW**	YGIHTH-KKVVFWVKFK-NH_2	12.5-25	3.1-6.2	6.2-12.5	3.1-6.2	6.2-12.5	6.2-12.5
**KSLW-PIP1**	KKVVFWVKFK-YGIHTH-NH_2	>50	3.1-6.2	3.1-6.2	3.1-6.2	>50	>50

[a]COC_3H_7, butanoyl; lower case letters correspond to D-amino acids.

[b]*Ea*, *Erwinia amylovora*; *Xap*, *Xanthomonas arboricola* pv. pruni; *Xf*, *Xanthomonas fragariae*; *Xav*, *Xanthomonas axonopodis* pv. vesicatoria; *Psa*, *Pseudomonas syringae* pv. actinidiae; *Pss*, *Pseudomonas syringae* pv. syringae.

Table B. Hemolytic activity of the peptide conjugates and of their corresponding monomers

Peptide	Structure[a]	50 µM	150 µM	250 µM	375 µM
**flg15**	RINSAKDDAAGLQIA-OH	0 ± 0	0 ± 0	0 ± 0	0 ± 0
**BP13**	FKLFKKILKVL-NH_2	5 ± 0.2	13 ± 2	27 ± 3	42 ± 3
**Pep13**	VWNQPVRGFKVYE-OH	0 ± 0.6	0 ± 0.3	2 ± 0.1	0 ± 0.1
**PIP1**	YGIHTH-NH_2	4 ± 0.8	0.5 ± 0.3	0 ± 2	1 ± 0.1
**BP16**	KKLFKKILKKL-NH_2	2 ± 1	1 ± 0.6	1 ± 0.4	3 ± 3
**BP100**	KKLFKKILKYL-NH_2	4 ± 0.5	7 ± 2	6 ± 0.3	11 ± 0.9
**BP143**	KKLfKKILKYL-NH_2	3 ± 0.6	4 ± 0.6	6 ± 0.3	7 ± 0.3
**BP387**	Ac-KKLFKKIK(COC_3H_7)KYL-NH_2	4 ± 0.9	11 ± 5	14 ± 0.5	18 ± 1
**BP475**	Ac-KKLfKKILKK(COC_3H_7)L-NH_2	0 ± 0	0 ± 0	0 ± 0	0 ± 0
**KSL-W**	KKVVFWVKFK-NH_2	4 ± 1	5 ± 0.2	6 ± 1	20 ± 2
**flg15-BP16**	RINSAKDDAAGLQIA-KKLFKKILKKL-NH_2	2 ± 0.1	3 ± 0.2	4 ± 0.9	5 ± 0.3
**BP16-flg15**	KKLFKKILKKL-RINSAKDDAAGLQIA-OH	0 ± 0.3	1 ± 0.1	0 ± 0.6	1 ± 0.5
**flg15-BP100**	RINSAKDDAAGLQIA-KKLFKKILKYL-NH_2	48 ± 4.1	52 ± 4.3	63 ± 2.4	66 ± 9.2
**BP100-flg15**	KKLFKKILKYL-RINSAKDDAAGLQIA-OH	1 ± 0.9	1 ± 0.8	3 ± 0.0	6 ± 1.6
**flg15-BP387**	Ac-RINSAKDDAAGLQIA-KKLFKKIK(COC_3H_7)KYL-NH_2	5 ± 0.4	15 ± 1.6	26 ± 3.5	39 ± 2.1
**BP387-flg15**	Ac-KKLFKKIK(COC_3H_7)KYL-RINSAKDDAAGLQIA-OH	3 ± 0.3	5 ± 0.9	7 ± 0.2	9 ± 2.5
**flg15-BP475**	Ac-RINSAKDDAAGLQIA-KKLfKKILKK(COC_3H_7)L-NH_2	7 ± 0.6	28 ± 2.3	43 ± 2.7	46 ± 3.6
**BP475-flg15**	Ac-KKLfKKILKK(COC_3H_7)L-RINSAKDDAAGLQIA-OH	1 ± 1.0	0 ± 0.2	1 ± 0.3	1 ± 0.3
**flg15-KSLW**	RINSAKDDAAGLQIA-KKVVFWVKFK-NH_2	0 ± 0.6	1 ± 0.4	1 ± 0.4	0 ± 0.3
**KSLW-flg15**	KKVVFWVKFK-RINSAKDDAAGLQIA-OH	4 ± 1.9	7 ± 3.4	8 ± 0.9	8 ± 2.7
**BP13-BP16**	FKLFKKILKVL-KKLFKKILKKL-NH_2	86 ± 13.2	86 ± 1.6	87 ± 1.2	93 ± 2.3
**BP16-BP13**	KKLFKKILKKL-FKLFKKILKVL-NH_2	100 ± 4.0	100 ± 2.3	100 ± 8.5	100 ± 2.1
**BP13-BP100**	FKLFKKILKVL-KKLFKKILKYL-NH_2	100 ± 8.3	100 ± 5.9	99 ± 7.1	100 ± 4.8
**BP100-BP13**	KKLFKKILKYL-FKLFKKILKVL-NH_2	100 ± 9.4	100 ± 5.5	98 ± 3.3	99 ± 3.7
**BP13-BP143**	FKLFKKILKVL-KKLfKKILKYL-NH_2	100 ± 0.6	100 ± 3.7	100 ± 3.2	100 ± 10.6
**BP143-BP13**	KKLfKKILKYL-FKLFKKILKVL-NH_2	100 ± 6.1	100 ± 3.6	100 ± 7.1	100 ± 7.0
**BP13-KSLW**	FKLFKKILKVL-KKVVFWVKFK-NH_2	80 ± 11.2	90 ± 7.3	100 ± 8.4	99 ± 8.4
**KSLW-BP13**	KKVVFWVKFK-FKLFKKILKVL-NH_2	62 ± 8.2	65 ± 5.9	77 ± 12.9	82 ± 10.4
**Pep13-BP16**	VWNQPVRGFKVYE-KKLFKKILKKL-NH_2	2 ± 0.6	3 ± 0.2	4 ± 0.3	4 ± 0.2
**BP16-Pep13**	KKLFKKILKKL-VWNQPVRGFKVYE-OH	1 ± 0.4	1 ± 0.3	1 ± 0.5	2 ± 0.4
**Pep13-BP100**	VWNQPVRGFKVYE-KKLFKKILKYL-NH_2	9 ± 1.0	25 ± 2.4	45 ± 9.0	49 ± 9.2
**BP100-Pep13**	KKLFKKILKYL-VWNQPVRGFKVYE-OH	5 ± 0.3	12 ± 1.1	20 ± 2.4	30 ± 3.2
**Pep13-BP143**	VWNQPVRGFKVYE-KKLfKKILKYL-NH_2	5 ± 0.5	8 ± 0.3	10 ± 0.6	17 ± 0.4
**BP143-Pep13**	KKLfKKILKYL-VWNQPVRGFKVYE-OH	5 ± 1.9	9 ± 0.4	16 ± 1.9	29 ± 0.9
**Pep13-KSLW**	VWNQPVRGFKVYE-KKVVFWVKFK-NH_2	9 ± 2.3	31 ± 3.7	48 ± 3.8	60 ± 1.5
**KSLW-Pep13**	KKVVFWVKFK-VWNQPVRGFKVYE-OH	0 ± 0.3	2 ± 1.0	3 ± 0.3	5 ± 1.0
**PIP1-BP475**	Ac-YGIHTH-KKLfKKILKK(COC_3H_7)L-NH_2	4 ± 0.4	11 ± 0.5	18 ± 0.1	24 ± 0.5
**BP475-PIP1**	Ac-KKLfKKILKK(COC_3H_7)L-YGIHTH-NH_2	9 ± 1.0	18 ± 1.4	26 ± 2.8	39 ± 2.0
**PIP1-KSLW**	YGIHTH-KKVVFWVKFK-NH_2	7 ± 0.8	7 ± 1.1	6 ± 0.4	6 ± 0.4
**KSLW-PIP1**	KKVVFWVKFK-YGIHTH-NH_2	8 ± 1.3	3 ± 2.1	3 ± 1.2	5 ± 4.9

[a]COC_3H_7, butanoyl; lower case letters correspond to D-amino acids

[b]Percent hemolysis at 50, 150, 250 and 375 µM plus confidence interval ($\alpha = 0.05$)

Table C. Effect of the peptide conjugates and of their corresponding mononers on the size of the lesions in infiltrated tobacco leaves (cm)

Peptide	Structure[a]	50 μM	150 μM	250 μM
**flg15**	RINSAKDDAAGLQIA-OH	0 ± 0	0 ± 0	0 ± 0
**BP13**	FKLFKKILKVL-NH_2	0.20 ± 0.24	0.48 ± 0.15	0.96 ± 0.19
**Pep13**	VWNQPVRGFKVYE-OH	0 ± 0	0 ± 0	0 ± 0
**PIP1**	YGIHTH-NH_2	0 ± 0	0 ± 0	0 ± 0
**BP16**	KKLFKKILKKL-NH_2	0.08 ± 0.11	0.16 ± 0.16	0.26 ± 0.08
**BP100**	KKLFKKILKYL-NH_2	0.40 ± 0.03		1.20 ± 0.16
**BP143**	KKLfKKILKYL-NH_2	0.10 ± 0.12	0.37 ± 0.15	0.65 ± 0.11
**BP387**	Ac-KKLFKKIK(COC_3H_7)KYL-NH_2	0.32 ± 0.07	0.84 ± 0.10	0.92 ± 0.01
**BP475**	Ac-KKLfKKILKK(COC_3H_7)L-NH_2	0.50 ± 0.10	0.70 ± 0.20	1.00 ± 0.10
**KSL-W**	KKVVFWVKFK-NH_2	0 ± 0	0.37 ± 0.97	0.62 ± 0.45
**flg15-BP16**	RINSAKDDAAGLQIA-KKLFKKILKKL-NH_2	0.20 ± 0.12	0.49 ± 0.13	0.42 ± 0.11
**BP16-flg15**	KKLFKKILKKL-RINSAKDDAAGLQIA-OH	0 ± 0	0.31 ± 0.10	0.30 ± 0.09
**flg15-BP100**	RINSAKDDAAGLQIA-KKLFKKILKYL-NH_2	0.46 ± 0.10	0.80 ± 0.22	1.02 ± 0.24
**BP100-flg15**	KKLFKKILKYL-RINSAKDDAAGLQIA-OH	0.10 ± 0.12	0.28 ± 0.14	0.36 ± 0.13
**flg15-BP387**	Ac-RINSAKDDAAGLQIA-KKLFKKIK(COC_3H_7)KYL-NH_2	0.07 ± 0.13	0.43 ± 0.13	0.80 ± 0.41
**BP387-flg15**	Ac-KKLFKKIK(COC_3H_7)KYL-RINSAKDDAAGLQIA-OH	0.23 ± 0.24	0.33 ± 0.35	0.93 ± 0.40
**flg15-BP475**	Ac-RINSAKDDAAGLQIA-KKLfKKILKK(COC_3H_7)L-NH_2	0.60 ± 0	1.20 ± 0	1.37 ± 0.06
**BP475-flg15**	Ac-KKLfKKILKK(COC_3H_7)L-RINSAKDDAAGLQIA-OH	0.43 ± 0.13	1.20 ± 0.23	1.23 ± 0.28
**flg15-KSLW**	RINSAKDDAAGLQIA-KKVVFWVKFK-NH_2	0.13 ± 0.13	0.43 ± 0.07	0.83 ± 0.13
**KSLW-flg15**	KKVVFWVKFK-RINSAKDDAAGLQIA-OH	0 ± 0	0.13 ± 0.09	0.26 ± 0.05
**BP13-BP16**	FKLFKKILKVL-KKLFKKILKKL-NH_2	0.52 ± 0.08	1.08 ± 0.21	1.40 ± 0.71
**BP16-BP13**	KKLFKKILKKL-FKLFKKILKVL-NH_2	0.68 ± 0.16	1.15 ± 0.28	1.52 ± 0.32
**BP13-BP100**	FKLFKKILKVL-KKLFKKILKYL-NH_2	0.60 ± 0.30	1.20 ± 0.17	1.30 ± 0.17
**BP100-BP13**	KKLFKKILKYL-FKLFKKILKVL-NH_2	0.73 ± 0.20	1.36 ± 0.23	1.46 ± 0.06
**BP13-BP143**	FKLFKKILKVL-KKLfKKILKYL-NH_2	0.53 ± 0.18	0.88 ± 0.15	1.07 ± 0.37
**BP143-BP13**	KKLfKKILKYL-FKLFKKILKVL-NH_2	0.18 ± 0.10	0.40 ± 0.14	1.14 ± 0.36
**BP13-KSLW**	FKLFKKILKVL-KKVVFWVKFK-NH_2	0.56 ± 0.23	1.00 ± 0.25	0.92 ± 0.36
**KSLW-BP13**	KKVVFWVKFK-FKLFKKILKVL-NH_2	0.52 ± 0.13	0.88 ± 0.15	1.07 ± 0.34
**Pep13-BP16**	VWNQPVRGFKVYE-KKLFKKILKKL-NH_2	0.23 ± 0.07	0.52 ± 0.14	0.93 ± 0.19
**BP16-Pep13**	KKLFKKILKKL-VWNQPVRGFKVYE-OH	0.23 ± 0.13	0.45 ± 0.12	1.02 ± 0.32
**Pep13-BP100**	VWNQPVRGFKVYE-KKLFKKILKYL-NH_2	0.26 ± 0.08	0.56 ± 0.13	0.70 ± 0.15
**BP100-Pep13**	KKLFKKILKYL-VWNQPVRGFKVYE-OH	0.04 ± 0.08	0.32 ± 0.11	0.42 ± 0.10
**Pep13-BP143**	VWNQPVRGFKVYE-KKLfKKILKYL-NH_2	0.16 ± 0.15	0.52 ± 0.14	0.62 ± 0.13
**BP143-Pep13**	KKLfKKILKYL-VWNQPVRGFKVYE-OH	0.10 ± 0.12	0.44 ± 0.16	0.60 ± 0.25
**Pep13-KSLW**	VWNQPVRGFKVYE-KKVVFWVKFK-NH_2	0.35 ± 0.06	0.50 ± 0.11	0.80 ± 0.20
**KSLW-Pep13**	KKVVFWVKFK-VWNQPVRGFKVYE-OH	0.38 ± 0.22	0.48 ± 0.12	0.83 ± 0.22
**PIP1-BP475**	Ac-YGIHTH-KKLfKKILKK(COC_3H_7)L-NH_2	0.47 ± 0.13	1.03 ± 0.06	1.17 ± 0.06
**BP475-PIP1**	Ac-KKLfKKILKK(COC_3H_7)L-YGIHTH-NH_2	0.47 ± 0.06	1.40 ± 0.00	1.67 ± 0.13
**PIP1-KSLW**	YGIHTH-KKVVFWVKFK-NH_2	0 ± 0	0.47 ± 0.24	0.70 ± 0.11
**KSLW-PIP1**	KKVVFWVKFK-YGIHTH-NH_2	0.17 ± 0.17	0.53 ± 0.07	0.97 ± 0.28

[a]COC_3H_7, butanoyl; lower case letters correspond to D-amino acids

[b]Size of the lesions (cm) at 50, 150 and 250 μM plus confidence interval (α = 0.05)

2. Synthesis of peptide conjugates

Arg-Ile-Asn-Ser-Ala-Lys-Asp-Asp-Ala-Ala-Gly-Leu-Gln-Ile-Ala-Lys-Lys-Leu-Phe-Lys-Lys-Ile-Leu-Lys-Lys-Leu-NH_2 (flg15-BP16)

This peptide conjugate was prepared following the procedure described in the manuscript using a Fmoc-Rink-ChemMatrix resin (200 mg, 0.66 mmol/g). Acidolytic cleavage of the resulting resin and purification eluting with H_2O/CH_3CN (68:32) afforded **flg15-BP16** in >99% HPLC purity. HPLC (λ = 220 nm): t_R = 6.94 min (Method A); t_R = 5.89 min (Method B). MS (ESI) *m/z*: 1456.0 $[M + 2H]^{2+}$, 2910.9 $[M + H]^{+}$. HRMS (ESI) *m/z*: calcd for $C_{133}H_{239}N_{39}O_{33}$ $[M + 2H]^{2+}$ 1455.9120, found 1455.9087; calcd for $C_{133}H_{238}N_{39}O_{33}Na$ $[M +H + Na]^{2+}$ 1466.9030, found 1466.9029; calcd for $C_{133}H_{237}N_{39}O_{33}Na_2$ $[M + 2Na]^{2+}$ 1477.8939, found 1477.8917.

Lys-Lys-Leu-Phe-Lys-Lys-Ile-Leu-Lys-Lys-Leu-Arg-Ile-Asn-Ser-Ala-Lys-Asp-Asp-Ala-Ala-Gly-Leu-Gln-Ile-Ala-OH (BP16-flg15)

This peptide conjugate was prepared following the procedure described in the manuscript using a PAC-ChemMatrix resin (200 mg, 0.66 mmol/g). Acidolytic cleavage of the resulting resin and purification eluting with H_2O/CH_3CN (80:20) yielded **BP16-flg15** in >99% HPLC purity. HPLC (λ = 220nm): t_R = 6.04 min (Method A); t_R = 4.59 min (Method B). MS (ESI) *m/z*: 971.4 $[M + 3H]^{3+}$, 986.2 $[M + H + 2Na]^{3+}$, 993.7 $[M + 3Na]^{3+}$, 1456.5 $[M + 2H]^{2+}$, 1467.5 $[M + H + Na]^{2+}$, 1478.9 $[M + 2Na]^{2+}$. HRMS (ESI) *m/z*: calcd for $C_{133}H_{238}N_{38}O_{34}$ $[M + 2H]^{2+}$ 1456.4040, found 1456.4023; calcd for $C_{133}H_{237}N_{38}O_{34}Na$ $[M + H + Na]^{2+}$ 1467.3950, found 1467.3915.

Arg-Ile-Asn-Ser-Ala-Lys-Asp-Asp-Ala-Ala-Gly-Leu-Gln-Ile-Ala-Lys-Lys-Leu-Phe-Lys-Lys-Ile-Leu-Lys-Tyr-Leu-NH_2 (flg15-BP100)

This peptide conjugate was prepared following the procedure described in the manuscript using a Fmoc-Rink-ChemMatrix resin (200 mg, 0.66 mmol/g). Acidolytic cleavage of the resulting resin and purification eluting with H_2O/CH_3CN (67:33) yielded **flg15-BP100** in >99% HPLC purity. HPLC (λ = 220 nm): t_R = 7.02 min (Method A); t_R = 6.29 min (Method B). MS (ESI) *m/z*: 590.0 $[M + 5H]^{5+}$, 737.3 $[M + 4H]^{4+}$, 982.6 $[M + 3H]^{3+}$, 1473.3 $[M + 2H]^{2+}$, 1484.8 $[M + H + Na]^{2+}$, 2944.8 $[M + H]^{+}$, 2966.7 $[M + Na]^{+}$. HRMS (ESI) *m/z*: calcd for $C_{136}H_{237}N_{38}O_{34}$ $[M + 3H]^{3+}$ 982.2656, found 982.2687; calcd for $C_{136}H_{236}N_{38}O_{34}$ $[M + 2H]^{2+}$ 1472.8948, found 1472.8977.

Lys-Lys-Leu-Phe-Lys-Lys-Ile-Leu-Lys-Tyr-Leu-Arg-Ile-Asn-Ser-Ala-Lys-Asp-Asp-Ala-Ala-Gly-Leu-Gln-Ile-Ala-OH (BP100-flg15)

This peptide conjugate was prepared following the procedure described in the manuscript using a PAC-ChemMatrix resin (200 mg, 0.66 mmol/g). Acidolytic cleavage of the resulting resin and purification eluting with H_2O/CH_3CN (68:32) yielded **BP100-flg15** in >99% HPLC purity. HPLC (λ

= 220 nm): t_R = 6.36 min (Method A); t_R = 4.96 min (Method B). MS (ESI) *m/z*: 983.0 $[M + 3H]^{3+}$, 990.6 $[M + 2H + Na]^{3+}$, 1473.9 $[M + 2H]^{2+}$, 1484.9 $[M +H + Na]^{2+}$, 2945.8 $[M + H]^{+}$. HRMS (ESI) *m/z*: calcd for $C_{136}H_{236}N_{37}O_{35}$ $[M + 3H]^{3+}$ 982.5936, found 982.5964; calcd for $C_{136}H_{235}N_{37}O_{35}$ $[M + 2H]^{2+}$ 1473.3868, found 1473.3903.

Ac-Arg-Ile-Asn-Ser-Ala-Lys-Asp-Asp-Ala-Ala-Gly-Leu-Gln-Ile-Ala-Lys-Lys-Leu-Phe-Lys-Lys-Ile-Lys(COC_3H_7)-Lys-Tyr-Leu-NH_2 (flg15-BP387)

This peptide conjugate was prepared following the procedure described in the manuscript using a Fmoc-Rink-ChemMatrix resin (200 mg, 0.66 mmol/g). Acidolytic cleavage of the resulting resin and purification eluting with H_2O/CH_3CN (70:30) afforded **flg15-BP387** in 96% HPLC purity. HPLC (λ = 220 nm): t_R = 6.35 min (Method B). MS (ESI) *m/z*: 615.5 $[M + 5H]^{5+}$, 769.2 $[M + 4H]^{4+}$, 1525.0 $[M + 3H]^{3+}$, 1536.9 $[M + 2H]^{2+}$. HRMS (ESI) *m/z*: calcd for $C_{142}H_{246}N_{39}O_{36}$ $[M + 3H]^{3+}$ 1024.6200, found 1024.6166; calcd for $C_{142}H_{245}N_{39}O_{36}$ $[M + 2H]^{2+}$ 1536.4264, found 1536.4212.

Ac-Lys-Lys-Leu-Phe-Lys-Lys-Ile-Lys(COC_3H_7)-Lys-Tyr-Leu-Arg-Ile-Asn-Ser-Ala-Lys-Asp-Asp-Ala-Ala-Gly-Leu-Gln-Ile-Ala-OH (BP387-flg15)

This peptide conjugate was prepared following the procedure described in the manuscript using a PAC-ChemMatrix resin (200 mg, 0.66 mmol/g). Acidolytic cleavage of the resulting resin and purification eluting with H_2O/CH_3CN (70:30) afforded **BP387-flg15** in 96% HPLC purity. HPLC (λ = 220 nm): t_R = 5.84 min (Method B). MS (ESI) *m/z*: 769.5 $[M + 4H]^{4+}$, 1025.3 $[M + 3H]^{3+}$, 1537.5 $[M + 2H]^{2+}$. HRMS (ESI) *m/z*: calcd for $C_{142}H_{245}N_{38}O_{37}$ $[M + 3H]^{3+}$ 1024.9480, found 1024.9477; calcd for $C_{142}H_{244}N_{38}O_{37}$ $[M + 2H]^{2+}$ 1536.9184, found 1536.9152.

Ac-Arg-Ile-Asn-Ser-Ala-Lys-Asp-Asp-Ala-Ala-Gly-Leu-Gln-Ile-Ala-Lys-Lys-Leu-D-Phe-Lys-Lys-Ile-Leu-Lys-Lys(COC_3H_7)-Leu-NH_2 (flg15-BP475)

This peptide conjugate was prepared following the procedure described in the manuscript using a Fmoc-Rink-ChemMatrix resin (200 mg, 0.66 mmol/g). Acidolytic cleavage of the resulting resin and purification eluting with H_2O/CH_3CN (60:40) afforded **flg15-BP475** in 97% HPLC purity. HPLC (λ = 220 nm): t_R = 6.79 min (Method B). MS (ESI) *m/z*: 605.5 $[M + 5H]^{5+}$, 756.5 $[M + 4H]^{4+}$, 1008.4 $[M + 3H]^{3+}$, 1511.9 $[M + 2H]^{2+}$. HRMS (ESI) *m/z*: calcd for $C_{139}H_{248}N_{39}O_{35}$ $[M + 3H]^{3+}$ 1007.9603, found 1007.9556; calcd for $C_{139}H_{247}N_{39}O_{35}$ $[M + 2H]^{2+}$ 1511.4368, found 1511.4329.

Ac-Lys-Lys-Leu-D-Phe-Lys-Lys-Ile-Leu-Lys-Lys(COC_3H_7)-Leu-Arg-Ile-Asn-Ser-Ala-Lys-Asp-Asp-Ala-Ala-Gly-Leu-Gln-Ile-Ala-OH (BP475-flg15)

This peptide conjugate was prepared following the procedure described in the manuscript using a PAC-ChemMatrix resin (200 mg, 0.66 mmol/g). Acidolytic cleavage of the resulting resin and purification eluting with H_2O/CH_3CN (60:40) afforded **BP475-flg15** in >99% HPLC purity. HPLC

(λ = 220 nm): t_R = 6.03 min (Method B). MS (ESI) *m/z*: 756.8 $[M + 4H]^{4+}$, 1008.7 $[M + 3H]^{3+}$, 1512.4 $[M + 2H]^{2+}$. HRMS (ESI) *m/z*: calcd for $C_{139}H_{247}N_{38}O_{36}$ $[M + 3H]^{3+}$ 1008.2883, found 1008.2867; calcd for $C_{139}H_{246}N_{38}O_{36}$ $[M + 2H]^{2+}$ 1511.9288, found 1511.9271.

Arg-Ile-Asn-Ser-Ala-Lys-Asp-Asp-Ala-Ala-Gly-Leu-Gln-Ile-Ala-Lys-Lys-Val-Val-Phe-Trp-Val-Lys-Phe-Lys-NH_2 (flg15-KSLW)

This peptide conjugate was prepared following the procedure described in the manuscript using a Fmoc-Rink-ChemMatrix resin (200 mg, 0.66 mmol/g). Acidolytic cleavage of the resulting resin and purification eluting with H_2O/CH_3CN (75:25) yielded **flg15-KSLW** in >99% HPLC purity. HPLC (λ = 220 nm): t_R = 6.59 min (Method A); t_R = 5.40 min (Method B). MS (ESI) *m/z*: 1416.3 $[M + 2H]^{2+}$, 1427.2 $[M + H + Na]^{2+}$, 1438.7 $[M + 2Na]^{2+}$. HRMS (ESI) *m/z*: calcd for $C_{132}H_{217}N_{37}O_{32}$ $[M + 2H]^{2+}$ 1416.8254, found 1416.8202; calcd for $C_{132}H_{216}N_{37}O_{32}Na$ $[M + H + Na]^{2+}$ 1427.8164, found 1427.8106; calcd for $C_{132}H_{215}N_{37}O_{32}Na_2$ $[M + 2Na]^{2+}$ 1438.8073, found 1438.8016.

Lys-Lys-Val-Val-Phe-Trp-Val-Lys-Phe-Lys-Arg-Ile-Asn-Ser-Ala-Lys-Asp-Asp-Ala-Ala-Gly-Leu-Gln-Ile-Ala-OH (KSLW-flg15)

This peptide conjugate was prepared following the procedure described in the manuscript using a PAC-ChemMatrix resin (200 mg, 0.66 mmol/g). Acidolytic cleavage of the resulting resin and purification eluting with H_2O/CH_3CN (75:25) yielded **KSLW-flg15** in >99% HPLC purity. HPLC (λ = 220 nm): t_R = 6.47 min (Method A); t_R = 4.75 min (Method B). MS (ESI) *m/z*: 1417.5 $[M + 2H]^{2+}$, 2833.7 $[M + H]^{+}$. HRMS (ESI) *m/z*: calcd for $C_{132}H_{216}N_{36}O_{33}$ $[M + 2H]^{2+}$ 1417.3174, found 1417.3209; calcd for $C_{132}H_{215}N_{36}O_{33}Na$ $[M + H + Na]^{2+}$ 1428.3084, found 1428.3120.

Phe-Lys-Leu-Phe-Lys-Lys-Ile-Leu-Lys-Val-Leu-Lys-Lys-Leu-Phe-Lys-Lys-Ile-Leu-Lys-Lys-Leu-NH_2 (BP13-BP16)

This peptide conjugate was prepared following the procedure described in the manuscript using a Fmoc-Rink-ChemMatrix resin (200 mg, 0.66 mmol/g). Acidolytic cleavage of the resulting resin and purification eluting with H_2O/CH_3CN (61:39) yielded **BP13-BP16** in >99% HPLC purity. HPLC (λ = 220 nm): t_R = 8.05 min (Method A); t_R = 7.85 min (Method B). MS (ESI) *m/z*: 1373.1 $[M + 2H]^{2+}$, 1384.5 $[M +H + Na]^{2+}$, 2745.1 $[M + H]^{+}$, 2767.0 $[M + Na]^{+}$. HRMS (ESI) *m/z*: calcd for $C_{140}H_{249}N_{33}O_{22}$ $[M + 2H]^{2+}$ 1372.4684, found 1372.4666.

Lys-Lys-Leu-Phe-LysLys-Ile-Leu-Lys-Lys-Leu-Phe-Lys-Leu-Phe-Lys-Lys-Ile-Leu-Lys-Val-Leu-NH_2 (BP16-BP13)

This peptide conjugate was prepared following the procedure described in the manuscript using a Fmoc-Rink-ChemMatrix resin (200 mg, 0.66 mmol/g). Acidolytic cleavage of the resulting resin and

purification eluting with H_2O/CH_3CN (66:34) yielded **BP16-BP13** in >99% HPLC purity. HPLC (λ = 220 nm): t_R = 8.04 min (Method A); t_R = 7.68 min (Method B). MS (ESI) *m/z*: 1373.1 $[M + 2H]^{2+}$, 1384.1 $[M + H + Na]^{2+}$, 2745.1 $[M + H]^{+}$, 2767.0 $[M + Na]^{+}$. HRMS (ESI) *m/z*: calcd for $C_{140}H_{249}N_{33}O_{22}$ $[M + 2H]^{2+}$ 1372.4684, found 1372.4627.

Phe-Lys-Leu-Phe-Lys-Lys-Ile-Leu-Lys-Val-Leu-Lys-Lys-Leu-Phe-Lys-Lys-Ile-Leu-Lys-Tyr-Leu-NH$_2$ (BP13-BP100)

This peptide conjugate was prepared following the procedure described in the manuscript using a Fmoc-Rink-ChemMatrix resin (200 mg, 0.66 mmol/g)). Acidolytic cleavage of the resulting resin and purification eluting with H_2O/CH_3CN (65:35) yielded **BP13-BP100** in >99% HPLC purity. HPLC (λ = 220 nm): t_R = 8.29 min (Method A); t_R = 8.56 min (Method B). MS (ESI) *m/z*: 927.4 $[M + 3H]^{3+}$, 934.8 $[M + 2H + Na]^{3+}$, 1390.6 $[M + 2H]^{2+}$, 1401.6 $[M + H + Na]^{2+}$, 1412.6 $[M + 2Na]^{2+}$. HRMS (ESI) *m/z*: calcd for $C_{143}H_{248}N_{32}O_{23}$ $[M + 4H]^{4+}$ 695.4800, found 695.4794; calcd for $C_{143}H_{247}N_{32}O_{23}$ $[M + 3H]^{3+}$ 926.9708, found 926.9693; calcd for $C_{143}H_{246}N_{32}O_{23}$ $[M + 2H]^{2+}$ 1389.9526, found 1389.9360.

Lys-Lys-Leu-Phe-Lys-Lys-Ile-Leu-Lys-Tyr-Leu-Phe-Lys-Leu-Phe-Lys-Lys-Ile-Leu-Lys-Val-Leu-NH$_2$ (BP100-BP13)

This peptide conjugate was prepared following the procedure described in the manuscript using a Fmoc-Rink-ChemMatrix resin (200 mg, 0.66 mmol/g). Acidolytic cleavage of the resulting resin and purification eluting with H_2O/CH_3CN (62:38) yielded **BP100-BP13** in >99% HPLC purity. HPLC (λ = 220 nm): t_R = 8.18 min (Method A); t_R = 8.23 min (Method B). MS (ESI) *m/z*: 1390.6 $[M + 2H]^{2+}$, 1401.6 $[M + H + Na]^{2+}$, 1412.6 $[M + 2Na]^{2+}$, 2780.2 $[M + H]^{+}$, 2802.2 $[M + Na]^{+}$. HRMS (ESI) *m/z*: calcd for $C_{143}H_{248}N_{32}O_{23}$ $[M + 4H]^{4+}$ 695.4800, found 695.4811; calcd for $C_{143}H_{247}N_{32}O_{23}$ $[M + 3H]^{3+}$ 926.9708, found 926.9719; calcd for $C_{143}H_{246}N_{32}O_{23}$ $[M + 2H]^{2+}$ 1389.9526, found 1389.9537.

Phe-Lys-Leu-Phe-Lys-Lys-Ile-Leu-Lys-Val-Leu-Lys-Lys-Leu-D-Phe-Lys-Lys-Ile-Leu-Lys-Tyr-Leu-NH$_2$ (BP13-BP143)

This peptide conjugate was prepared following the procedure described in the manuscript using a Fmoc-Rink-ChemMatrix resin (200 mg, 0.66 mmol/g). Acidolytic cleavage of the resulting resin and purification eluting with H_2O/CH_3CN (80:20) yielded **BP13-BP143** in >99% HPLC purity. HPLC (λ = 220 nm): t_R = 8.22 min (Method A); t_R = 8.10 min (Method B). MS (ESI) *m/z*: 1390.7 $[M + 2H]^{2+}$, 1401.7 $[M + H + Na]^{2+}$, 1412.7 $[M + 2Na]^{2+}$, 2780.3 $[M + H]^{+}$, 2802.3 $[M + Na]^{+}$. HRMS (ESI) *m/z*: calcd for $C_{143}H_{248}N_{32}O_{23}$ $[M + 4H]^{4+}$ 695.4800, found 695.4786; calcd for $C_{143}H_{247}N_{32}O_{23}$ $[M + 3H]^{3+}$ 926.9708, found 926.9691; calcd for $C_{143}H_{246}N_{32}O_{23}$ $[M + 2H]^{2+}$ 1389.9526, found 1389.9499.

Lys-Lys-Leu-D-Phe-Lys-Lys-Ile-Leu-Lys-Tyr-Leu-Phe-Lys-Leu-Phe-Lys-Lys-Ile-Leu-Lys-Val-Leu-NH_2 (BP143-BP13)

This peptide conjugate was prepared following the procedure described in the manuscript using a Fmoc-Rink-ChemMatrix resin (200 mg, 0.66 mmol/g). Acidolytic cleavage of the resulting resin and purification eluting with H_2O/CH_3CN (55:45) yielded **BP143-BP13** in >99% HPLC purity. HPLC (λ = 220 nm): t_R = 8.23 min (Method A); t_R = 8.34 min (Method B). MS (ESI) *m*/*z*: 1390.5 $[M + 2H]^{2+}$, 1402.0 $[M + H + Na]^{2+}$, 2780.0 $[M + H]^{+}$, 2801.9 $[M + Na]^{+}$, 2817.8 $[M + K]^{+}$. HRMS (ESI) *m*/*z*: calcd for $C_{143}H_{249}N_{32}O_{23}$ $[M + 5H]^{5+}$ 556.5854, found 556.5850; calcd for $C_{143}H_{248}N_{32}O_{23}$ $[M + 4H]^{4+}$ 695.4800, found 695.4795; calcd for $C_{143}H_{247}N_{32}O_{23}$ $[M + 3H]^{3+}$ 926.9708, found 926.9697; calcd for $C_{143}H_{246}N_{32}O_{23}$ $[M + 2H]^{2+}$ 1389.9526, found 1389.9498.

Phe-Lys-Leu-Phe-Lys-Lys-Ile-Leu-Lys-Val-Leu-Lys-Lys-Val-Val-Phe-Trp-Val-Lys-Phe-Lys-NH_2 (BP13-KSLW)

This peptide conjugate was prepared following the procedure described in the manuscript using a Fmoc-Rink-ChemMatrix resin (200 mg, 0.66 mmol/g). Acidolytic cleavage of the resulting resin and purification eluting with H_2O/CH_3CN (67:33) yielded **BP13-KSLW** in >99% HPLC purity. HPLC (λ = 220 nm): t_R = 7.49 min (Method A); t_R = 7.02 min (Method B). MS (ESI) *m*/*z*: 1334.5 $[M + 2H]^{2+}$, 1345.5 $[M +H + Na]^{2+}$, 1356.0 $[M + 2Na]^{2+}$, 2667.0 $[M + H]^{+}$, 2688.9 $[M + Na]^{+}$. HRMS (ESI) *m*/*z*: calcd for $C_{139}H_{227}N_{31}O_{21}$ $[M + 2H]^{2+}$ 1333.8833, found 1333.8812, calcd for $C_{139}H_{226}N_{31}O_{21}Na$ $[M + H + Na]^{2+}$ 1344.8743, found 1344.8720; calcd for $C_{139}H_{225}N_{31}O_{21}Na_2$ $[M + 2Na]^{2+}$ 1355.8653, found 1355.8654.

Lys-Lys-Val-Val-Phe-Trp-Val-Lys-Phe-Lys-Phe-Lys-Leu-Phe-Lys-Lys-Ile-Leu-Lys-Val-Leu-NH_2 (KSLW-BP13)

This peptide conjugate was prepared following the procedure described in the manuscript using a Fmoc-Rink-ChemMatrix resin (200 mg, 0.66 mmol/g). Acidolytic cleavage of the resulting resin and purification eluting with H_2O/CH_3CN (70:30) yielded **KSLW-BP13** in >99% HPLC purity. HPLC (λ = 220 nm): t_R = 7.03 min (Method A); t_R = 6.26 min (Method B). MS (ESI) *m*/*z*: 2666.8 $[M + H]^{+}$, 2688.7 $[M + Na]^{+}$, 2705.7 $[M + K]^{+}$. HRMS (ESI) *m*/*z*: calcd for $C_{139}H_{227}N_{31}O_{21}$ $[M + 2H]^{2+}$ 1333.8833, found 1333.8801; calcd for $C_{139}H_{226}N_{31}O_{21}Na$ $[M + H +Na]^{2+}$ 1344.8743, found 1344.8711.

Val-Trp-Asn-Gln-Pro-Val-Arg-Gly-Phe-Lys-Val-Tyr-Glu-Lys-Lys-Leu-Phe-Lys-Lys-Ile-Leu-Lys-Lys-Leu-NH_2 (Pep13-BP16)

This peptide conjugate was prepared following the procedure described in the manuscript using a Fmoc-Rink-ChemMatrix resin (200 mg, 0.66 mmol/g). Acidolytic cleavage of the resulting resin and purification eluting with H_2O/CH_3CN (72:28) yielded **Pep13-BP16** in >99% HPLC purity. HPLC (λ = 220 nm): t_R = 6.63 min (Method A); t_R = 5.70 min (Method B). MS (ESI) *m/z*: 1495.5 $[M + 2H]^{2+}$, 2989.9 $[M + H]^{+}$. HRMS (ESI) *m/z*: calcd for $C_{146}H_{240}N_{38}O_{29}$ $[M + 2H]^{2+}$ 1494.9231, found 1494.9216.

Lys-Lys-Leu-Phe-Lys-Lys-Ile-Leu-Lys-Lys-Leu-Val-Trp-Asn-Gln-Pro-Val-Arg-Gly-Phe-Lys-Val-Tyr-Glu-OH(BP16-Pep13)

This peptide conjugate was prepared following the procedure described in the manuscript using a PAC-ChemMatrix resin (200 mg, 0.66 mmol/g). Acidolytic cleavage of the resulting resin and purification eluting with H_2O/CH_3CN (75:25) yielded **BP16-Pep13** in >99% HPLC purity. HPLC (λ = 220 nm): t_R = 6.63 min (Method A); t_R = 5.12 min (Method B). MS (ESI) *m/z*: 1496.5 $[M + 2H]^{2+}$, 2991.8 $[M + H]^{+}$. HRMS (ESI) *m/z*: calcd for $C_{146}H_{239}N_{37}O_{30}$ $[M + 2H]^{2+}$ 1495.4151, found 1495.4161.

Val-Trp-Asn-Gln-Pro-Val-Arg-Gly-Phe-Lys-Val-Tyr-Glu-Lys-Lys-Leu-Phe-Lys-Lys-Ile-Leu-Lys-Tyr-Leu-NH_2 (Pep13-BP100)

This peptide conjugate was prepared following the procedure described in the manuscript using a Fmoc-Rink-ChemMatrix resin (200 mg, 0.66 mmol/g). Acidolytic cleavage of the resulting resin and purification eluting with H_2O/CH_3CN (72:28) yielded **Pep13-BP100** in >99% HPLC purity. HPLC (λ = 220 nm): t_R = 6.85 min (Method A); t_R = 6.14 min (Method B). MS (ESI) *m/z*: 1009.2 $[M + 3H]^{3+}$, 1016.6 $[M + 2H + Na]^{3+}$, 1512.9 $[M + 2H]^{2+}$, 1523.9 $[M + H + Na]^{2+}$. HRMS (ESI) *m/z*: calcd for $C_{149}H_{239}N_{37}O_{30}$ $[M + 4H]^{4+}$ 756.7073, found 756.7061; calcd for $C_{149}H_{238}N_{37}O_{30}$ $[M + 3H]^{3+}$ 1008.6073, found 1008.6047; calcd for $C_{149}H_{237}N_{37}O_{30}$ $[M + 2H]^{2+}$ 1512.4073, found 1512.4007.

Lys-Lys-Leu-Phe-Lys-Lys-Ile-Leu-Lys-Tyr-Leu-Val-Trp-Asn-Gln-Pro-Val-Arg-Gly-Phe-Lys-Val-Tyr-Glu-OH (BP100-Pep13)

This peptide conjugate was prepared following the procedure described in the manuscript using a PAC-ChemMatrix resin (200 mg, 0.66 mmol/g). Acidolytic cleavage of the resulting resin and purification eluting with H_2O/CH_3CN (75:25) yielded **BP100-Pep13** in >99% HPLC purity. HPLC (λ = 220 nm): t_R = 6.44 min (Method A); t_R = 5.53 min (Method B). MS (ESI) *m/z*: 606.0 $[M + 5H]^{5+}$, 757.2 $[M + 4H]^{4+}$, 1009.3 $[M + 3H]^{3+}$, 1513.9 $[M + 2H]^{2+}$, 1524.8 $[M + H + Na]^{2+}$, 1535.9 $[M + 2Na]^{2+}$. HRMS (ESI) *m/z*: calcd for $C_{149}H_{238}N_{36}O_{31}$ $[M + 4H]^{4+}$ 756.9533, found 756.9535; calcd for $C_{149}H_{237}N_{36}O_{31}$ $[M + 3H]^{3+}$ 1008.9353, found 1008.9335; calcd for $C_{149}H_{236}N_{36}O_{31}$ $[M + 2H]^{2+}$ 1512.8993, found 1512.8950.

Val-Trp-Asn-Gln-Pro-Val-Arg-Gly-Phe-Lys-Val-Tyr-Glu-Lys-Lys-Leu-D-Phe-Lys-Lys-Ile-Leu-Lys-Tyr-Leu-NH_2 (Pep13-BP143)

This peptide conjugate was prepared following the procedure described in the manuscript using a Fmoc-Rink-ChemMatrix resin (200 mg, 0.66 mmol/g). Acidolytic cleavage of the resulting resin and purification eluting with H_2O/CH_3CN (70:30) yielded **Pep13-BP143** in >99% HPLC purity. HPLC (λ = 220 nm): t_R = 7.11 min (Method A); t_R = 5.97 min (Method B). MS (ESI) *m/z*: 605.9 $[M + 5H]^{5+}$, 757.1 $[M + 4H]^{4+}$, 1009.3 $[M + 3H]^{3+}$, 1512.9 $[M + 2H]^{2+}$, 1524.4 $[M + H + Na]^{2+}$, 1535.4 $[M + 2Na]^{2+}$. HRMS (ESI) *m/z*: calcd for $C_{149}H_{240}N_{37}O_{30}$ $[M + 5H]^{5+}$ 605.5673, found 605.5688; calcd for $C_{149}H_{239}N_{37}O_{30}$ $[M + 4H]^{4+}$ 756.7073, found 756.7094; calcd for $C_{149}H_{238}N_{37}O_{30}$ $[M + 3H]^{3+}$ 1008.6073, found 1008.6115; calcd for $C_{149}H_{237}N_{37}O_{30}$ $[M + 2H]^{2+}$ 1512.4073, found 1512.4139.

Lys-Lys-Leu-D-Phe-Lys-Lys-Ile-Leu-Lys-Tyr-Leu-Val-Trp-Asn-Gln-Pro-Val-Arg-Gly-Phe-Lys-Val-Tyr-Glu-OH (BP143-Pep13)

This peptide conjugate was prepared following the procedure described in the manuscript using a PAC-ChemMatrix resin (200 mg, 0.66 mmol/g). Acidolytic cleavage of the resulting resin and purification eluting with H_2O/CH_3CN (70:30) yielded **BP143-Pep13** in >99% HPLC purity. HPLC (λ = 220 nm): t_R = 6.92 min (Method A); t_R = 5.62 min (Method B). MS (ESI) *m/z*: 606.1 $[M + 5H]^{5+}$, 757.3 $[M + 4H]^{4+}$, 1009.2 $[M + 3H]^{3+}$, 1513.3 $[M + 2H]^{2+}$. HRMS (ESI) *m/z*: calcd for $C_{149}H_{239}N_{36}O_{31}$ $[M + 5H]^{5+}$ 605.7641, found 605.7628; calcd for $C_{149}H_{238}N_{36}O_{31}$ $[M + 4H]^{4+}$ 756.9533, found 756.9520; calcd for $C_{149}H_{237}N_{36}O_{31}$ $[M + 3H]^{3+}$ 1008.9353, found 1008.9342; calcd for $C_{149}H_{236}N_{36}O_{31}$ $[M + 2H]^{2+}$ 1512.8993, found 1512.8990.

Val-Trp-Asn-Gln-Pro-Val-Arg-Gly-Phe-Lys-Val-Tyr-Glu-Lys-Lys-Val-Val-Phe-Trp-Val-Lys-Phe-Lys-NH_2 (Pep13-KSLW)

This peptide conjugate was prepared following the procedure described in the manuscript using a Fmoc-Rink-ChemMatrix resin (200 mg, 0.66 mmol/g). Acidolytic cleavage of the resulting resin and purification eluting with H_2O/CH_3CN (84:16) yielded **Pep13-KSLW** in >99% HPLC purity. HPLC (λ = 220 nm): t_R = 6.75 min (Method A); t_R = 5.15 min (Method B). MS (ESI) *m/z*: 1456.5 $[M + 2H]^{2+}$, 2910.8 $[M + H]^{+}$. HRMS (ESI) *m/z*: calcd for $C_{145}H_{221}N_{36}O_{28}$ $[M + 5H]^{5+}$ 582.9390, found 582.9387; calcd for $C_{145}H_{220}N_{36}O_{28}$ $[M + 4H]^{4+}$ 728.4219, found 728.4214; calcd for $C_{145}H_{219}N_{36}O_{28}$ $[M + 3H]^{3+}$ 970.8934, found 970.8935; calcd for $C_{145}H_{218}N_{36}O_{28}$ $[M + 2H]^{2+}$ 1455.8365, found 1455.8384.

Lys-Lys-Val-Val-Phe-Trp-Val-Lys-Phe-Lys-Val-Trp-Asn-Gln-Pro-Val-Arg-Gly-Phe-Lys-Val-Tyr-Glu-OH (KSLW-Pep13)

This peptide conjugate was prepared following the procedure described in the manuscript using a PAC-ChemMatrix resin (200 mg, 0.66 mmol/g). Acidolytic cleavage of the resulting resin and purification eluting with H_2O/CH_3CN (80:20) yielded **KSLW-Pep13** in >99% HPLC purity. HPLC (λ = 220 nm): t_R = 6.66 min (Method A); t_R = 5.10 min (Method B). MS (ESI) *m*/*z*: 971.6 $[M + 3H]^{3+}$, 978.9 $[M + 2H + Na]^{3+}$, 1456.8 $[M + 2H]^{2+}$, 1467.8 $[M + H + Na]^{2+}$. HRMS (ESI) *m*/*z*: calcd for $C_{145}H_{219}N_{35}O_{29}$ $[M + 4H]^{4+}$ 728.6679, found 728.6690; calcd for $C_{145}H_{218}N_{35}O_{29}$ $[M + 3H]^{3+}$ 971.2214, found 971.2214; calcd for $C_{145}H_{217}N_{35}O_{29}$ $[M + 2H]^{2+}$ 1456.3285, found 1456.3288.

Ac-Tyr-Gly-Ile-His-Thr-His-Lys-Lys-Leu-D-Phe-Lys-Lys-Ile-Leu-Lys-Lys(COC_3H_7)-Leu-NH_2 (PIP1-BP475)

This peptide conjugate was prepared following the procedure described in the manuscript using a Fmoc-Rink-ChemMatrix resin (200 mg, 0.66 mmol/g). Acidolytic cleavage of the resulting resin and purification eluting with H_2O/CH_3CN (80:20) afforded **PIP1-BP475** in >99% HPLC purity. HPLC (λ = 220 nm): t_R = 5.55 min (Method B). MS (ESI) *m*/*z*: 736.5 $[M + 3H]^{3+}$, 1104.3 $[M + 2H]^{2+}$, 2245.4 $[M + K]^{+}$. HRMS (ESI) *m*/*z*: calcd for $C_{108}H_{182}N_{28}O_{21}$ $[M + 2H]^{2+}$ 1103.7012, found 1103.6984; calcd for $C_{108}H_{181}N_{28}O_{21}$ $[M + H]^{+}$ 2206.3951, found 2206.3902.

Ac-Lys-Lys-Leu-D-Phe-Lys-Lys-Ile-Leu-Lys-Lys(COC_3H_7)-Leu-Tyr-Gly-Ile-His-Thr-His-NH_2 (BP475-PIP1)

This peptide conjugate was prepared following the procedure described in the manuscript using a Fmoc-Rink-ChemMatrix resin (200 mg, 0.66 mmol/g). Acidolytic cleavage of the resulting resin and purification eluting with H_2O/CH_3CN (75:25) afforded **BP475-PIP1** in >99% HPLC purity. HPLC (λ = 220 nm): t_R = 6.28 min (Method B). MS (ESI) *m*/*z*: 736.5 $[M + 3H]^{3+}$, 1104.3 $[M + 2H]^{2+}$, 2229.5 $[M + Na]^{+}$. HRMS (ESI) *m*/*z*: calcd for $C_{108}H_{182}N_{28}O_{21}$ $[M + 2H]^{2+}$ 1103.7012, found 1103.7012; calcd for $C_{108}H_{181}N_{28}O_{21}$ $[M + H]^{+}$ 2206.3951, found 2206.3981.

Tyr-Gly-Ile-His-Thr-His-Lys-Lys-Val-Val-Phe-Trp-Val-Lys-Phe-Lys-NH_2 (PIP1-KSLW)

This peptide conjugate was prepared following the procedure described in the manuscript using a Fmoc-Rink-ChemMatrix resin (200 mg, 0.66 mmol/g). Acidolytic cleavage of the resulting resin and purification eluting with H_2O/CH_3CN (77:23) yielded **PIP1-KSLW** in >99% HPLC purity. HPLC (λ = 220 nm): t_R = 6.15 min (Method A); t_R = 4.81 min (Method B). MS (ESI) *m*/*z*: 1009.1 $[M + 2H]^{2+}$, 2017.2 $[M + H]^{+}$, 2039.2 $[M + Na]^{+}$. HRMS (ESI) *m*/*z*: calcd for $C_{101}H_{152}N_{26}O_{18}$ $[M + 2H]^{2+}$ 1008.5883, found 1008.5881; calcd for $C_{101}H_{151}N_{26}O_{18}$ $[M + H]^{+}$ 2016.1694, found 2016.1636.

Lys-Lys-Val-Val-Phe-Trp-Val-Lys-Phe-Lys-Tyr-Gly-Ile-His-Thr-His-NH_2 (KSLW-PIP1)

This peptide conjugate was prepared following the procedure described in the manuscript using a Fmoc-Rink-ChemMatrix resin (200 mg, 0.66 mmol/g). Acidolytic cleavage of the resulting resin and

purification eluting with H_2O/CH_3CN (75:25) yielded **KSLW-PIP1** in >99% HPLC purity. HPLC (λ = 220 nm): t_R = 6.17 min (Method A); t_R = 4.83 min (Method B). MS (ESI) *m*/*z*: 1009.1 $[M + 2H]^{2+}$, 2017.2 $[M + H]^{+}$, 2039.1 $[M + Na]^{+}$. HRMS (ESI) *m*/*z*: calcd for $C_{101}H_{152}N_{26}O_{18}$ $[M + 2H]^{2+}$ 1008.5883, found 1008.5871; calcd for $C_{101}H_{151}N_{26}O_{18}$ $[M + H]^{+}$ 2016.1694, found 2016.1636.

Table D. Sequences, retention times and purities on HPLC, and mass spectrometry data of peptide conjugates

Peptide	Sequence[a]	t_R (min)[b]	Purity (%)[c]	HRMS (ESI)		
					Calcd.	Found
**flg15-BP16**	RINSAKDDAAGLQIA-KKLFKKILKKL-NH_2	5.89	>99	$C_{133}H_{239}N_{39}O_{33}$ [M + 2H]$^{2+}$	1455.9120	1455.9087
**BP16-flg15**	KKLFKKILKKL-RINSAKDDAAGLQIA-OH	4.59	>99	$C_{133}H_{238}N_{38}O_{34}$ [M + 2H]$^{2+}$	1456.4040	1456.4023
**flg15-BP100**	RINSAKDDAAGLQIA-KKLFKKILKYL-NH_2	6.29	>99	$C_{136}H_{236}N_{38}O_{34}$ [M + 2H]$^{2+}$	1472.8948	1472.8977
**BP100-flg15**	KKLFKKILKYL-RINSAKDDAAGLQIA-OH	4.96	>99	$C_{136}H_{235}N_{37}O_{35}$ [M + 2H]$^{2+}$	1473.3868	1473.3903
**flg15-BP387**	Ac-RINSAKDDAAGLQIA-KKLFKKIK(COC_3H_7)KYL-NH_2	6.35	96	$C_{142}H_{245}N_{39}O_{36}$ [M + 2H]$^{2+}$	1536.4264	1536.4212
**BP387-flg15**	Ac-KKLFKKIK(COC_3H_7)KYL-RINSAKDDAAGLQIA-OH	5.84	96	$C_{142}H_{244}N_{38}O_{37}$ [M + 2H]$^{2+}$	1536.9184	1536.9152
**flg15-BP475**	Ac-RINSAKDDAAGLQIA-KKLfKKILKK(COC_3H_7)L-NH_2	6.79	97	$C_{139}H_{247}N_{39}O_{35}$ [M + 2H]$^{2+}$	1511.4368	1511.4329
**BP475-flg15**	Ac-KKLfKKILKK(COC_3H_7)L-RINSAKDDAAGLQIA-OH	6.03	>99	$C_{139}H_{246}N_{38}O_{36}$ [M + 2H]$^{2+}$	1511.9288	1511.9271
**flg15-KSLW**	RINSAKDDAAGLQIA-KKVVFWVKFK-NH_2	5.40	>99	$C_{132}H_{217}N_{37}O_{32}$ [M + 2H]$^{2+}$	1416.8254	1416.8202
**KSLW-flg15**	KKVVFWVKFK-RINSAKDDAAGLQIA-OH	4.75	>99	$C_{132}H_{216}N_{36}O_{33}$ [M + 2H]$^{2+}$	1417.3174	1417.3209
**BP13-BP16**	FKLFKKILKVL-KKLFKKILKKL-NH_2	7.85	>99	$C_{140}H_{249}N_{33}O_{22}$ [M + 2H]$^{2+}$	1372.4684	1372.4666
**BP16-BP13**	KKLFKKILKKL-FKLFKKILKVL-NH_2	7.68	>99	$C_{140}H_{249}N_{33}O_{22}$ [M + 2H]$^{2+}$	1372.4684	1372.4627
**BP13-BP100**	FKLFKKILKVL-KKLFKKILKYL-NH_2	8.56	>99	$C_{143}H_{246}N_{32}O_{23}$ [M + 2H]$^{2+}$	1389.9526	1389.9360
**BP100-BP13**	KKLFKKILKYL-FKLFKKILKVL-NH_2	8.23	>99	$C_{143}H_{246}N_{32}O_{23}$ [M + 2H]$^{2+}$	1389.9526	1389.9537
**BP13-BP143**	FKLFKKILKVL-KKLfKKILKYL-NH_2	8.10	>99	$C_{143}H_{246}N_{32}O_{23}$ [M + 2H]$^{2+}$	1389.9526	1389.9499
**BP143-BP13**	KKLfKKILKYL-FKLFKKILKVL-NH_2	8.34	>99	$C_{143}H_{246}N_{32}O_{23}$ [M + 2H]$^{2+}$	1389.9526	1389.9498
**BP13-KSLW**	FKLFKKILKVL-KKVVFWVKFK-NH_2	7.02	>99	$C_{139}H_{227}N_{31}O_{21}$ [M + 2H]$^{2+}$	1333.8833	1333.8812
**KSLW-BP13**	KKVVFWVKFK-FKLFKKILKVL-NH_2	6.26	>99	$C_{139}H_{227}N_{31}O_{21}$ [M + 2H]$^{2+}$	1333.8833	1333.8801
**Pep13-BP16**	VWNQPVRGFKVYE-KKLFKKILKKL-NH_2	5.70	>99	$C_{146}H_{240}N_{38}O_{29}$ [M + 2H]$^{2+}$	1494.9231	1494.9216
**BP16-Pep13**	KKLFKKILKKL-VWNQPVRGFKVYE-OH	5.12	>99	$C_{146}H_{239}N_{37}O_{30}$ [M + 2H]$^{2+}$	1495.4151	1495.4161
**Pep13-BP100**	VWNQPVRGFKVYE-KKLFKKILKYL-NH_2	6.14	>99	$C_{149}H_{237}N_{37}O_{30}$ [M + 2H]$^{2+}$	1512.4073	1512.4007
**BP100-Pep13**	KKLFKKILKYL-VWNQPVRGFKVYE-OH	5.53	>99	$C_{149}H_{236}N_{36}O_{31}$ [M + 2H]$^{2+}$	1512.8993	1512.8950
**Pep13-BP143**	VWNQPVRGFKVYE-KKLfKKILKYL-NH_2	5.97	>99	$C_{149}H_{237}N_{37}O_{30}$ [M + 2H]$^{2+}$	1512.4073	1512.4139
**BP143-Pep13**	KKLfKKILKYL-VWNQPVRGFKVYE-OH	5.62	>99	$C_{149}H_{236}N_{36}O_{31}$ [M + 2H]$^{2+}$	1512.8993	1512.8990
**Pep13-KSLW**	VWNQPVRGFKVYE-KKVVFWVKFK-NH_2	5.15	>99	$C_{145}H_{218}N_{36}O_{28}$ [M + 2H]$^{2+}$	1455.8365	1455.8384
**KSLW-Pep13**	KKVVFWVKFK-VWNQPVRGFKVYE-OH	5.10	>99	$C_{145}H_{217}N_{35}O_{29}$ [M + 2H]$^{2+}$	1456.3285	1456.3288
**PIP1-BP475**	Ac-YGIHTH-KKLfKKILKK(COC_3H_7)L-NH_2	5.55	>99	$C_{108}H_{181}N_{28}O_{21}$ [M + H]$^{+}$	2206.3951	2206.3902
**BP475-PIP1**	Ac-KKLfKKILKK(COC_3H_7)L-YGIHTH-NH_2	6.28	>99	$C_{108}H_{181}N_{28}O_{21}$ [M + H]$^{+}$	2206.3951	2206.3981
**PIP1-KSLW**	YGIHTH-KKVVFWVKFK-NH_2	4.81	>99	$C_{101}H_{152}N_{26}O_{18}$ [M + 2H]$^{2+}$	1008.5883	1008.5881
**KSLW-PIP1**	KKVVFWVKFK-YGIHTH-NH_2	4.83	>99	$C_{101}H_{152}N_{26}O_{18}$ [M + 2H]$^{2+}$	1008.5883	1008.5871

[a] COC_3H_7, butanoyl; lower case letters correspond to D-amino acids

[b] HPLC retention time (Method B)

[c] Percentage determined by HPLC at 220 nm after purification

3. Characterization of peptide conjugates: HPLC, ESI-MS and HRMS

Arg-Ile-Asn-Ser-Ala-Lys-Asp-Asp-Ala-Ala-Gly-Leu-Gln-Ile-Ala-Lys-Lys-Leu-Phe-Lys-Lys-Ile-Leu-Lys-Lys-Leu- NH_2 (flg15-BP16)

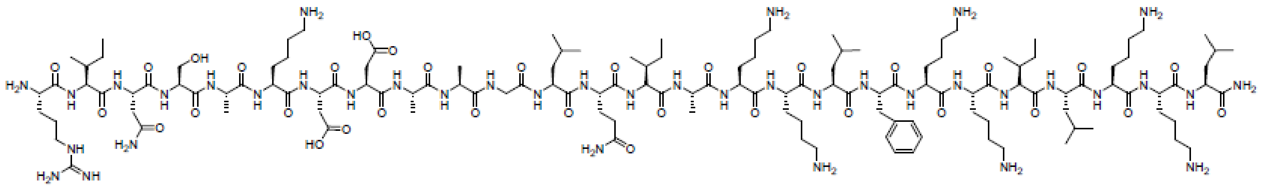

HPLC (λ=220 nm)

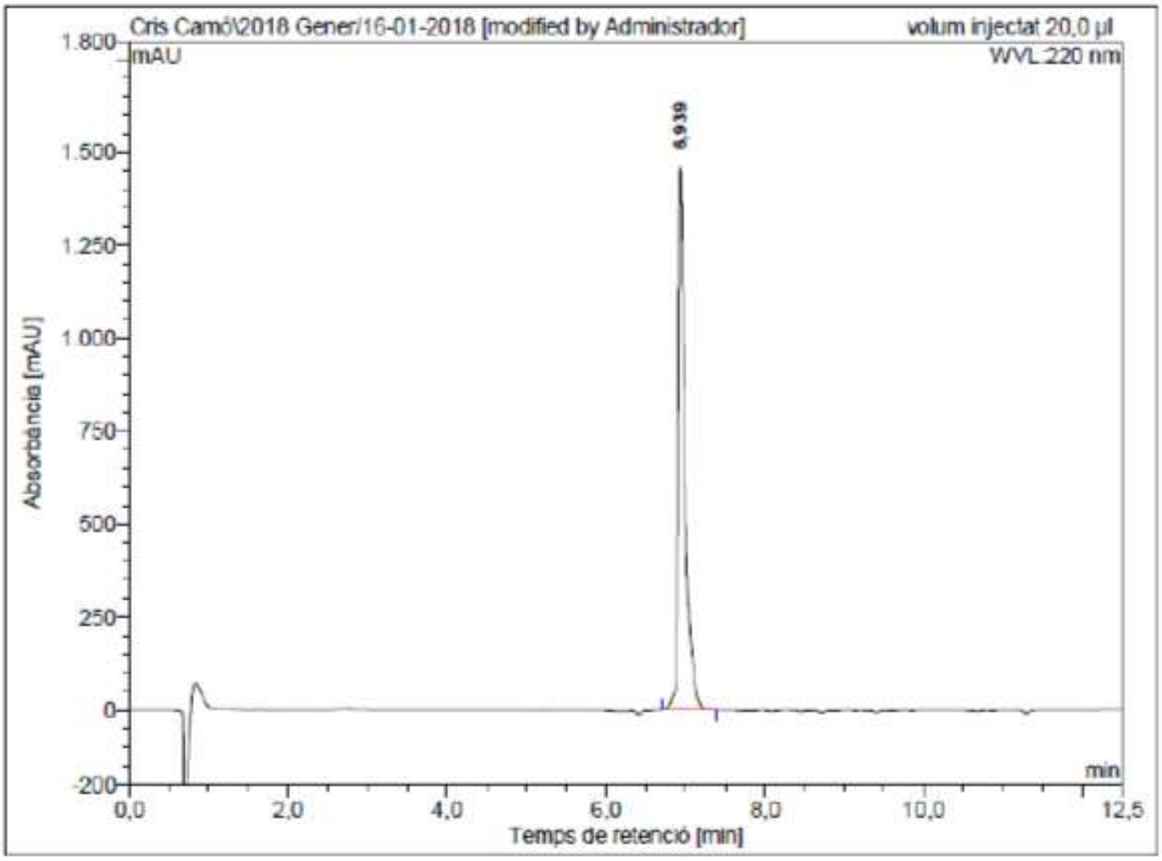

No.	Temps retenció min	alçada mAU	Area mAU*min	Area relativa %
1	6,94	1457,802	142,886	100,00
Total:		1457,802	142,886	100,00

ESI-MS (*m/z*)

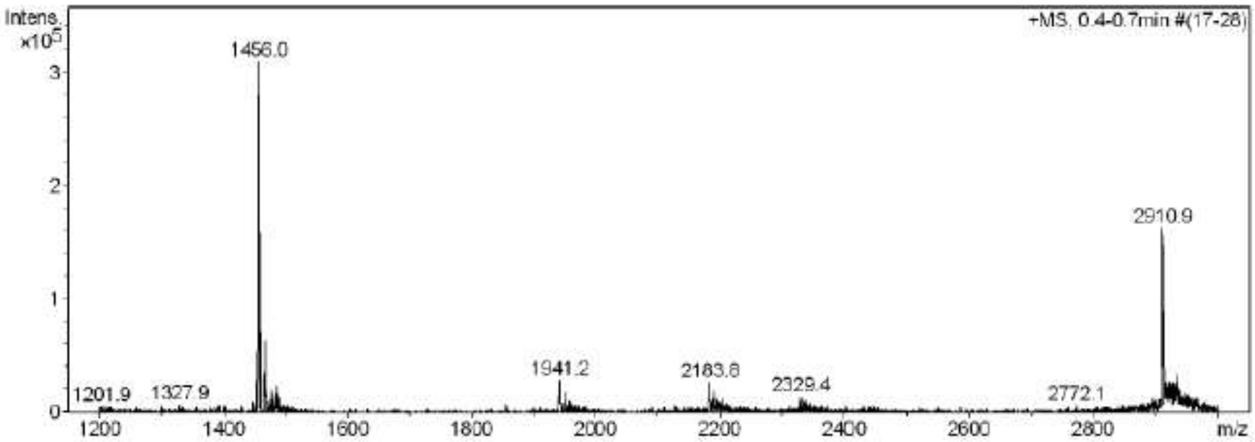

HRMS (*m/z*)

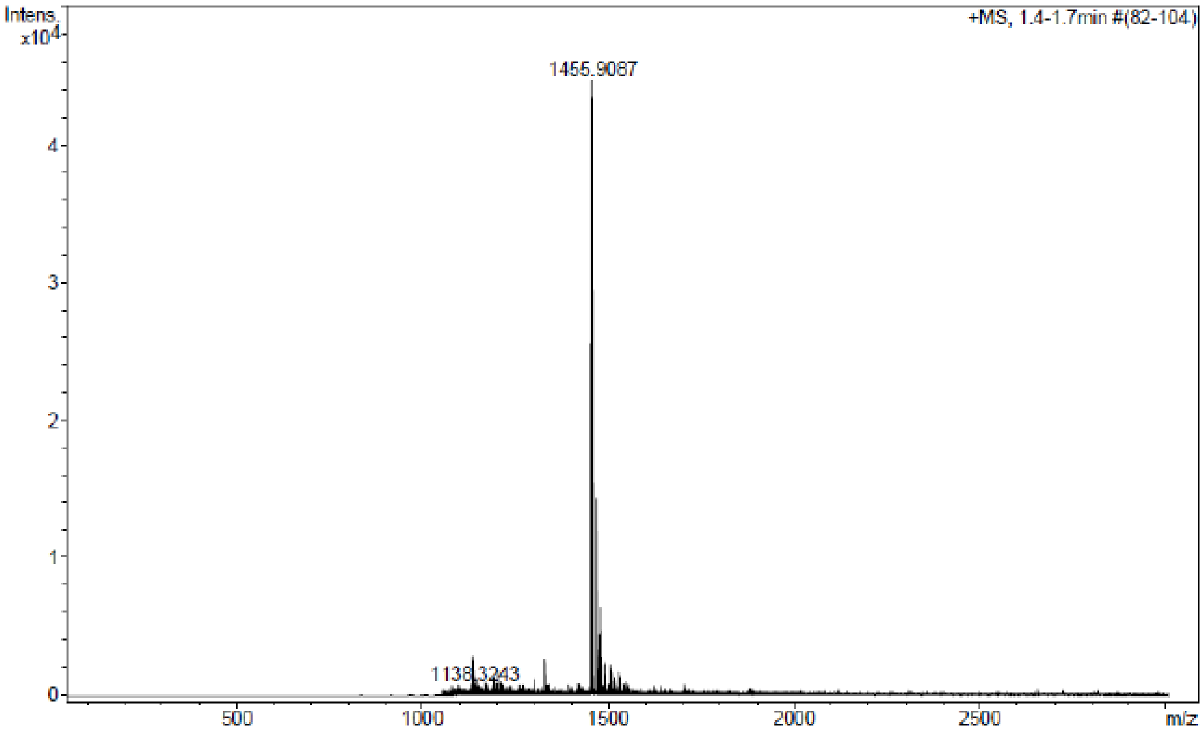

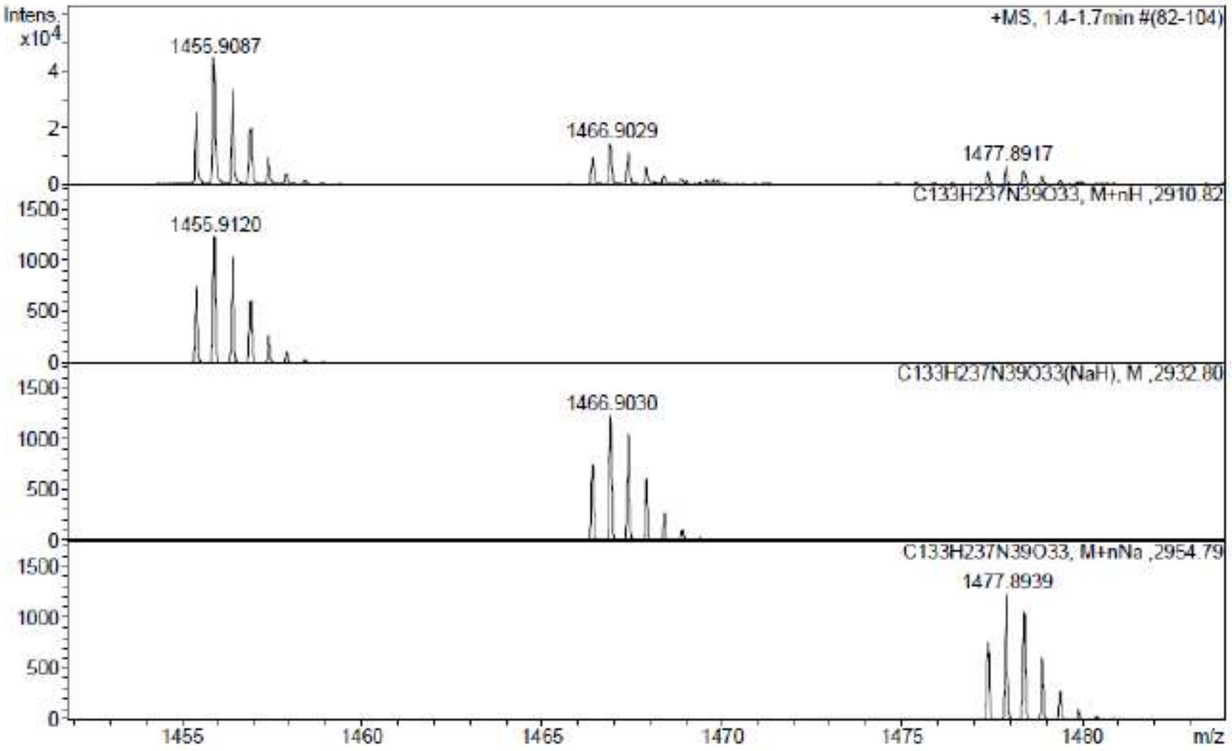

Intens.
x10^4
+MS, 1.4-1.7min #(82-104)
1455.9087
1466.9029
1477.8917
C133H237N39O33, M+nH ,2910.82
1455.9120
C133H237N39O33(NaH), M ,2932.80
1466.9030
C133H237N39O33, M+nNa ,2954.79
1477.8939
m/z

Lys-Lys-Leu-Phe-Lys-Lys-Ile-Leu-Lys-Lys-Leu-Arg-Ile-Asn-Ser-Ala-Lys-Asp-Asp-Ala-Ala-Gly-Leu-Gln-Ile-Ala-OH (BP16-flg15)

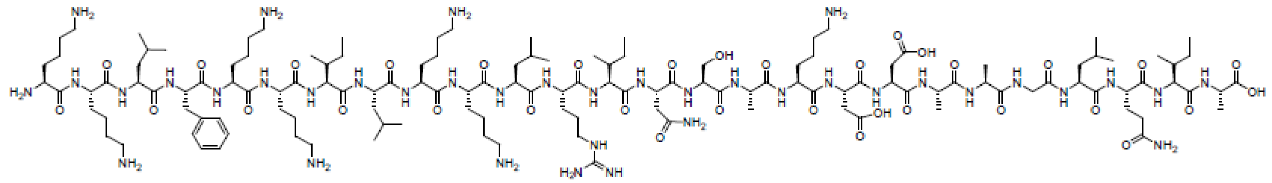

HPLC (λ=220 nm)

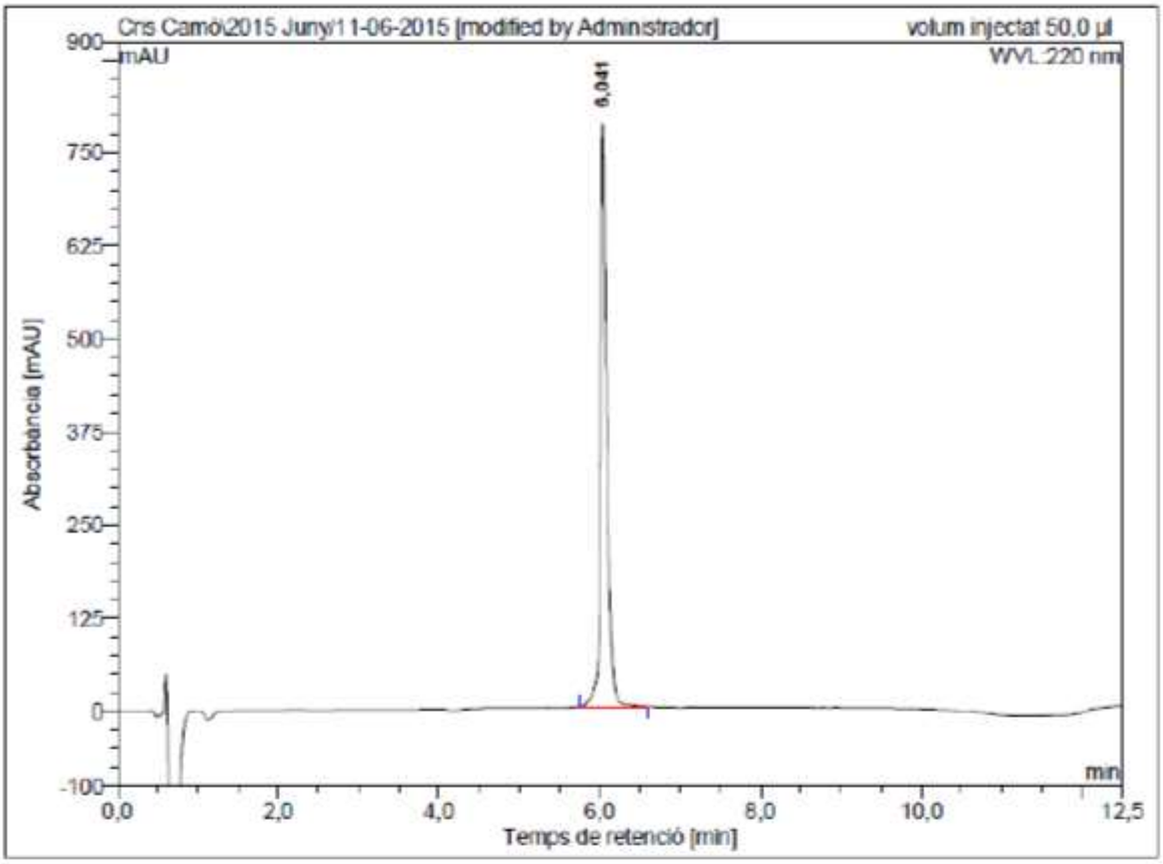

No.	Temps retenció min	alçada mAU	Area mAU*min	Area relativa %
1	6,04	782,370	71,301	100,00
Total:		782,370	71,301	100,00

ESI-MS (*m/z*)

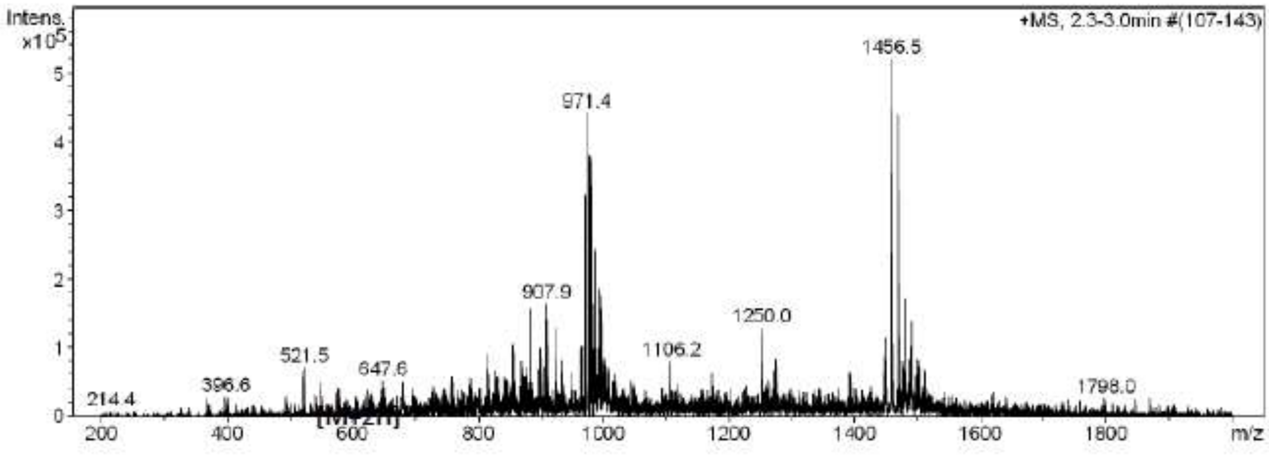

HRMS (*m/z*)

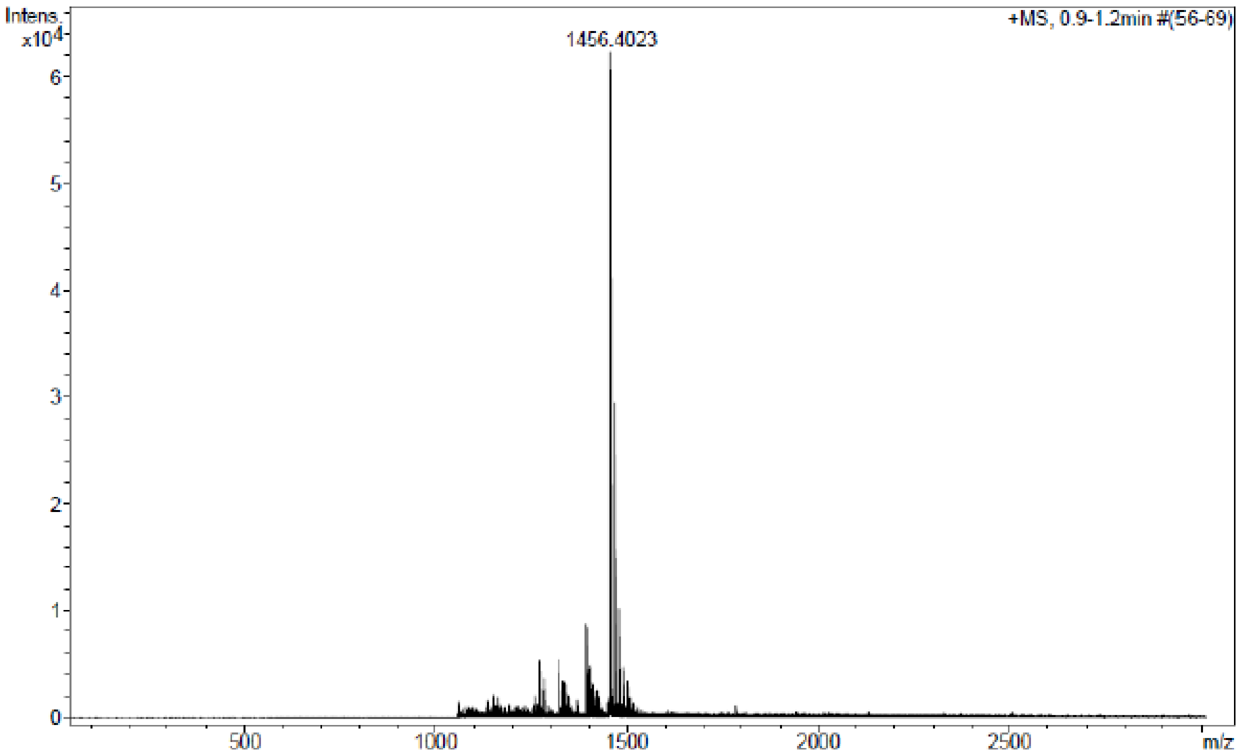

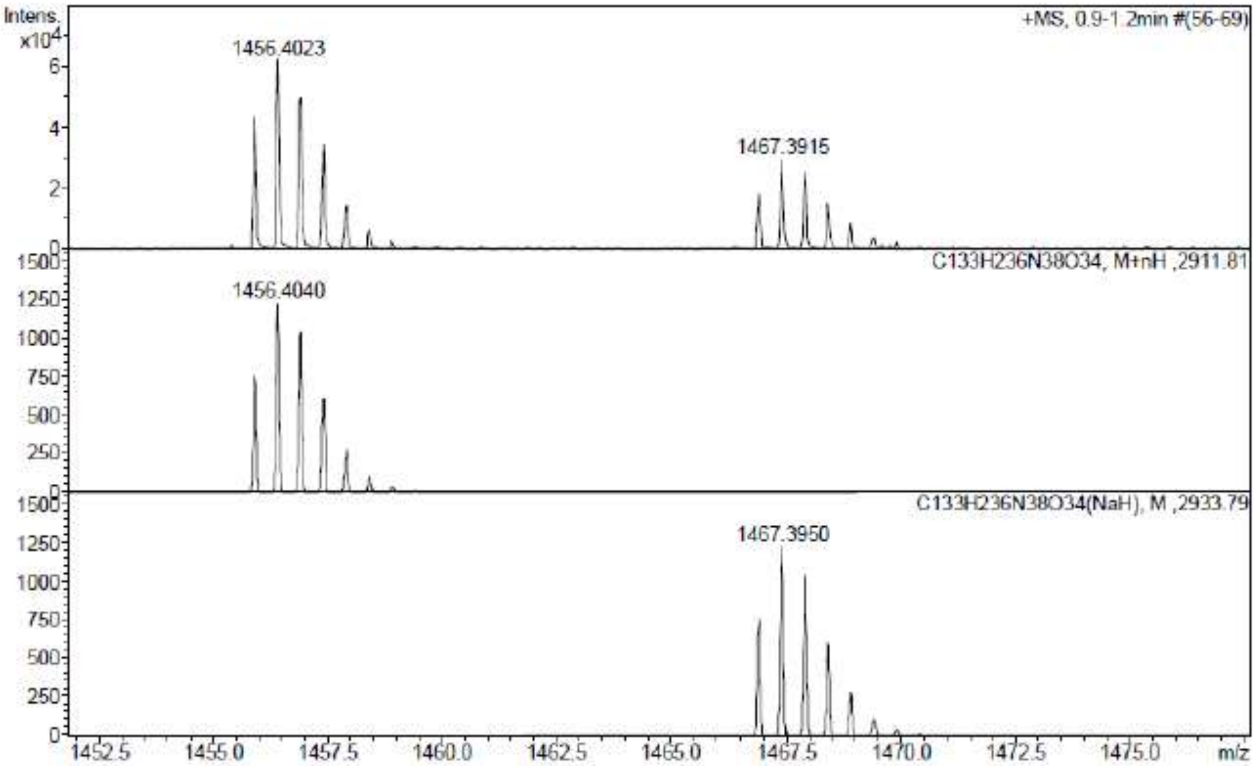
Intens.
x10^4
+MS, 0.9-1.2min #(56-69)
1456.4023
1467.3915
C133H236N38O34, M+nH ,2911.81
1456.4040
C133H236N38O34(NaH), M ,2933.79
1467.3950
m/z

Arg-Ile-Asn-Ser-Ala-Lys-Asp-Asp-Ala-Ala-Gly-Leu-Gln-Ile-Ala-Lys-Lys-Leu-Phe-Lys-Lys-Ile-Leu-Lys-Tyr-Leu-NH_2 (flg15-BP100)

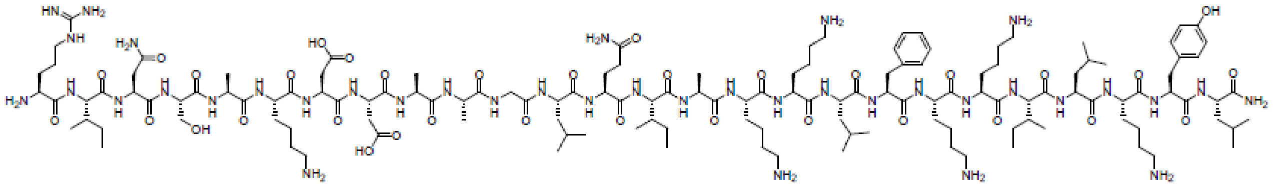

HPLC (λ=220 nm)

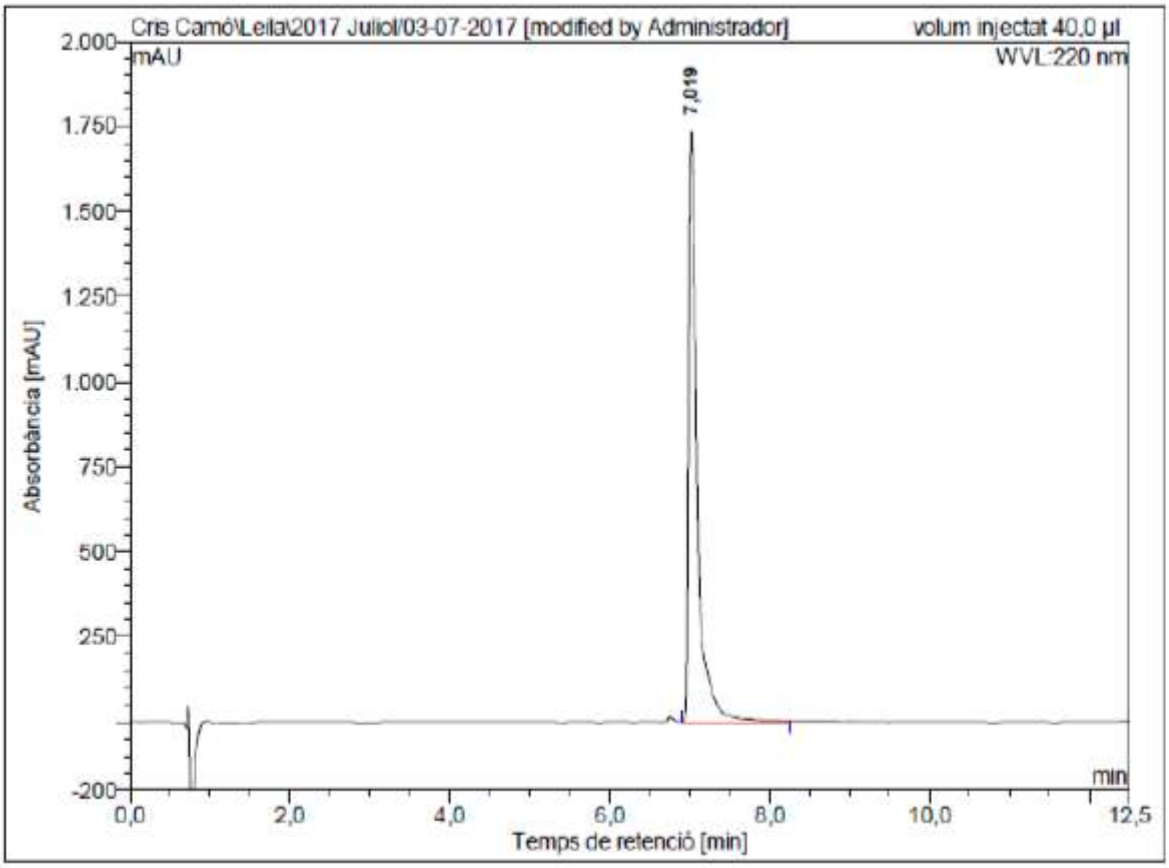

No.	Temps retenció min	alçada mAU	Area mAU*min	Area relativa %
1	7,02	1735,316	214,596	100,00
Total:		1735,316	214,596	100,00

ESI-MS (*m/z*)

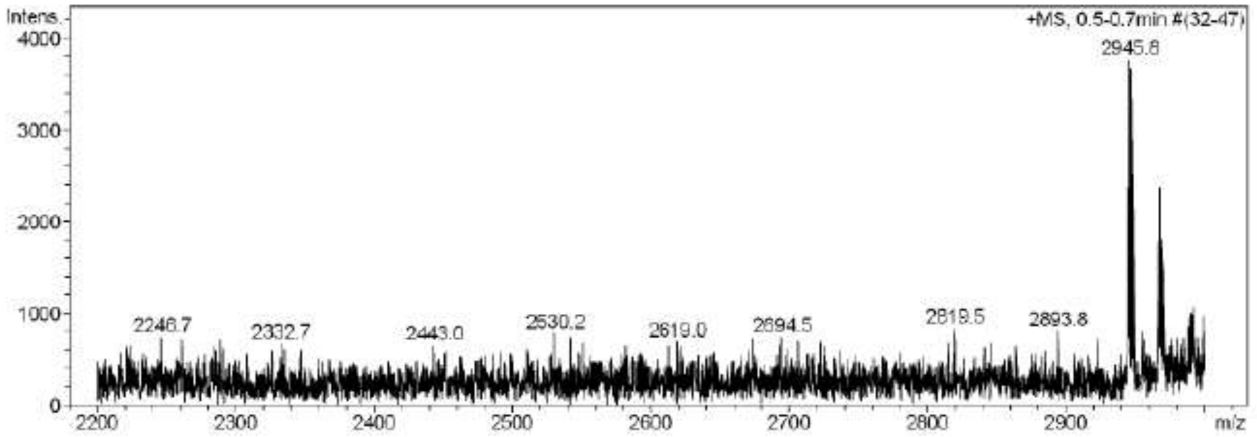
Intens.
+MS, 0.5-0.7min #(32-47)
2945.8
2246.7
2332.7
2443.0
2530.2
2619.0
2694.5
2819.5
2893.8
m/z

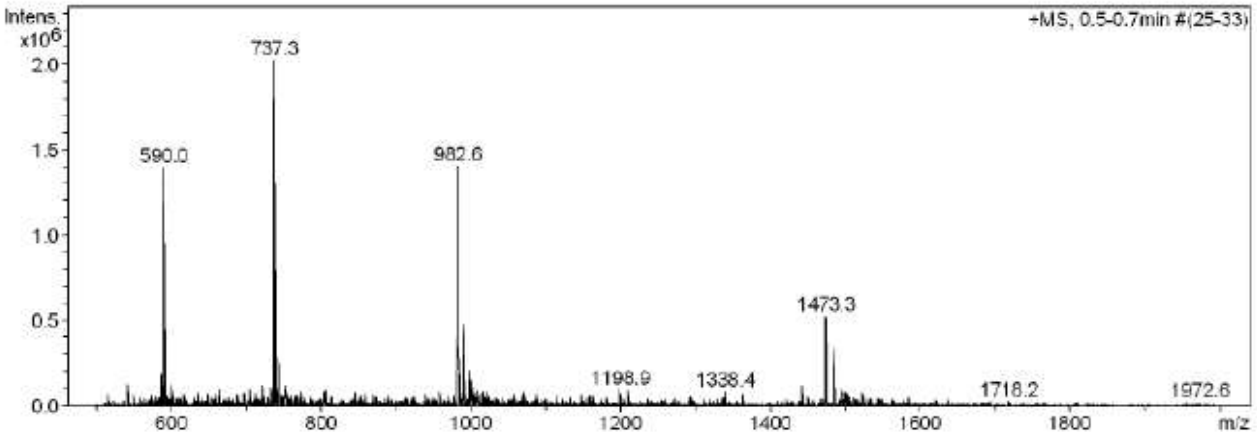
Intens.
x10^6
+MS, 0.5-0.7min #(25-33)
737.3
590.0
982.6
1473.3
1198.9
1338.4
1718.2
1972.6
m/z

HRMS (*m/z*)

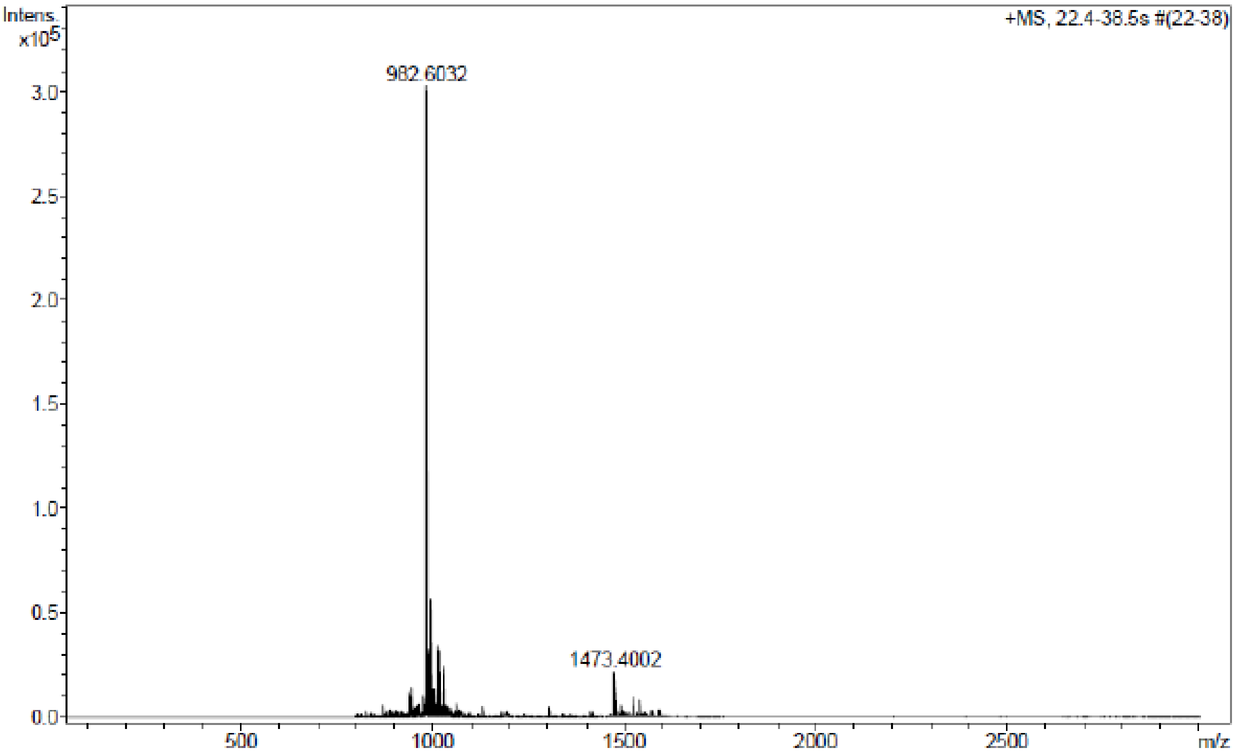

Intens.
x10^5
+MS, 22.4-38.5s #(22-38)
982.6032
1473.4002
3.0
2.5
2.0
1.5
1.0
0.5
0.0
500
1000
1500
2000
2500
m/z

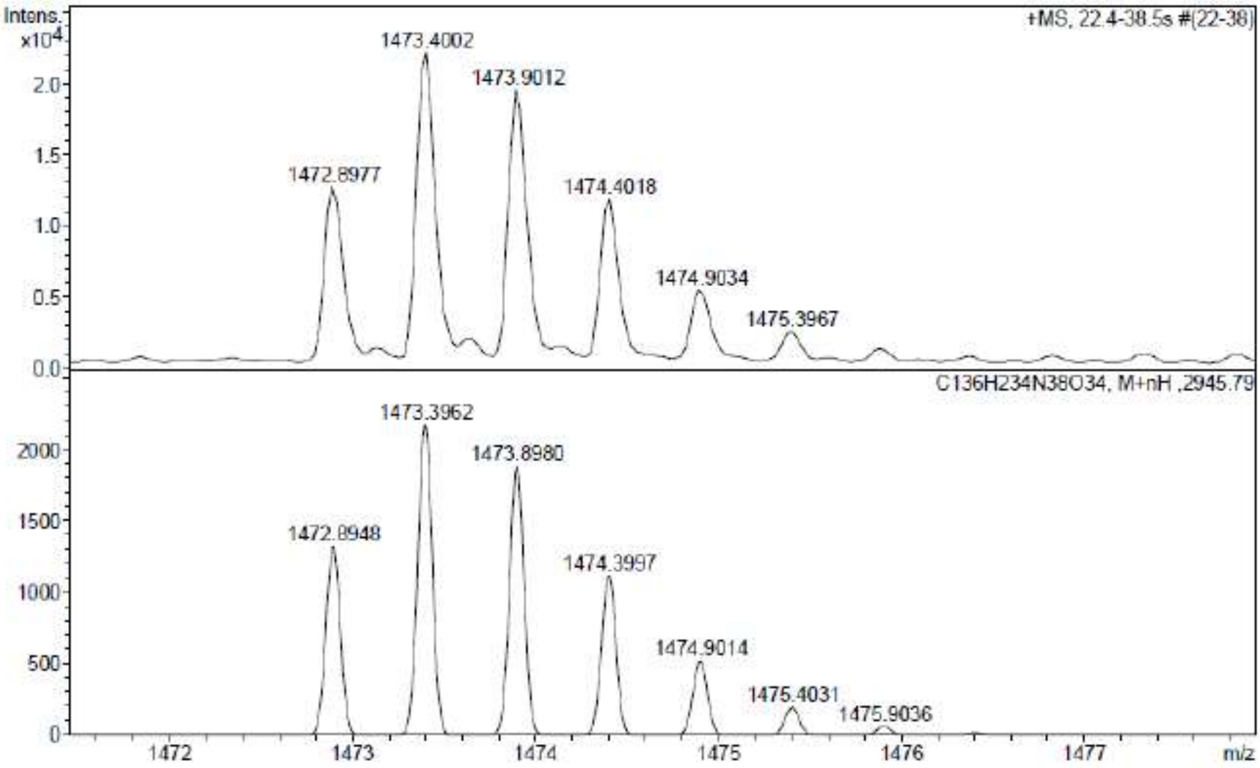

Intens.
x10^4
+MS, 22.4-38.5s #(22-38)
1473.4002
1473.9012
1472.8977
1474.4018
1474.9034
1475.3967
2.0
1.5
1.0
0.5
0.0
C136H234N38O34, M+nH ,2945.79
1473.3962
1473.8980
1472.8948
1474.3997
1474.9014
1475.4031
1475.9036
2000
1500
1000
500
0
1472
1473
1474
1475
1476
1477
m/z

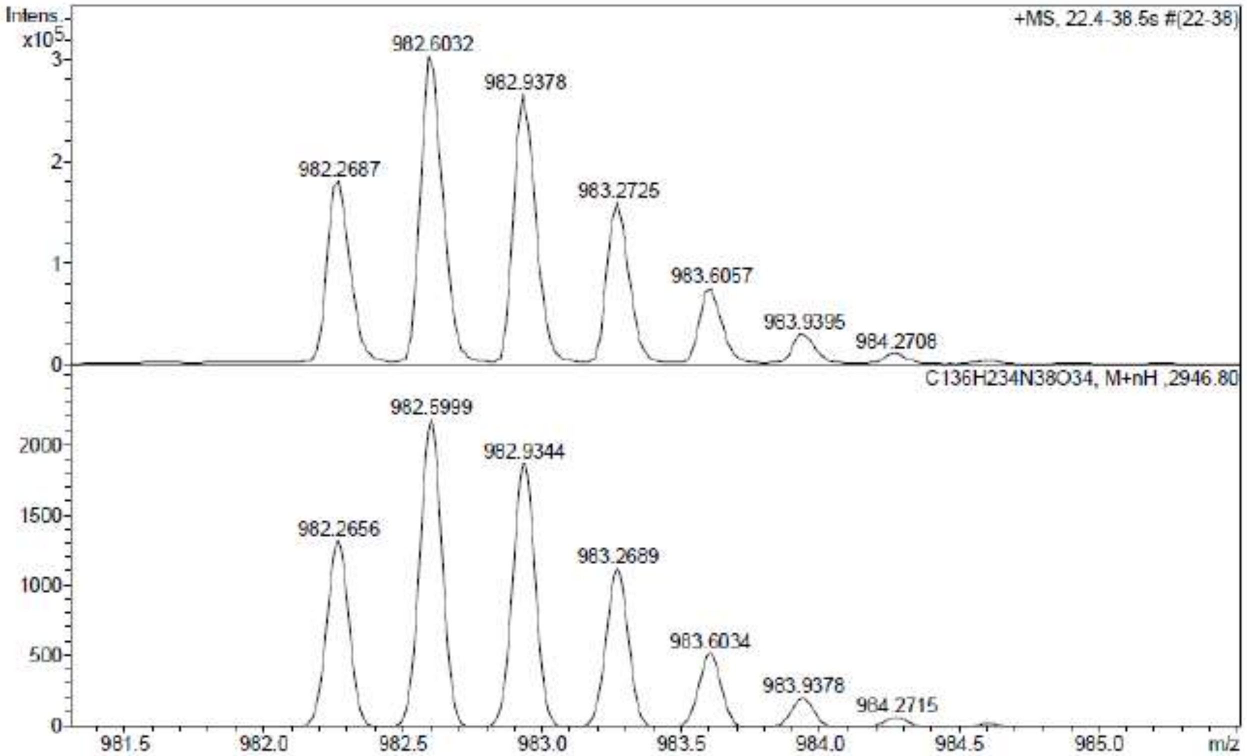
Intens.
x10^5
+MS, 22.4-38.5s #(22-38)
982.6032
982.9378
982.2687
983.2725
983.6057
983.9395
984.2708
C136H234N38O34, M+nH ,2946.80
982.5999
982.9344
982.2656
983.2689
983.6034
983.9378
984.2715
981.5
982.0
982.5
983.0
983.5
984.0
984.5
985.0
m/z

Lys-Lys-Leu-Phe-Lys-Lys-Ile-Leu-Lys-Tyr-Leu-Arg-Ile-Asn-Ser-Ala-Lys-Asp-Asp-Ala-Ala-Gly-Leu-Gln-Ile-Ala- OH (BP100-flg15)

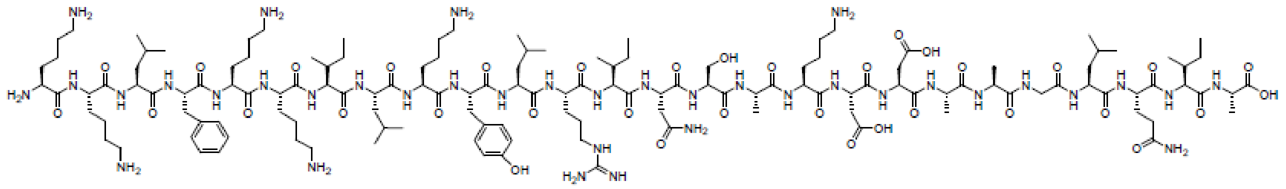

HPLC (λ=220 nm)

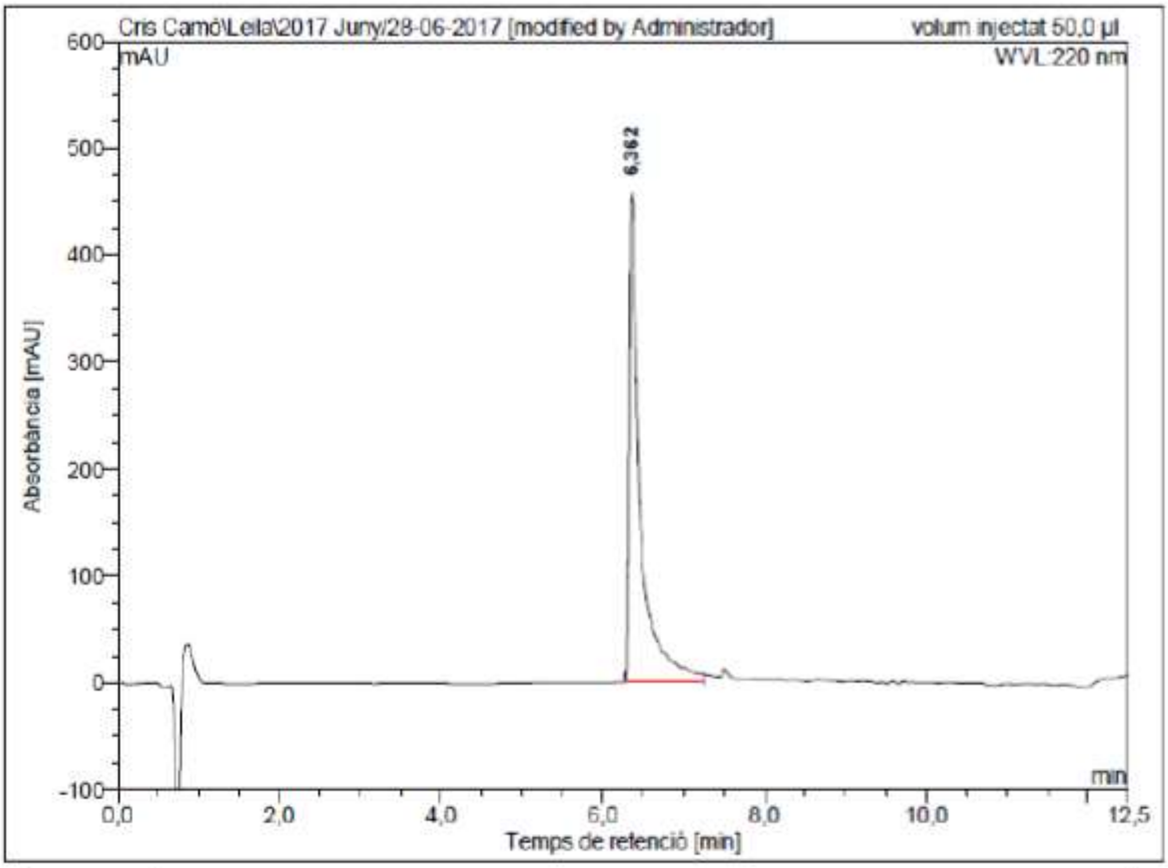

No.	Temps retenció min	alçada mAU	Area mAU*min	Area relativa %
1	6,36	457,267	73,103	100,00
Total:		457,267	73,103	100,00

ESI-MS (*m/z*)

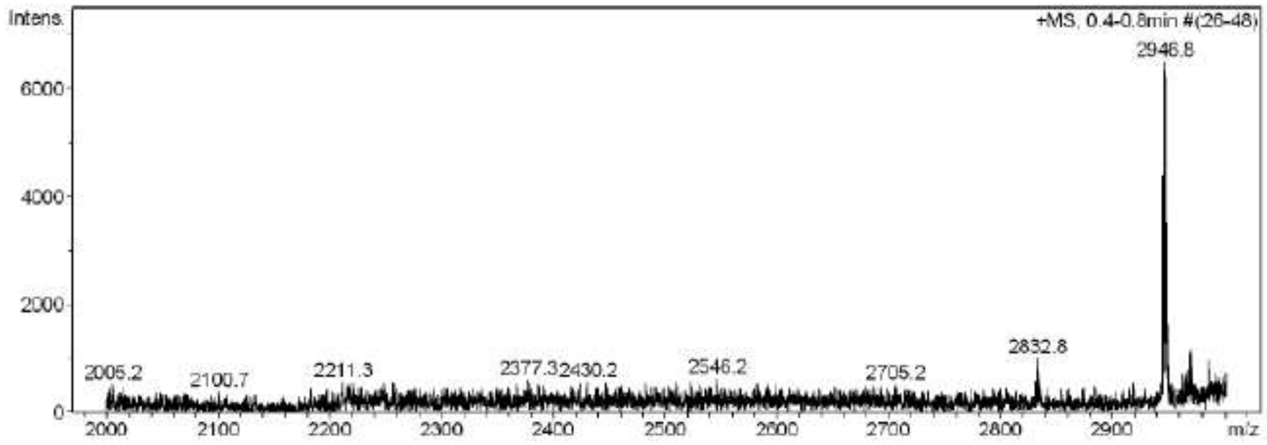
Intens.
+MS, 0.4-0.8min #(26-48)
2946.8
2832.8
2005.2
2100.7
2211.3
2377.3
2430.2
2546.2
2705.2
m/z

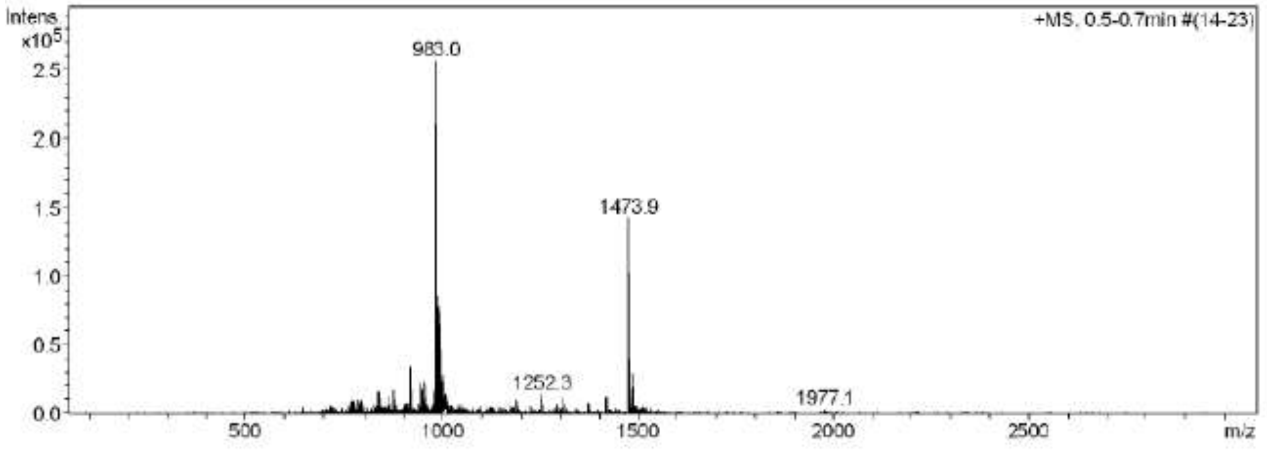
Intens.
x10^5
+MS, 0.5-0.7min #(14-23)
983.0
1473.9
1252.3
1977.1
m/z

HRMS (*m/z*)

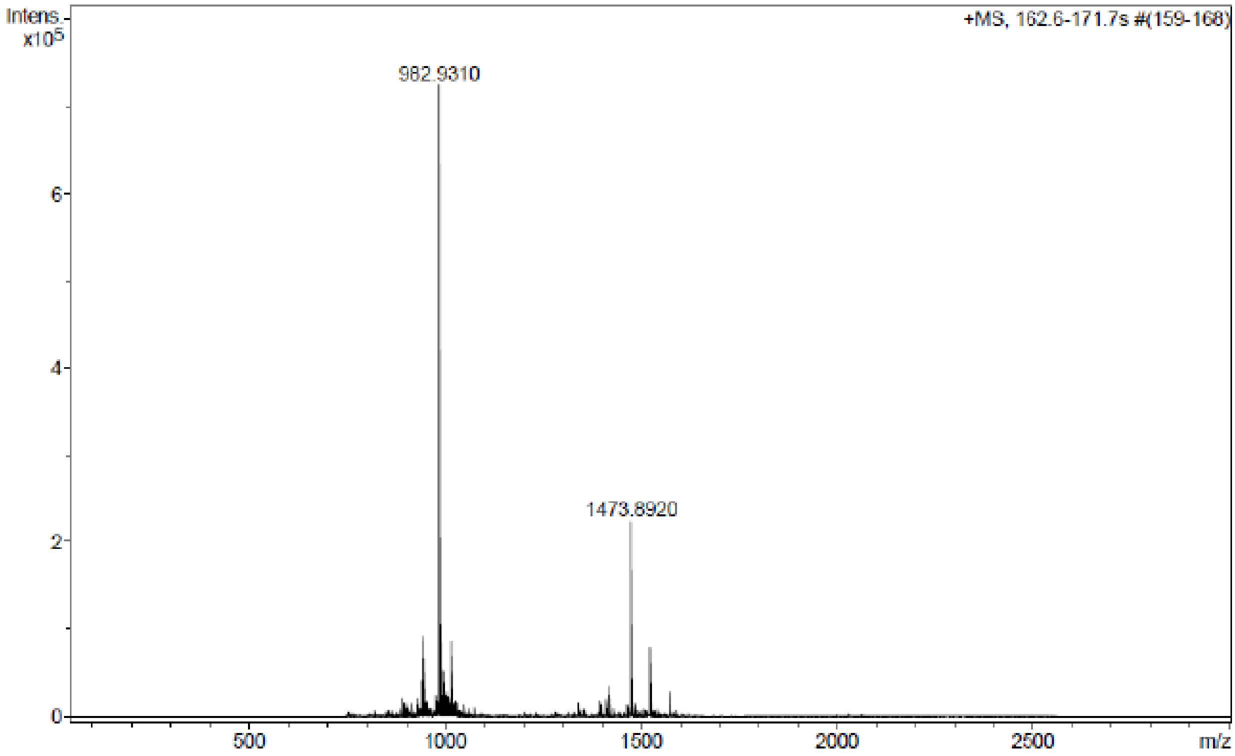
Intens.
x10^5
+MS, 162.6-171.7s #(159-168)
982.9310
1473.8920
6
4
2
0
500
1000
1500
2000
2500
m/z

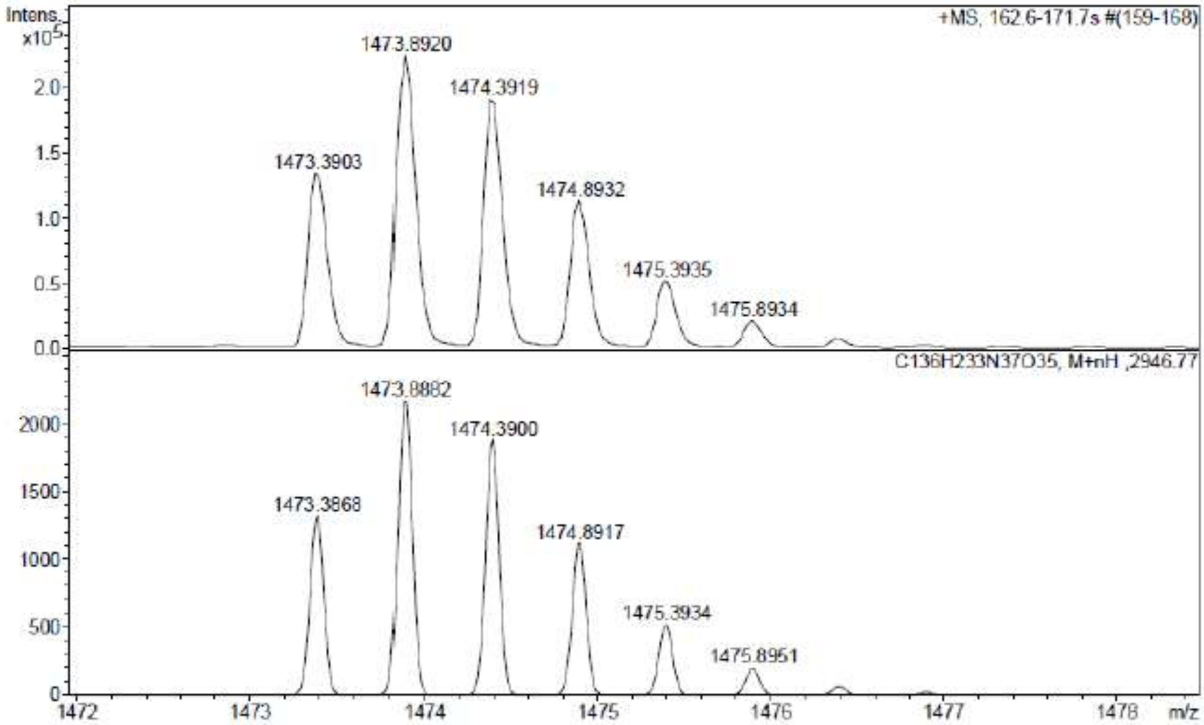
Intens.
x10^5
+MS, 162.6-171.7s #(159-168)
1473.8920
1474.3919
1473.3903
1474.8932
1475.3935
1475.8934
2.0
1.5
1.0
0.5
0.0
C136H233N37O35, M+nH ,2946.77
1473.8882
1474.3900
1473.3868
1474.8917
1475.3934
1475.8951
2000
1500
1000
500
0
1472
1473
1474
1475
1476
1477
1478
m/z

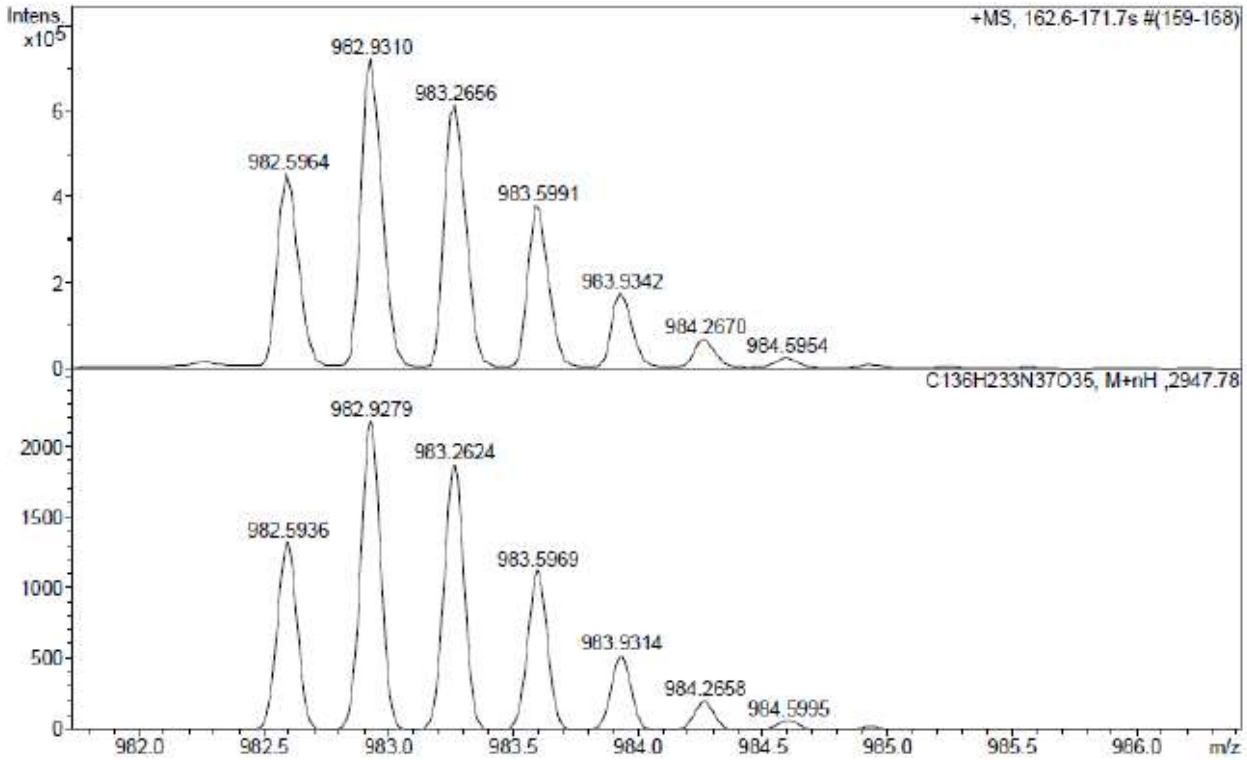

Intens.
x10^5
+MS, 162.6-171.7s #(159-168)
982.9310
983.2656
982.5964
983.5991
983.9342
984.2670
984.5954
C136H233N37O35, M+nH ,2947.78
982.9279
983.2624
982.5936
983.5969
983.9314
984.2658
984.5995
m/z

Ac-Arg-Ile-Asn-Ser-Ala-Lys-Asp-Asp-Ala-Ala-Gly-Leu-Gln-Ile-Ala-Lys-Lys-Leu-Phe-Lys-Lys-Ile-Lys(COC_3H_7)-Lys-Tyr-Leu-NH_2 (flg15-BP387)

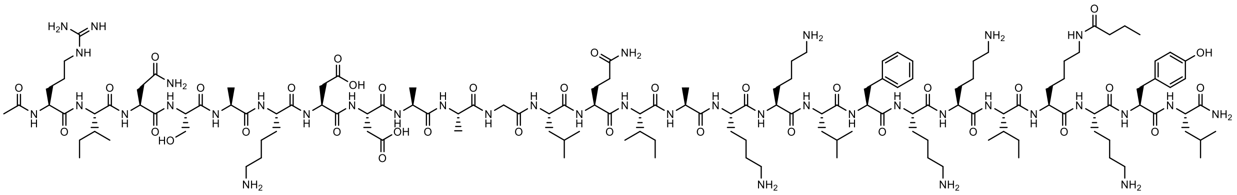

HPLC (λ=220 nm)

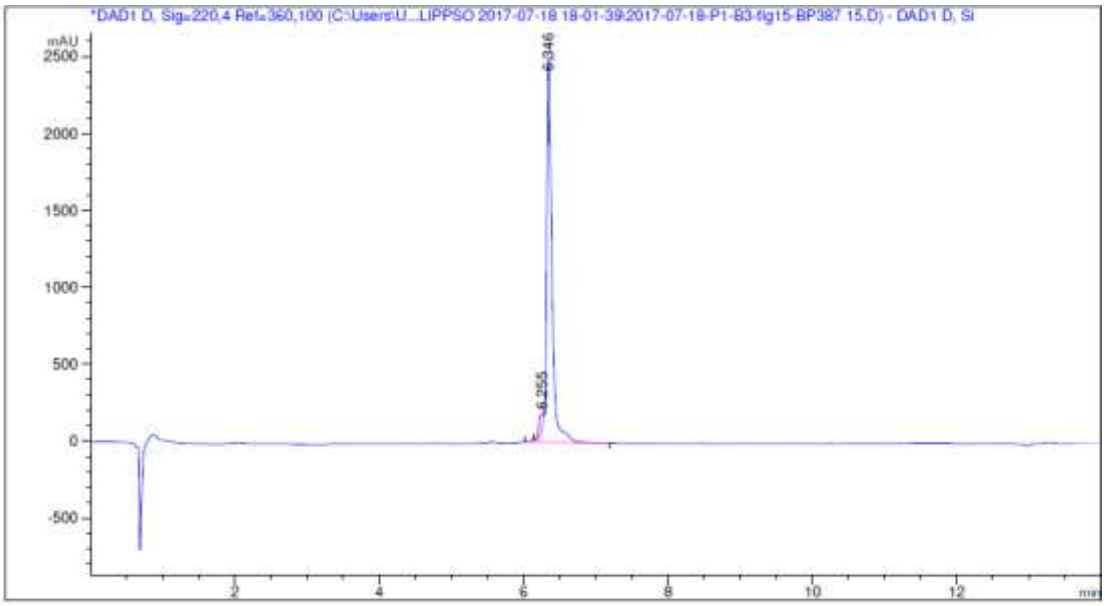

Peak #	RetTime [min]	Type	Width [min]	Area [mAU*s]	Height [mAU]	Area %
1	6.255	VV E	0.0724	496.30545	100.95900	3.6633
2	6.346	VB R	0.0768	1.30517e4	2504.88135	96.3367

ESI-MS (*m/z*)

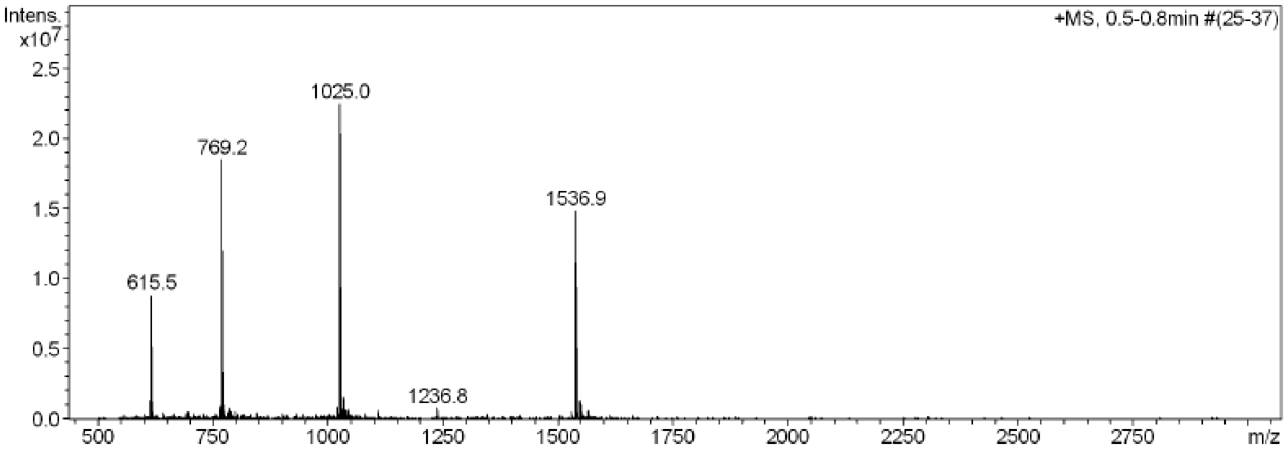

HRMS (*m/z*)

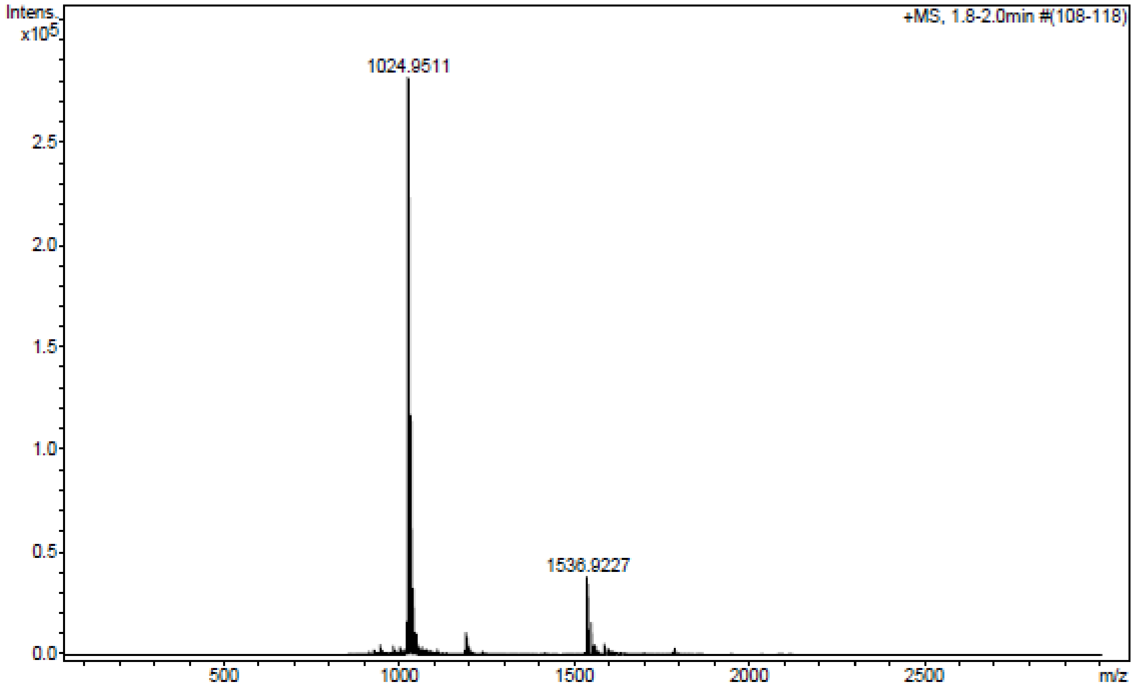
Intens.
x10^5
+MS, 1.8-2.0min #(108-118)
1024.9511
1536.9227
2.5
2.0
1.5
1.0
0.5
0.0
500
1000
1500
2000
2500
m/z

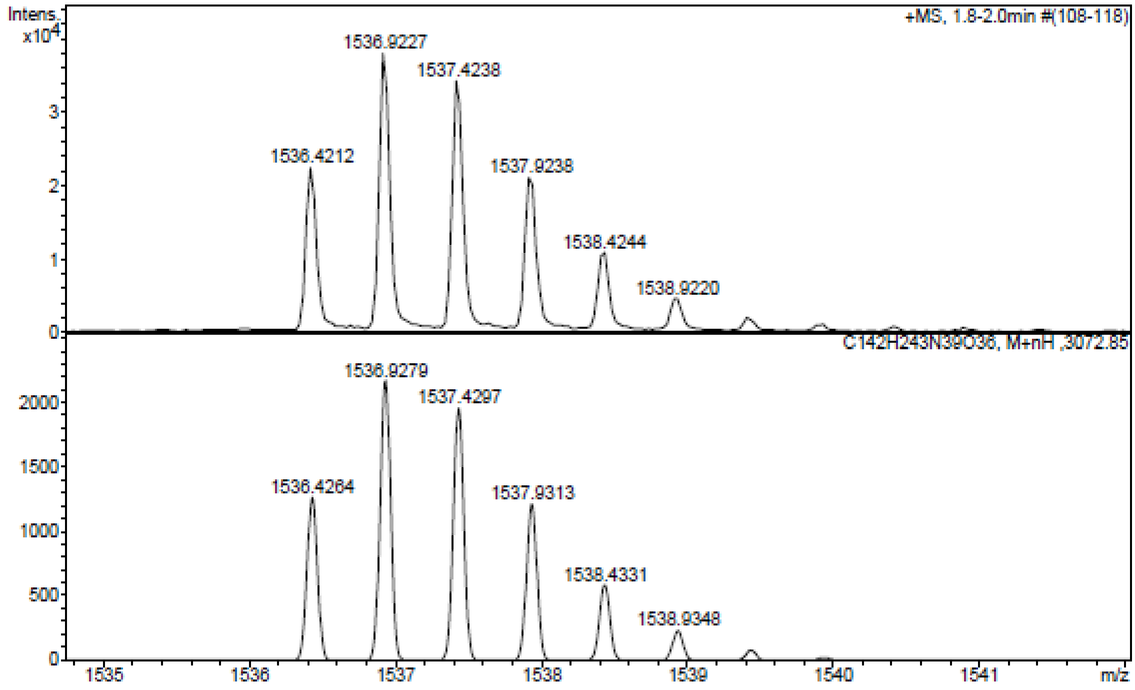
Intens.
x10^4
+MS, 1.8-2.0min #(108-118)
1536.9227
1537.4238
1536.4212
1537.9238
1538.4244
1538.9220
C142H243N39O36, M+nH ,3072.85
1536.9279
1537.4297
1536.4264
1537.9313
1538.4331
1538.9348
m/z

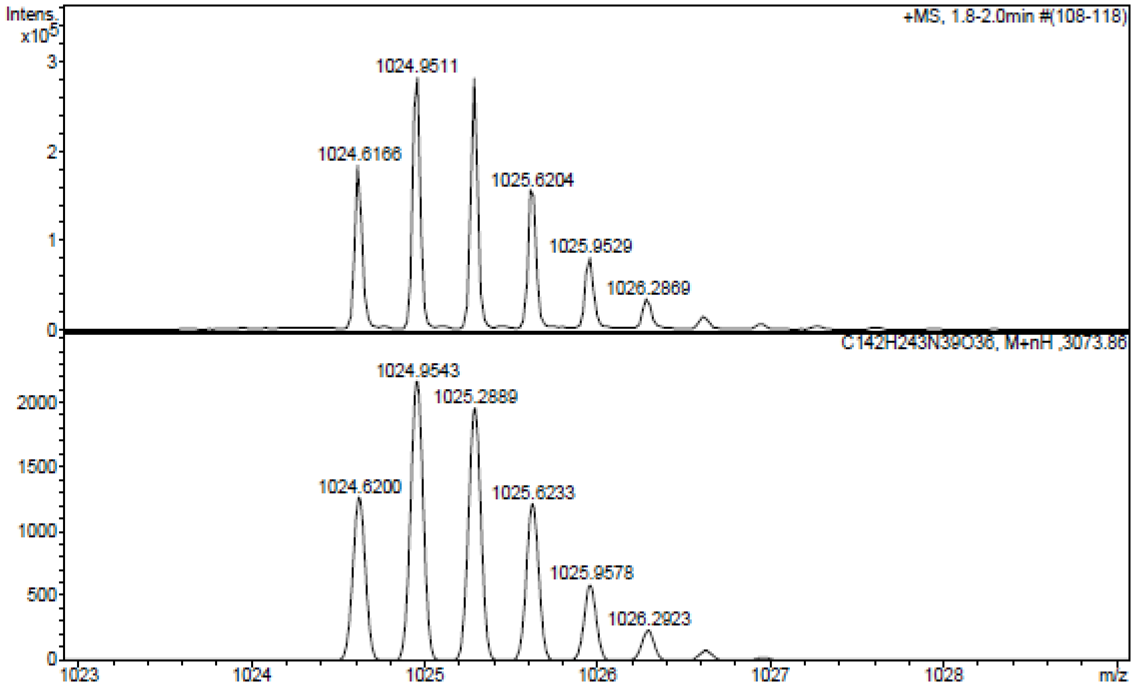
Intens.
x10^5
+MS, 1.8-2.0min #(108-118)
1024.9511
1024.6166
1025.6204
1025.9529
1026.2869
C142H243N39O36, M+nH ,3073.86
1024.9543
1025.2889
1024.6200
1025.6233
1025.9578
1026.2923
m/z

Ac-Lys-Lys-Leu-Phe-Lys-Lys-Ile-Lys(COC3H7)-Lys-Tyr-Leu-Arg-Ile-Asn-Ser-Ala-Lys-Asp-Asp-Ala-Ala-Gly-Leu-Gln-Ile-Ala-OH (BP387-flg15)

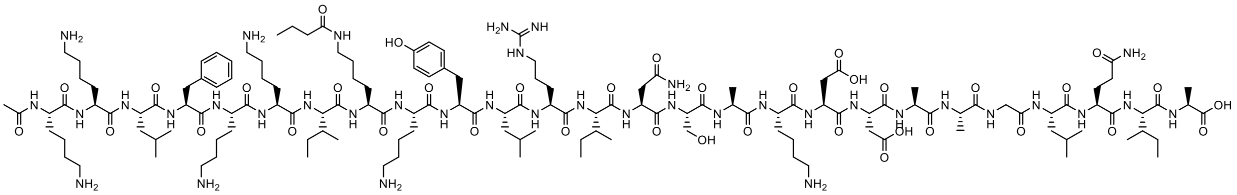

HPLC (λ=220 nm)

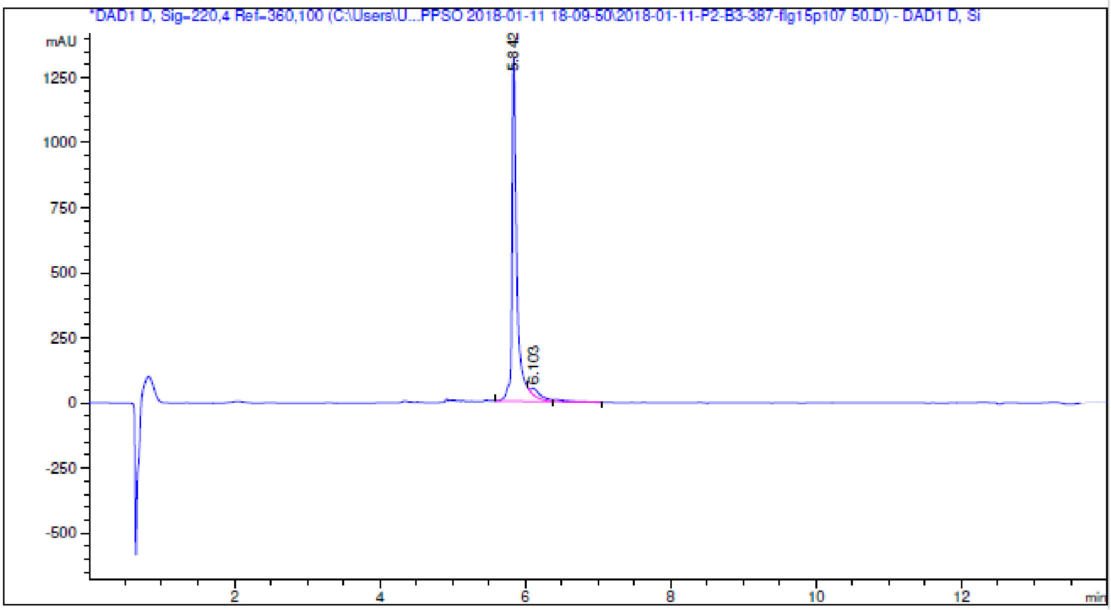

Peak #	RetTime [min]	Type	Width [min]	Area [mAU*s]	Height [mAU]	Area %
1	5.842	BV R	0.0681	6357.41650	1321.30750	95.8923
2	6.103	VV E	0.1565	272.32904	23.98207	4.1077
Totals :				6629.74554	1345.28957	

ESI-MS (*m/z*)

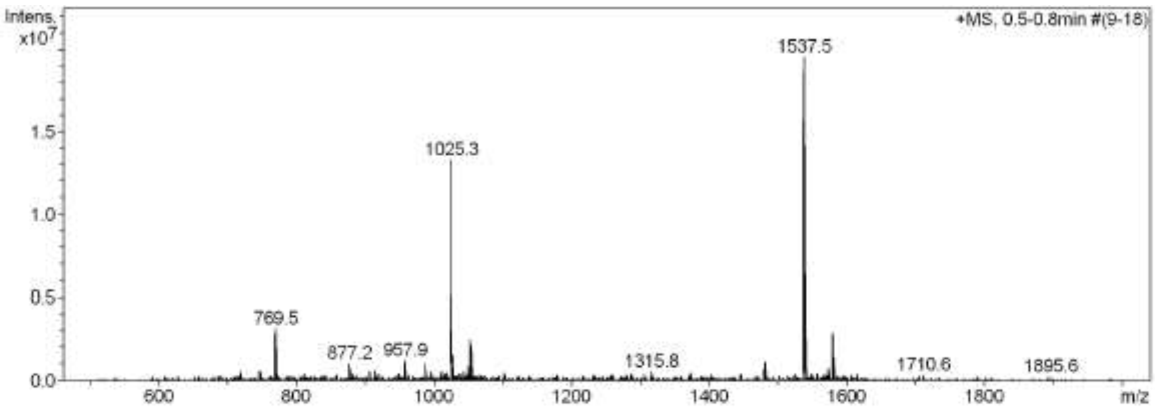

HRMS (*m/z*)

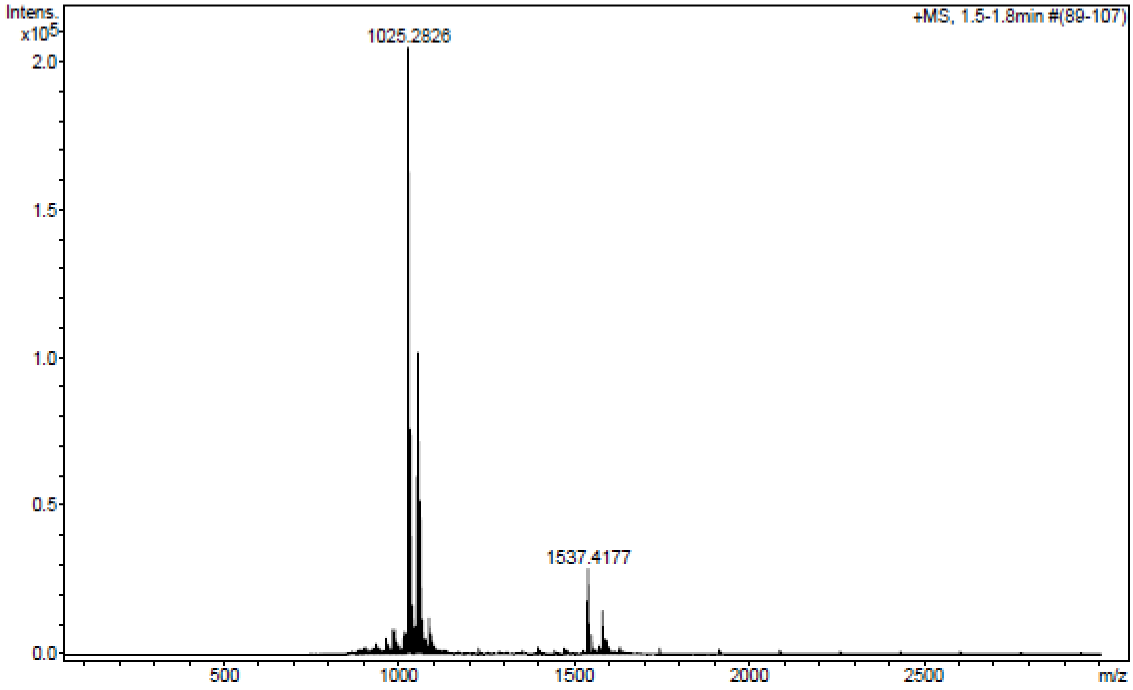
Intens.
x10^5
+MS, 1.5-1.8min #(89-107)
1025.2826
1537.4177
2.0
1.5
1.0
0.5
0.0
500
1000
1500
2000
2500
m/z

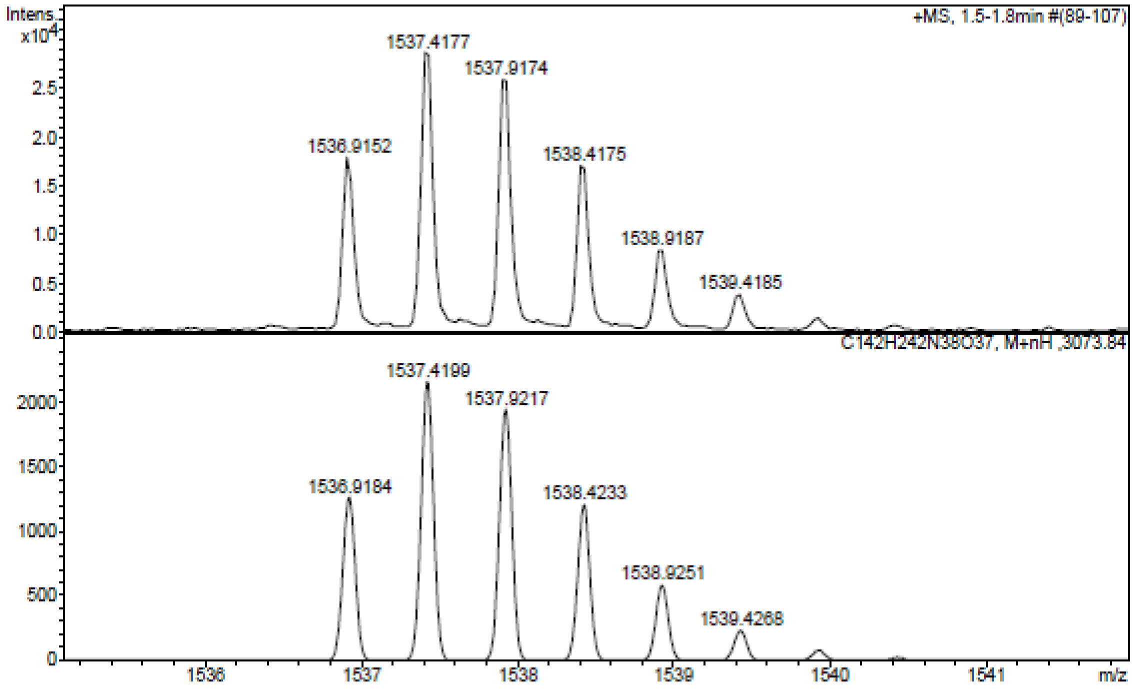
Intens.
x10^4
+MS, 1.5-1.8min #(89-107)
1537.4177
1537.9174
1536.9152
1538.4175
1538.9187
1539.4185
C142H242N38O37, M+nH ,3073.84
1537.4199
1537.9217
1536.9184
1538.4233
1538.9251
1539.4268
2.5
2.0
1.5
1.0
0.5
0.0
2000
1500
1000
500
0
1536
1537
1538
1539
1540
1541
m/z

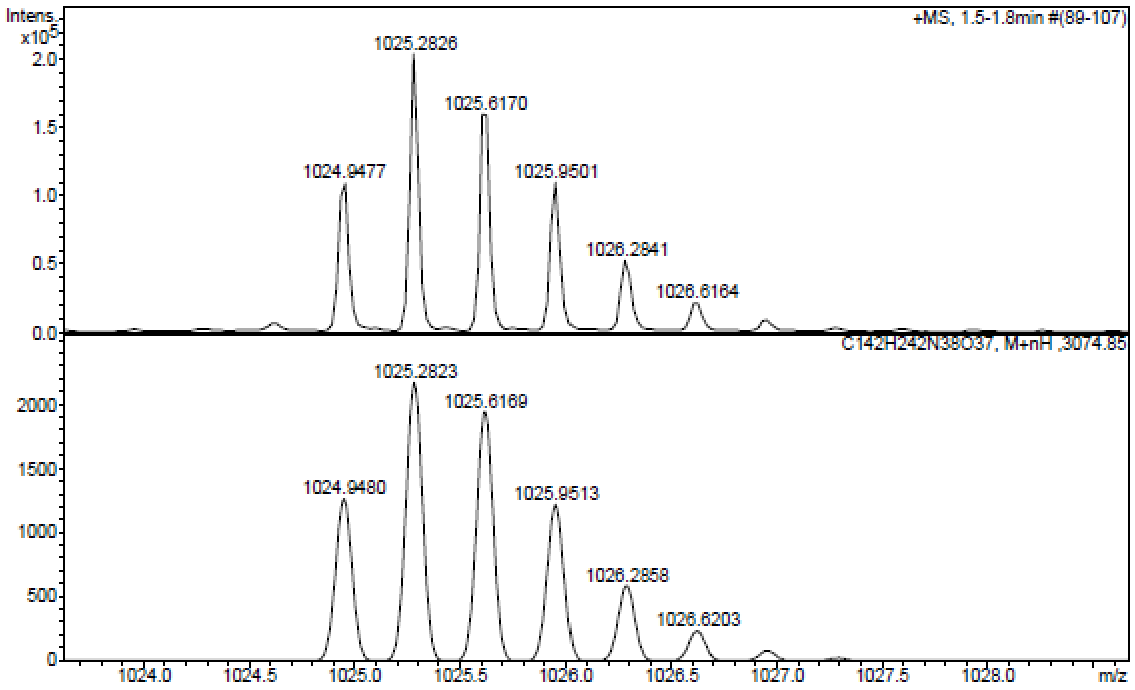

Intens.
x10^5
+MS, 1.5-1.8min #(89-107)
1025.2826
1025.6170
1024.9477
1025.9501
1026.2841
1026.6164
C142H242N38O37, M+nH ,3074.85
1025.2823
1025.6169
1024.9480
1025.9513
1026.2858
1026.6203
m/z

Ac-Arg-Ile-Asn-Ser-Ala-Lys-Asp-Asp-Ala-Ala-Gly-Leu-Gln-Ile-Ala-Lys-Lys-Leu-D-Phe-Lys-Lys-Ile-Leu-Lys-Lys(COC$_3$H$_7$)-Leu-NH$_2$ (flg15-BP475)

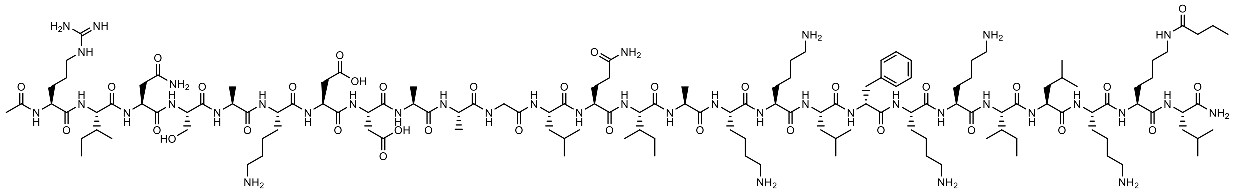

HPLC (λ=220 nm)

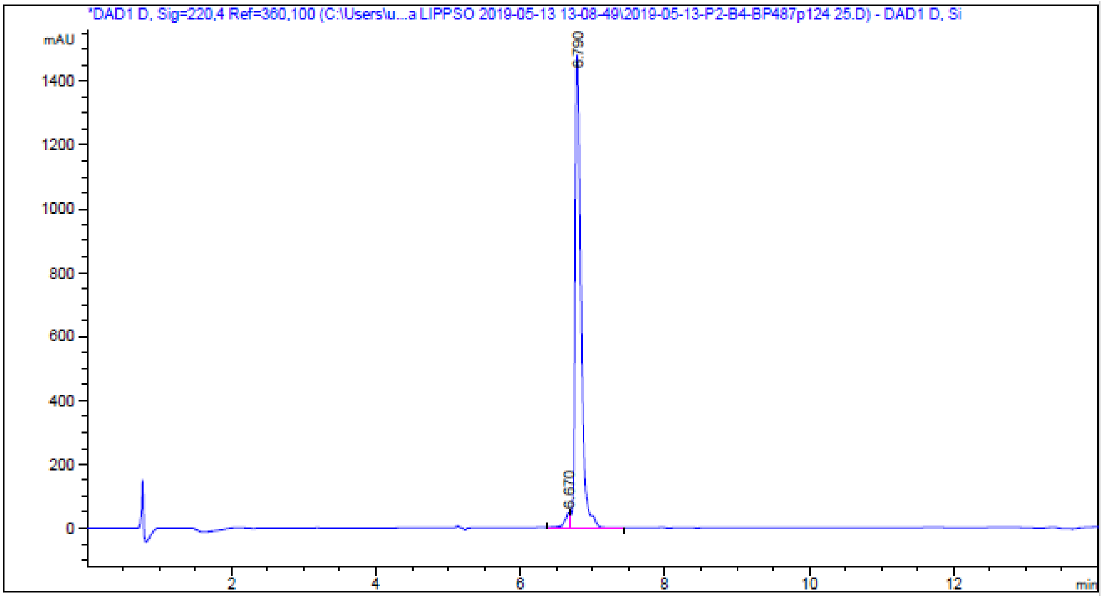

Peak #	RetTime [min]	Type	Width [min]	Area [mAU*s]	Height [mAU]	Area %
1	6.670	HH	0.0798	288.50366	49.63588	3.2581
2	6.790	HH	0.0894	8566.53027	1482.00330	96.7419

Totals : 8855.03394 1531.63918

ESI-MS (*m/z*)

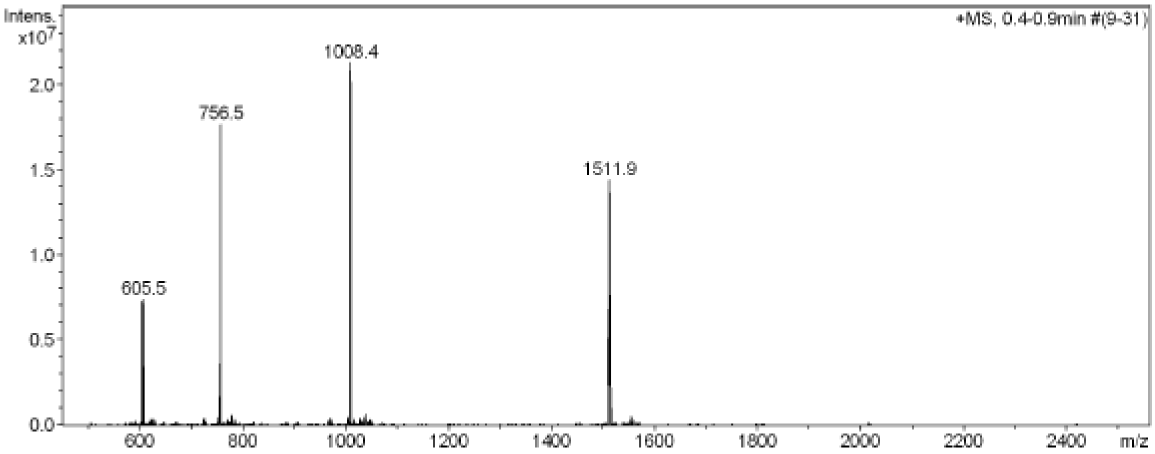

HRMS (*m/z*)

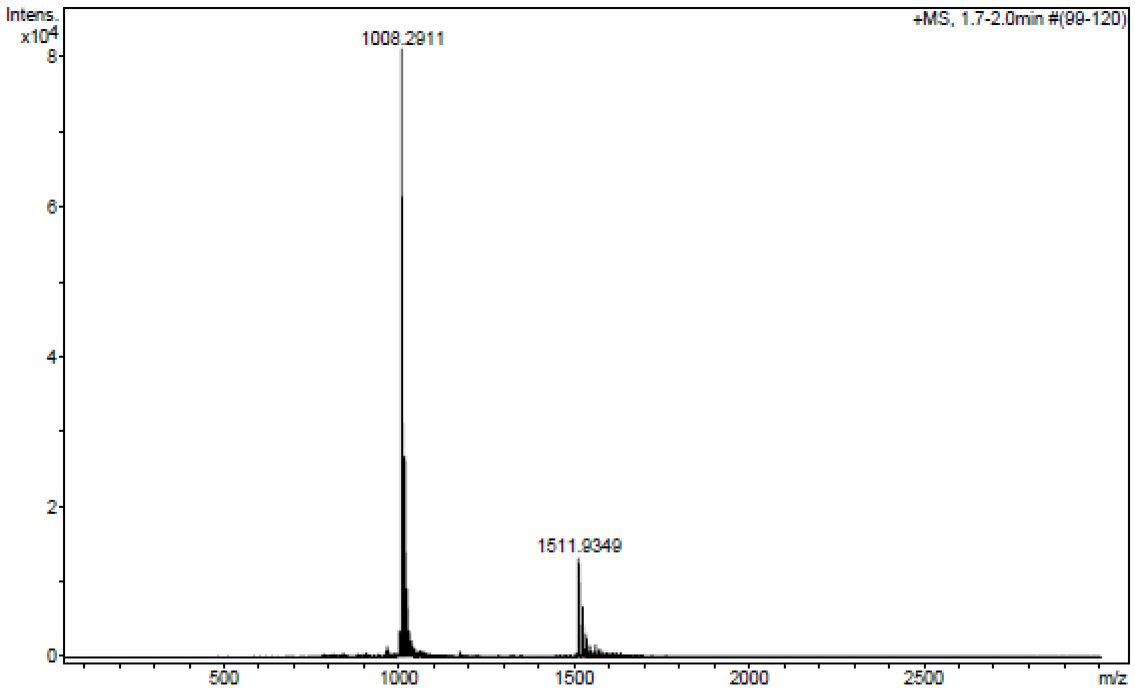
Intens.
x10^4
+MS, 1.7-2.0min #(99-120)
1008.2911
1511.9349
m/z

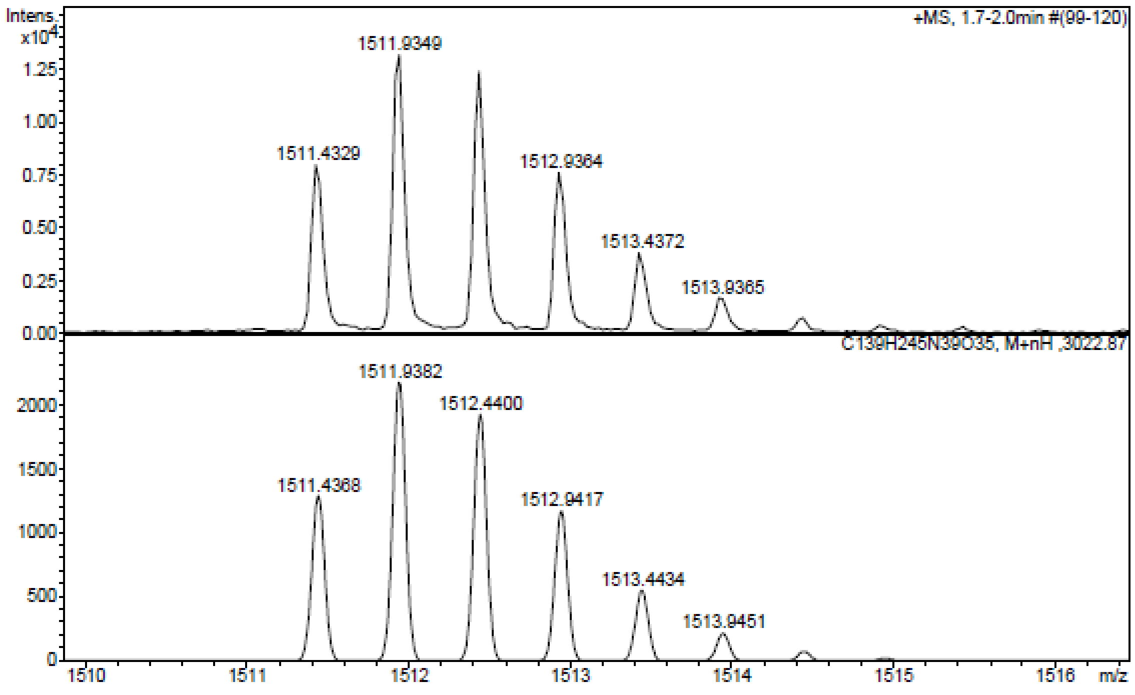
Intens.
x10^4
+MS, 1.7-2.0min #(99-120)
1511.9349
1511.4329
1512.9364
1513.4372
1513.9365
C139H245N39O35, M+nH ,3022.87
1511.9382
1512.4400
1511.4368
1512.9417
1513.4434
1513.9451
m/z

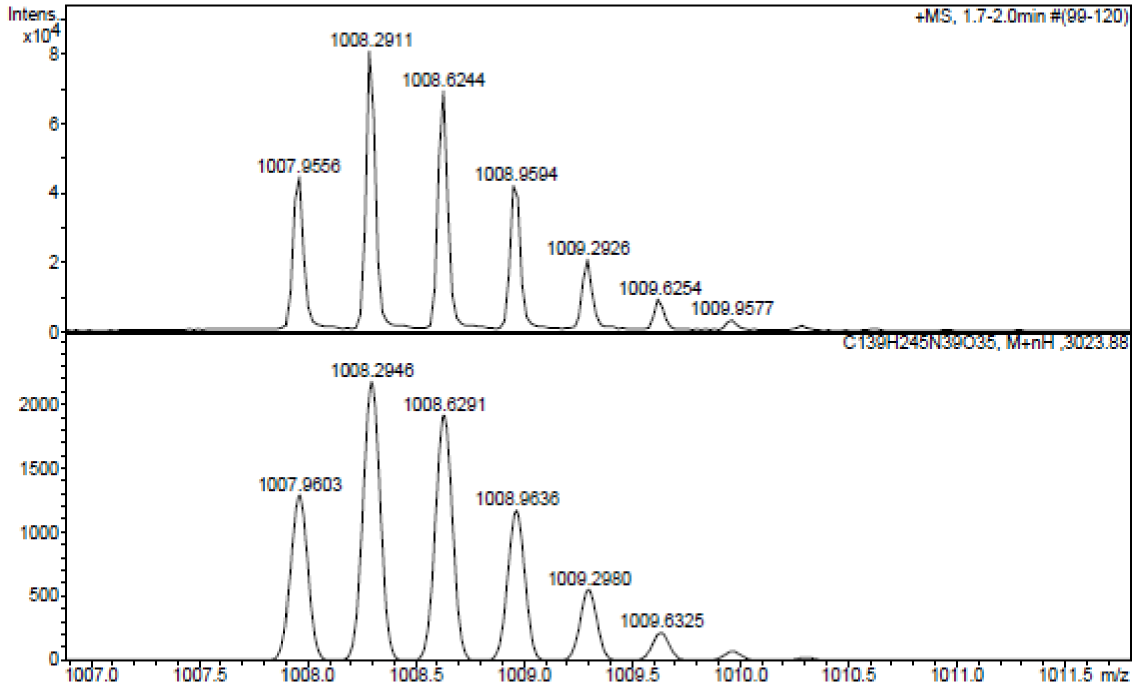
Intens.
x10^4
+MS, 1.7-2.0min #(99-120)
1008.2911
1008.6244
1007.9556
1008.9594
1009.2926
1009.6254
1009.9577
C139H245N39O35, M+nH ,3023.88
1008.2946
1008.6291
1007.9603
1008.9636
1009.2980
1009.6325
m/z

Ac-Lys-Lys-Leu-D-Phe-Lys-Lys-Ile-Leu-Lys-Lys(COC_3H_7)-Leu-Arg-Ile-Asn-Ser-Ala-Lys-Asp-Asp-Ala-Ala-Gly-Leu-Gln-Ile-Ala-OH (BP475-flg15)

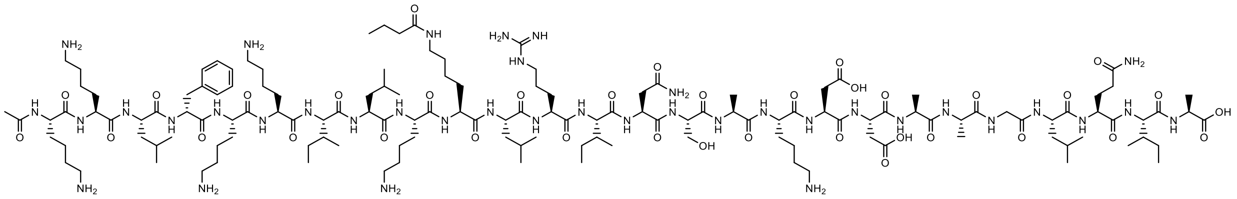

HPLC (λ=220 nm)

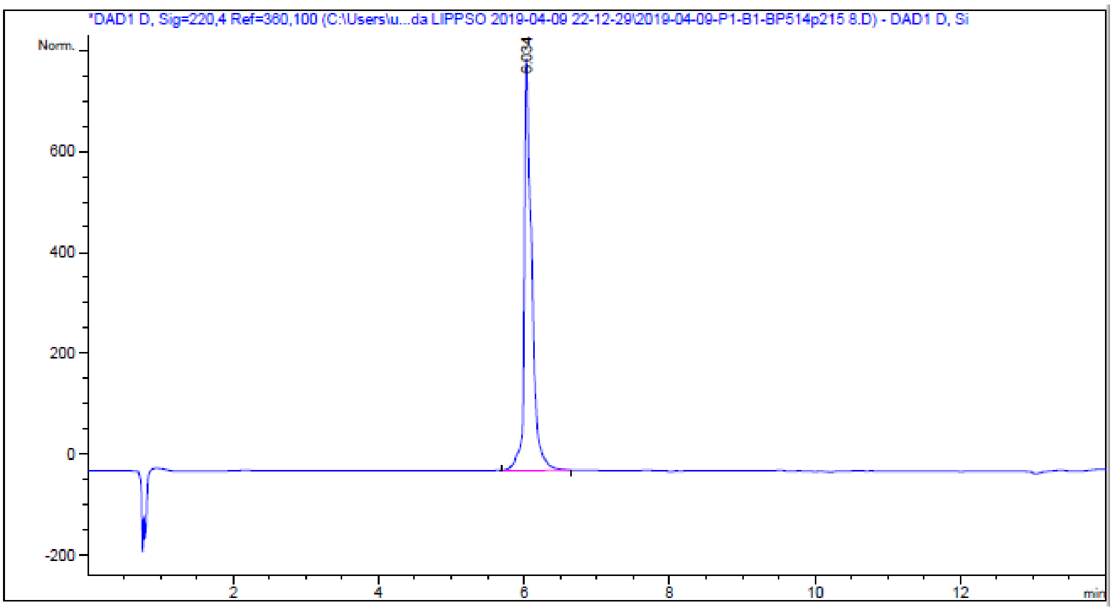

Peak #	RetTime [min]	Type	Width [min]	Area [mAU*s]	Height [mAU]	Area %
1	6.034	BB	0.0976	5274.44629	736.12421	100.0000
Totals :				5274.44629	736.12421	

ESI-MS (*m/z*)

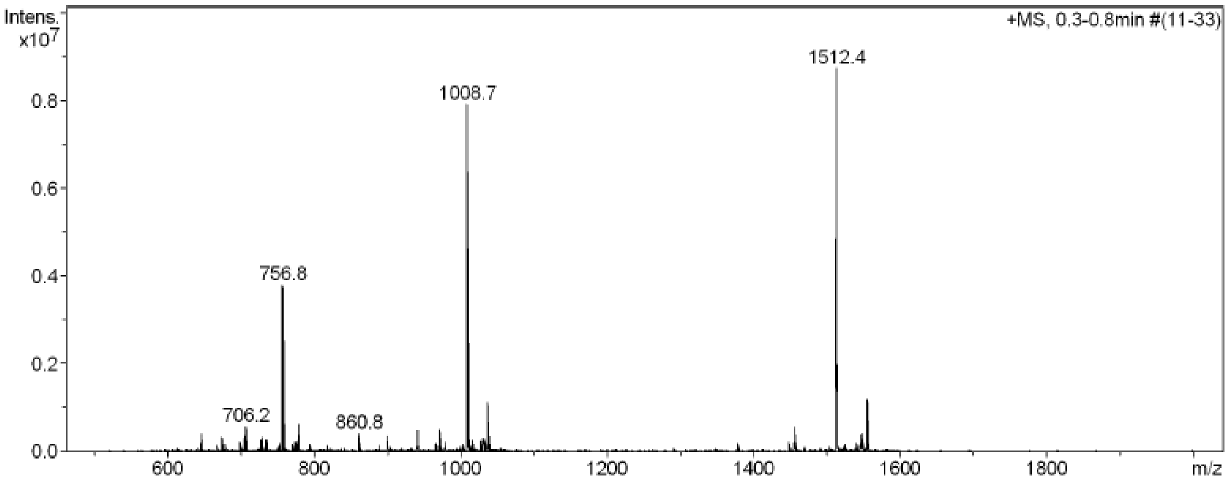

HRMS (m/z)

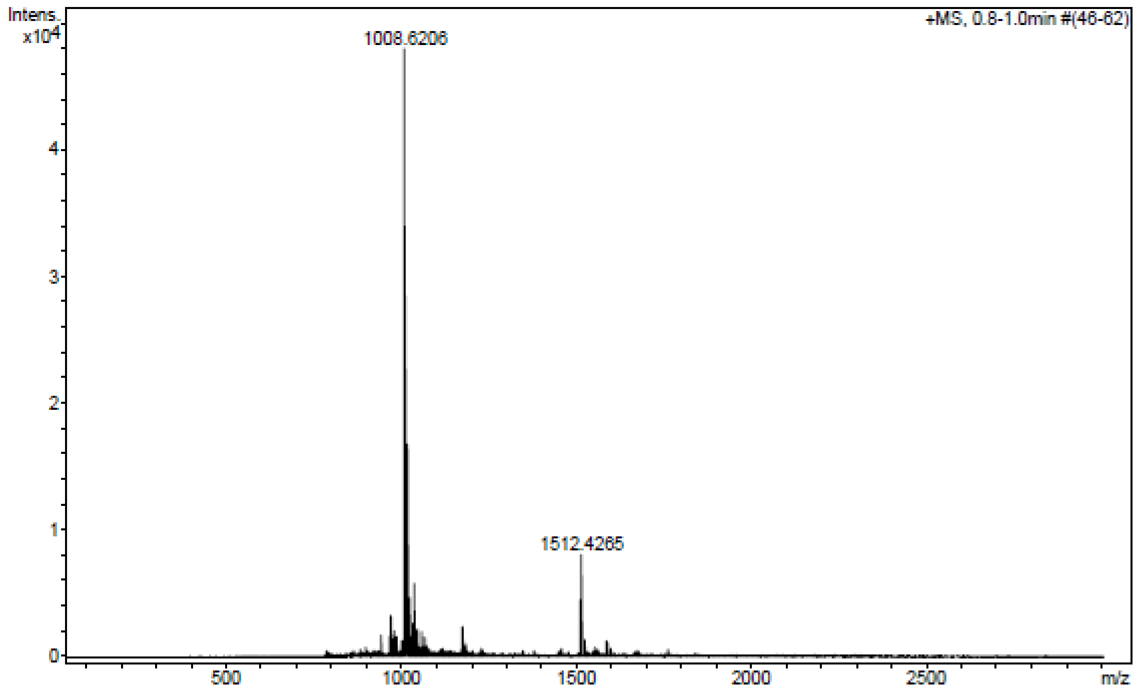
Intens.
x10^4
+MS, 0.8-1.0min #(46-62)
1008.6206
1512.4265
m/z

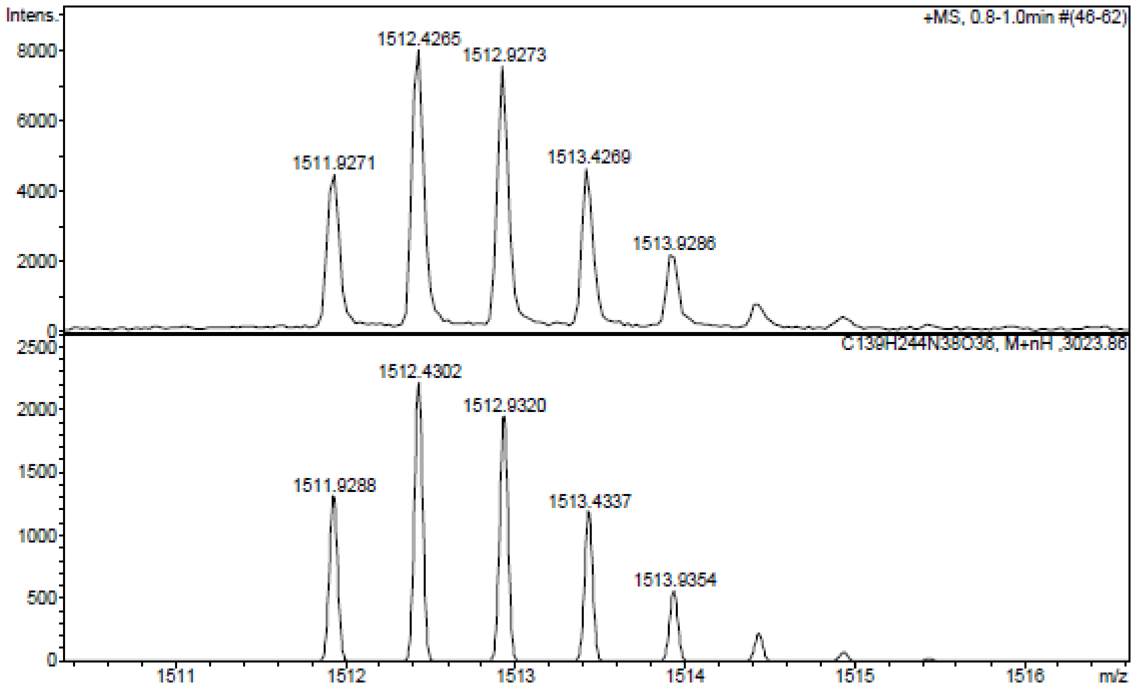
Intens.
+MS, 0.8-1.0min #(46-62)
1512.4265
1512.9273
1511.9271
1513.4269
1513.9286
C139H244N38O36, M+nH ,3023.86
1512.4302
1512.9320
1511.9288
1513.4337
1513.9354
m/z

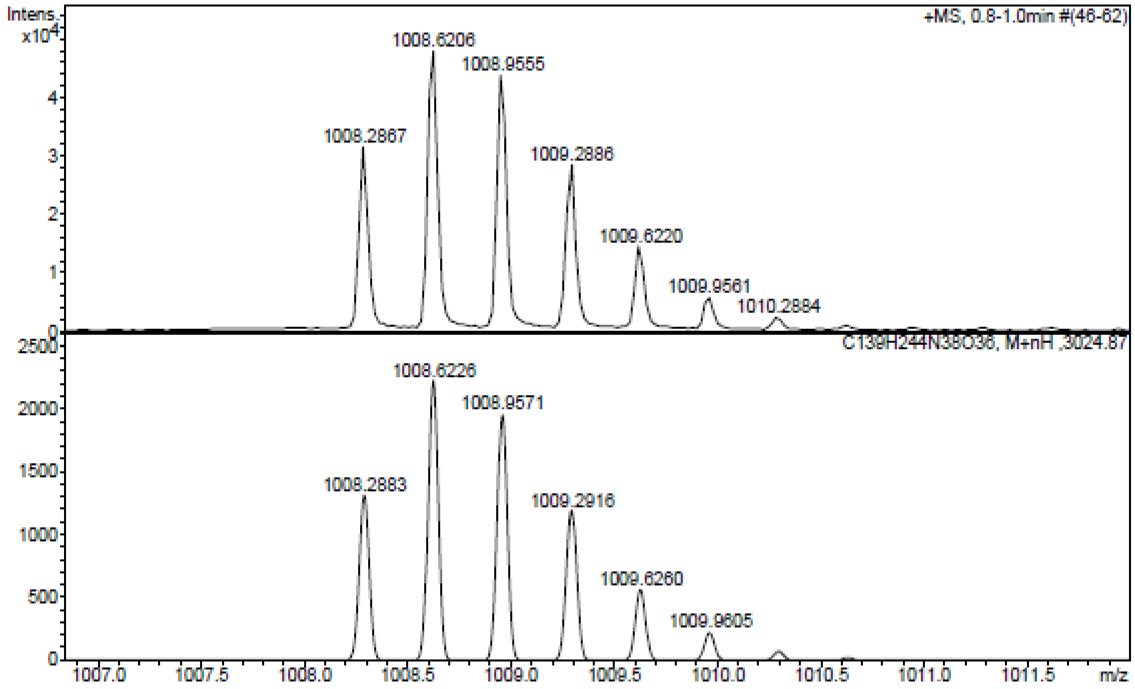

Intens.
x10^4
+MS, 0.8-1.0min #(46-62)
1008.2867
1008.6206
1008.9555
1009.2886
1009.6220
1009.9561
1010.2884
C139H244N38O36, M+nH ,3024.87
1008.2883
1008.6226
1008.9571
1009.2916
1009.6260
1009.9605
m/z

Arg-Ile-Asn-Ser-Ala-Lys-Asp-Asp-Ala-Ala-Gly-Leu-Gln-Ile-Ala-Lys-Lys-Val-Val-Phe-Trp-Val-Lys-Phe-Lys-NH_2 (flg15-KSLW)

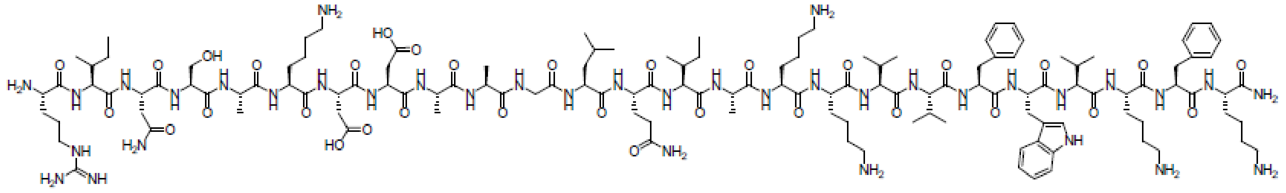

HPLC (λ=220 nm)

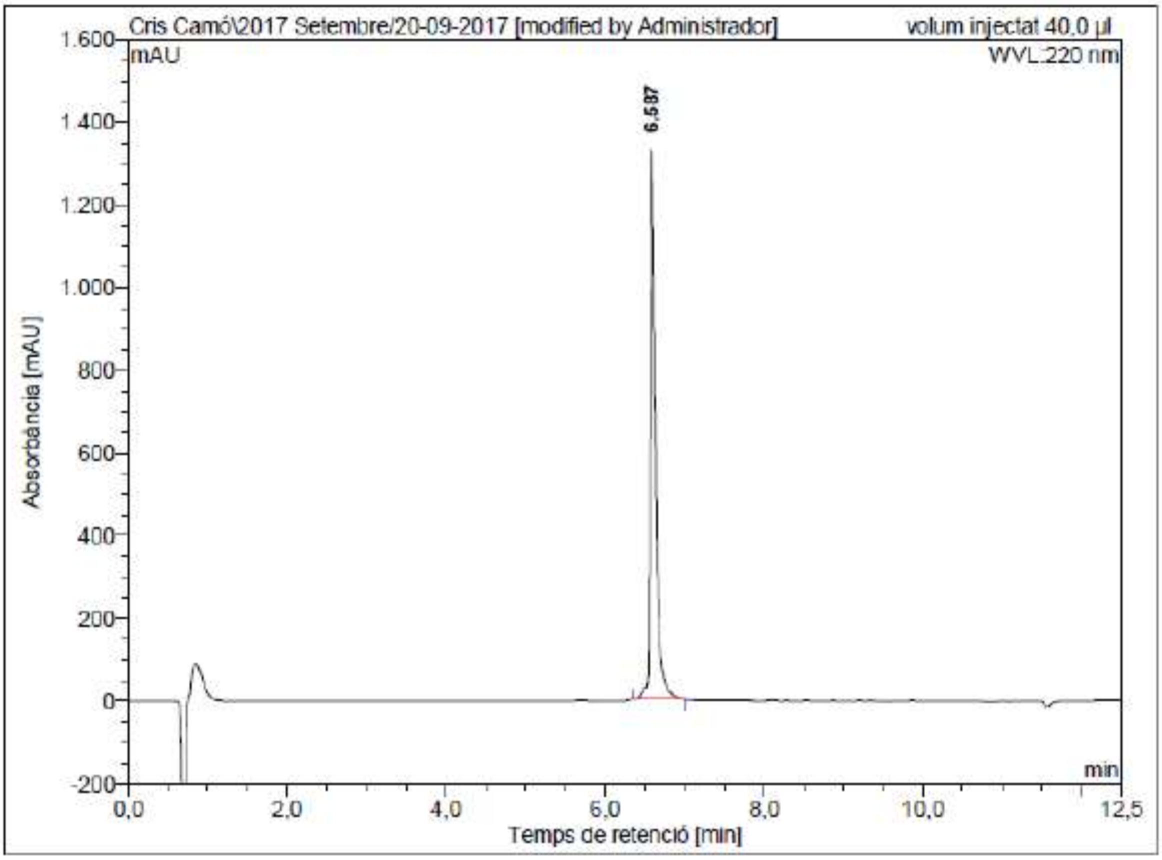

No.	Temps retenció<br>min	alçada<br>mAU	Area<br>mAU*min	Area relativa<br>%
1	6,59	1331,652	106,809	100,00
Total:		1331,652	106,809	100,00

ESI-MS (*m/z*)

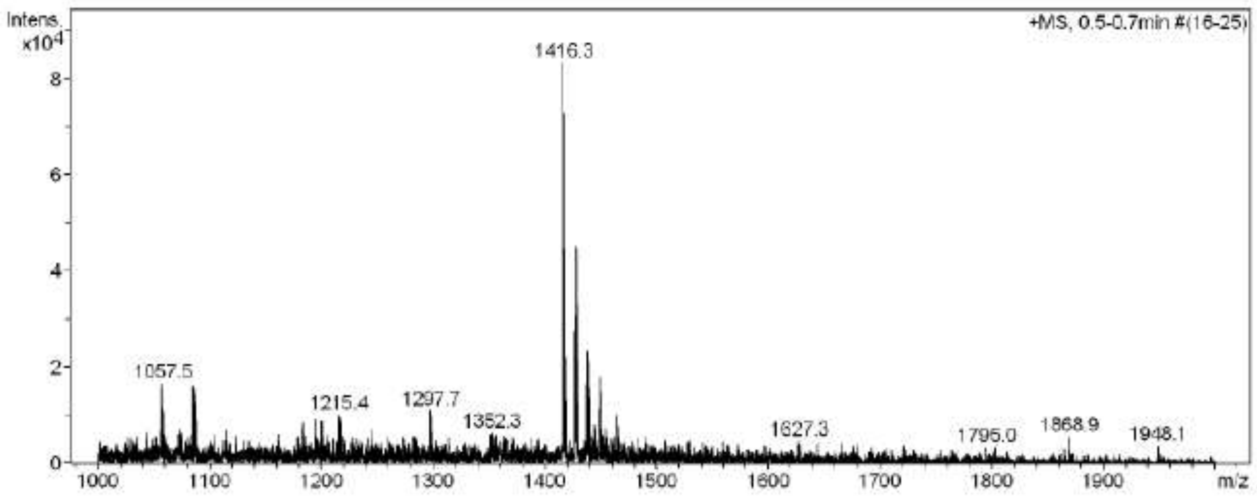

HRMS (*m/z*)

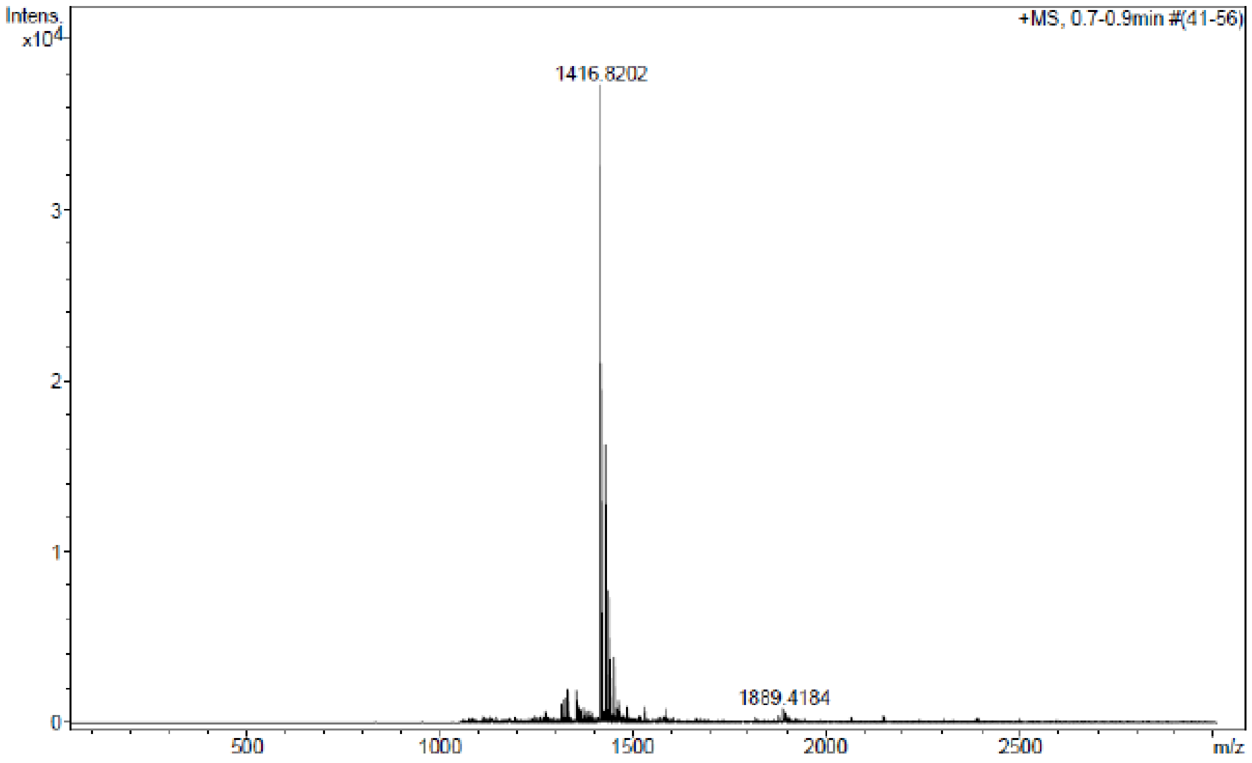

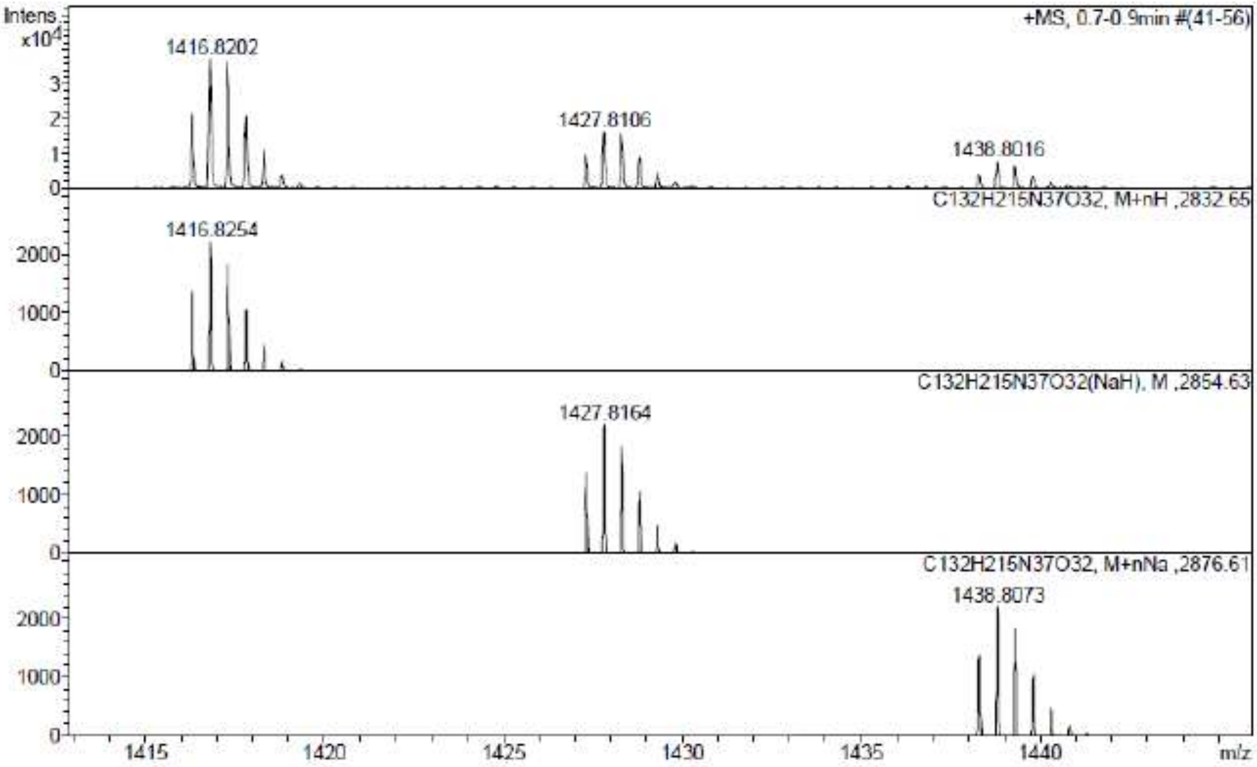
Intens. x10^4
+MS, 0.7-0.9min #(41-56)
1416.8202
1427.8106
1438.8016
C132H215N37O32, M+nH ,2832.65
1416.8254
C132H215N37O32(NaH), M ,2854.63
1427.8164
C132H215N37O32, M+nNa ,2876.61
1438.8073
m/z

Lys-Lys-Val-Val-Phe-Trp-Val-Lys-Phe-Lys-Arg-Ile-Asn-Ser-Ala-Lys-Asp-Asp-Ala-Ala-Gly-Leu-Gln-Ile-Ala-OH (KSLW-flg15)

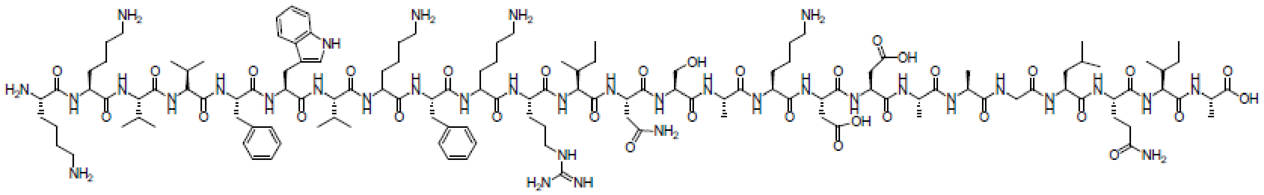

HPLC (λ=220 nm)

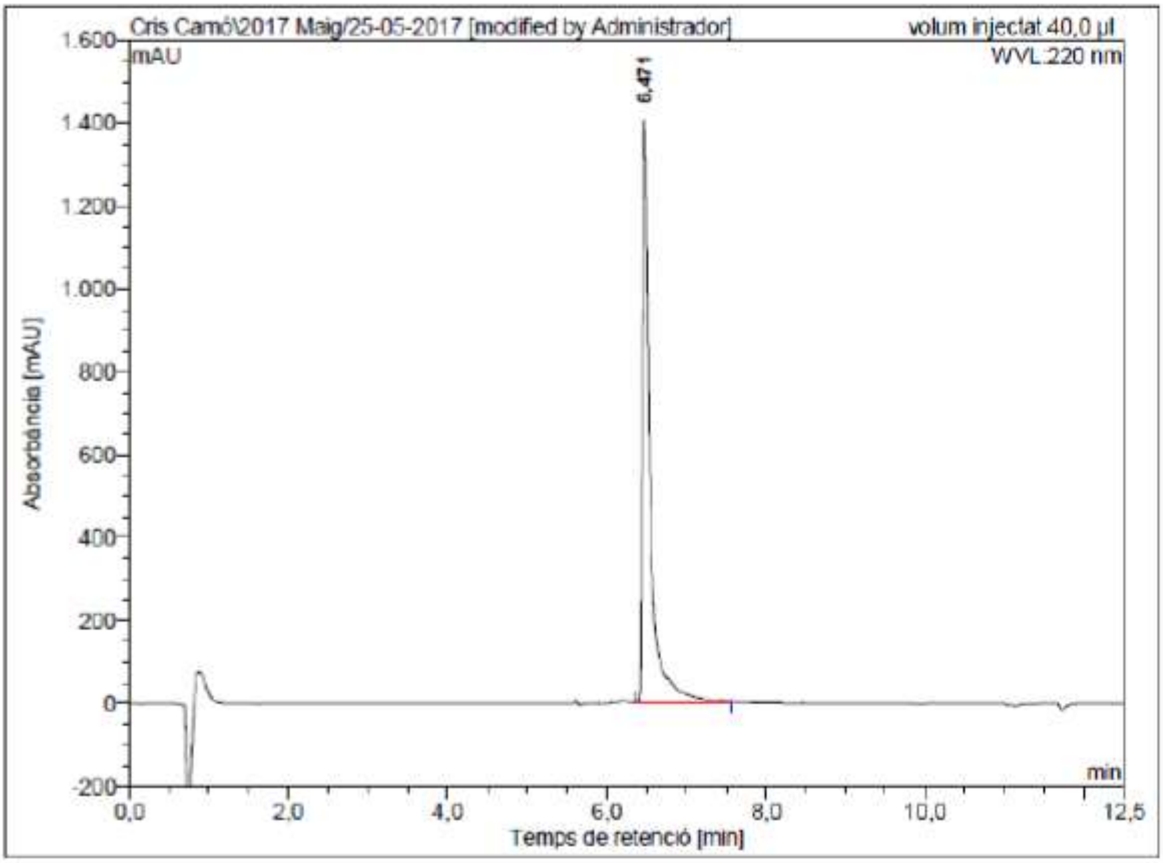

No.	Temps retenció<br>min	alçada<br>mAU	Area<br>mAU*min	Area relativa<br>%
1	6,47	1407,259	163,110	100,00
Total:		1407,259	163,110	100,00

ESI-MS (*m/z*)

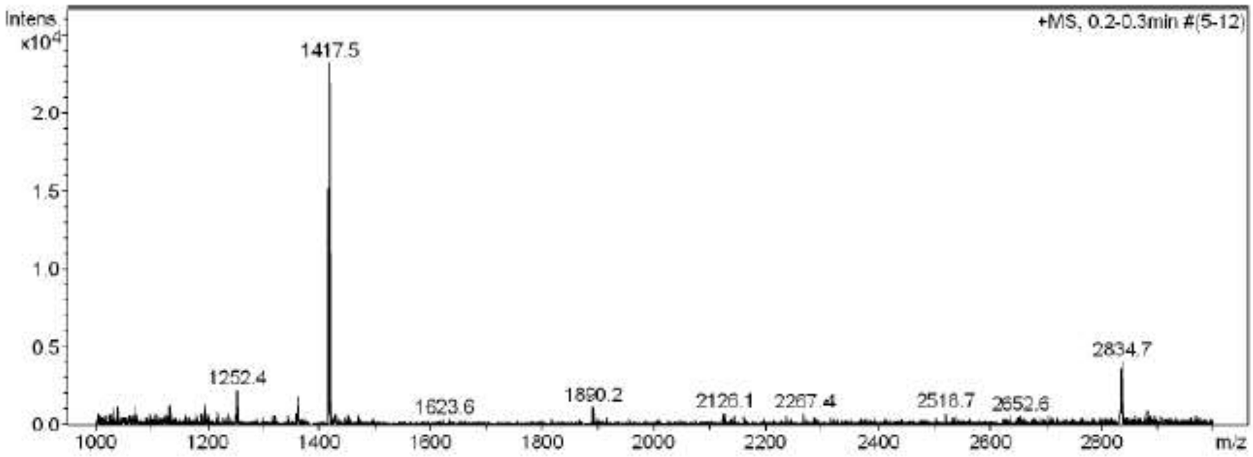

HRMS (*m/z*)

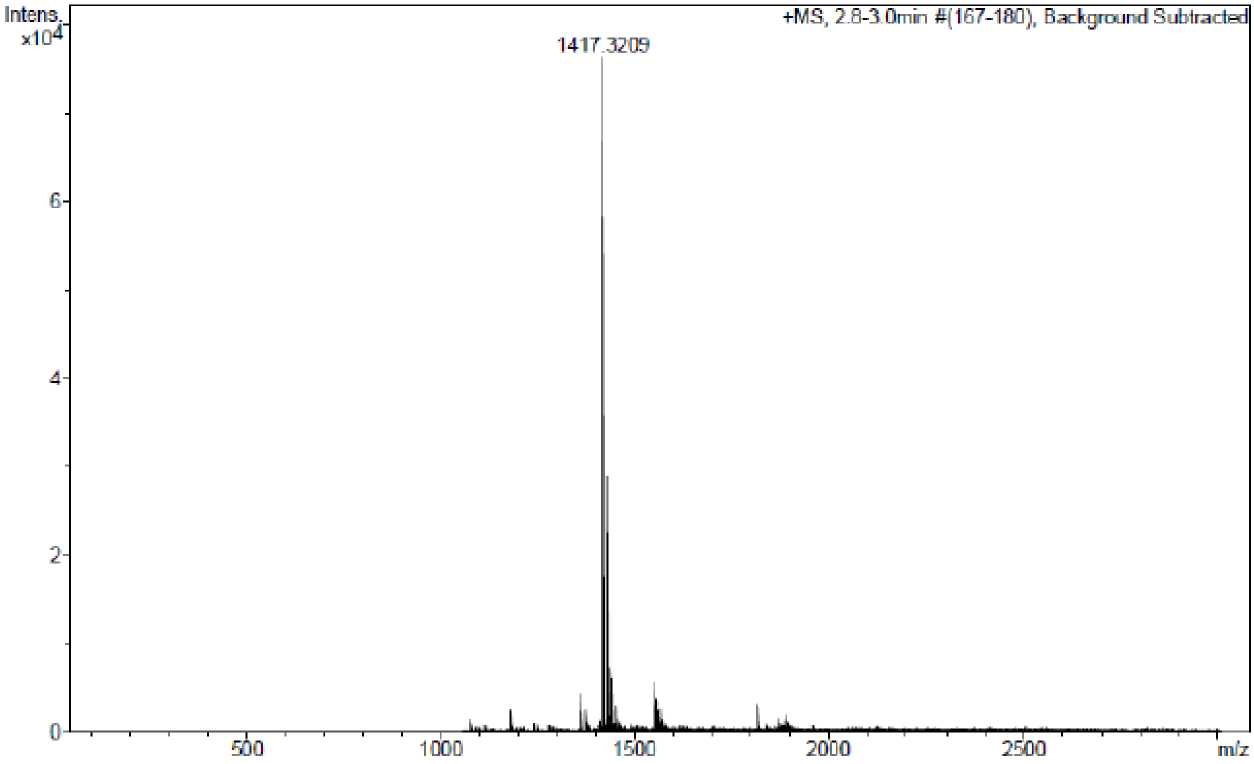

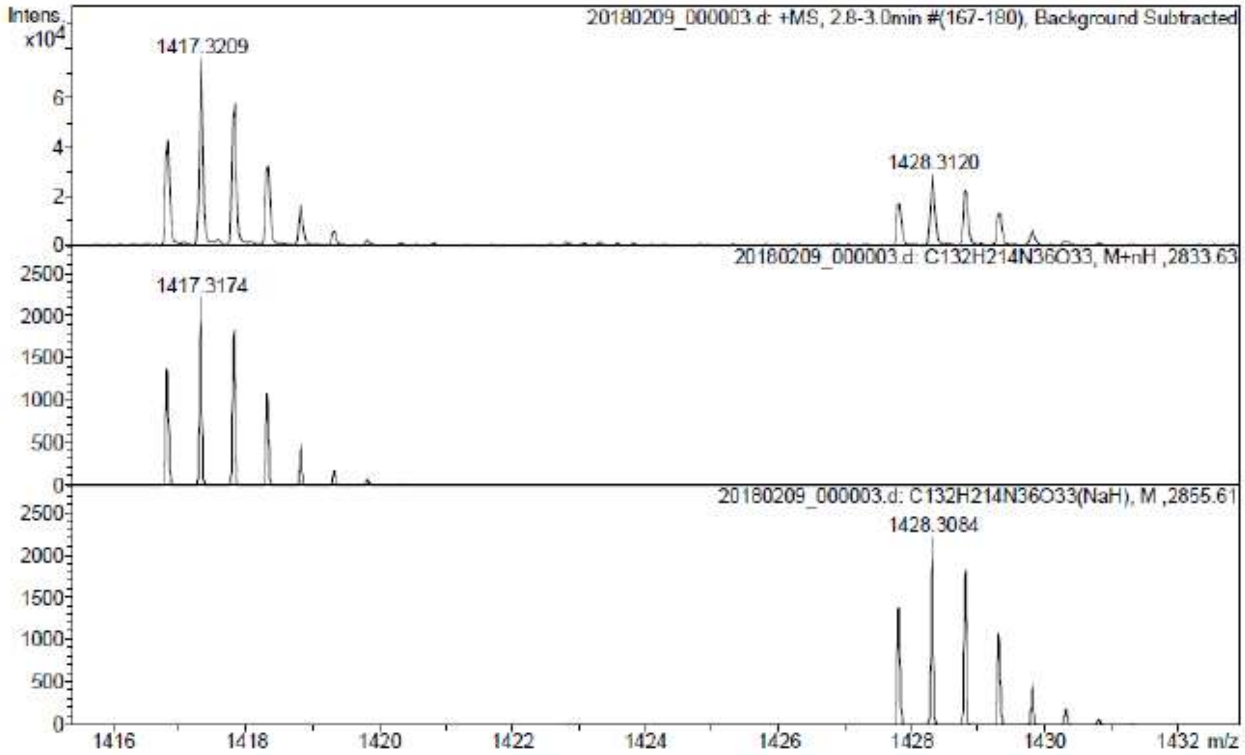
Intens. x10^4
20180209_000003.d: +MS, 2.8-3.0min #(167-180), Background Subtracted
1417.3209
1428.3120
20180209_000003.d: C132H214N36O33, M+nH ,2833.63
1417.3174
20180209_000003.d: C132H214N36O33(NaH), M ,2855.61
1428.3084
m/z

Phe-Lys-Leu-Phe-Lys-Lys-Ile-Leu-Lys-Val-Leu-Lys-Lys-Leu-Phe-Lys-Lys-Ile-Leu-Lys-Lys-Leu-NH_2 (BP13-BP16)

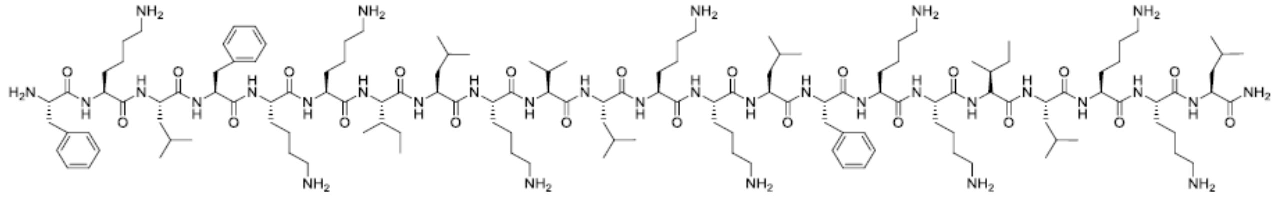

HPLC (λ=220 nm)

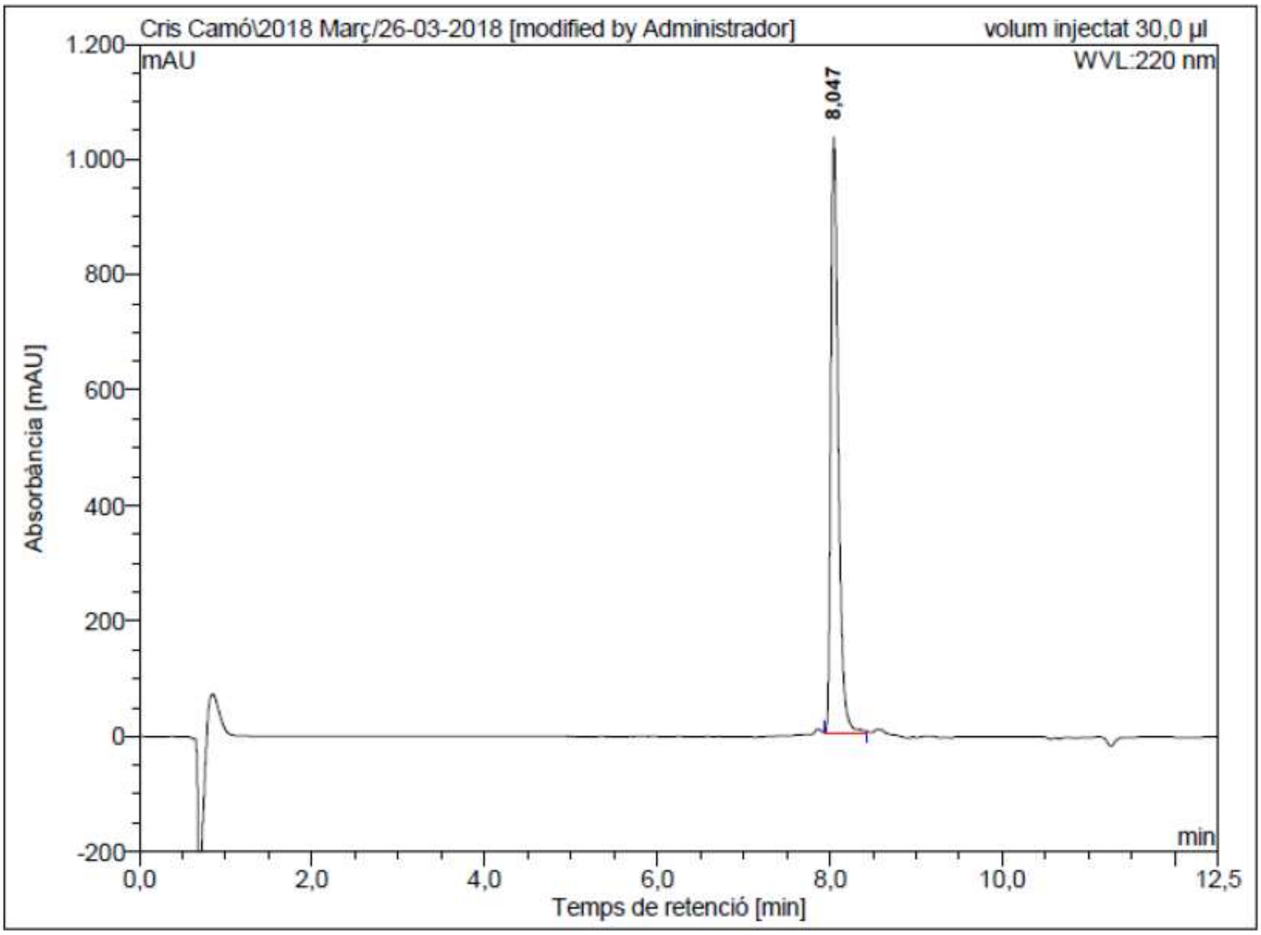

No.	Temps retenció min	alçada mAU	Area mAU*min	Area relativa %
1	8,05	1030,543	102,971	100,00
Total:		1030,543	102,971	100,00

ESI-MS (*m/z*)

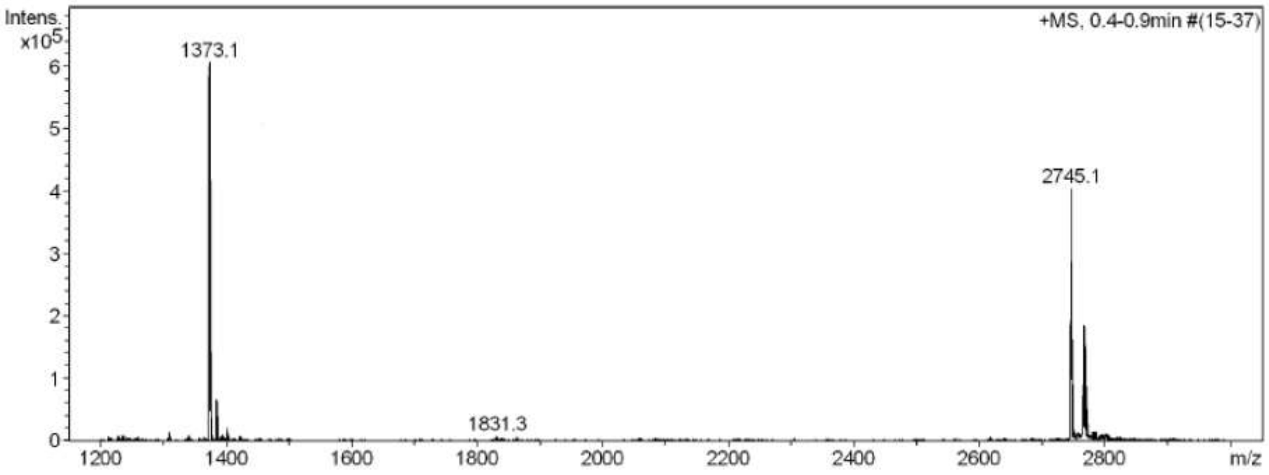

HRMS (*m/z*)

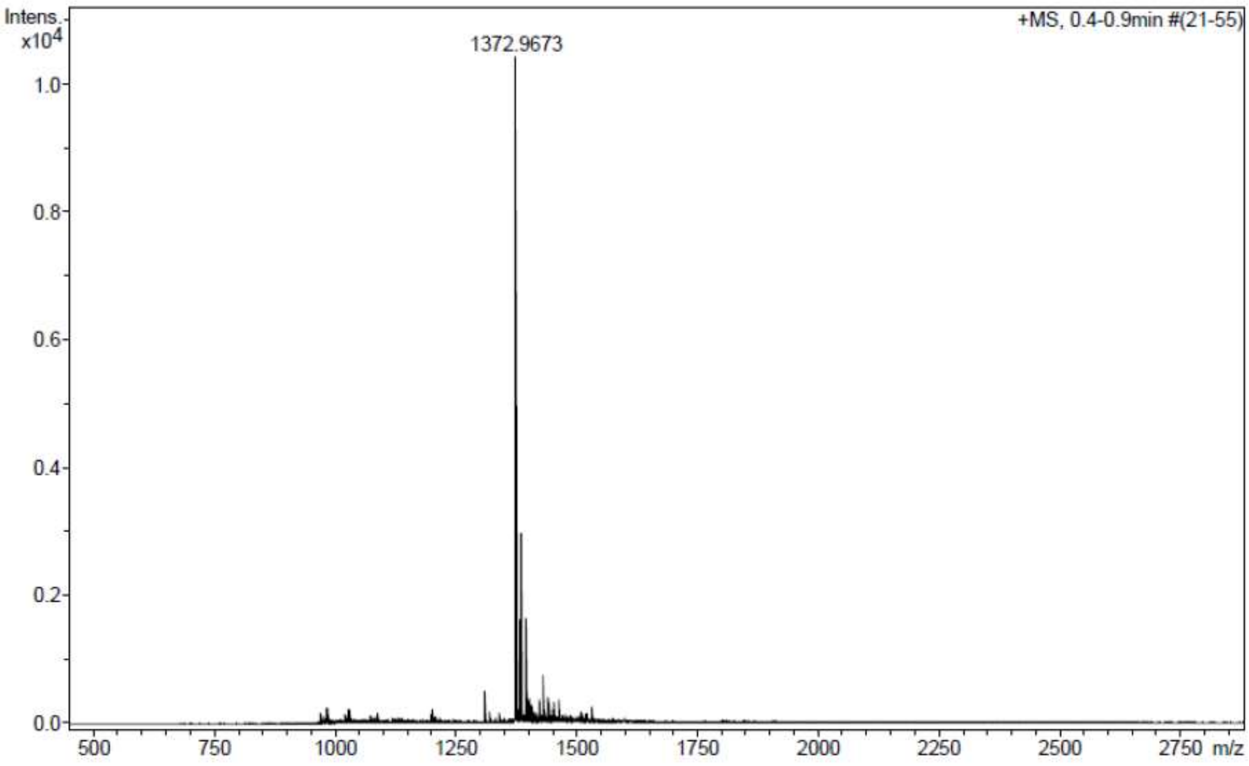

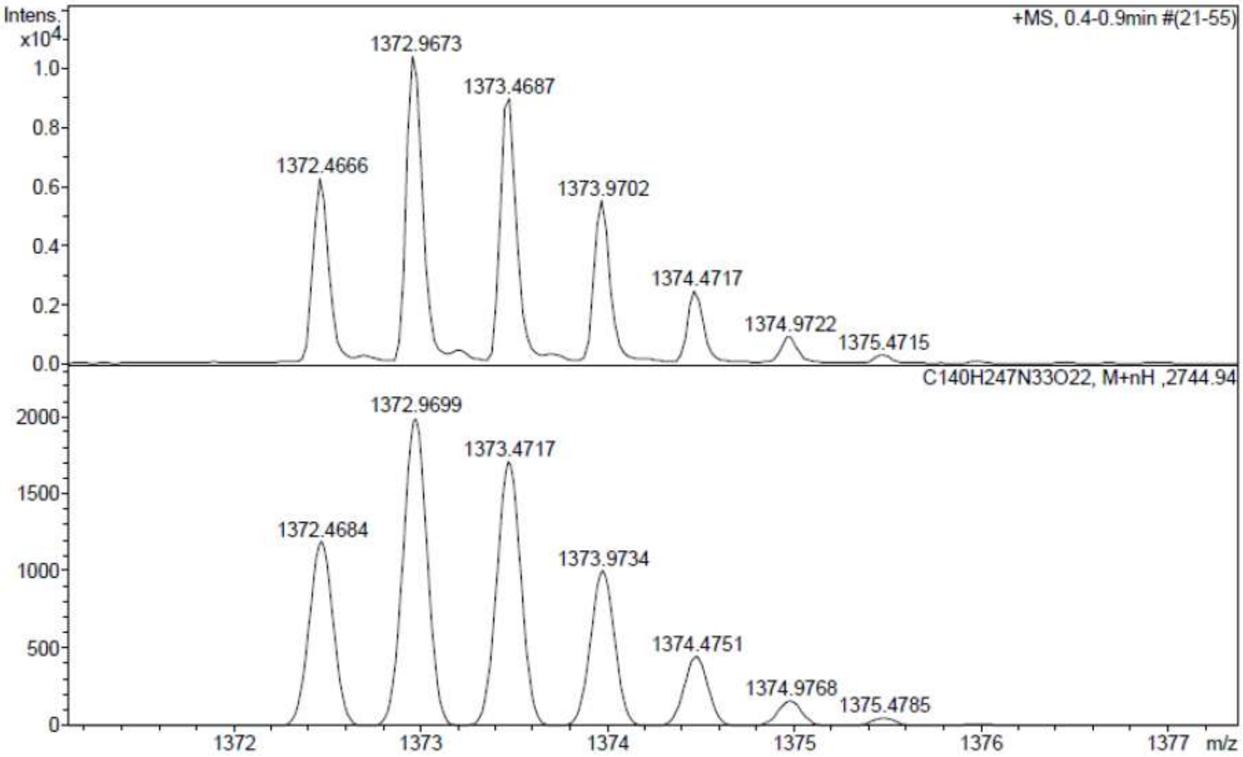
Intens.
x10^4
+MS, 0.4-0.9min #(21-55)
1372.9673
1373.4687
1372.4666
1373.9702
1374.4717
1374.9722
1375.4715
C140H247N33O22, M+nH ,2744.94
1372.9699
1373.4717
1372.4684
1373.9734
1374.4751
1374.9768
1375.4785
m/z

Lys-Lys-Leu-Phe-LysLys-Ile-Leu-Lys-Lys-Leu-Phe-Lys-Leu-Phe-Lys-Lys-Ile-Leu-Lys-Val-Leu-NH_2 (BP16-BP13)

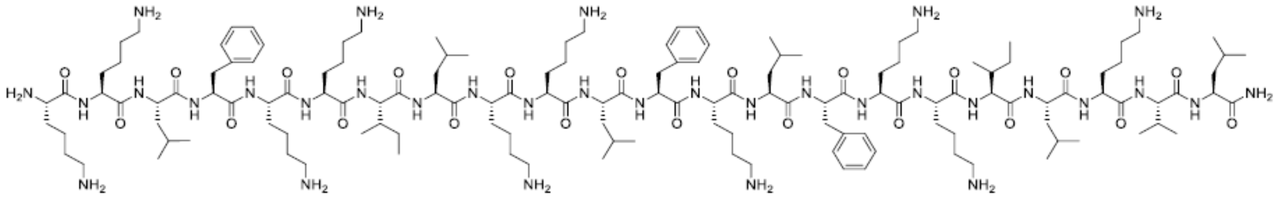

HPLC (λ=220 nm)

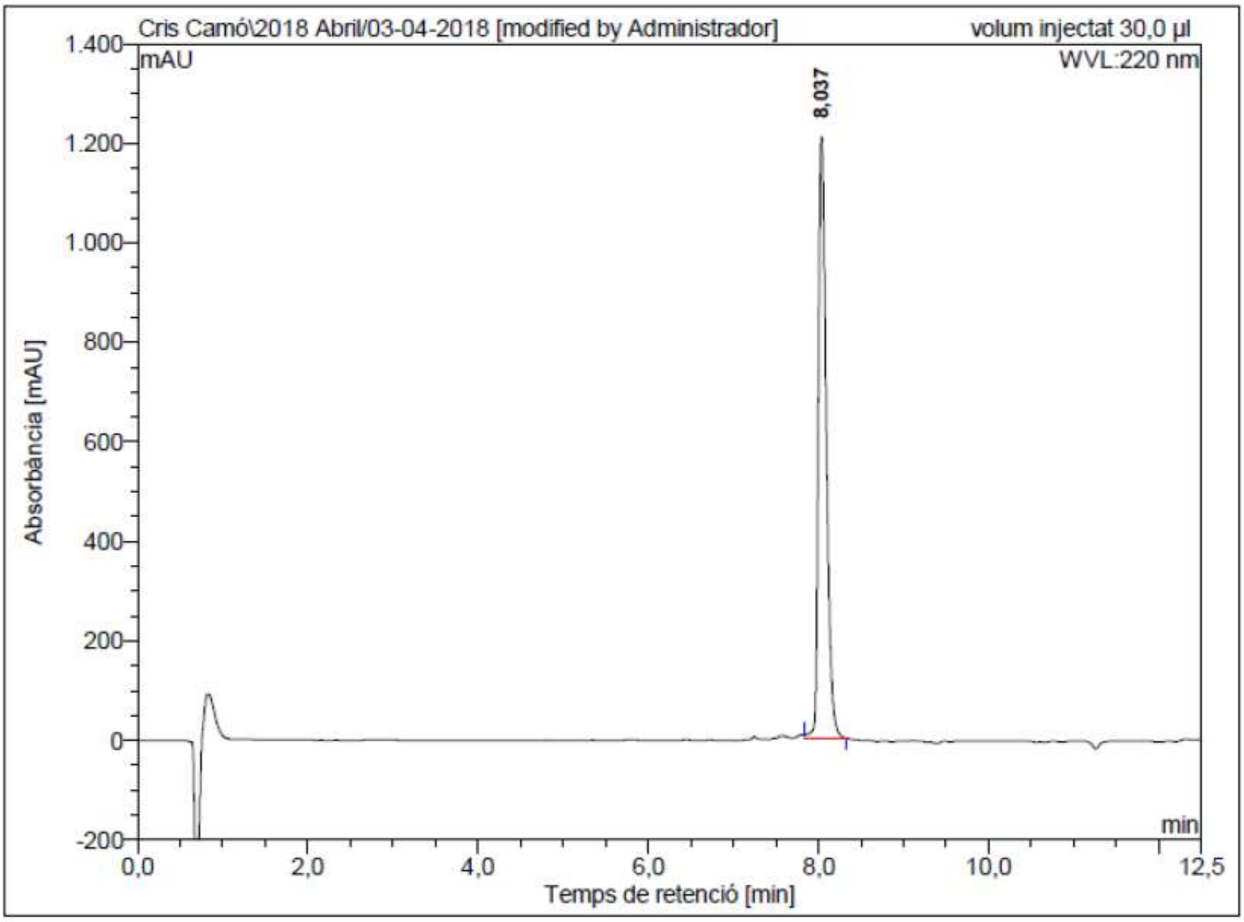

No.	Temps retenció min	alçada mAU	Area mAU*min	Area relativa %
1	8,04	1208,599	130,156	100,00
Total:		1208,599	130,156	100,00

ESI-MS (*m/z*)

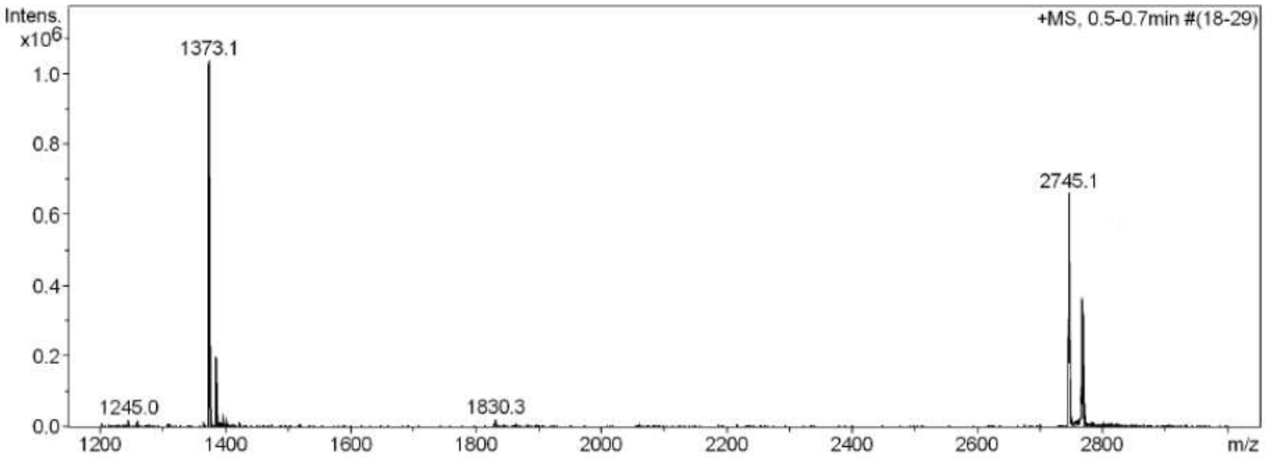

HRMS (*m/z*)

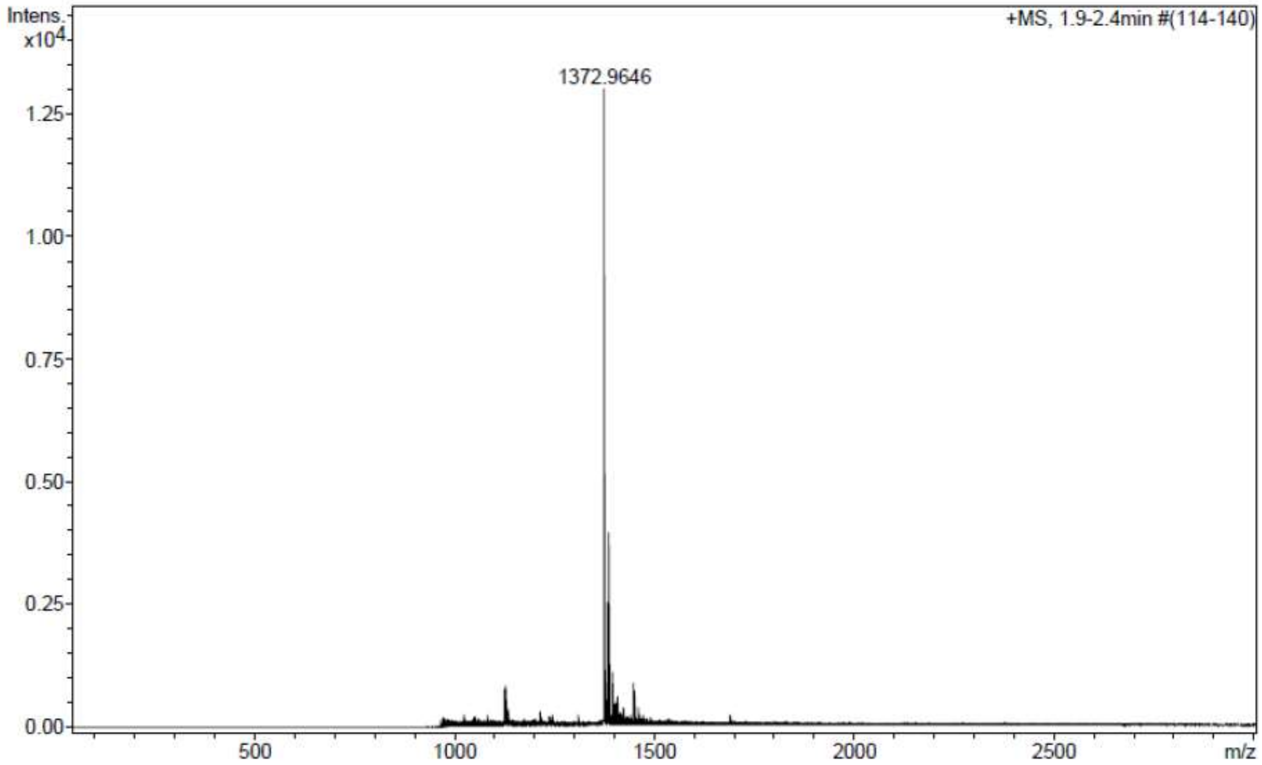

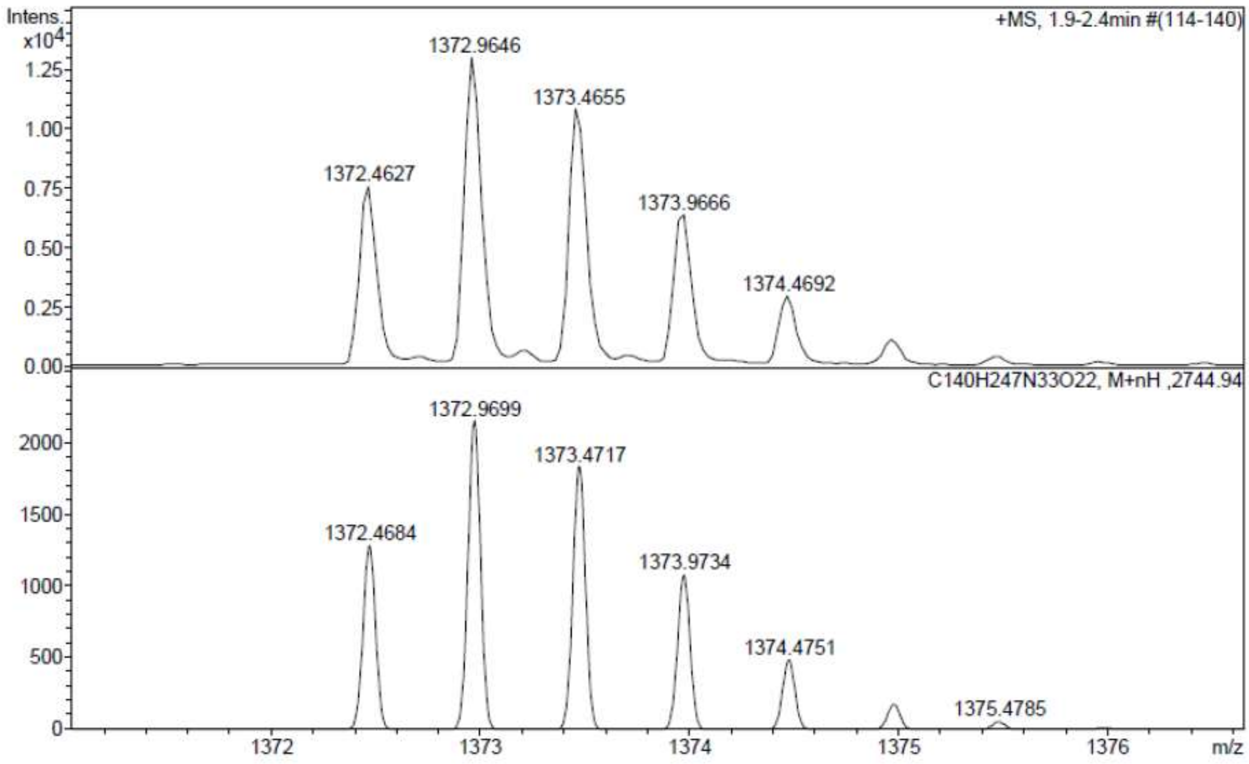
Intens.
x10^4
+MS, 1.9-2.4min #(114-140)
1372.9646
1373.4655
1372.4627
1373.9666
1374.4692
C140H247N33O22, M+nH ,2744.94
1372.9699
1373.4717
1372.4684
1373.9734
1374.4751
1375.4785
m/z

Phe-Lys-Leu-Phe-Lys-Lys-Ile-Leu-Lys-Val-Leu-Lys-Lys-Leu-Phe-Lys-Lys-Ile-Leu-Lys-Tyr-Leu-NH_2 (BP13- BP100)

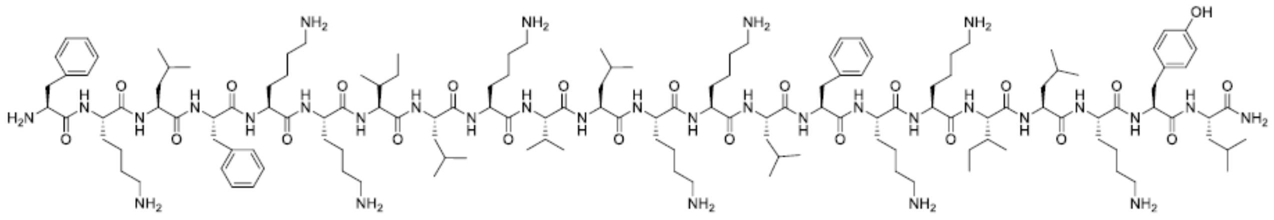

HPLC (λ=220 nm)

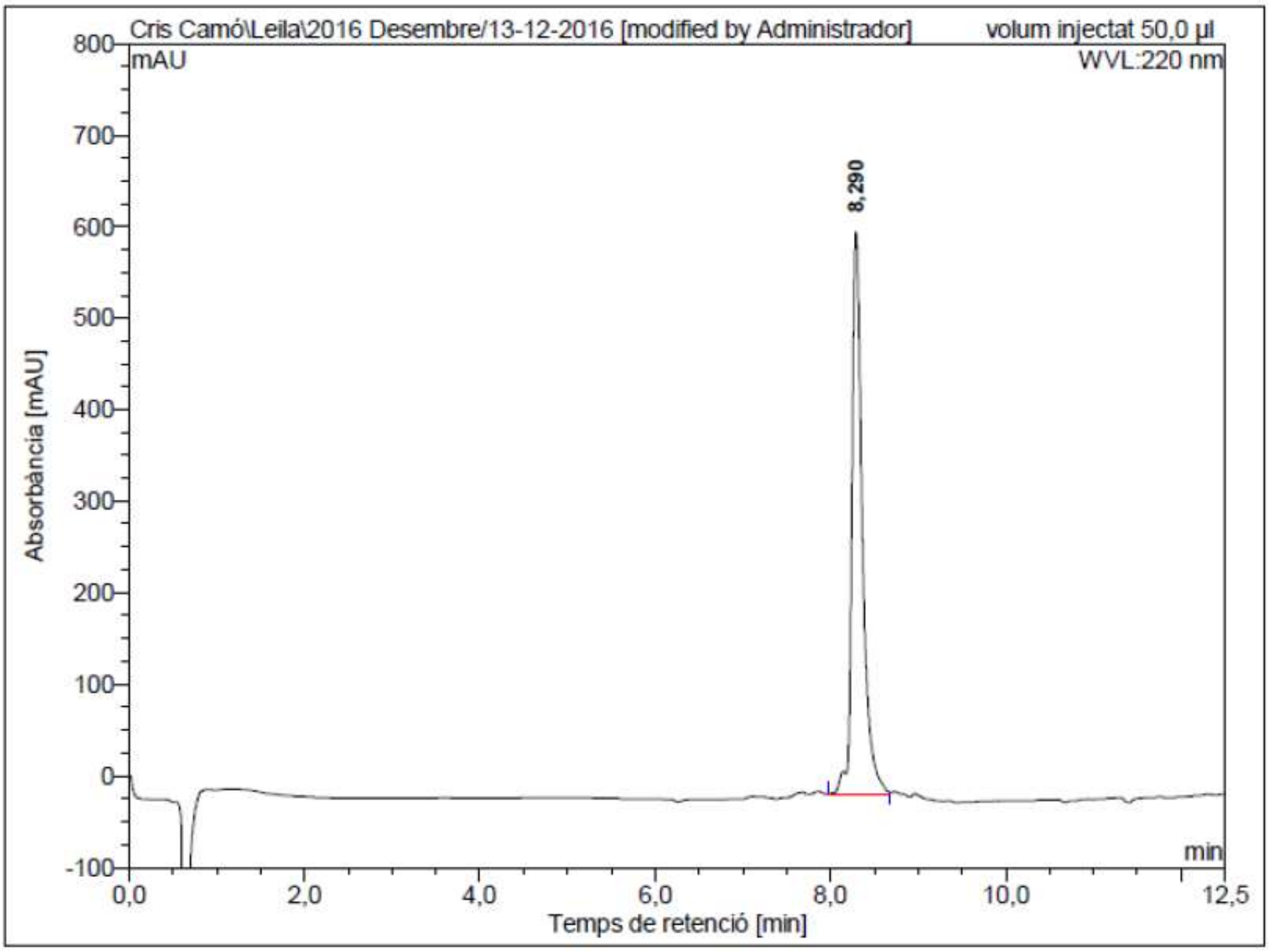

No.	Temps retenció min	alçada mAU	Area mAU*min	Area relativa %
1	8,29	615,017	85,815	100,00
Total:		615,017	85,815	100,00

ESI-MS (*m/z*)

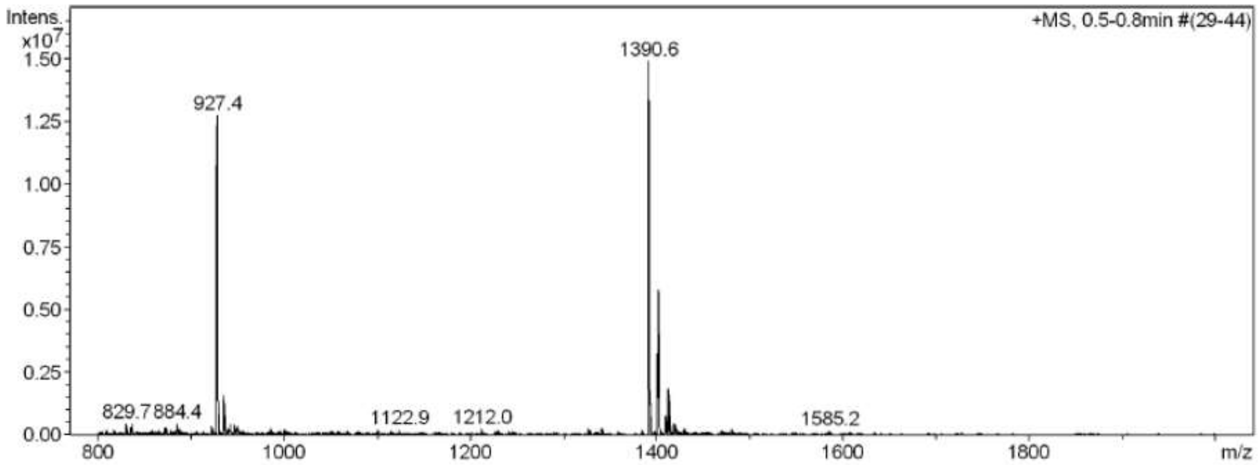

HRMS (*m/z*)

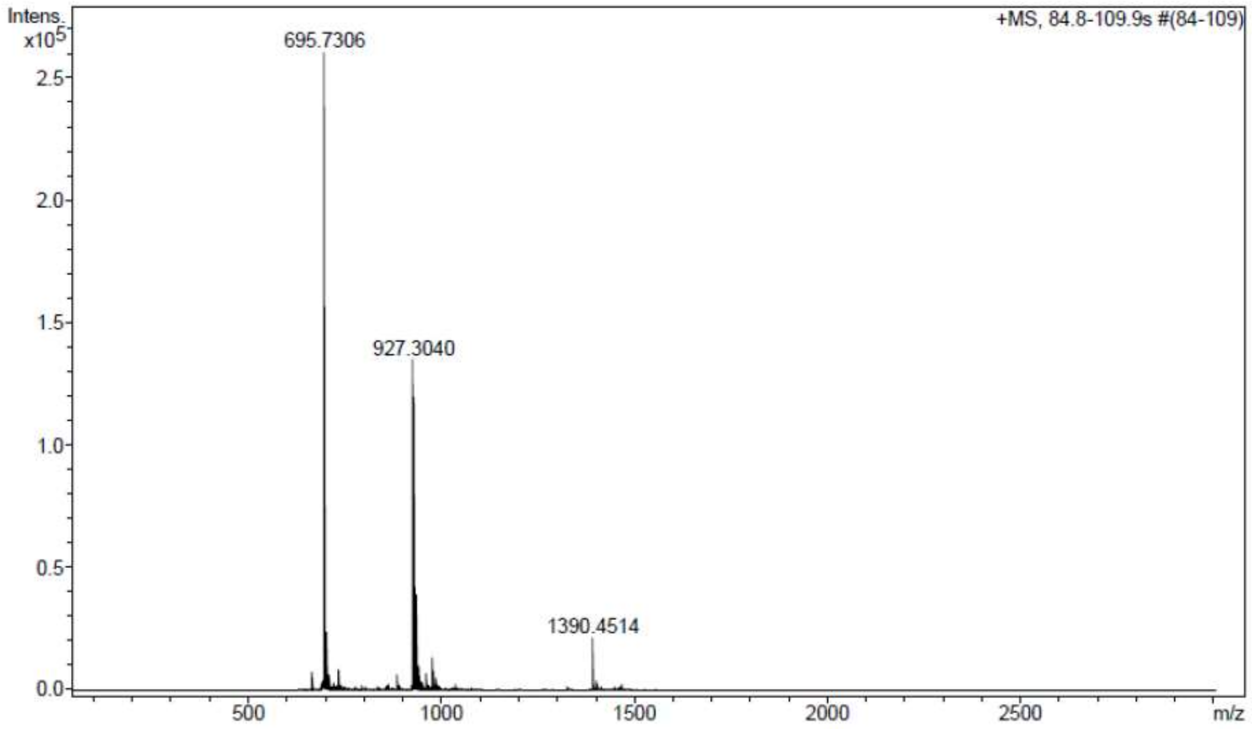

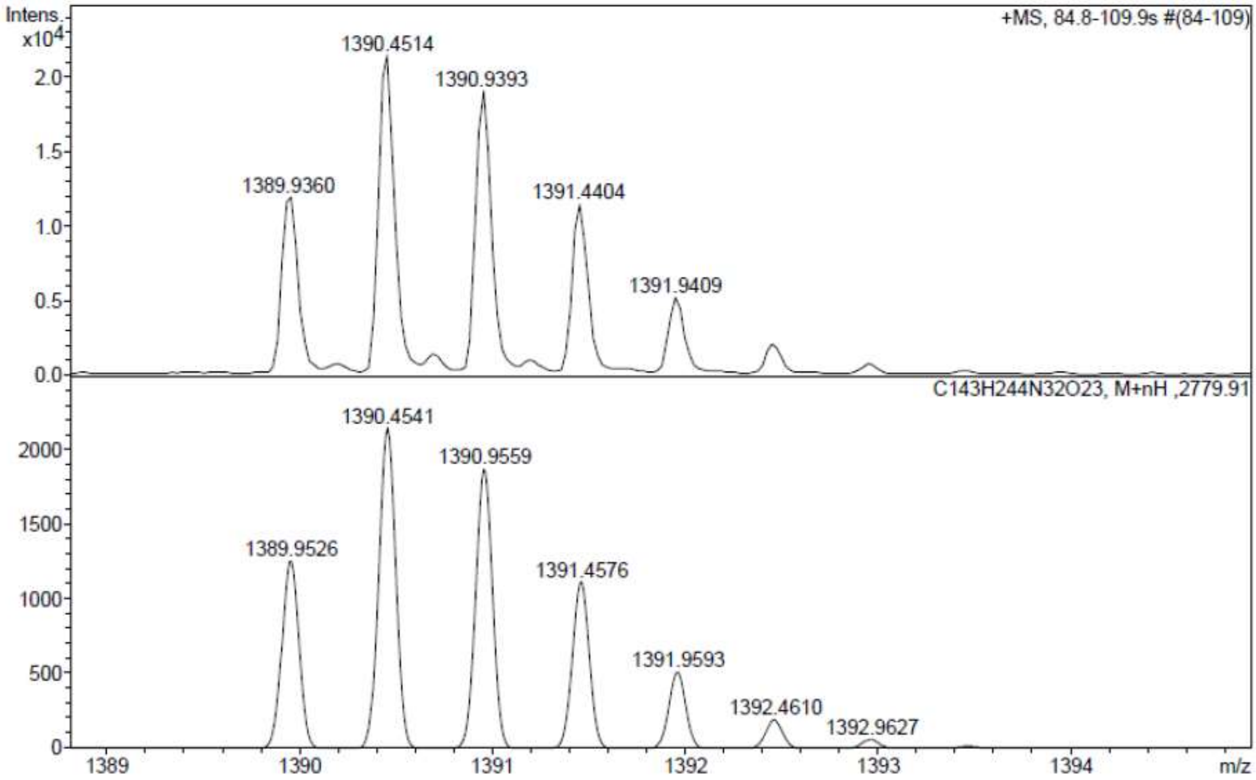
Intens. x10^4
+MS, 84.8-109.9s #(84-109)
1389.9360
1390.4514
1390.9393
1391.4404
1391.9409
C143H244N32O23, M+nH ,2779.91
1389.9526
1390.4541
1390.9559
1391.4576
1391.9593
1392.4610
1392.9627
m/z

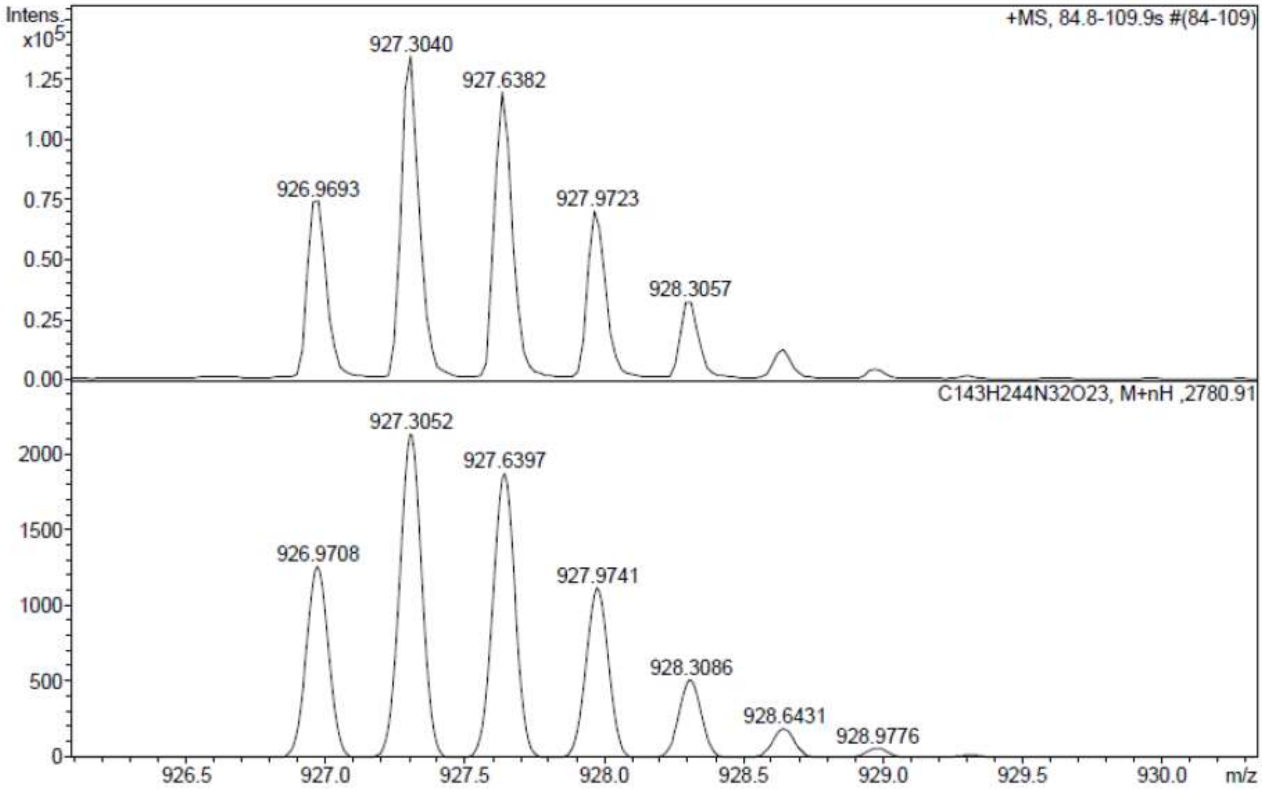
Intens. x10^5
+MS, 84.8-109.9s #(84-109)
926.9693
927.3040
927.6382
927.9723
928.3057
C143H244N32O23, M+nH ,2780.91
926.9708
927.3052
927.6397
927.9741
928.3086
928.6431
928.9776
m/z

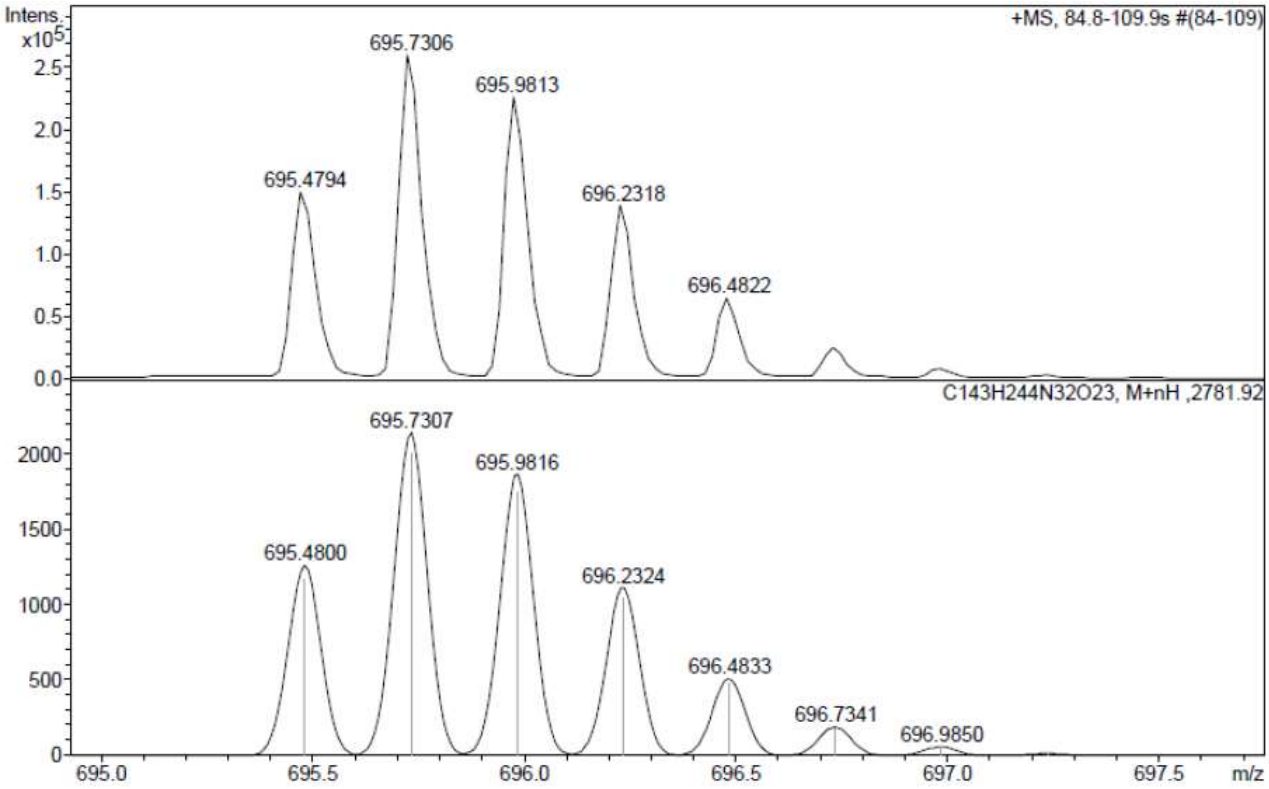
Intens.
x10^5
+MS, 84.8-109.9s #(84-109)
695.7306
695.9813
695.4794
696.2318
696.4822
C143H244N32O23, M+nH ,2781.92
695.7307
695.9816
695.4800
696.2324
696.4833
696.7341
696.9850
m/z

Lys-Lys-Leu-Phe-Lys-Lys-Ile-Leu-Lys-Tyr-Leu-Phe-Lys-Leu-Phe-Lys-Lys-Ile-Leu-Lys-Val-Leu-NH_2 (BP100-BP13)

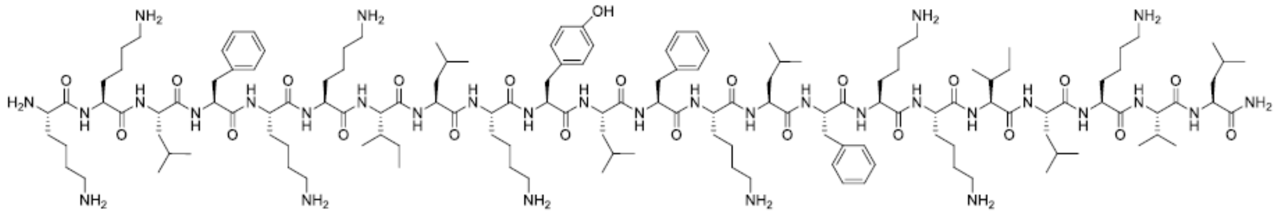

HPLC (λ=220 nm)

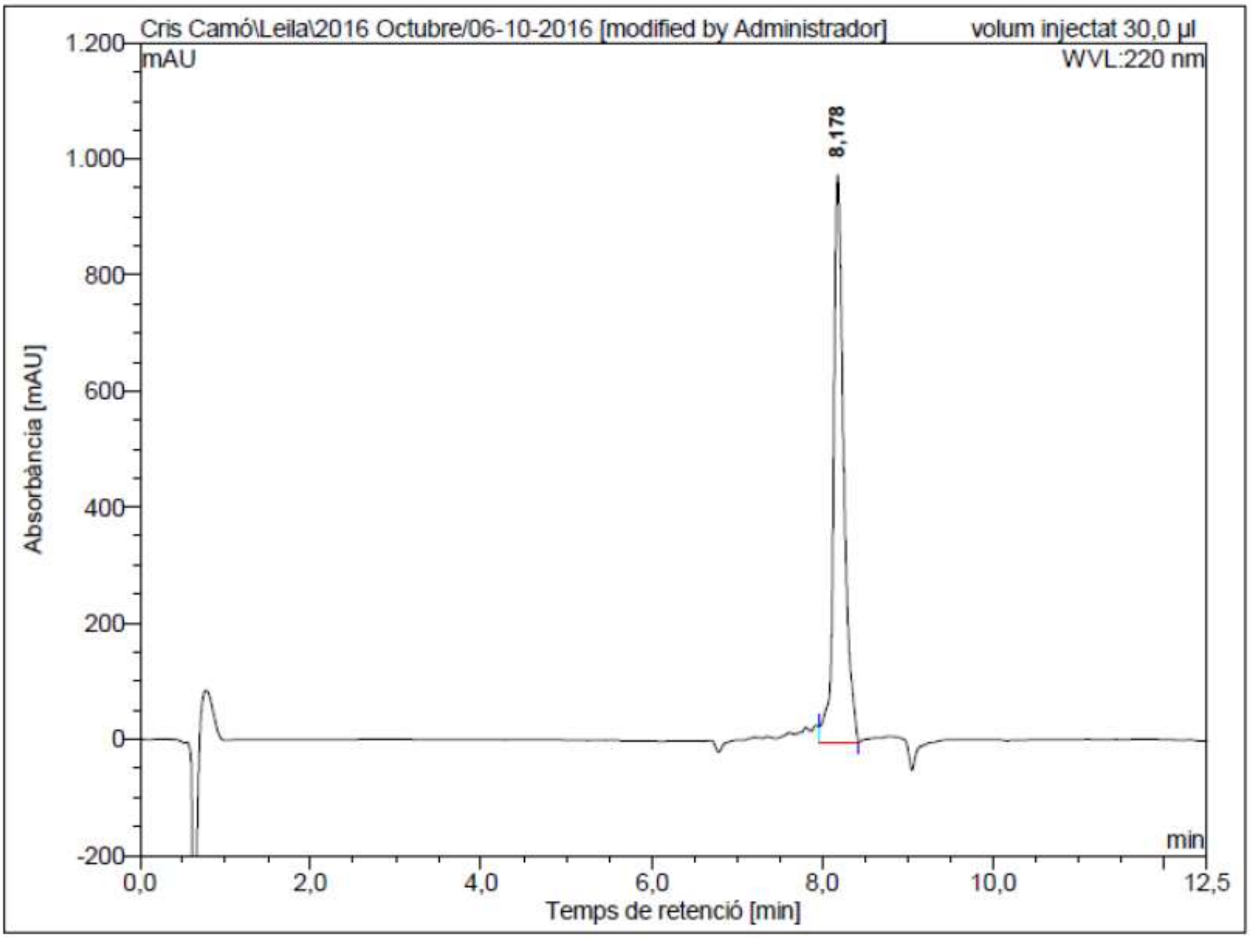

No.	Temps retenció min	alçada mAU	Area mAU*min	Area relativa %
1	8,18	976,959	135,414	100,00
Total:		976,959	135,414	100,00

ESI-MS (*m/z*)

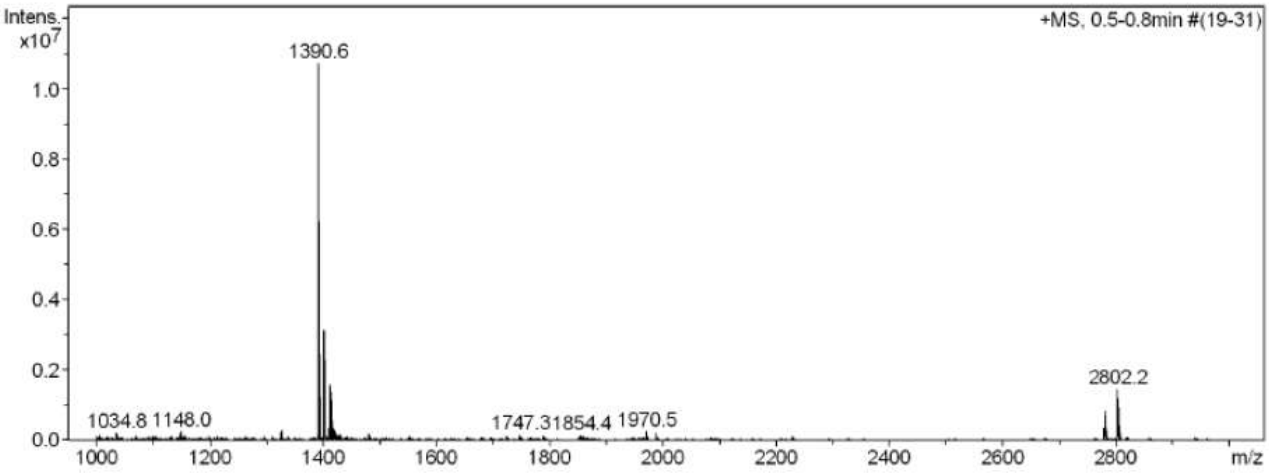

HRMS (*m/z*)

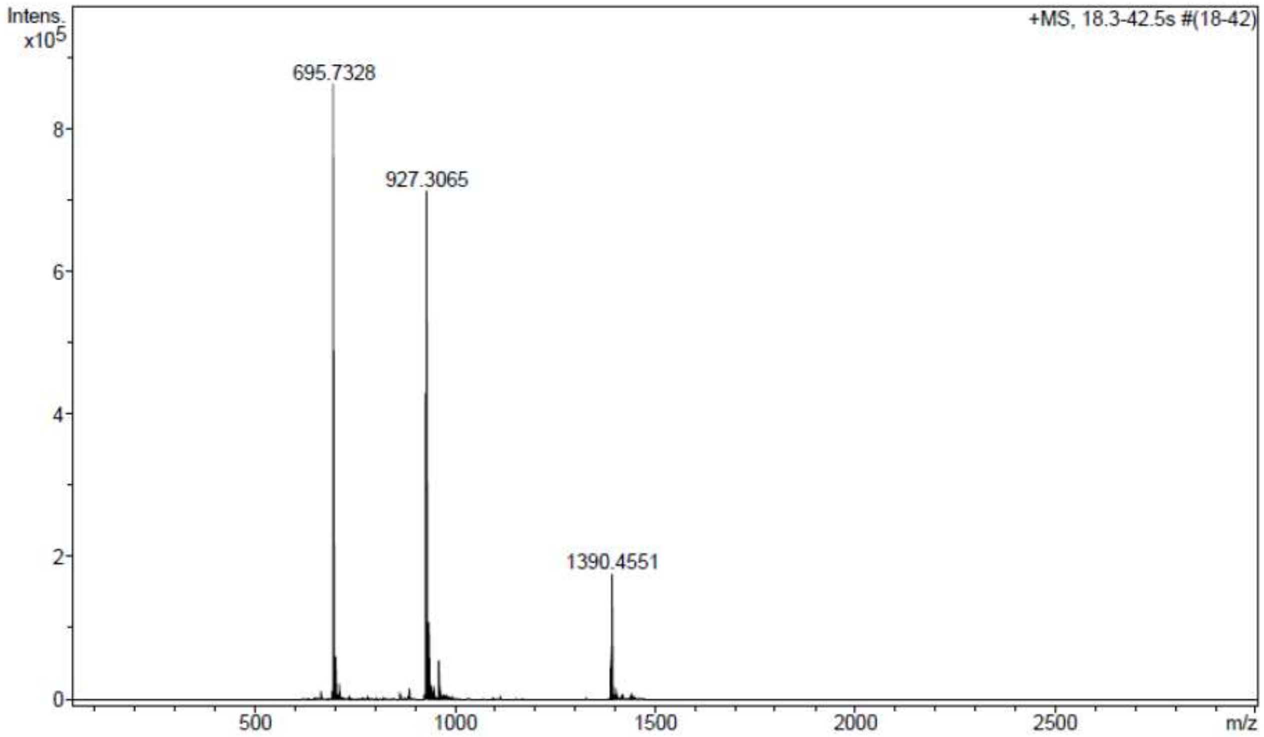

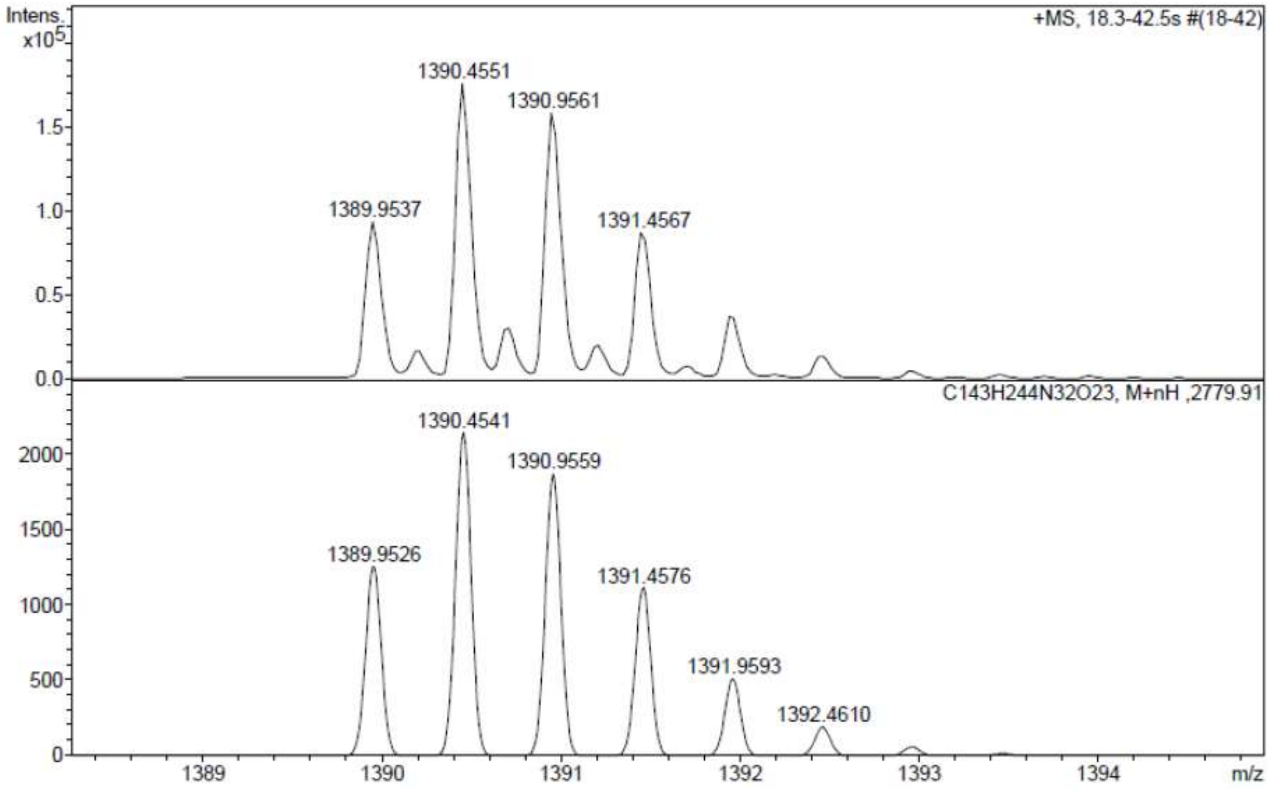

Intens.
x10^5
+MS, 18.3-42.5s #(18-42)
1390.4551
1390.9561
1389.9537
1391.4567
C143H244N32O23, M+nH ,2779.91
1390.4541
1390.9559
1389.9526
1391.4576
1391.9593
1392.4610
m/z

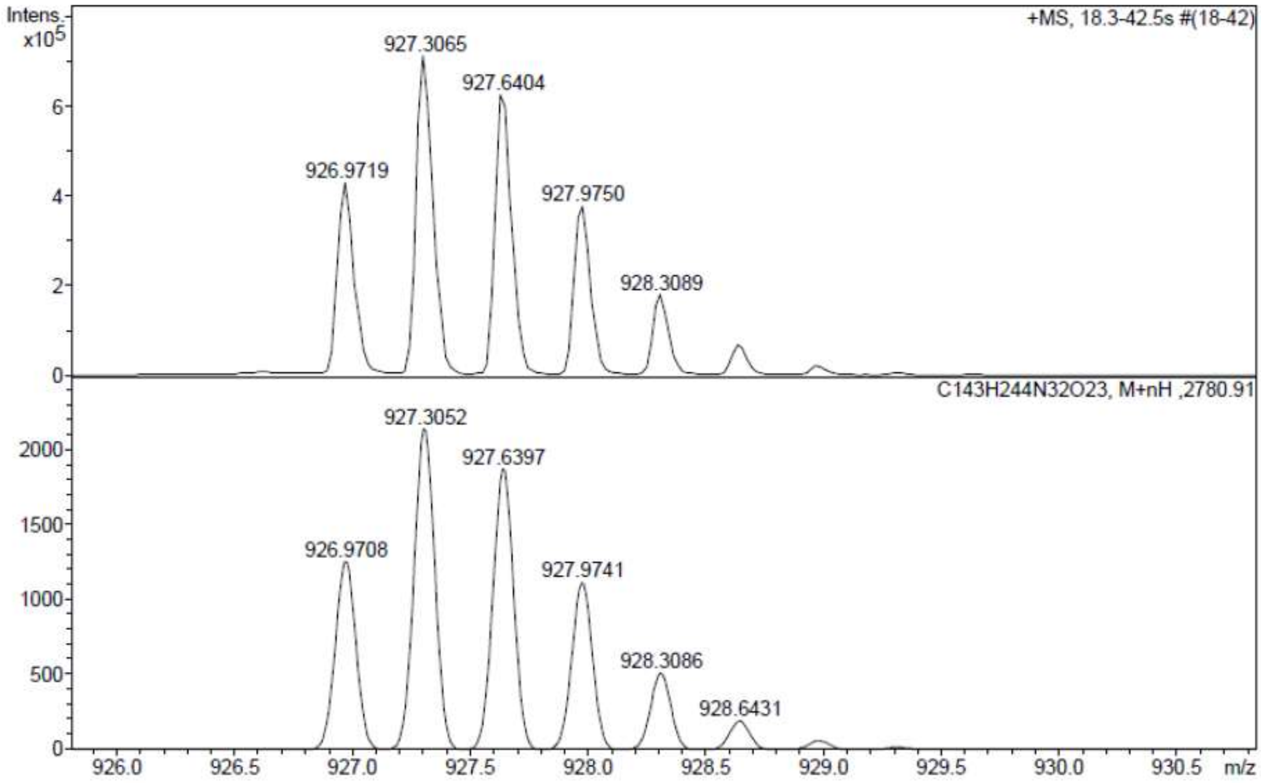

Intens.
x10^5
+MS, 18.3-42.5s #(18-42)
927.3065
927.6404
926.9719
927.9750
928.3089
C143H244N32O23, M+nH ,2780.91
927.3052
927.6397
926.9708
927.9741
928.3086
928.6431
m/z

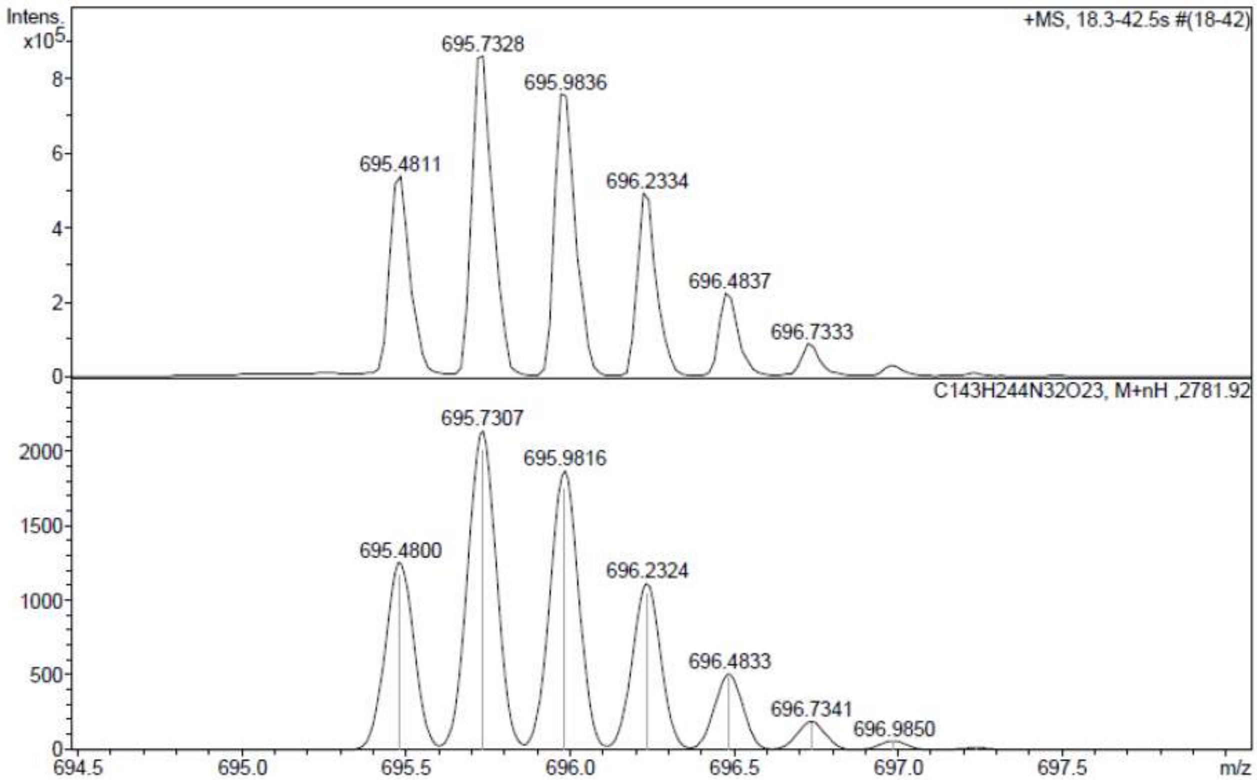
Intens.
x10^5
+MS, 18.3-42.5s #(18-42)
695.7328
695.9836
695.4811
696.2334
696.4837
696.7333
C143H244N32O23, M+nH ,2781.92
695.7307
695.9816
695.4800
696.2324
696.4833
696.7341
696.9850
m/z

Phe-Lys-Leu-Phe-Lys-Lys-Ile-Leu-Lys-Val-Leu-Lys-Lys-Leu-D-Phe-Lys-Lys-Ile-Leu-Lys-Tyr-Leu-NH_2 (BP13-BP143)

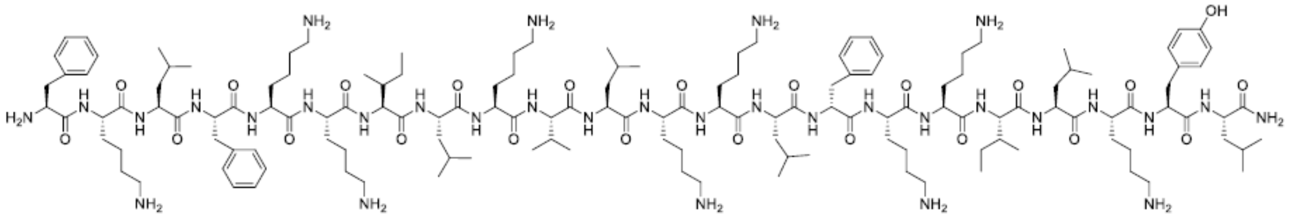

HPLC (λ=220 nm)

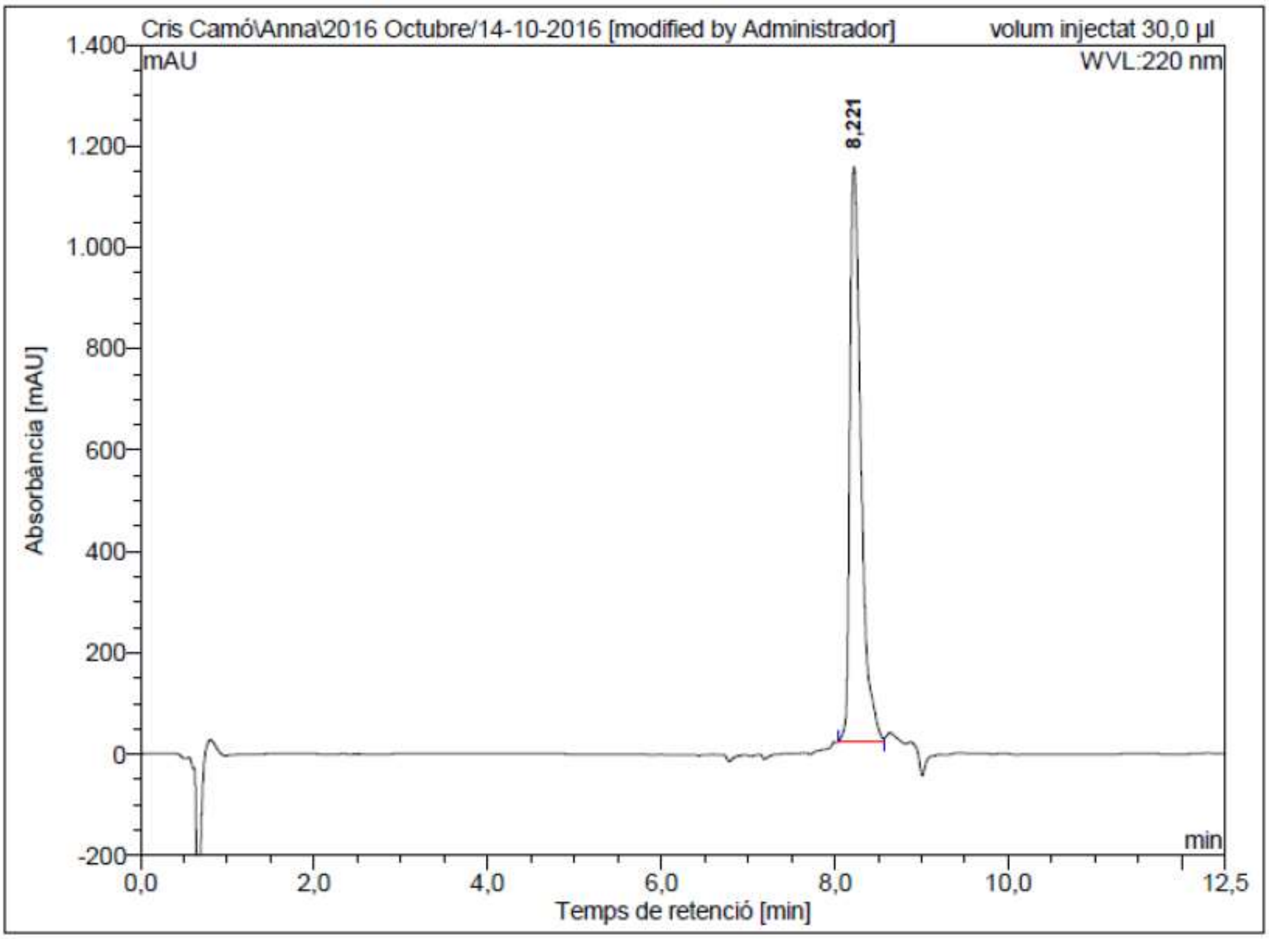

No.	Temps retenció<br>min	alçada<br>mAU	Area<br>mAU*min	Area relativa<br>%
1	8,22	1134,712	169,763	100,00
Total:		1134,712	169,763	100,00

ESI-MS (*m/z*)

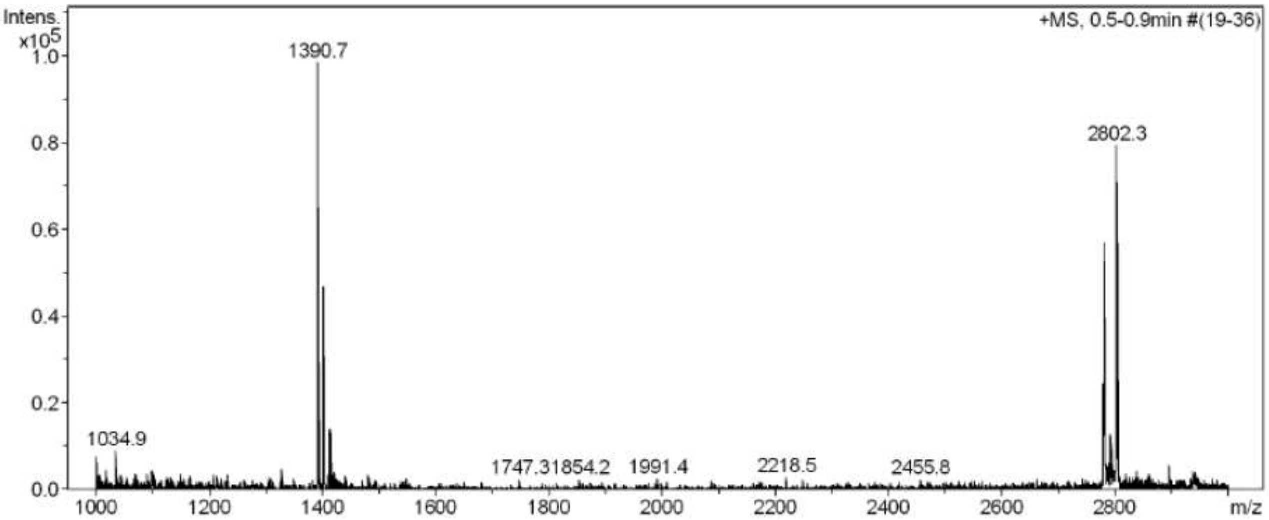

HRMS (*m/z*)

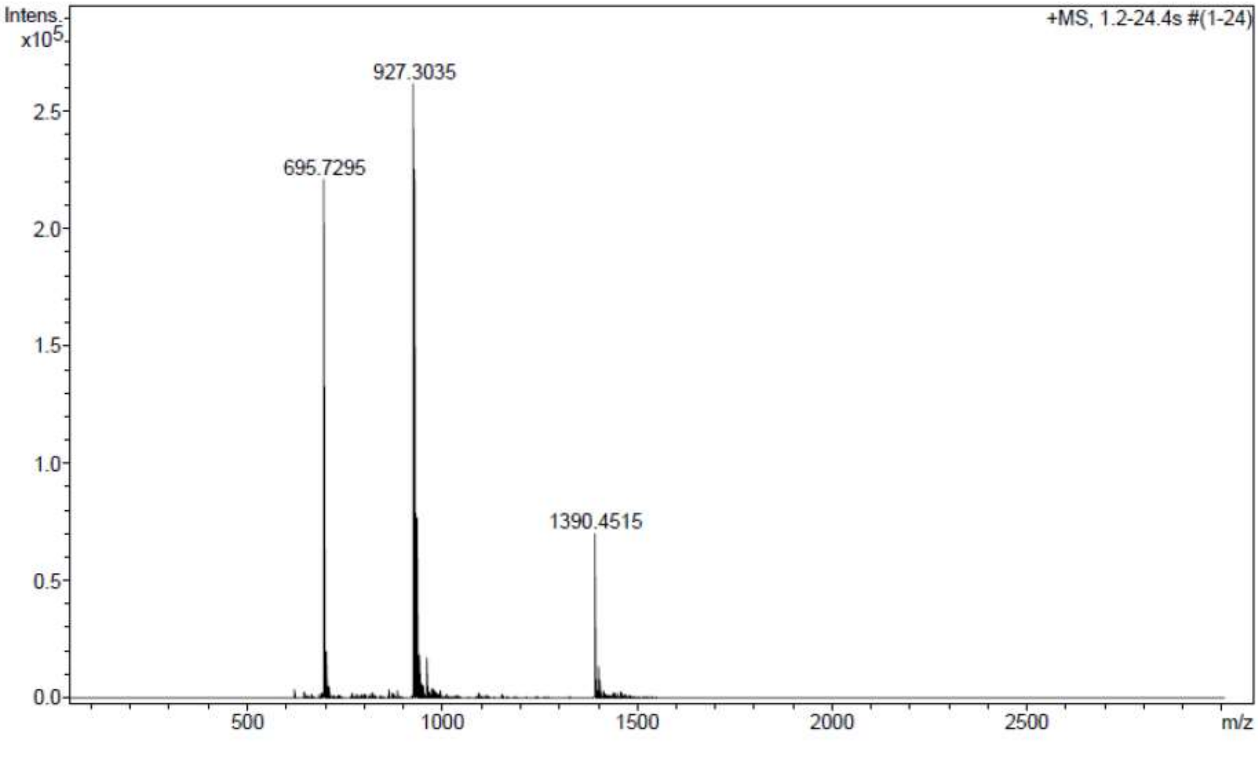

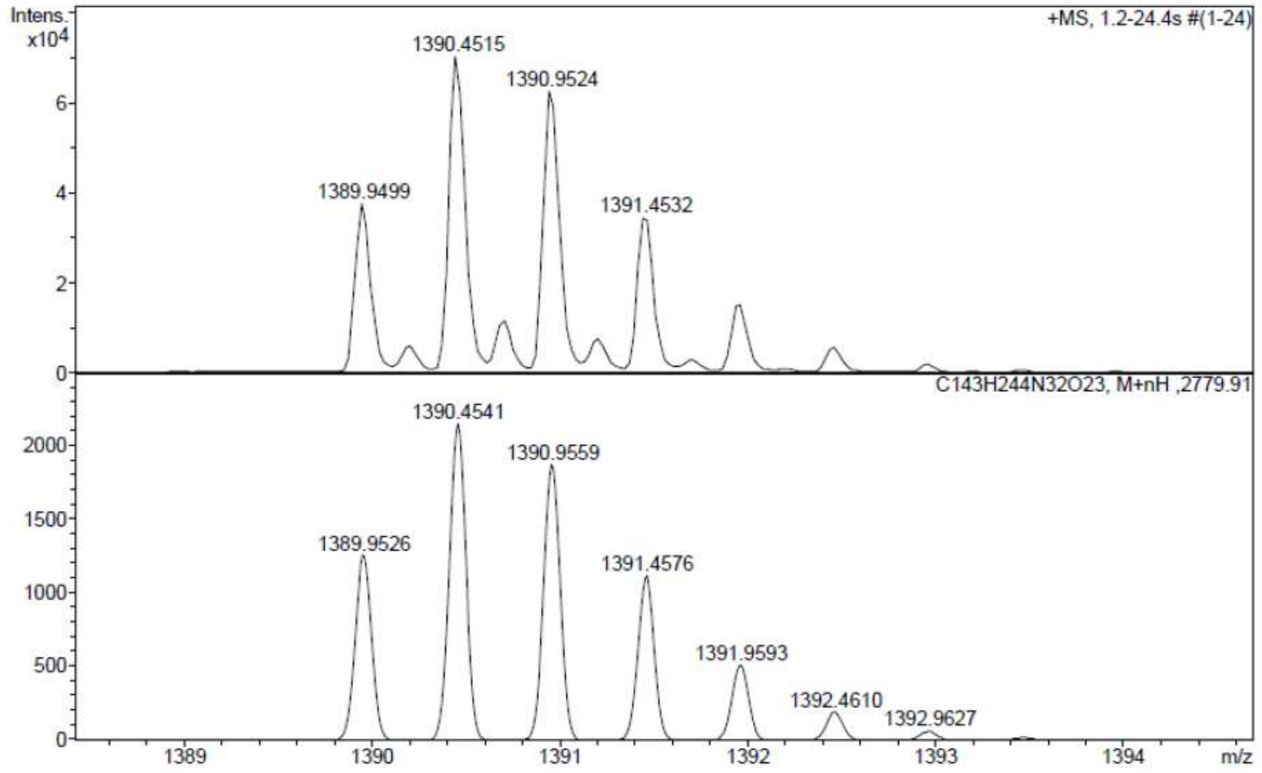
Intens.
x10^4
+MS, 1.2-24.4s #(1-24)
1390.4515
1390.9524
1389.9499
1391.4532
C143H244N32O23, M+nH ,2779.91
1390.4541
1390.9559
1389.9526
1391.4576
1391.9593
1392.4610
1392.9627
m/z

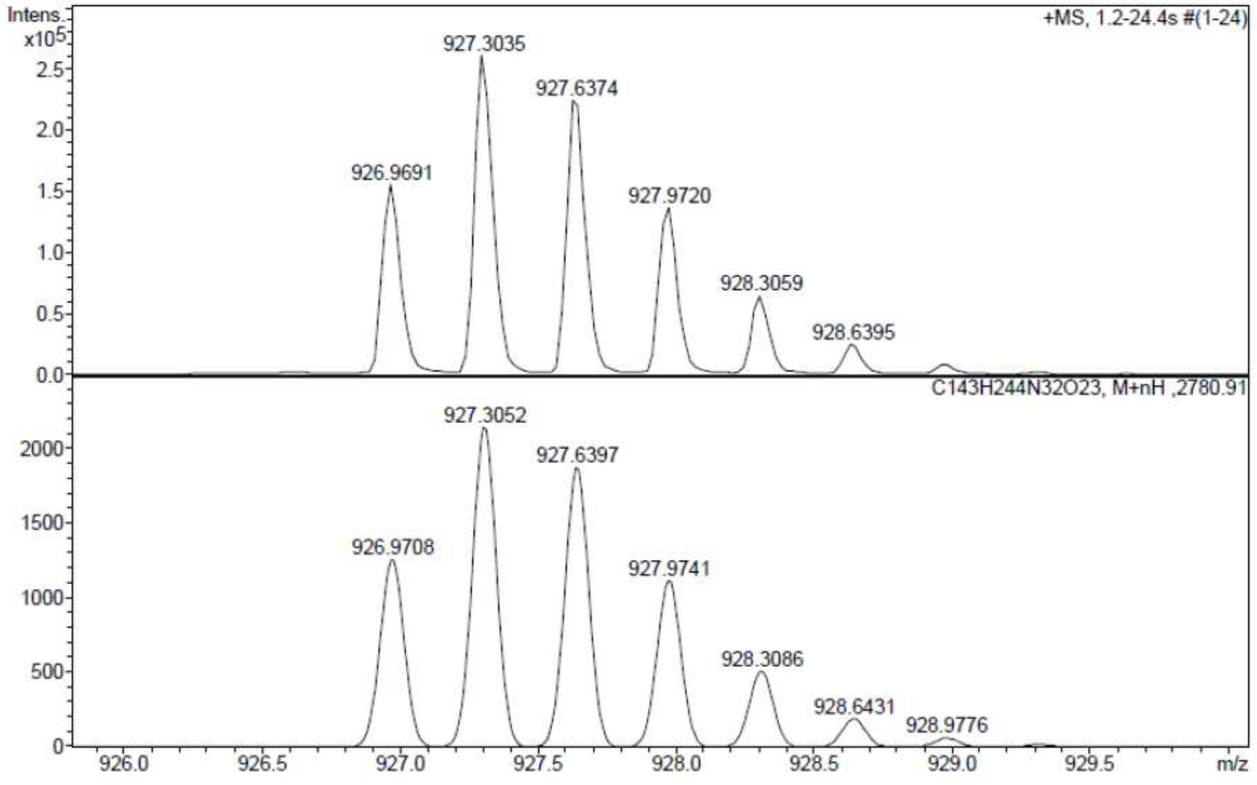
Intens.
x10^5
+MS, 1.2-24.4s #(1-24)
927.3035
927.6374
926.9691
927.9720
928.3059
928.6395
C143H244N32O23, M+nH ,2780.91
927.3052
927.6397
926.9708
927.9741
928.3086
928.6431
928.9776
m/z

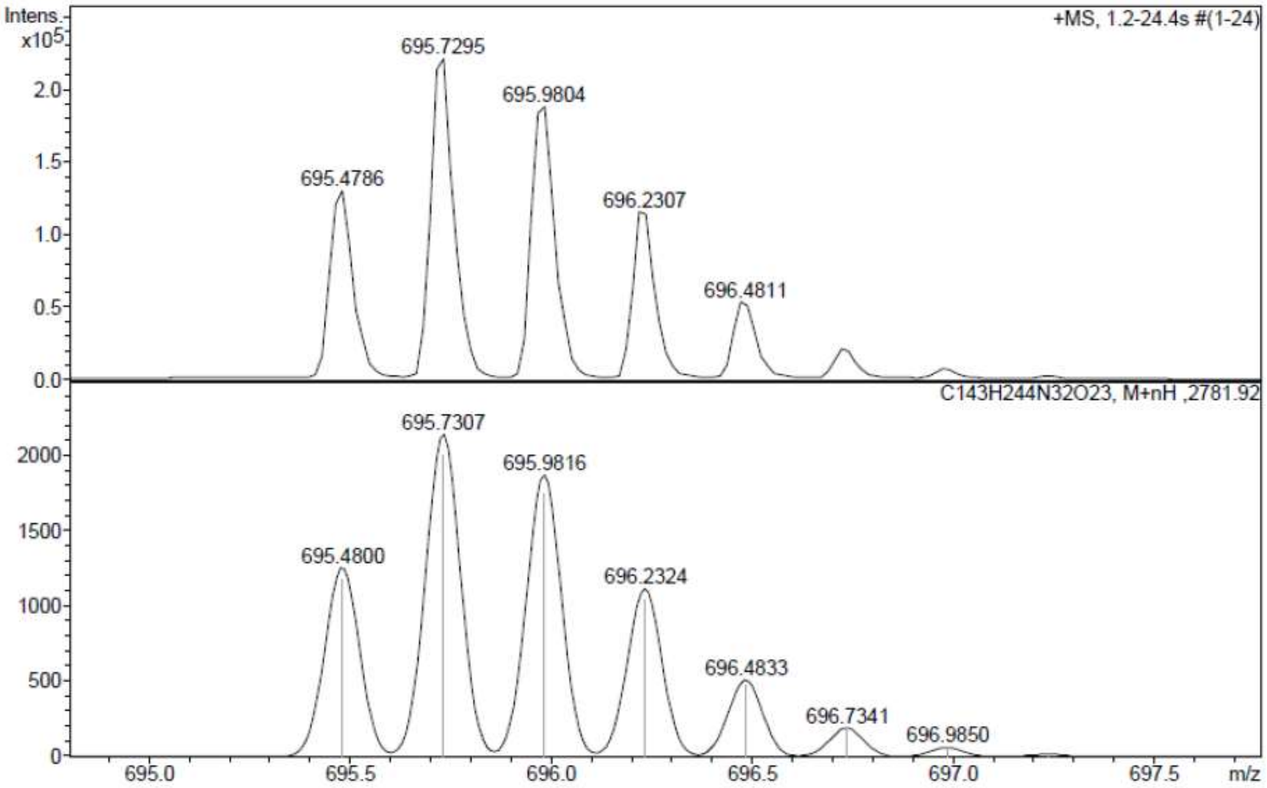
Intens.
x10^5
+MS, 1.2-24.4s #(1-24)
695.7295
695.9804
695.4786
696.2307
696.4811
C143H244N32O23, M+nH ,2781.92
695.7307
695.9816
695.4800
696.2324
696.4833
696.7341
696.9850
m/z

Lys-Lys-Leu-D-Phe-Lys-Lys-Ile-Leu-Lys-Tyr-Leu-Phe-Lys-Leu-Phe-Lys-Lys-Ile-Leu-Lys-Val-Leu-NH_2 (BP143-BP13)

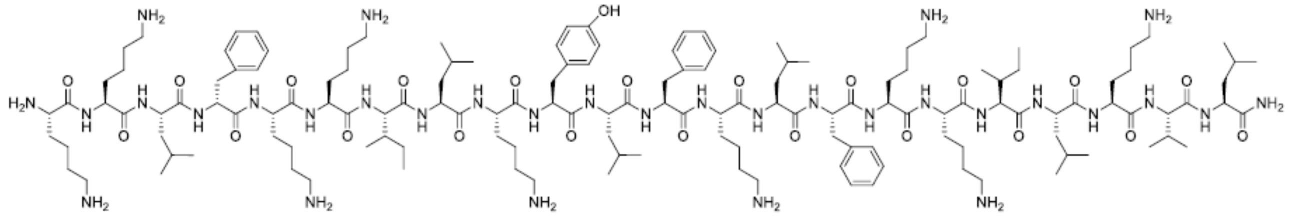

HPLC (λ=220 nm)

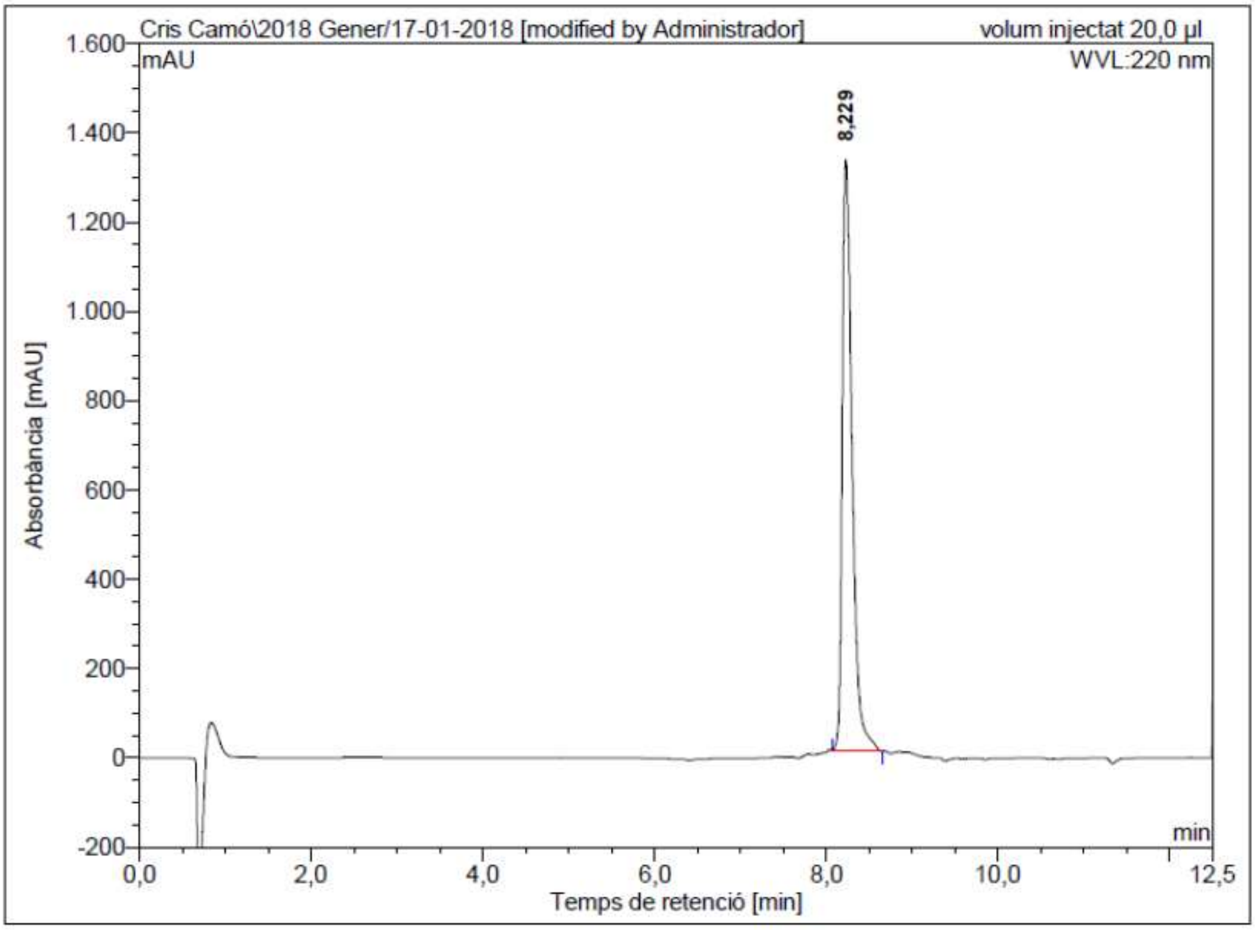

No.	Temps retenció min	alçada mAU	Area mAU*min	Area relativa %
1	8,23	1325,618	176,508	100,00
Total:		1325,618	176,508	100,00

ESI-MS (*m/z*)

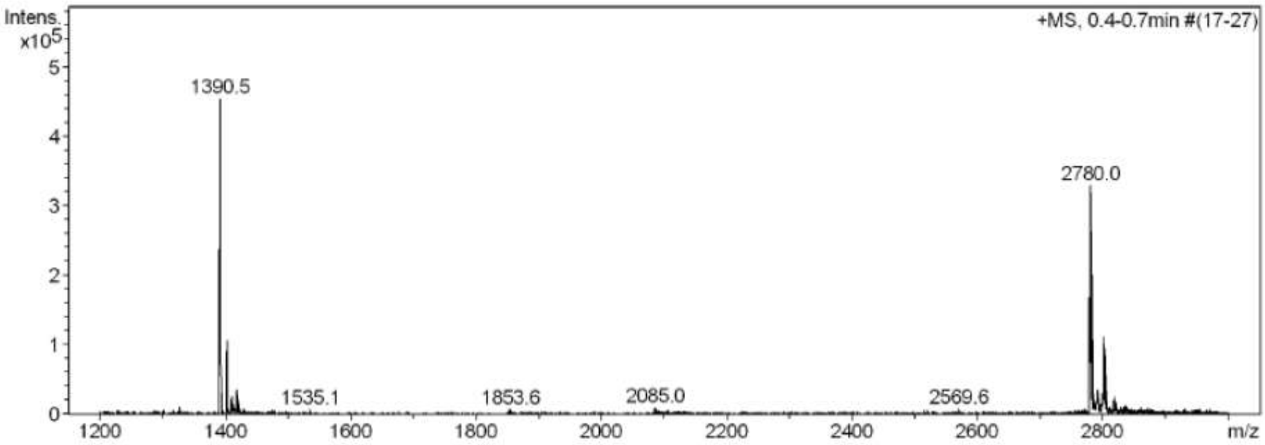

HRMS (*m/z*)

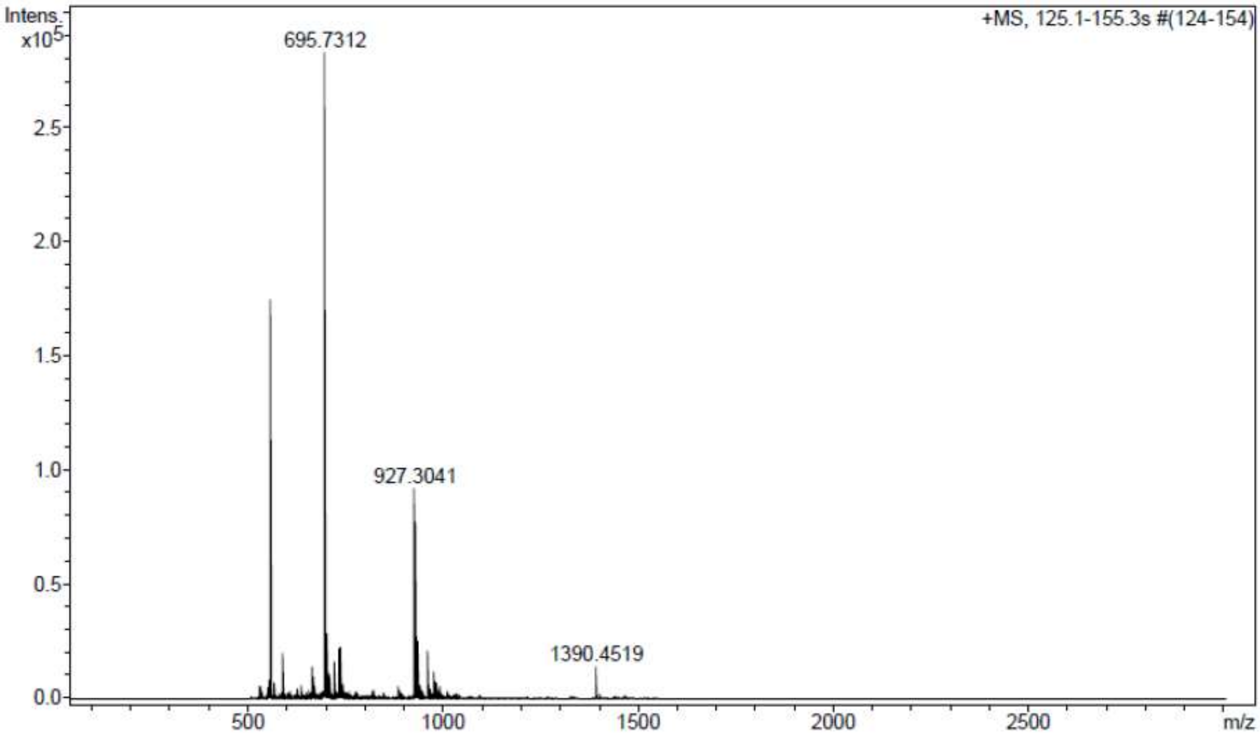

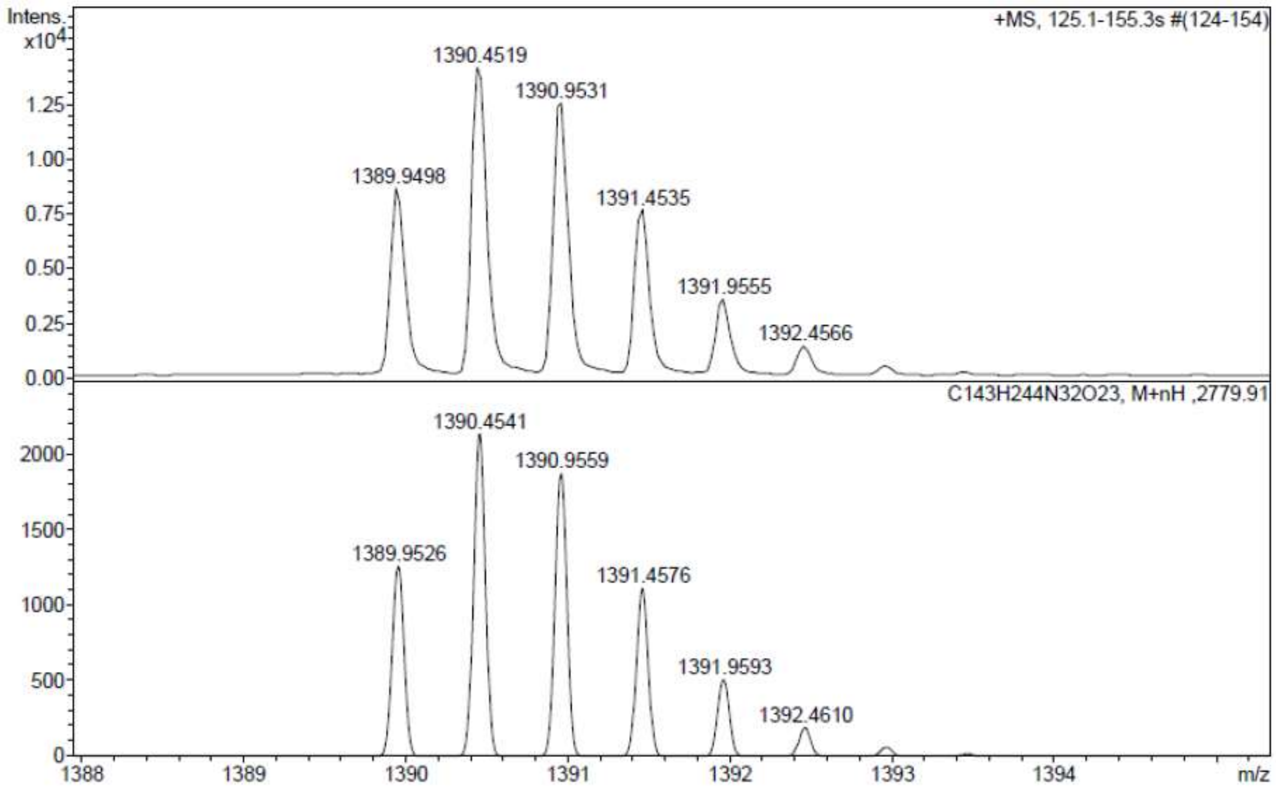
Intens.
x10^4
+MS, 125.1-155.3s #(124-154)
1390.4519
1390.9531
1389.9498
1391.4535
1391.9555
1392.4566
C143H244N32O23, M+nH ,2779.91
1390.4541
1390.9559
1389.9526
1391.4576
1391.9593
1392.4610
m/z

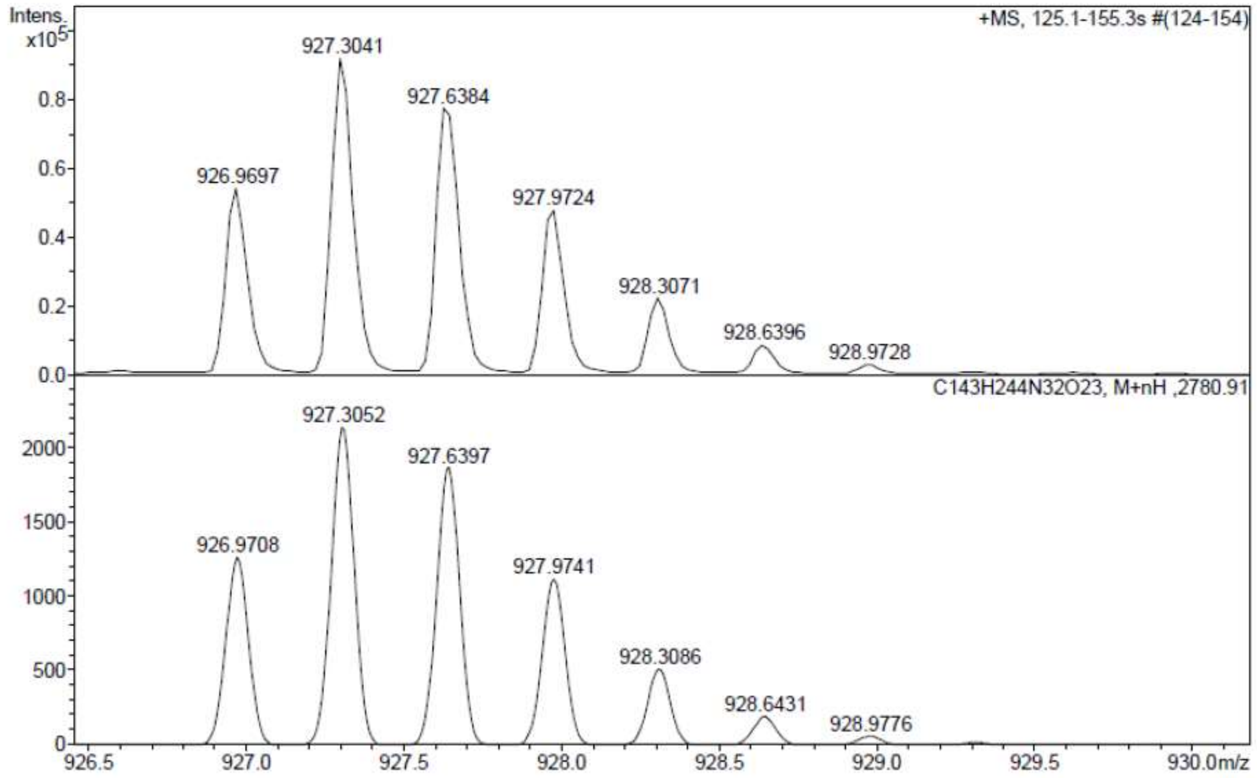
Intens.
x10^5
+MS, 125.1-155.3s #(124-154)
927.3041
927.6384
926.9697
927.9724
928.3071
928.6396
928.9728
C143H244N32O23, M+nH ,2780.91
927.3052
927.6397
926.9708
927.9741
928.3086
928.6431
928.9776
m/z

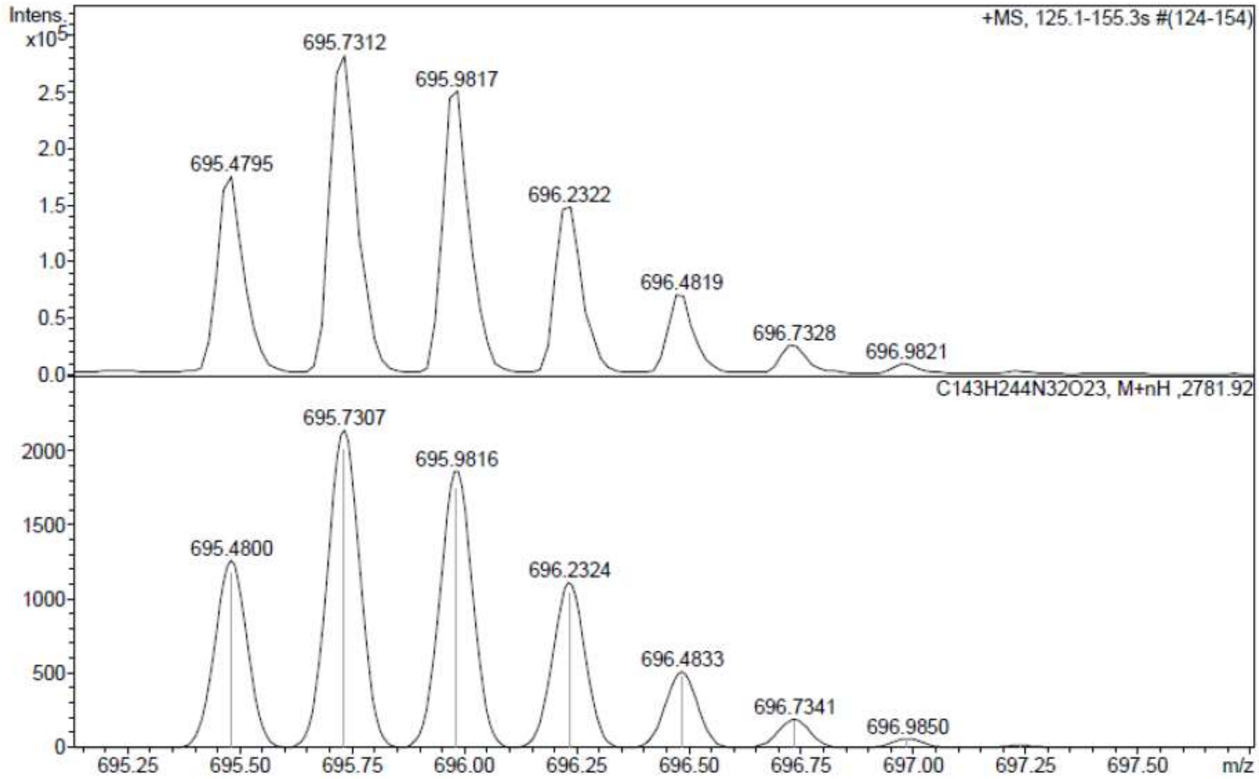
Intens.
x10^5
+MS, 125.1-155.3s #(124-154)
695.4795
695.7312
695.9817
696.2322
696.4819
696.7328
696.9821
C143H244N32O23, M+nH ,2781.92
695.4800
695.7307
695.9816
696.2324
696.4833
696.7341
696.9850
m/z

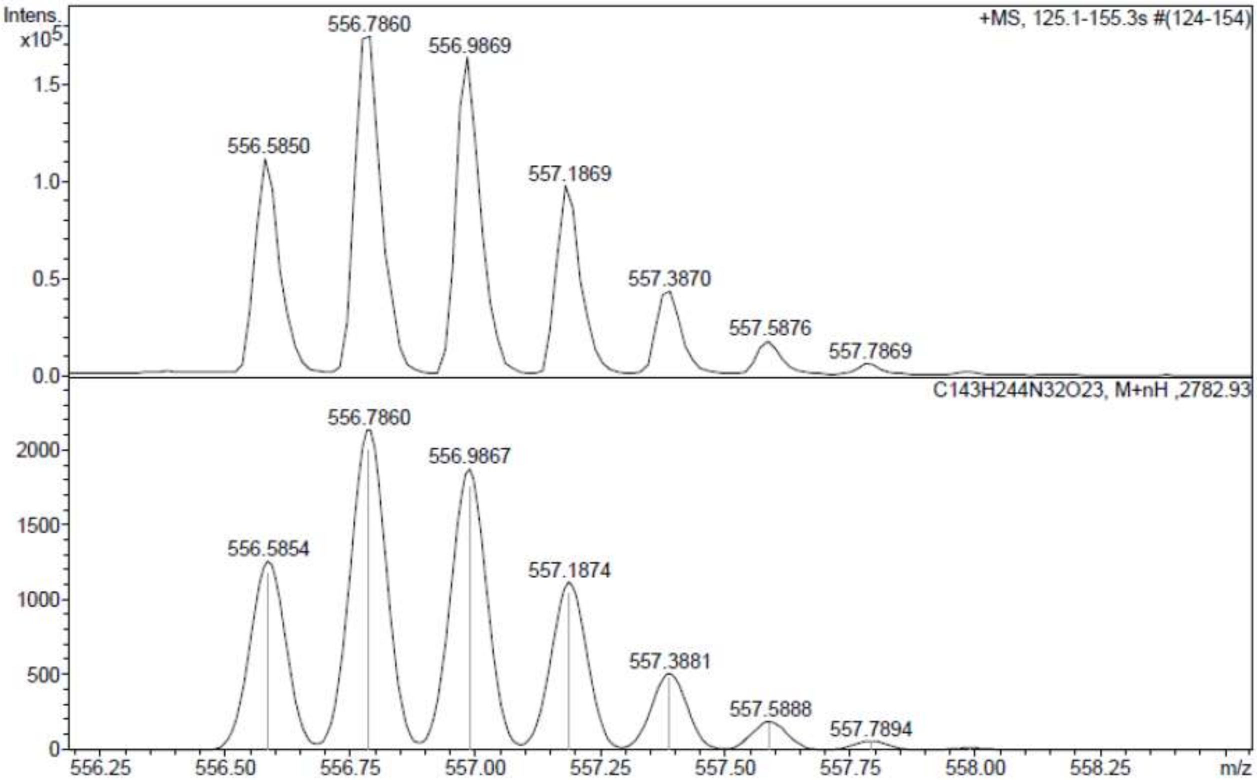
Intens.
x10^5
+MS, 125.1-155.3s #(124-154)
556.5850
556.7860
556.9869
557.1869
557.3870
557.5876
557.7869
C143H244N32O23, M+nH ,2782.93
556.5854
556.7860
556.9867
557.1874
557.3881
557.5888
557.7894
m/z

Phe-Lys-Leu-Phe-Lys-Lys-Ile-Leu-Lys-Val-Leu-Lys-Lys-Val-Val-Phe-Trp-Val-Lys-Phe-Lys-NH$_2$ (BP13- KSLW)

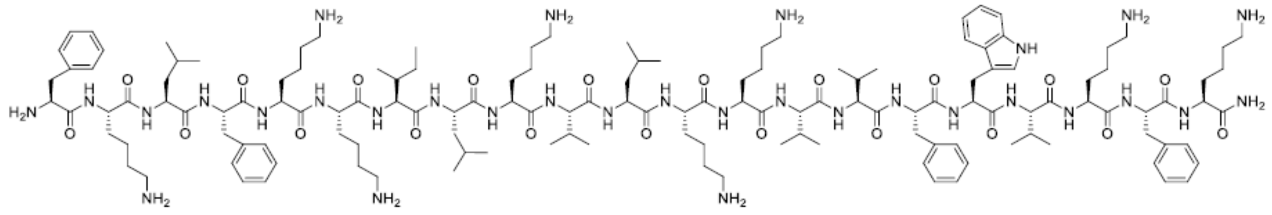

HPLC (λ=220 nm)

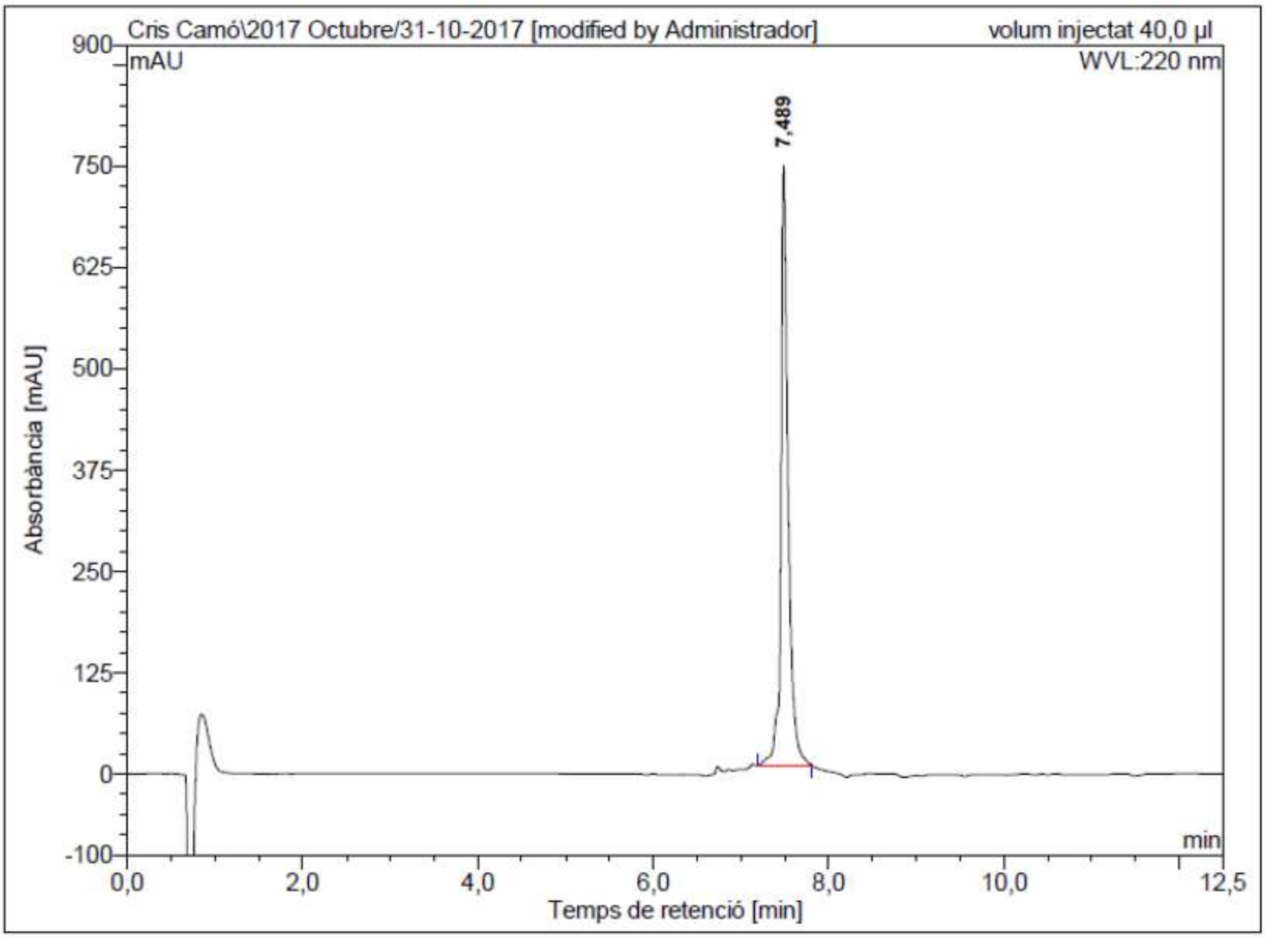

No.	Temps retenció min	alçada mAU	Area mAU*min	Area relativa %
1	7,49	740,412	71,122	100,00
Total:		740,412	71,122	100,00

ESI-MS (*m/z*)

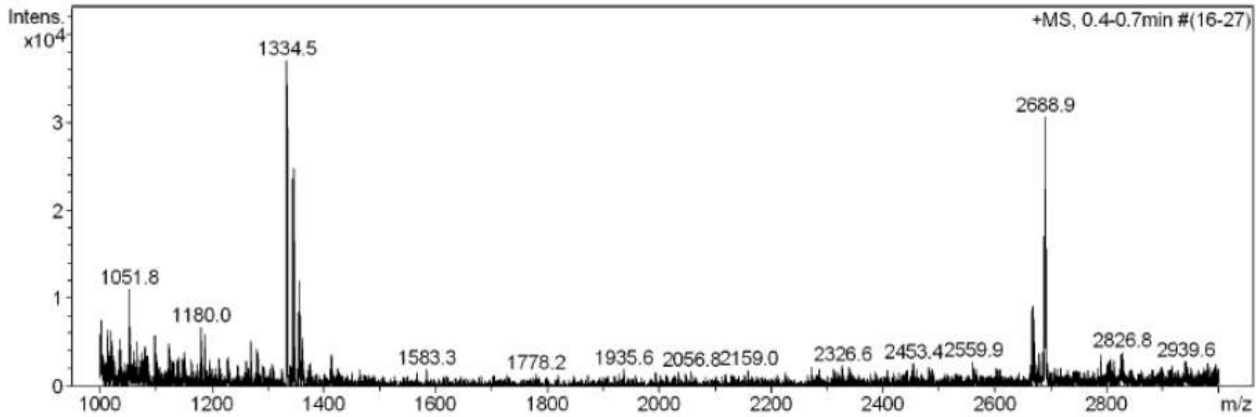

HRMS (*m/z*)

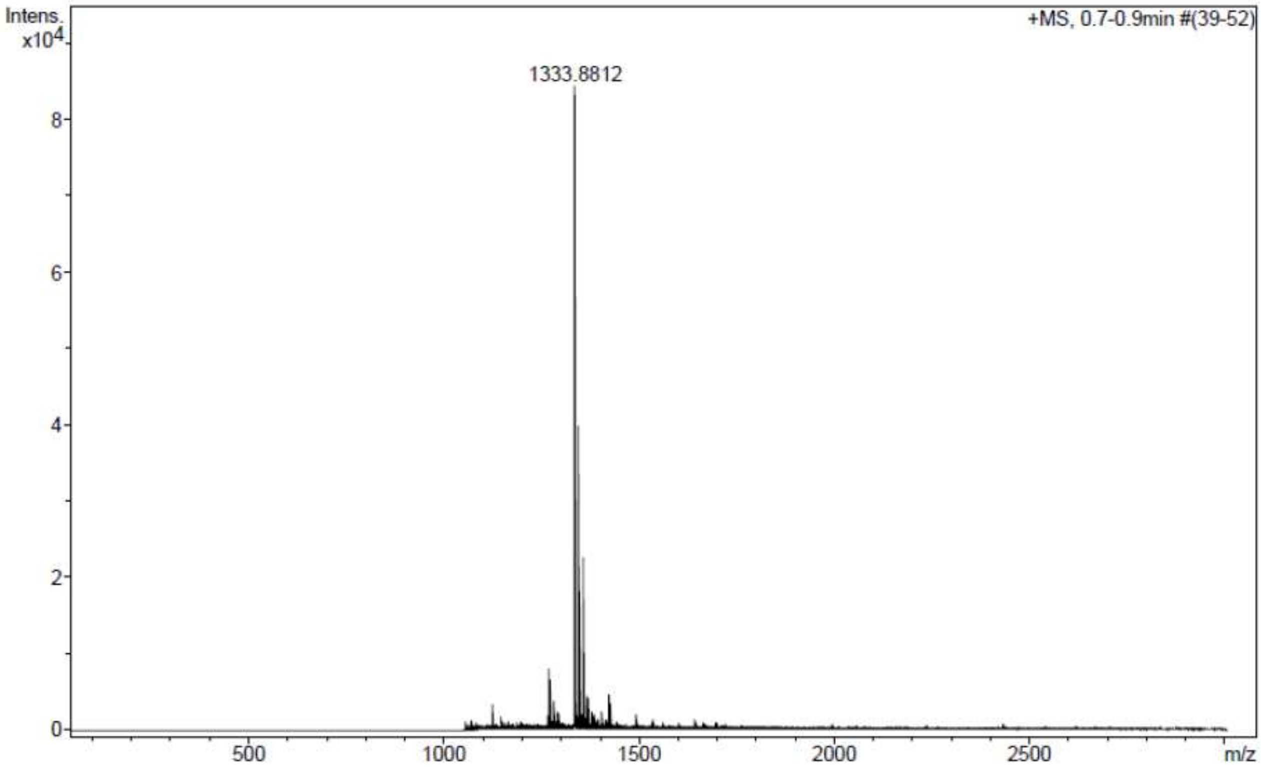

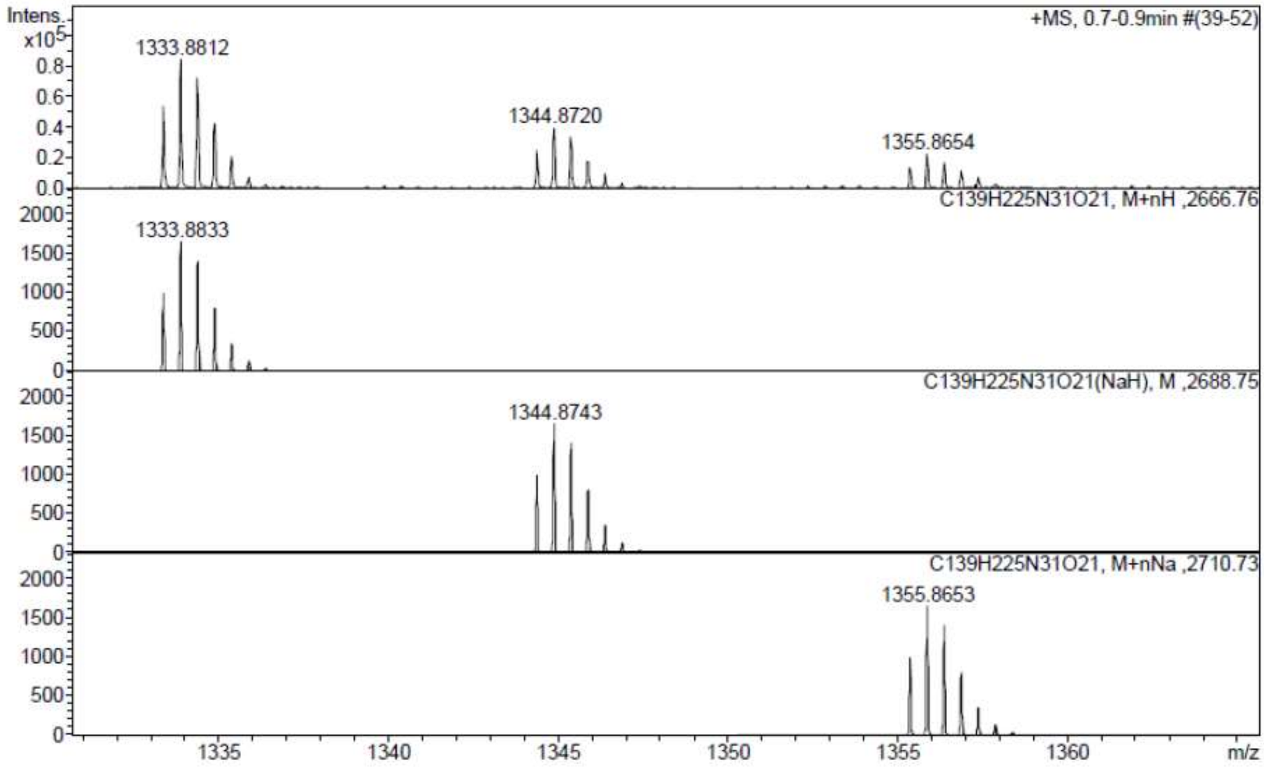

Intens.
x10^5
+MS, 0.7-0.9min #(39-52)
1333.8812
1344.8720
1355.8654
C139H225N31O21, M+nH ,2666.76
1333.8833
C139H225N31O21(NaH), M ,2688.75
1344.8743
C139H225N31O21, M+nNa ,2710.73
1355.8653
m/z

Lys-Lys-Val-Val-Phe-Trp-Val-Lys-Phe-Lys-Phe-Lys-Leu-Phe-Lys-Lys-Ile-Leu-Lys-Val-Leu-NH_2 (KSLW-BP13)

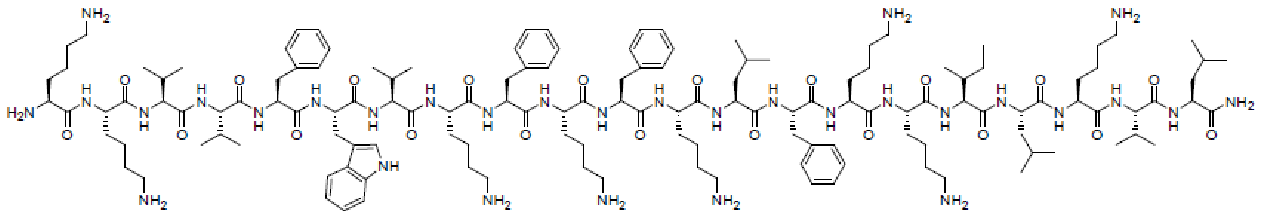

HPLC (λ=220 nm)

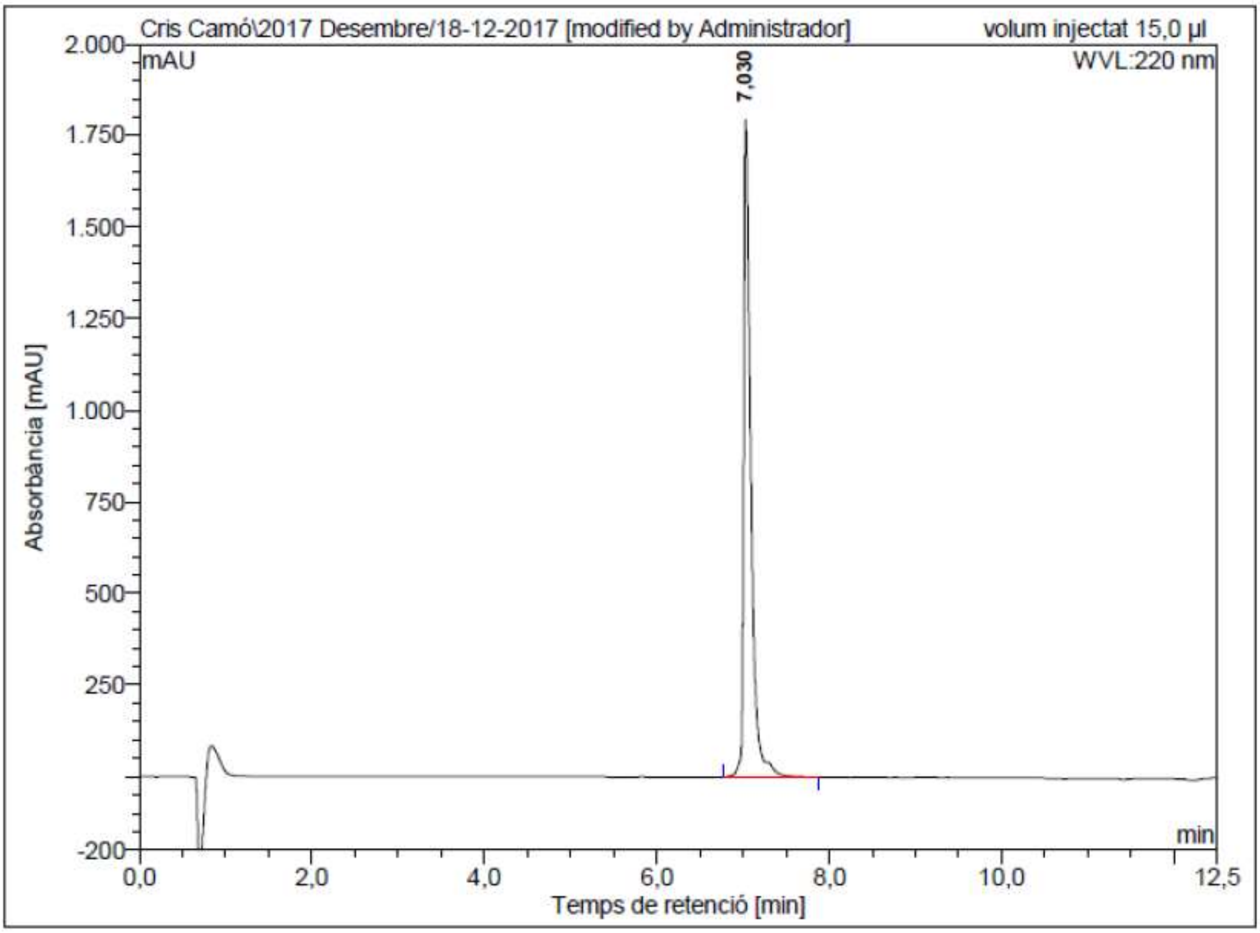

No.	Temps retenció min	alçada mAU	Area mAU*min	Area relativa %
1	7,03	1794,460	173,647	100,00
Total:		1794,460	173,647	100,00

ESI-MS (*m/z*)

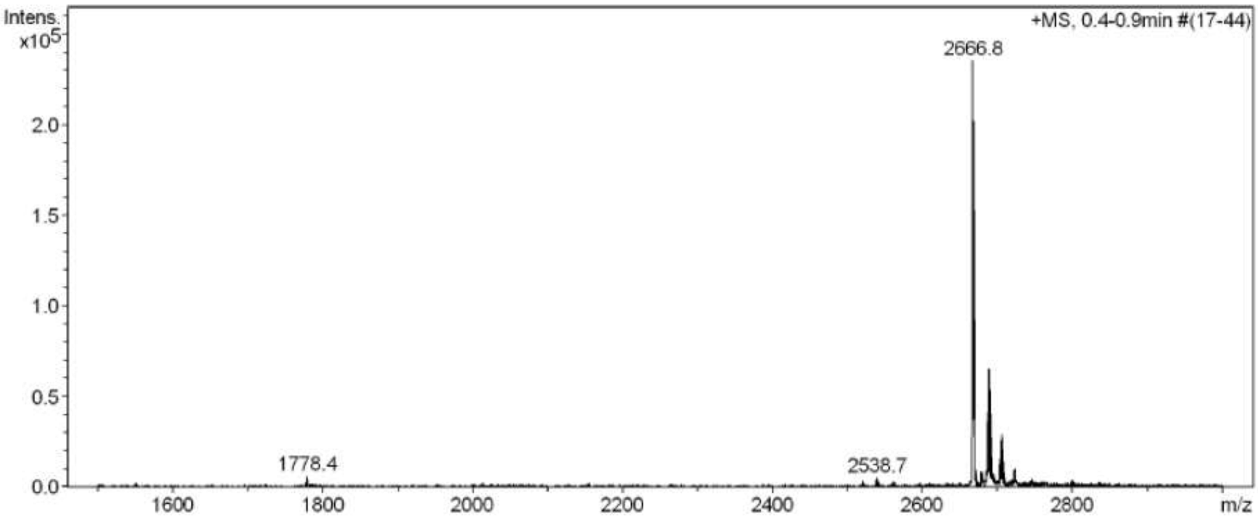

HRMS (*m/z*)

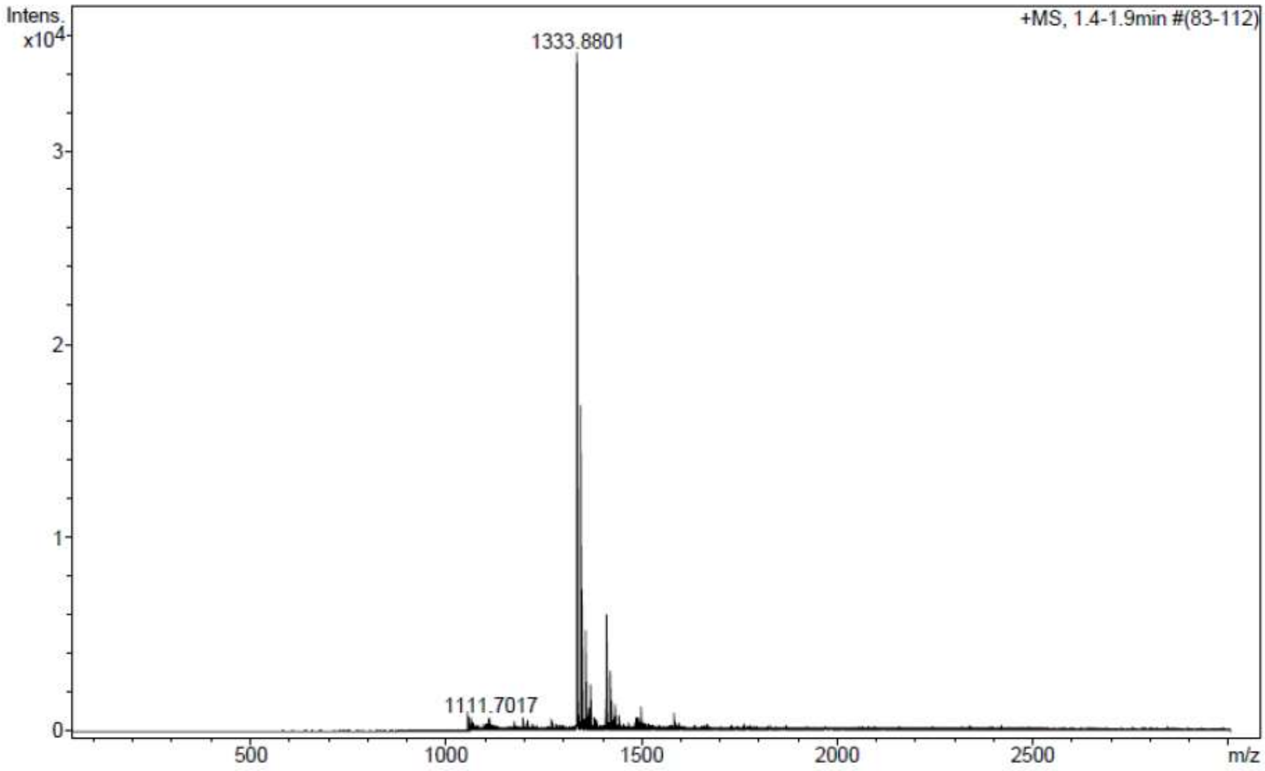

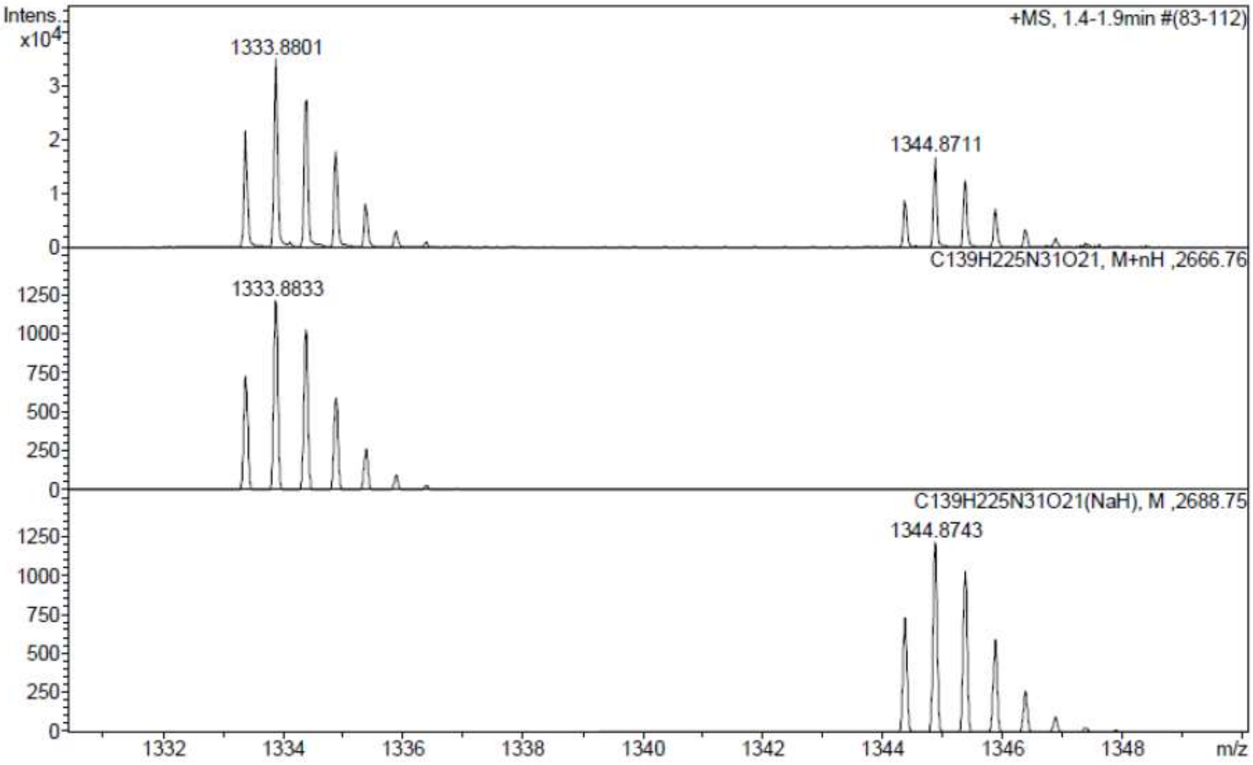
Intens.
x10^4
+MS, 1.4-1.9min #(83-112)
1333.8801
1344.8711
C139H225N31O21, M+nH ,2666.76
1333.8833
C139H225N31O21(NaH), M ,2688.75
1344.8743
m/z

Val-Trp-Asn-Gln-Pro-Val-Arg-Gly-Phe-Lys-Val-Tyr-Glu-Lys-Lys-Leu-Phe-Lys-Lys-Ile-Leu-Lys-Lys-Leu-NH_2 (Pep13-BP16)

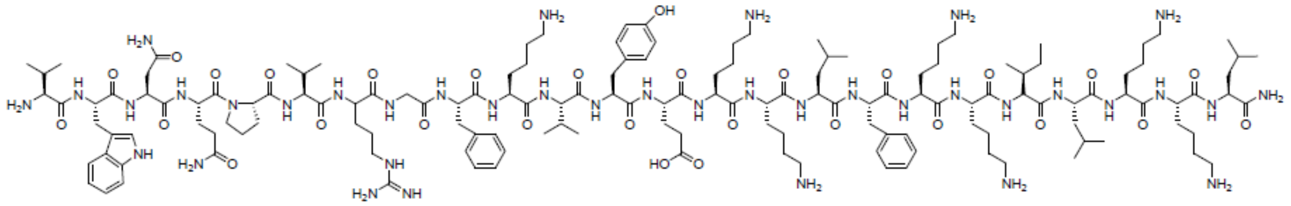

HPLC (λ=220 nm)

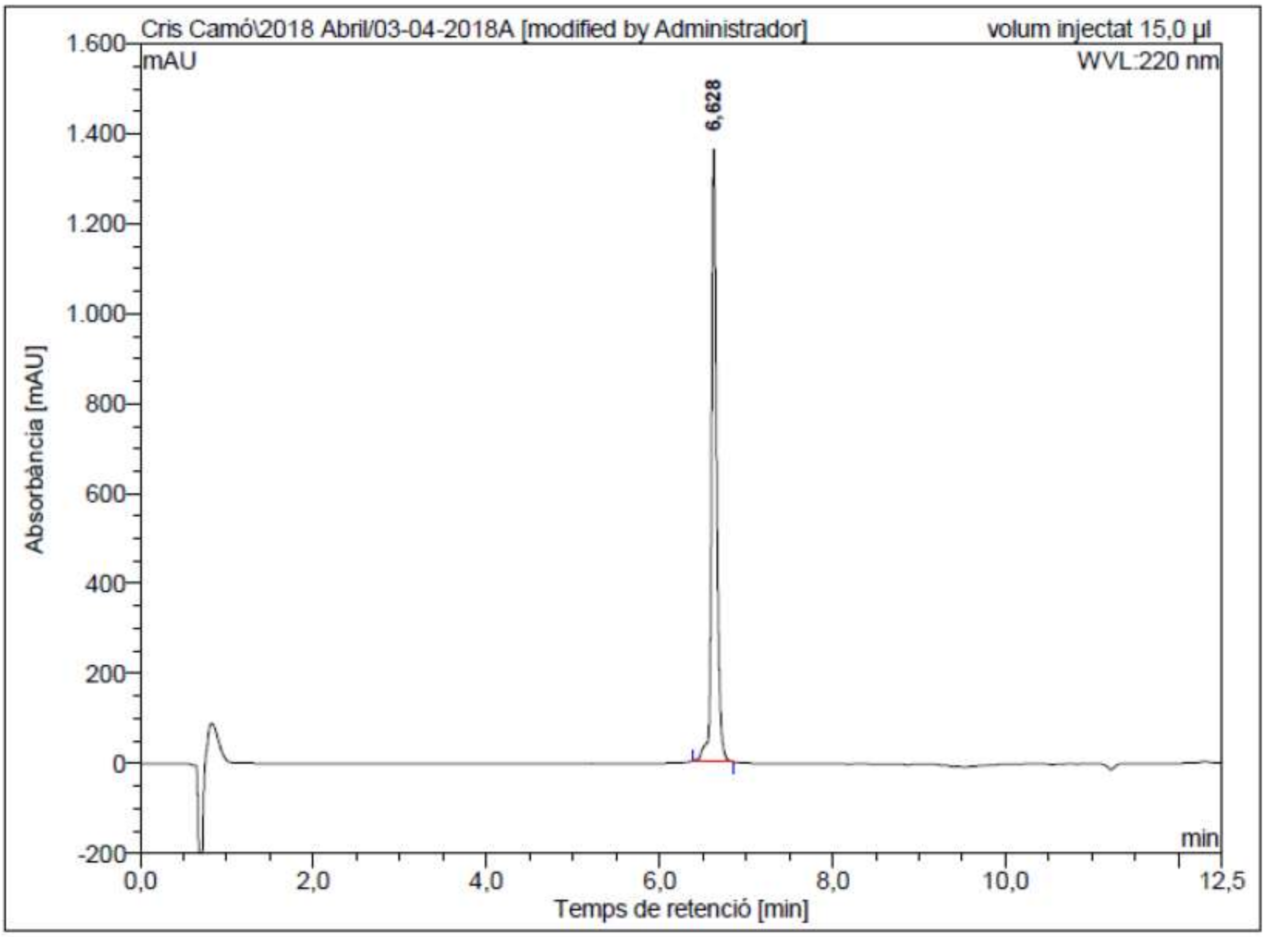

No.	Temps retenció<br>min	alçada<br>mAU	Area<br>mAU*min	Area relativa<br>%
1	6,63	1361,436	93,334	100,00
Total:		1361,436	93,334	100,00

ESI-MS (*m/z*)

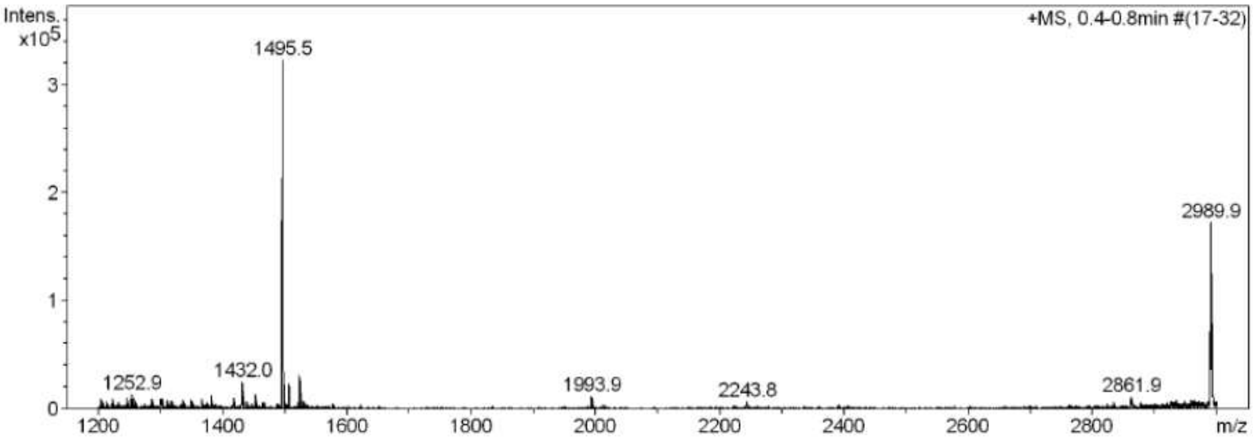

HRMS (*m/z*)

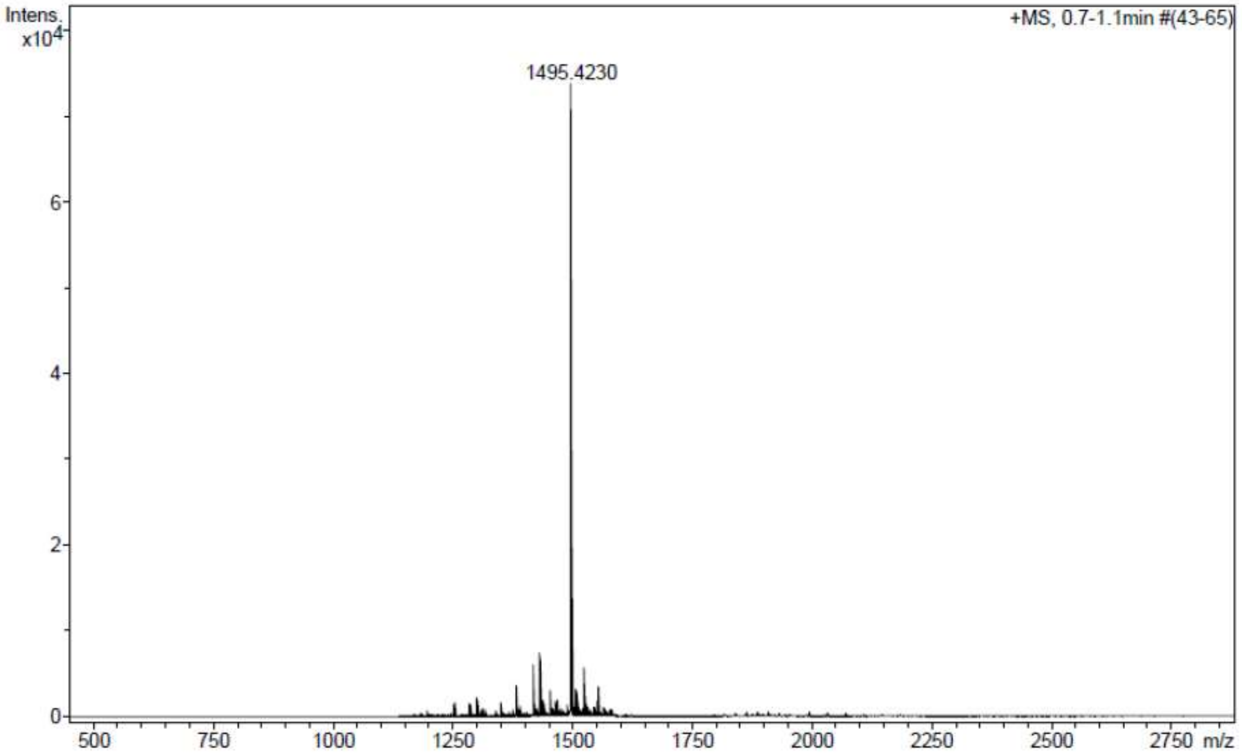

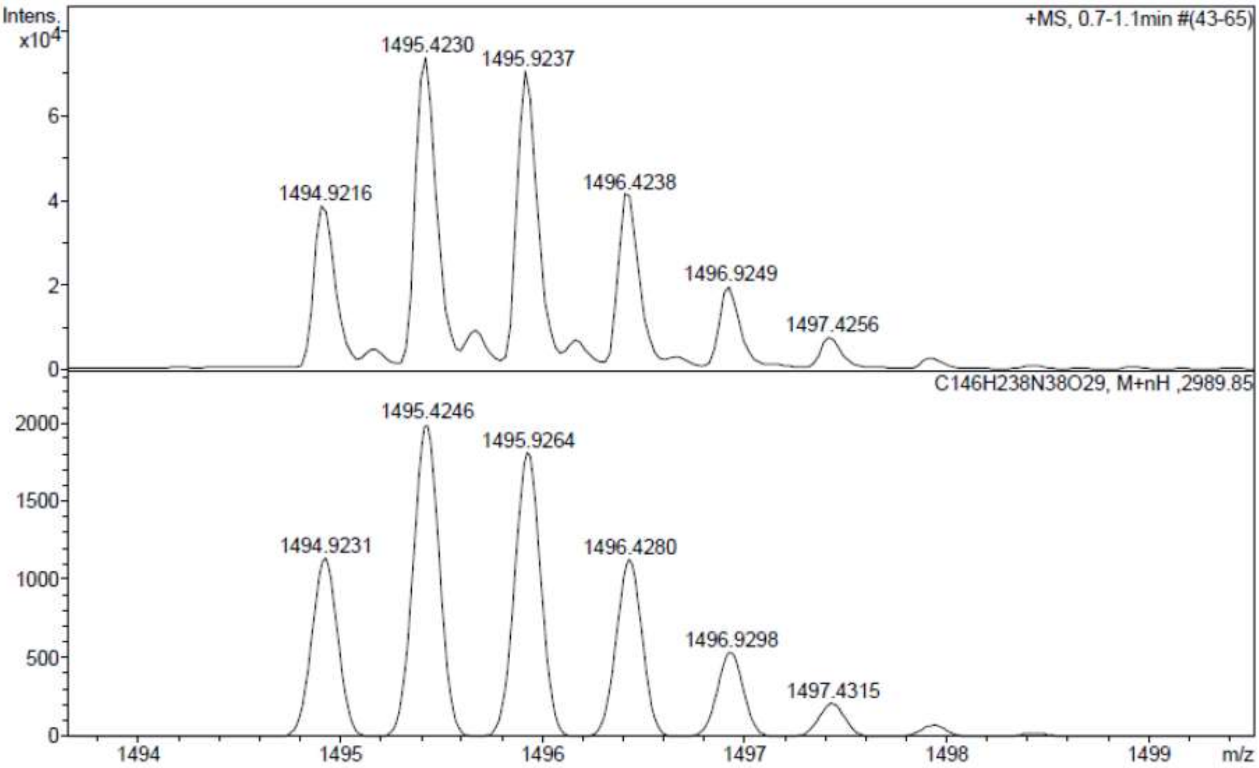

Intens.
x10^4
+MS, 0.7-1.1min #(43-65)
1495.4230
1495.9237
1494.9216
1496.4238
1496.9249
1497.4256
C146H238N38O29, M+nH ,2989.85
1495.4246
1495.9264
1494.9231
1496.4280
1496.9298
1497.4315
m/z

Lys-Lys-Leu-Phe-Lys-Lys-Ile-Leu-Lys-Lys-Leu-Val-Trp-Asn-Gln-Pro-Val-Arg-Gly-Phe-Lys-Val-Tyr-Glu-OH (BP16-Pep13)

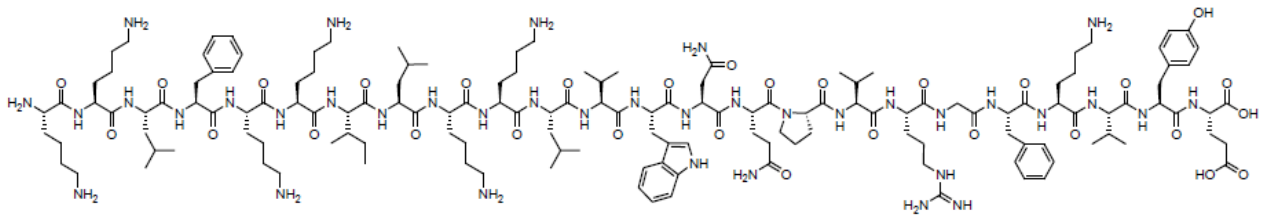

HPLC (λ=220 nm)

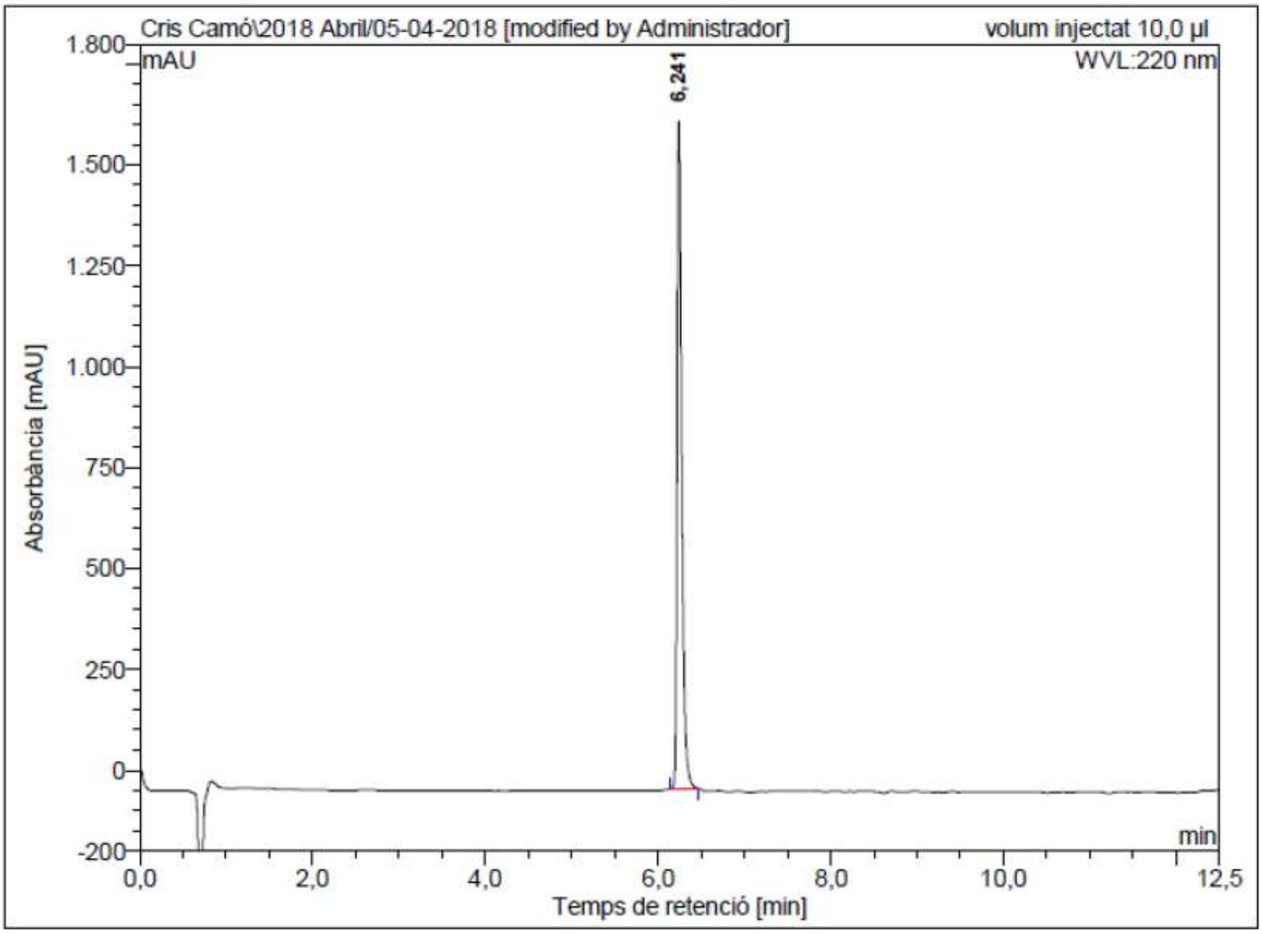

No.	Temps retenció min	alçada mAU	Area mAU*min	Area relativa %
1	6,24	1656,133	103,122	100,00
Total:		1656,133	103,122	100,00

ESI-MS (*m/z*)

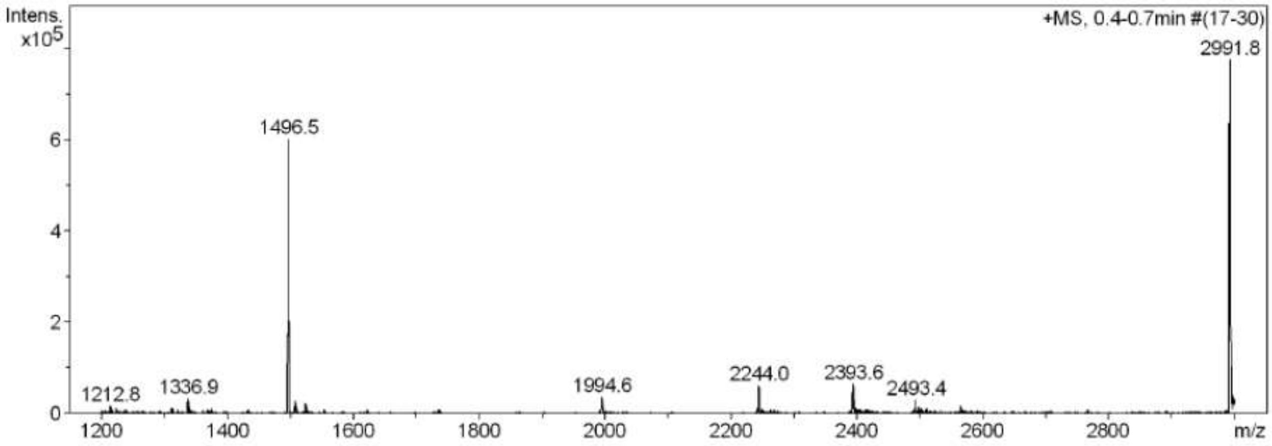

HRMS (*m/z*)

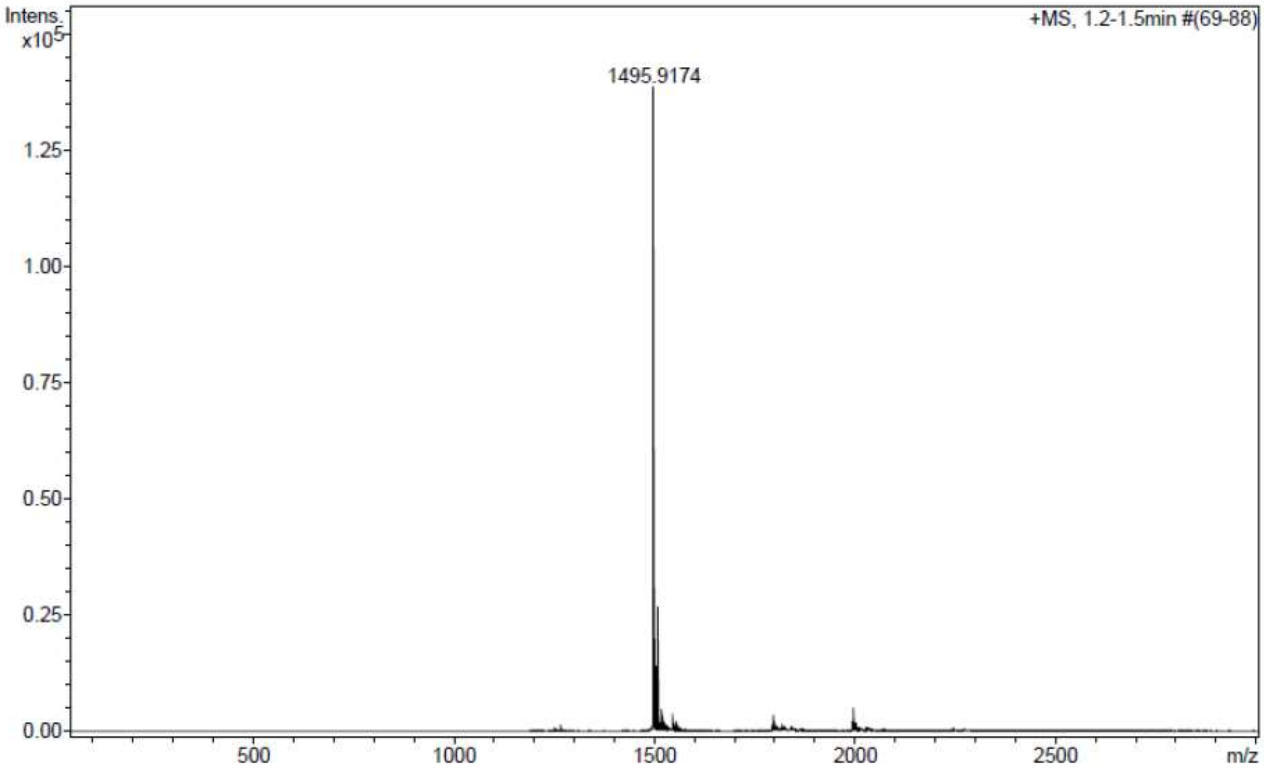

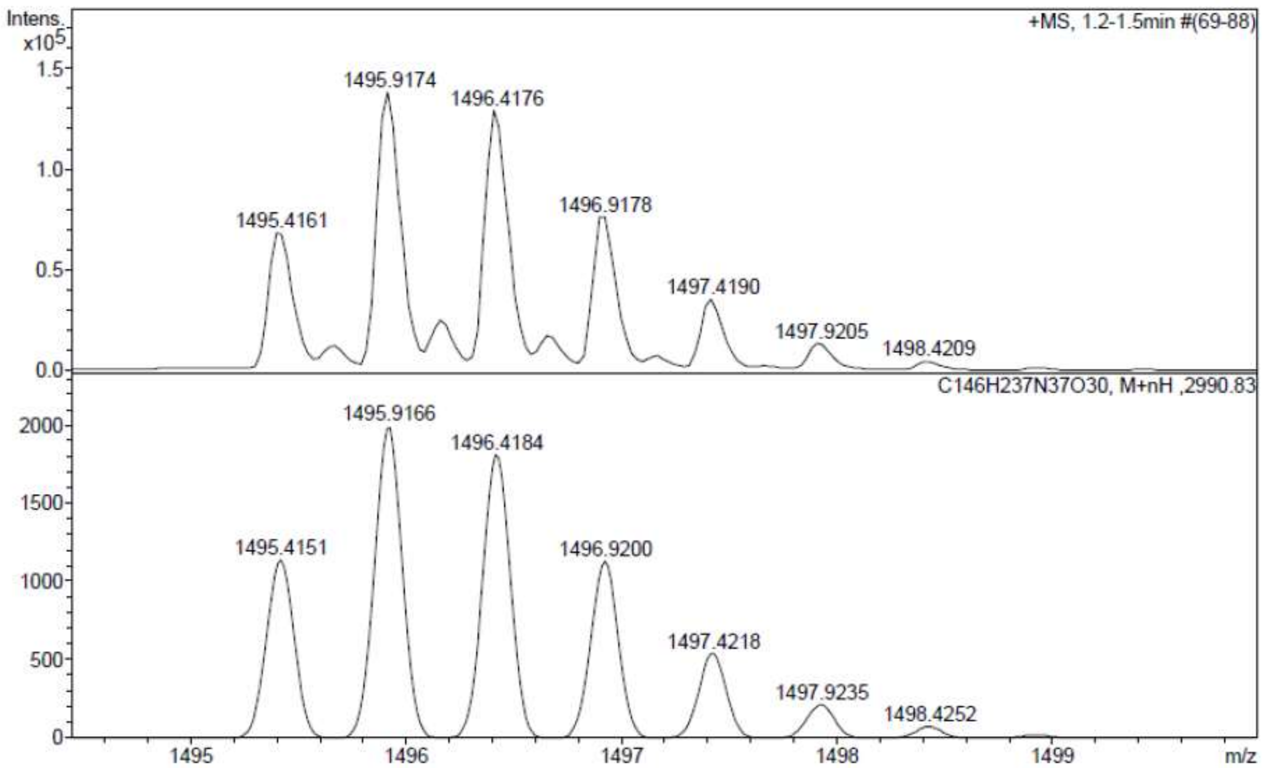
Intens.
x10^5
+MS, 1.2-1.5min #(69-88)
1495.4161
1495.9174
1496.4176
1496.9178
1497.4190
1497.9205
1498.4209
C146H237N37O30, M+nH ,2990.83
1495.4151
1495.9166
1496.4184
1496.9200
1497.4218
1497.9235
1498.4252
m/z

Val-Trp-Asn-Gln-Pro-Val-Arg-Gly-Phe-Lys-Val-Tyr-Glu-Lys-Lys-Leu-Phe-Lys-Lys-Ile-Leu-Lys-Tyr-Leu-NH_2 (Pep13-BP100)

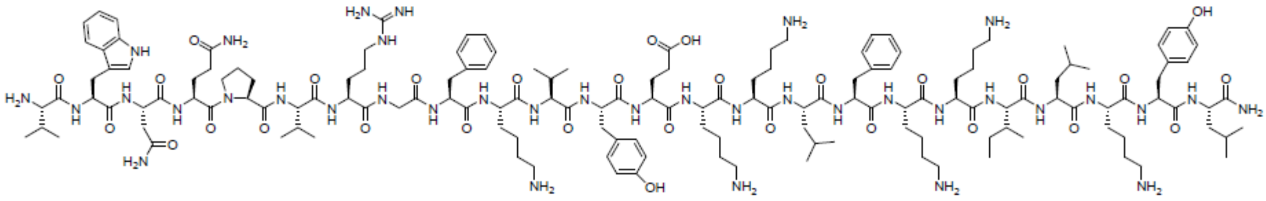

HPLC (λ=220 nm)

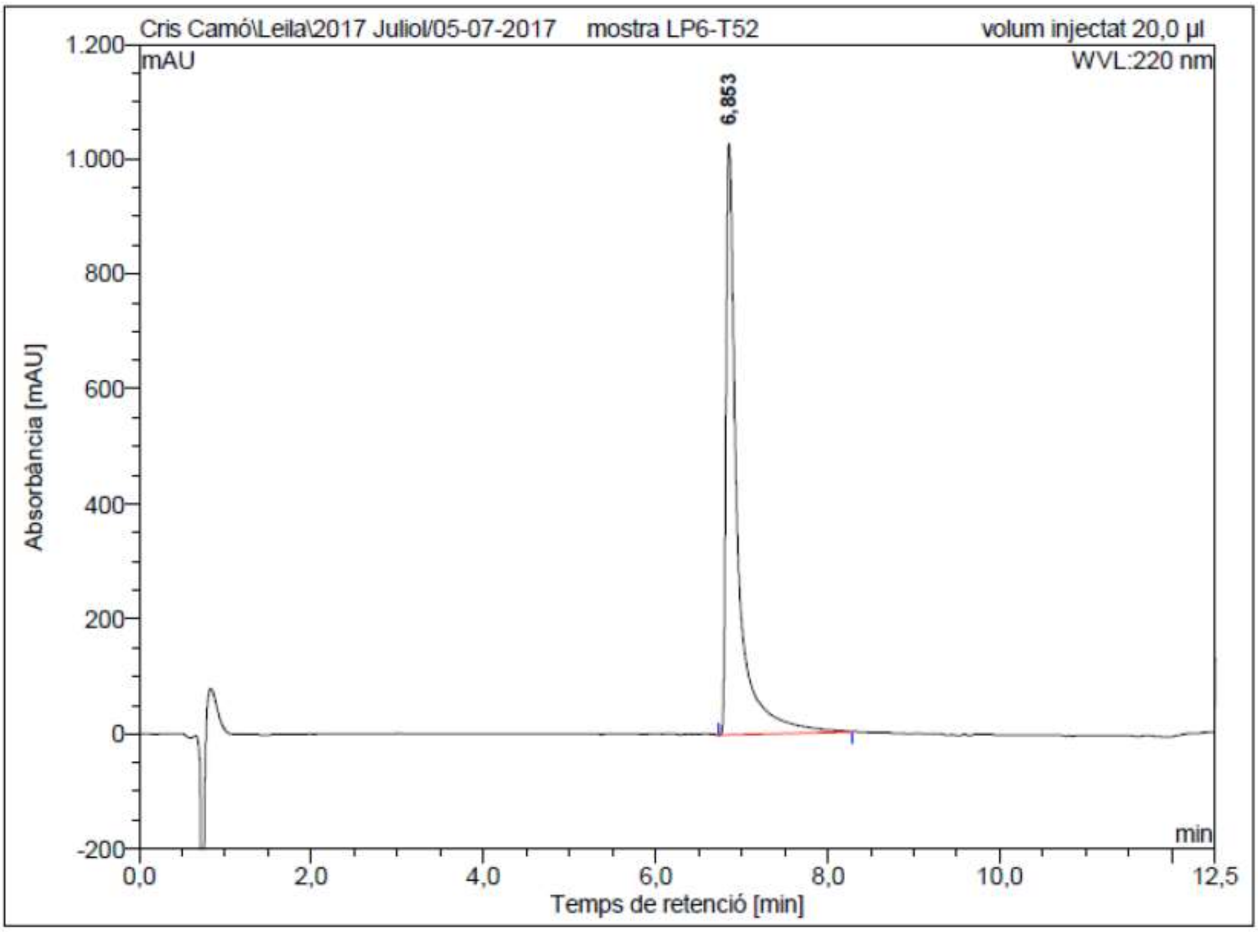

No.	Temps retenció min	alçada mAU	Area mAU*min	Area relativa %
1	6,85	1028,500	158,294	100,00
Total:		1028,500	158,294	100,00

ESI-MS (*m/z*)

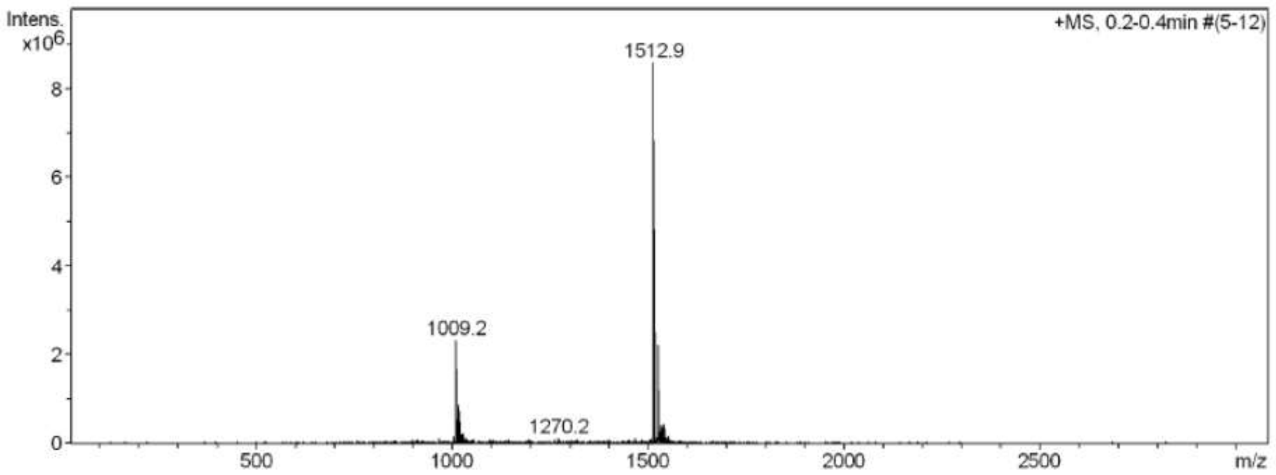

HRMS (*m/z*)

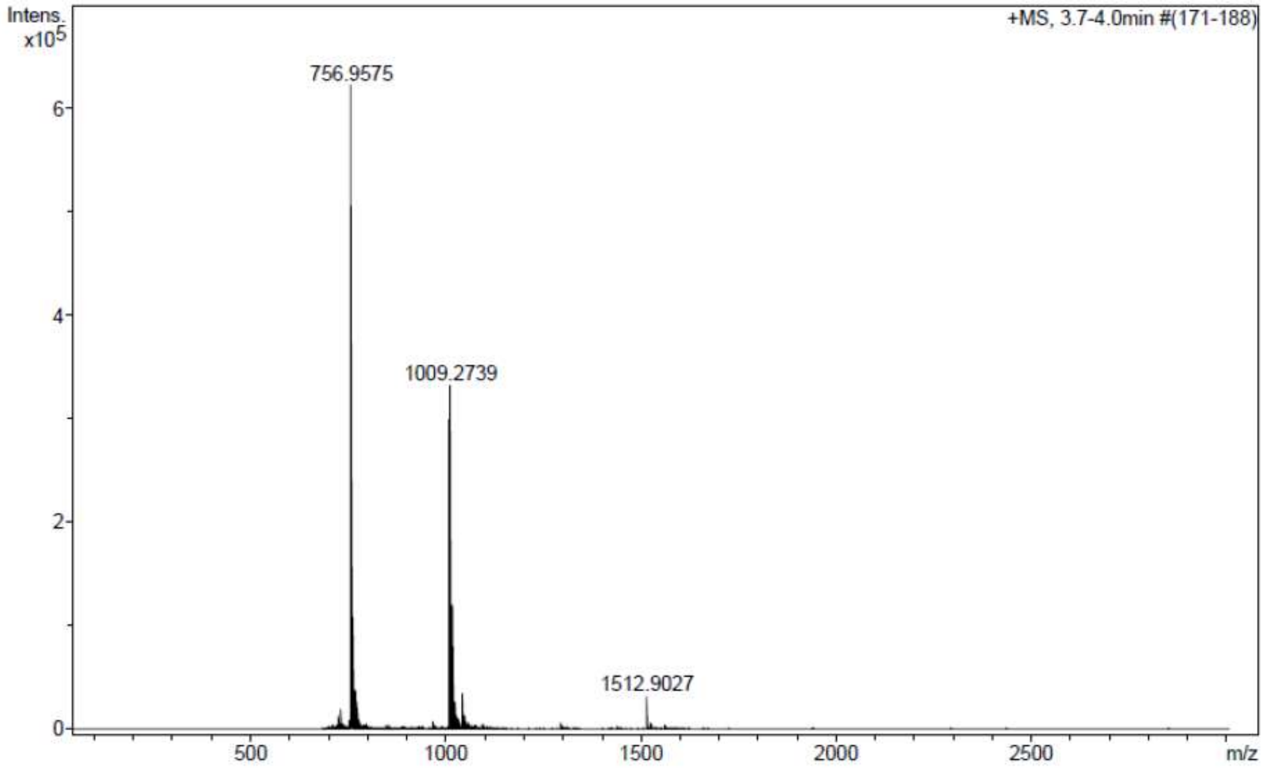

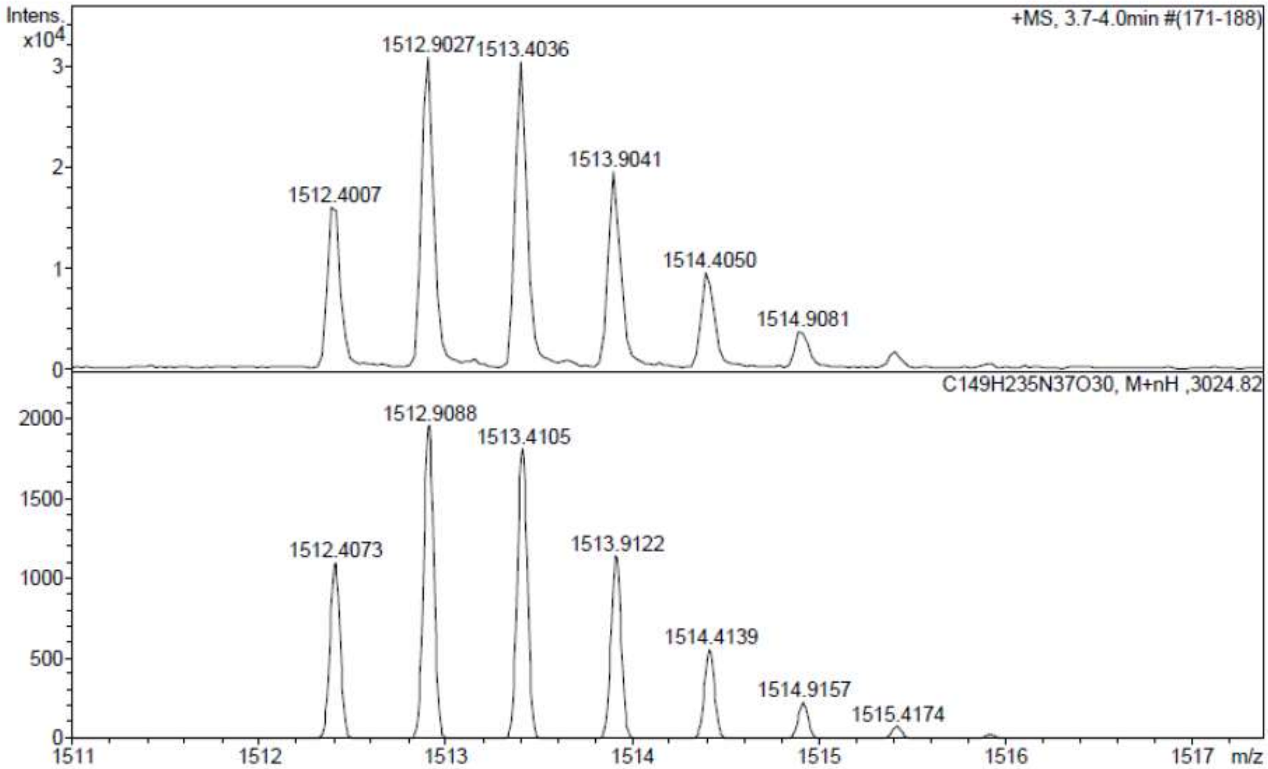
Intens.
x10^4
+MS, 3.7-4.0min #(171-188)
1512.4007
1512.9027
1513.4036
1513.9041
1514.4050
1514.9081
C149H235N37O30, M+nH ,3024.82
1512.4073
1512.9088
1513.4105
1513.9122
1514.4139
1514.9157
1515.4174
m/z

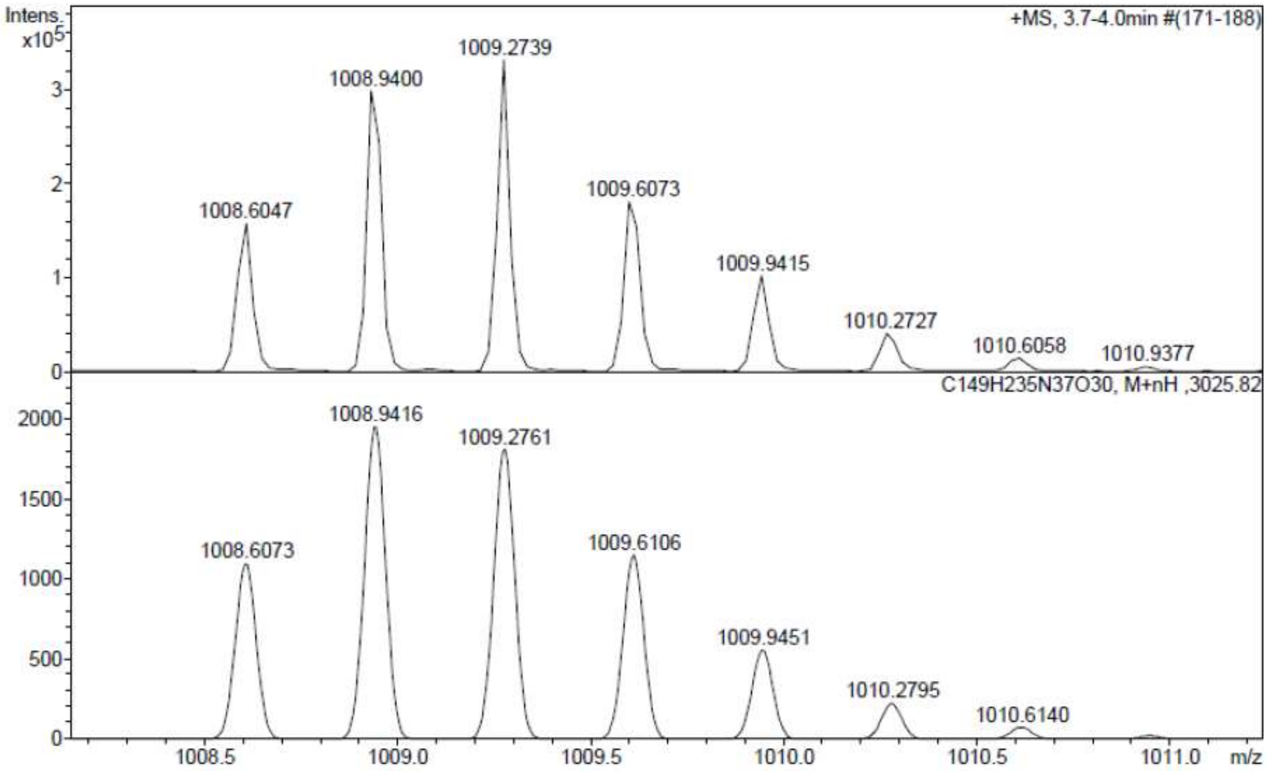
Intens.
x10^5
+MS, 3.7-4.0min #(171-188)
1008.6047
1008.9400
1009.2739
1009.6073
1009.9415
1010.2727
1010.6058
1010.9377
C149H235N37O30, M+nH ,3025.82
1008.6073
1008.9416
1009.2761
1009.6106
1009.9451
1010.2795
1010.6140
m/z

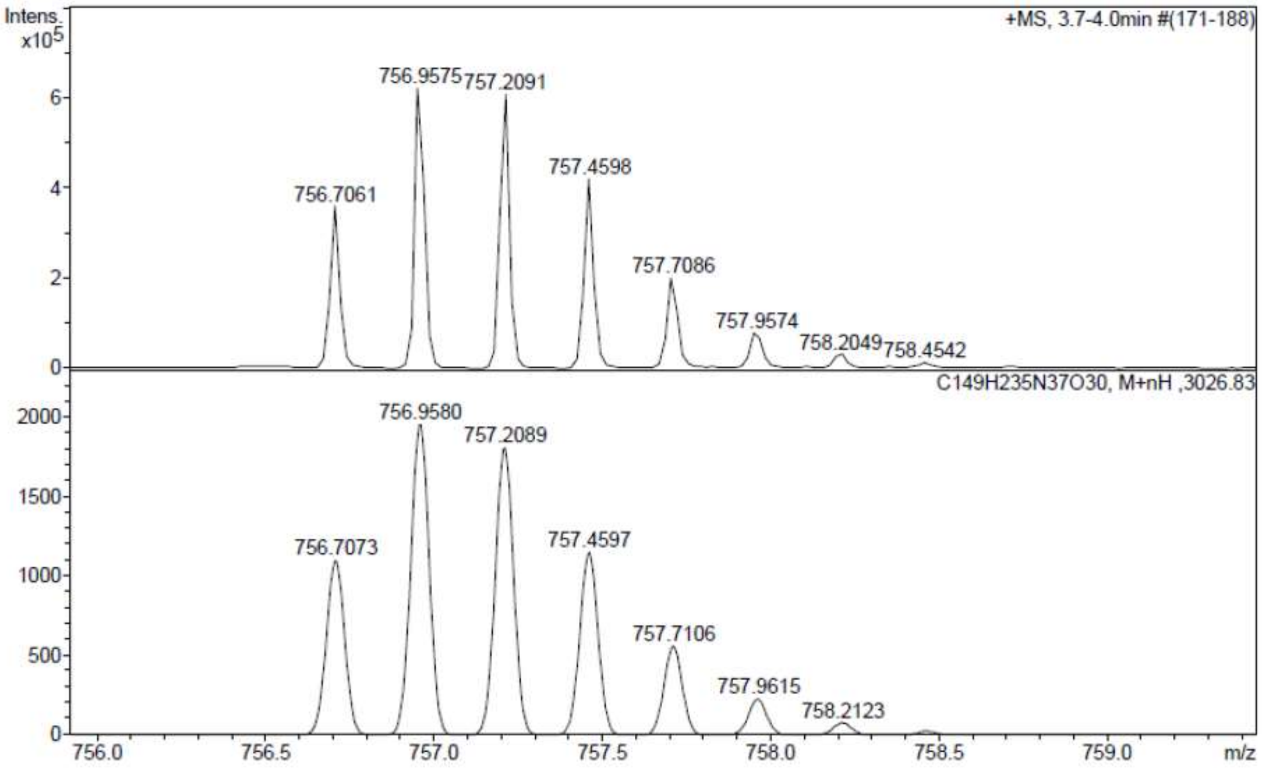
Intens.
x10^5
+MS, 3.7-4.0min #(171-188)
756.9575
757.2091
757.4598
756.7061
757.7086
757.9574
758.2049
758.4542
C149H235N37O30, M+nH ,3026.83
756.9580
757.2089
756.7073
757.4597
757.7106
757.9615
758.2123
756.0
756.5
757.0
757.5
758.0
758.5
759.0
m/z

Lys-Lys-Leu-Phe-Lys-Lys-Ile-Leu-Lys-Tyr-Leu-Val-Trp-Asn-Gln-Pro-Val-Arg-Gly-Phe-Lys-Val-Tyr-Glu-OH (BP100-Pep13)

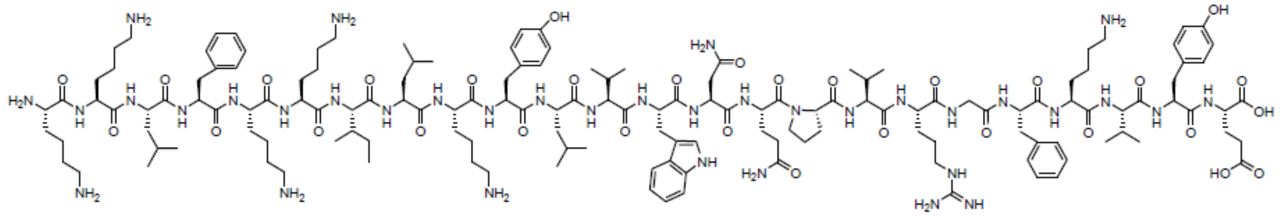

HPLC (λ=220 nm)

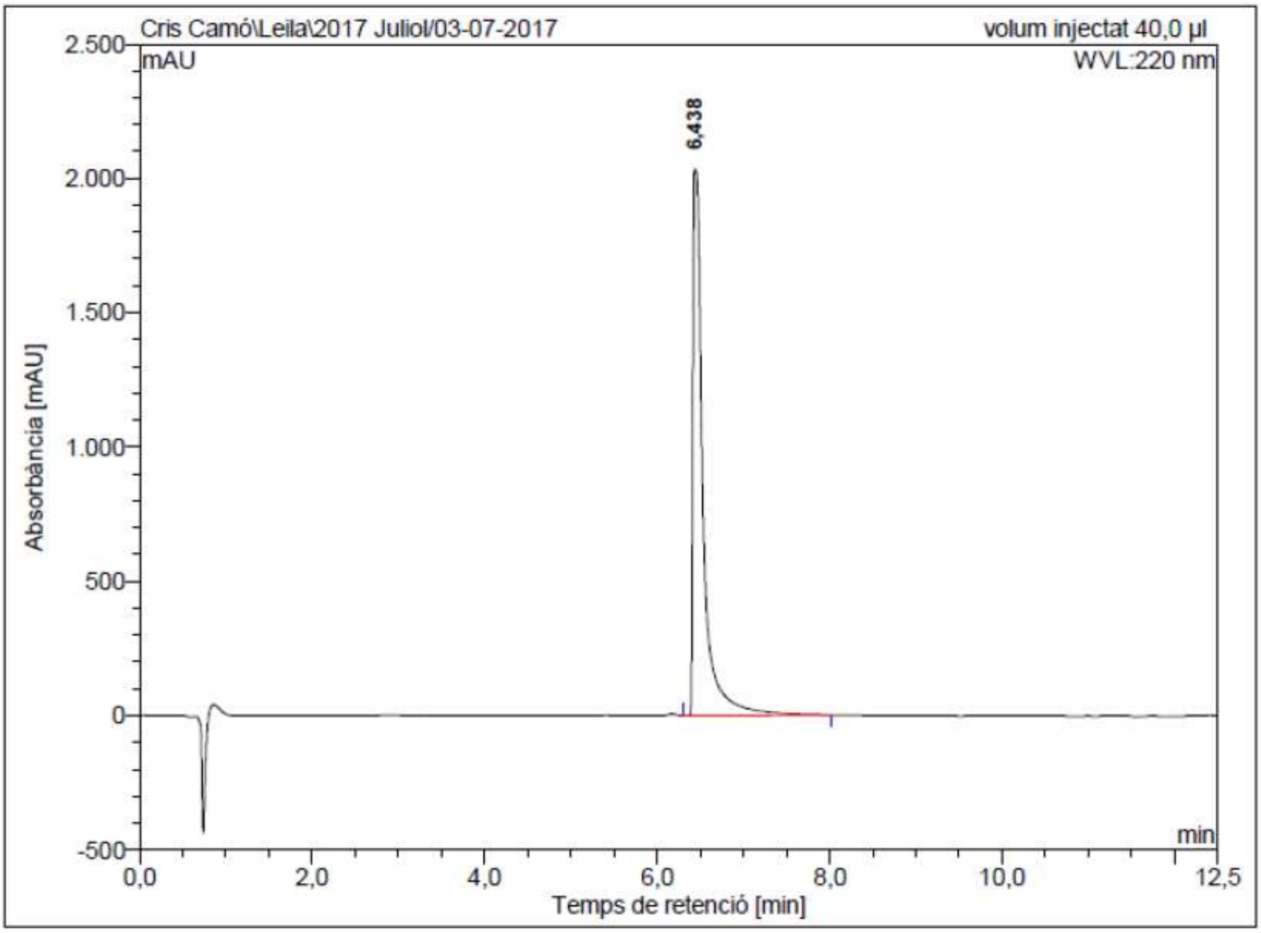

No.	Temps retenció min	alçada mAU	Area mAU*min	Area relativa %
1	6,44	2035,594	279,119	100,00
Total:		2035,594	279,119	100,00

ESI-MS (*m/z*)

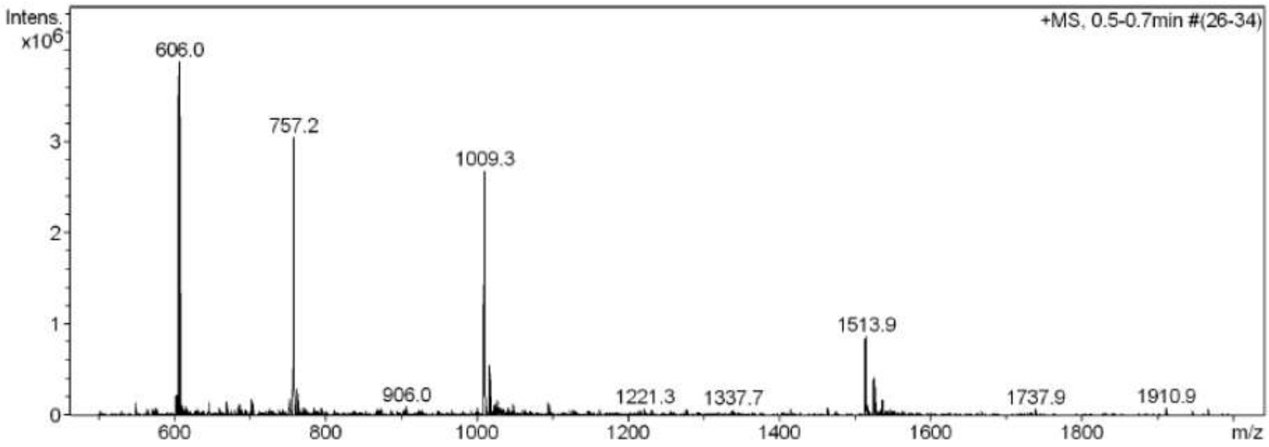

HRMS (*m/z*)

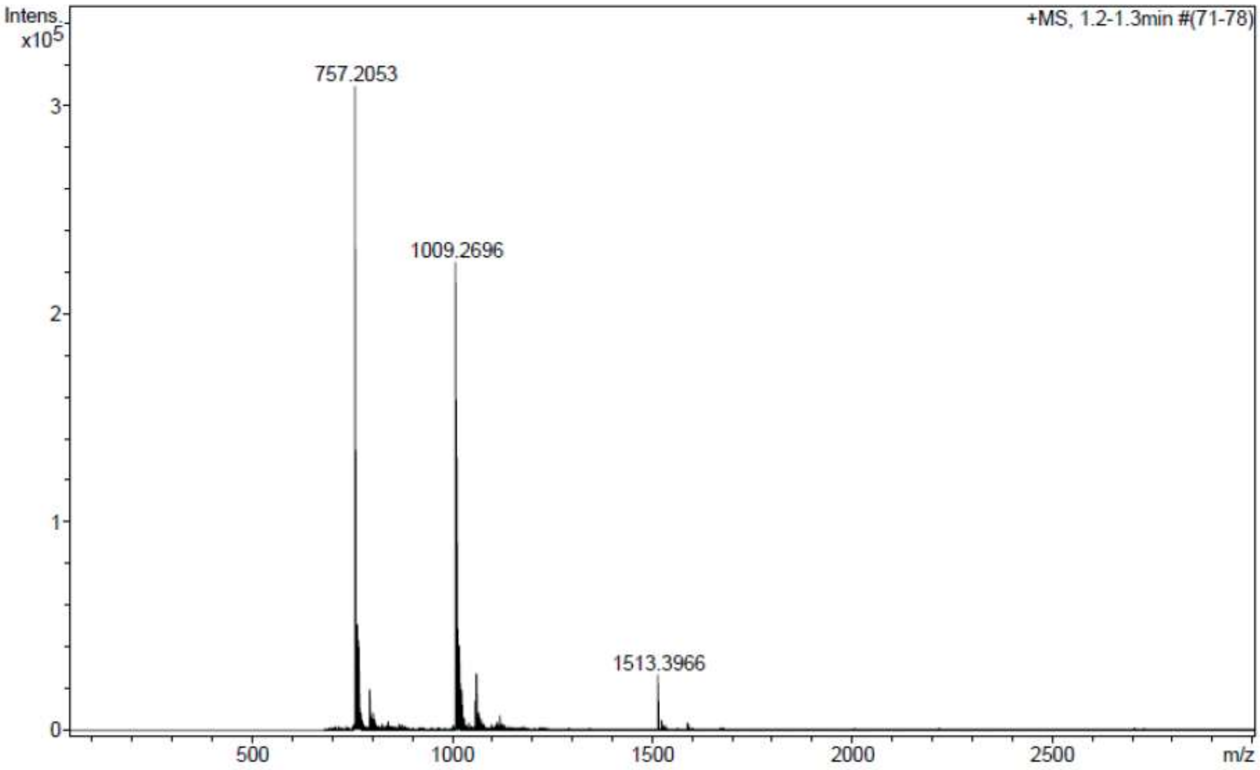

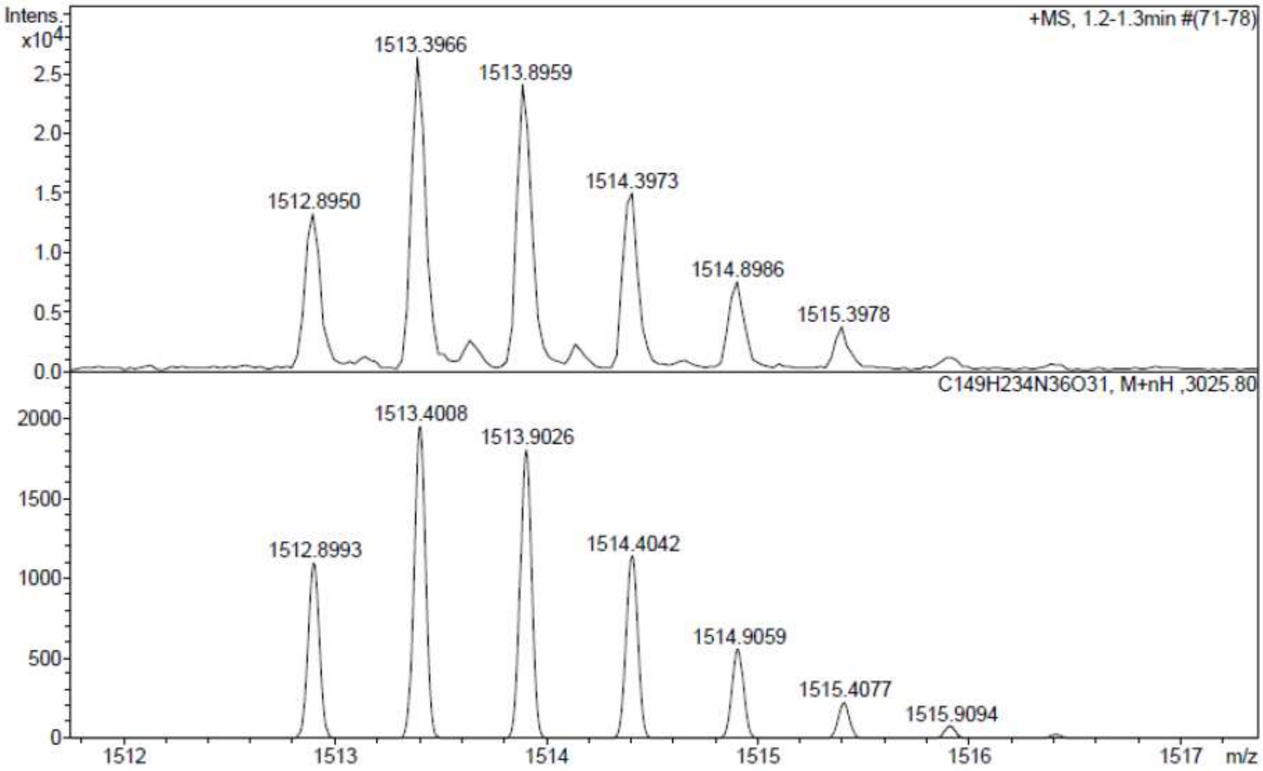
Intens.
x10^4
+MS, 1.2-1.3min #(71-78)
1512.8950
1513.3966
1513.8959
1514.3973
1514.8986
1515.3978
C149H234N36O31, M+nH ,3025.80
1512.8993
1513.4008
1513.9026
1514.4042
1514.9059
1515.4077
1515.9094
m/z

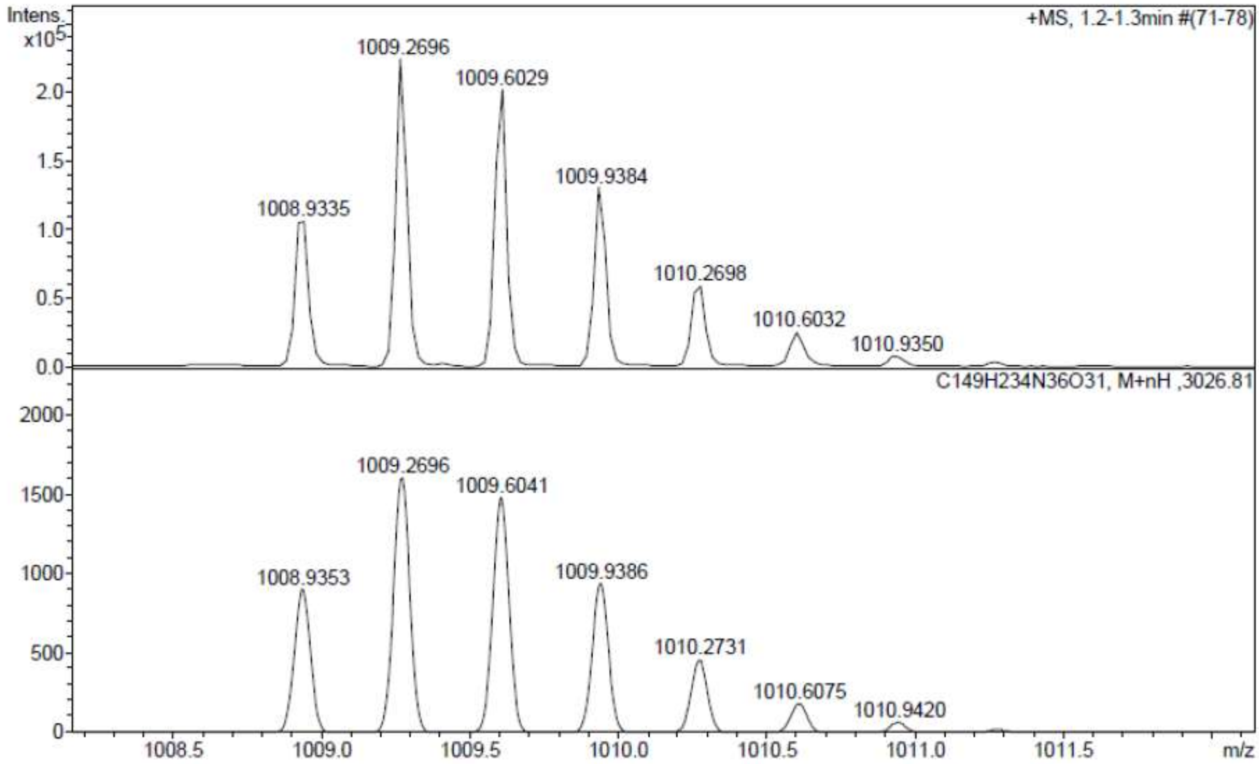
Intens.
x10^5
+MS, 1.2-1.3min #(71-78)
1008.9335
1009.2696
1009.6029
1009.9384
1010.2698
1010.6032
1010.9350
C149H234N36O31, M+nH ,3026.81
1008.9353
1009.2696
1009.6041
1009.9386
1010.2731
1010.6075
1010.9420
m/z

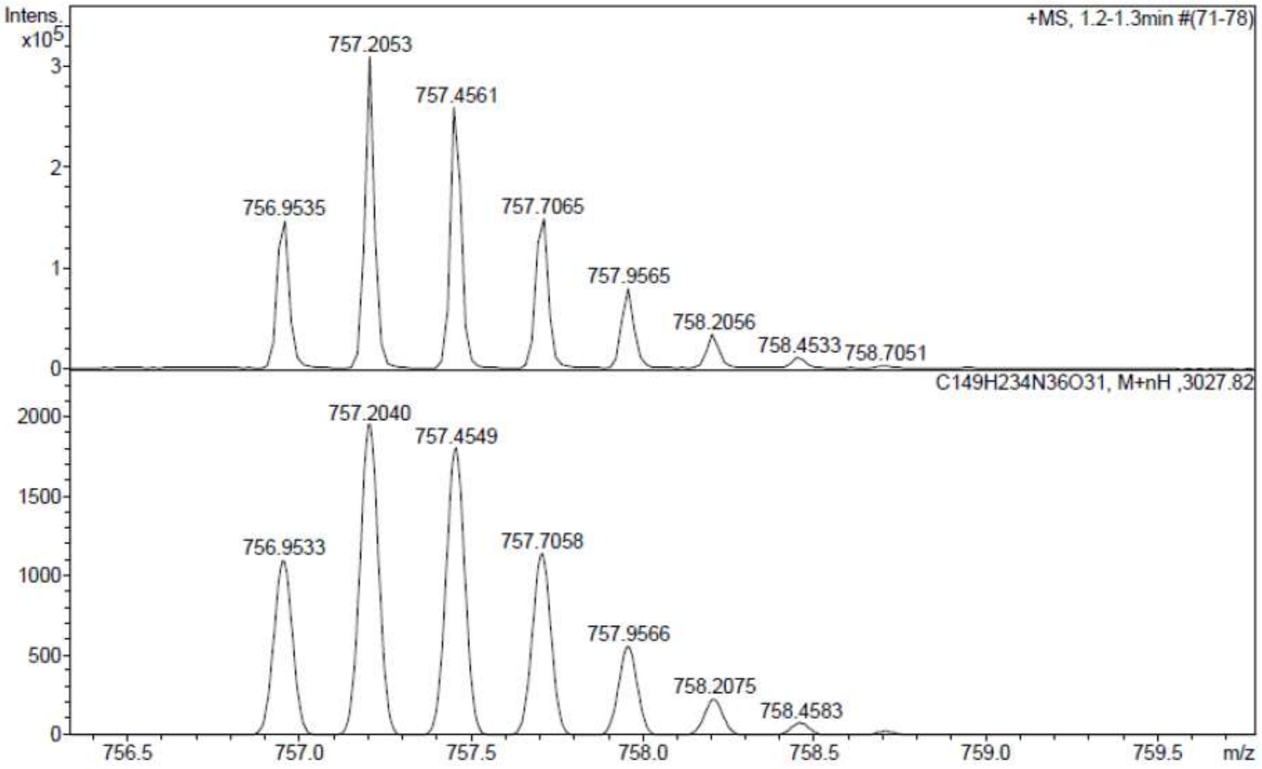

Intens.
x10^5
+MS, 1.2-1.3min #(71-78)
757.2053
757.4561
756.9535
757.7065
757.9565
758.2056
758.4533
758.7051
C149H234N36O31, M+nH ,3027.82
757.2040
757.4549
756.9533
757.7058
757.9566
758.2075
758.4583
m/z

Val-Trp-Asn-Gln-Pro-Val-Arg-Gly-Phe-Lys-Val-Tyr-Glu- Lys-Lys-Leu-D-Phe-Lys-Lys-Ile-Leu-Lys-Tyr-Leu-NH_2 (Pep13-BP143)

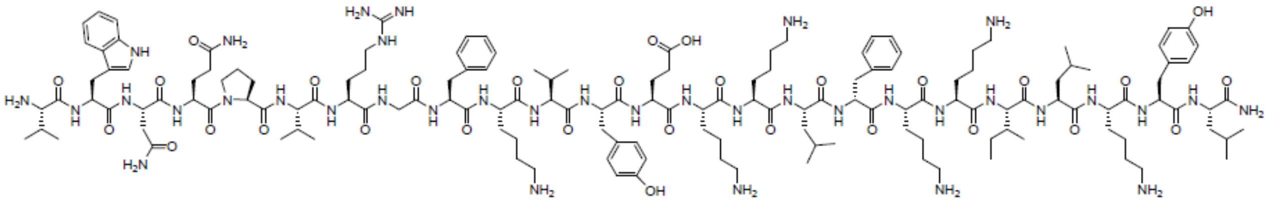

HPLC (λ=220 nm)

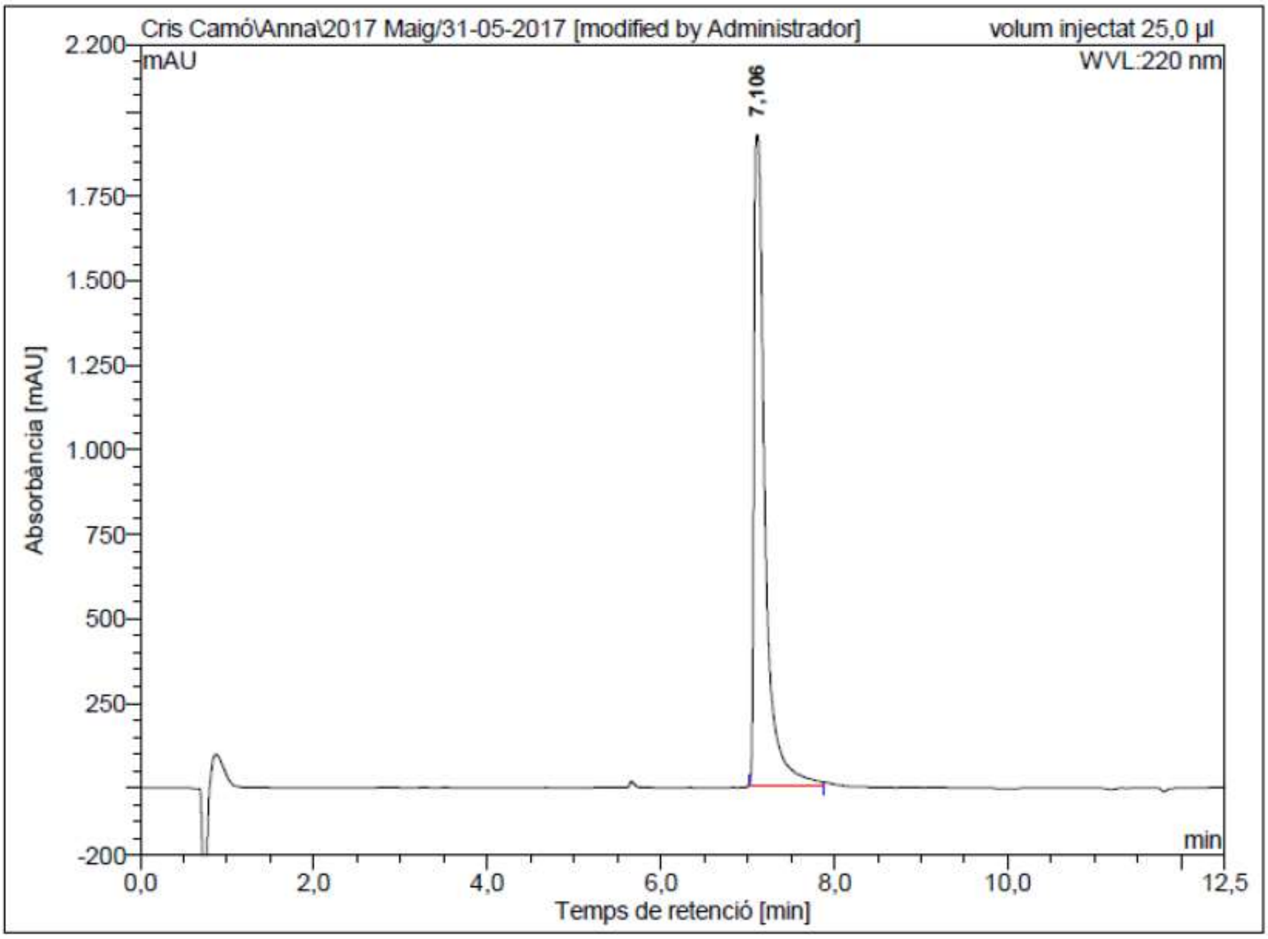

No.	Temps retenció min	alçada mAU	Area mAU*min	Area relativa %
1	7,11	1925,876	283,494	100,00
Total:		1925,876	283,494	100,00

ESI-MS (*m/z*)

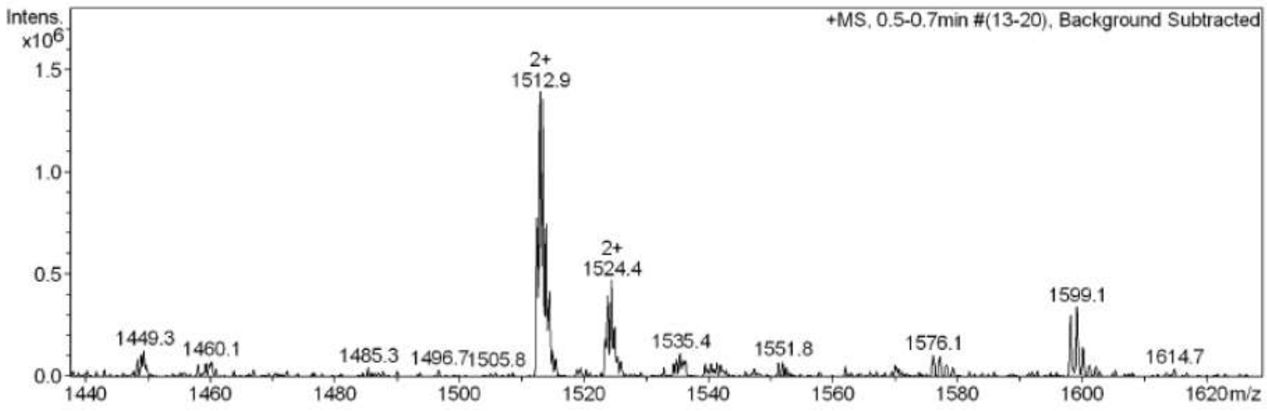

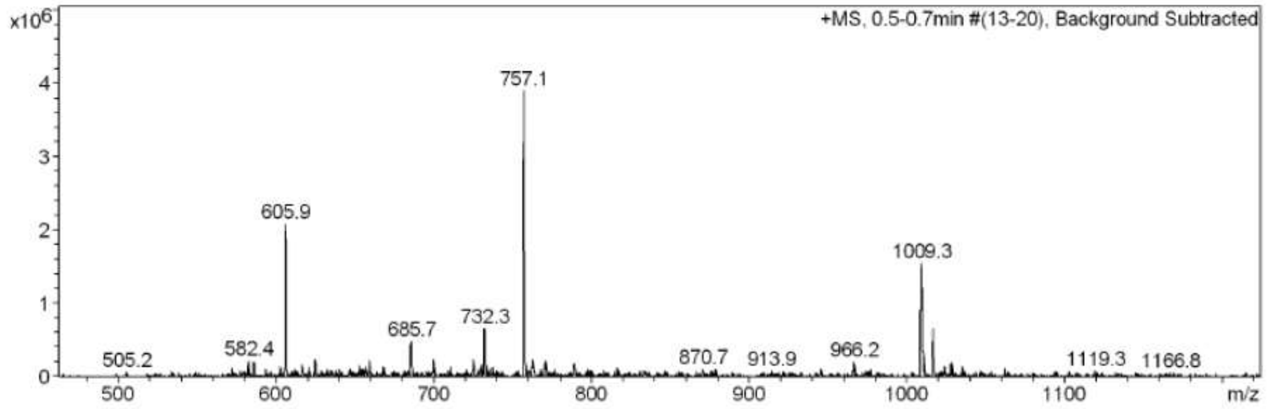

HRMS (*m/z*)

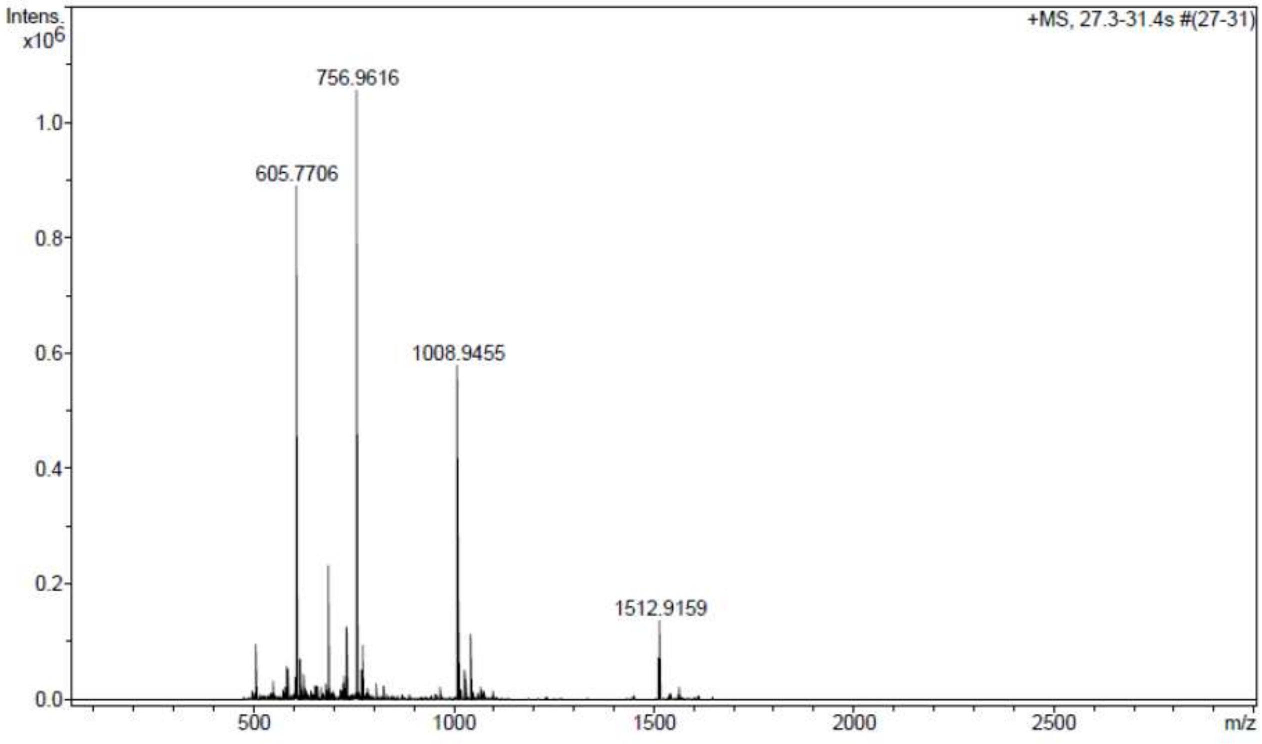

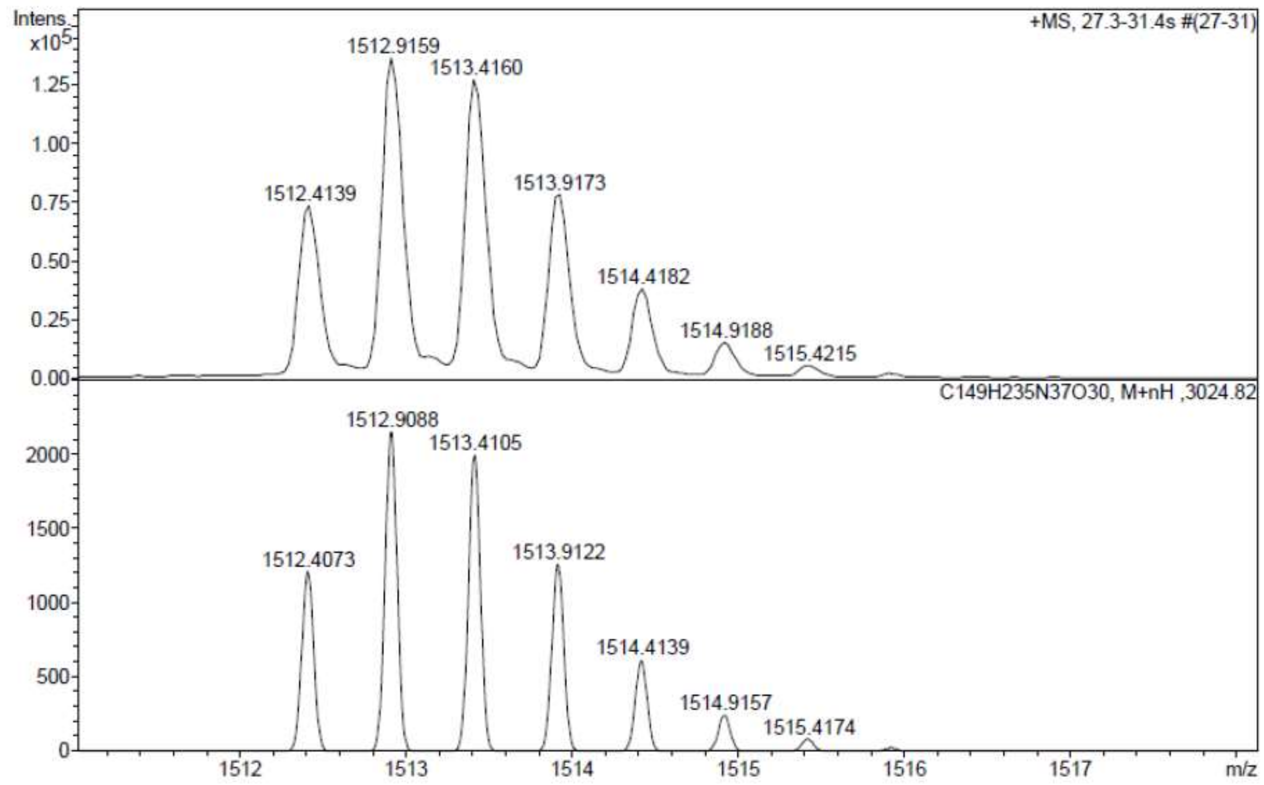
Intens. x10^5
+MS, 27.3-31.4s #(27-31)
1512.4139
1512.9159
1513.4160
1513.9173
1514.4182
1514.9188
1515.4215
C149H235N37O30, M+nH ,3024.82
1512.4073
1512.9088
1513.4105
1513.9122
1514.4139
1514.9157
1515.4174
m/z

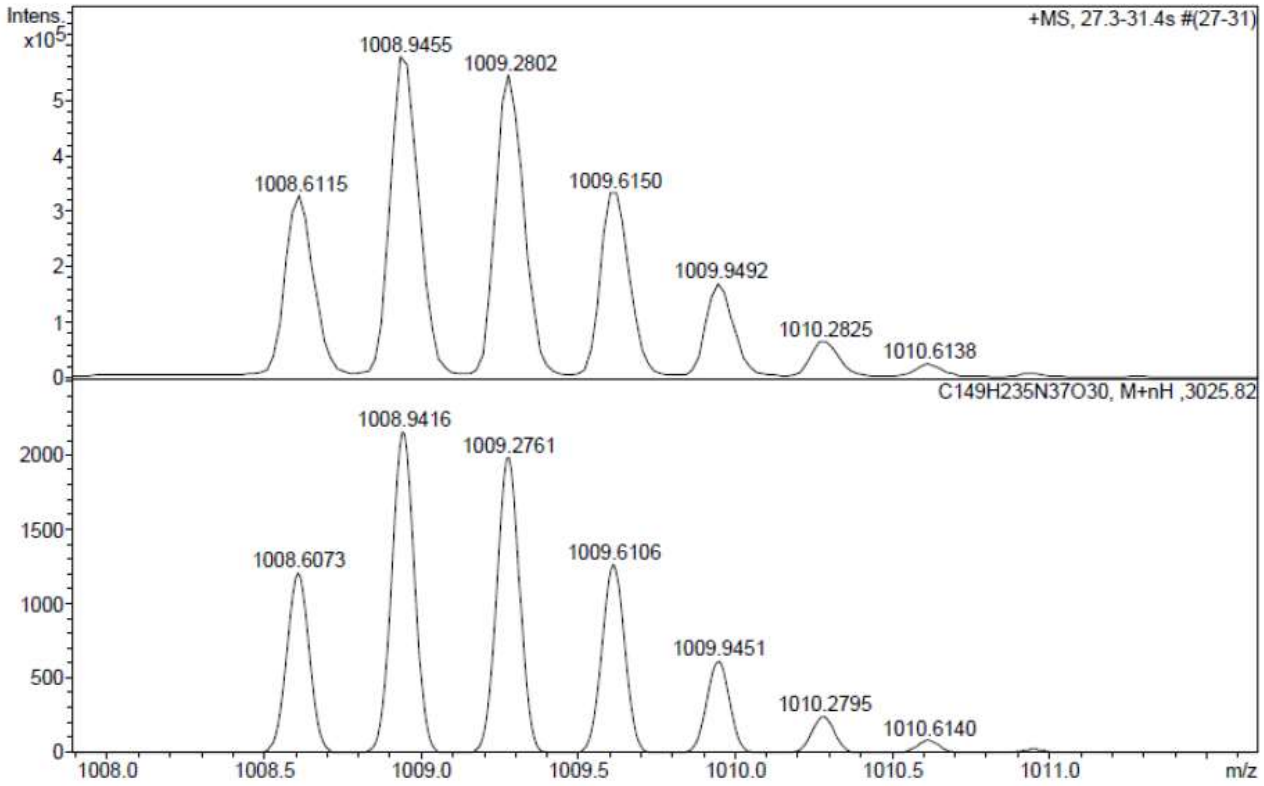
Intens. x10^5
+MS, 27.3-31.4s #(27-31)
1008.6115
1008.9455
1009.2802
1009.6150
1009.9492
1010.2825
1010.6138
C149H235N37O30, M+nH ,3025.82
1008.6073
1008.9416
1009.2761
1009.6106
1009.9451
1010.2795
1010.6140
m/z

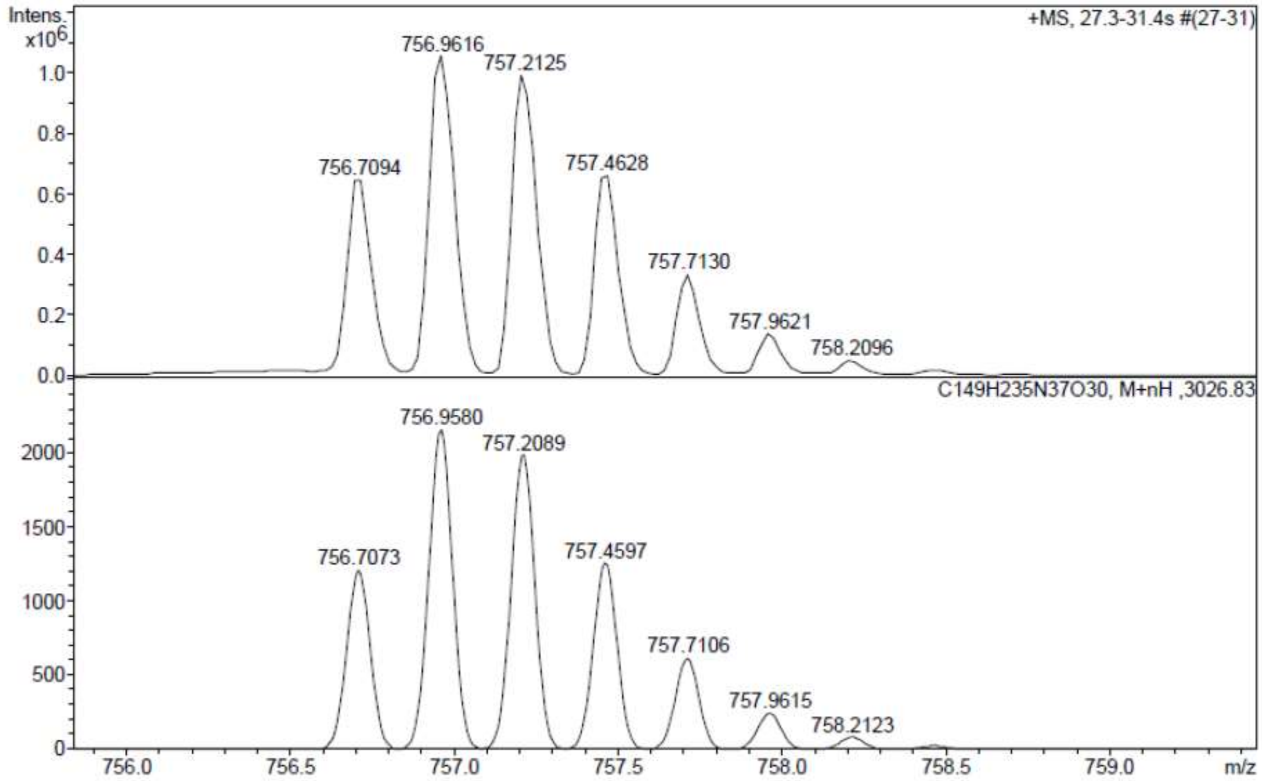
Intens.
x10^6
+MS, 27.3-31.4s #(27-31)
756.7094
756.9616
757.2125
757.4628
757.7130
757.9621
758.2096
C149H235N37O30, M+nH ,3026.83
756.7073
756.9580
757.2089
757.4597
757.7106
757.9615
758.2123
m/z

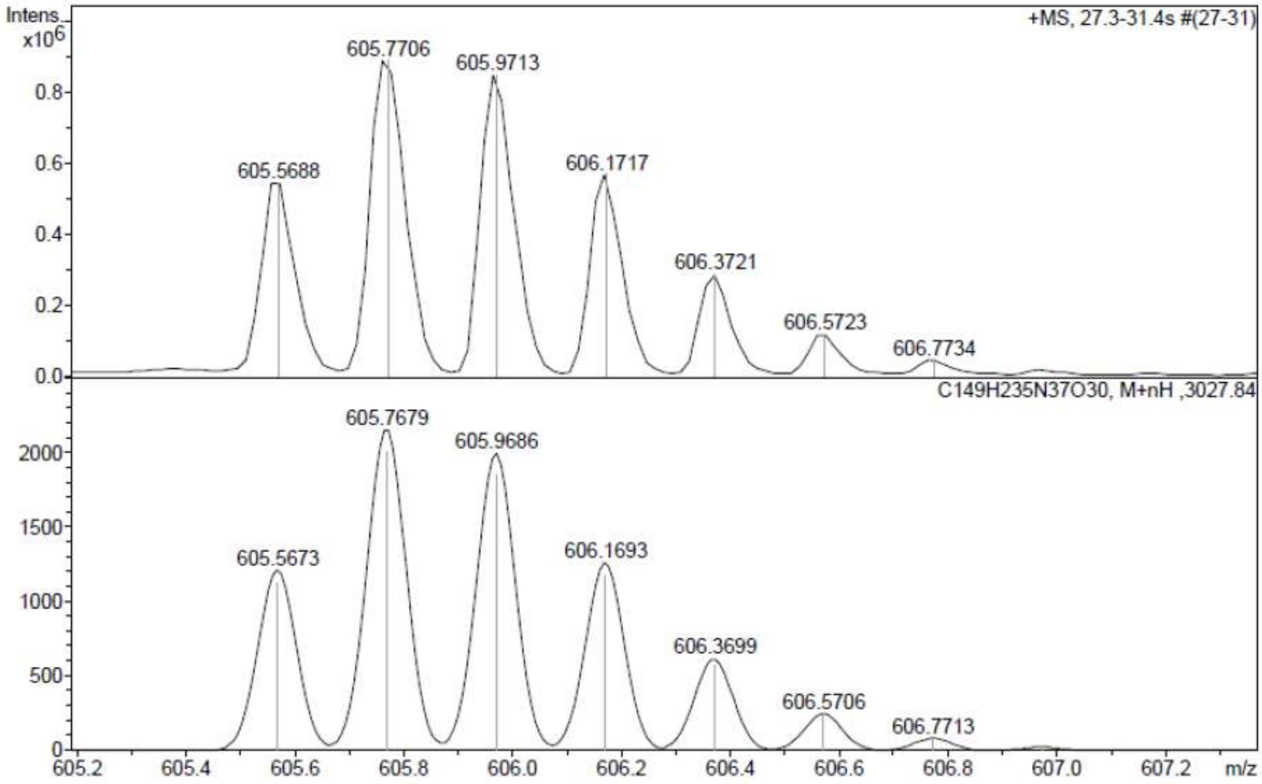
Intens.
x10^6
+MS, 27.3-31.4s #(27-31)
605.5688
605.7706
605.9713
606.1717
606.3721
606.5723
606.7734
C149H235N37O30, M+nH ,3027.84
605.5673
605.7679
605.9686
606.1693
606.3699
606.5706
606.7713
m/z

Lys-Lys-Leu-D-Phe-Lys-Lys-Ile-Leu-Lys-Tyr-Leu-Val-Trp-Asn-Gln-Pro-Val-Arg-Gly-Phe-Lys-Val-Tyr-Glu-OH (BP143-Pep13)

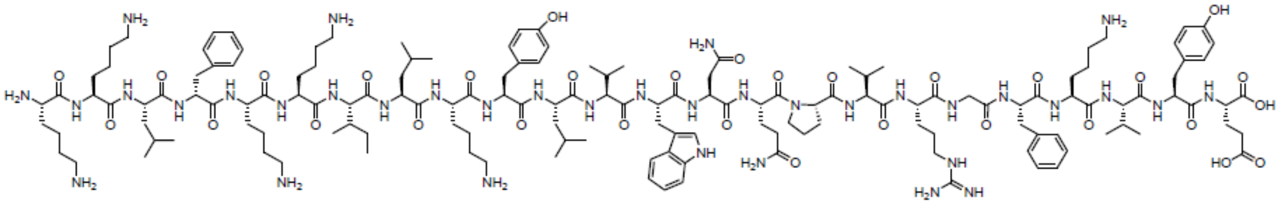

HPLC (λ=220 nm)

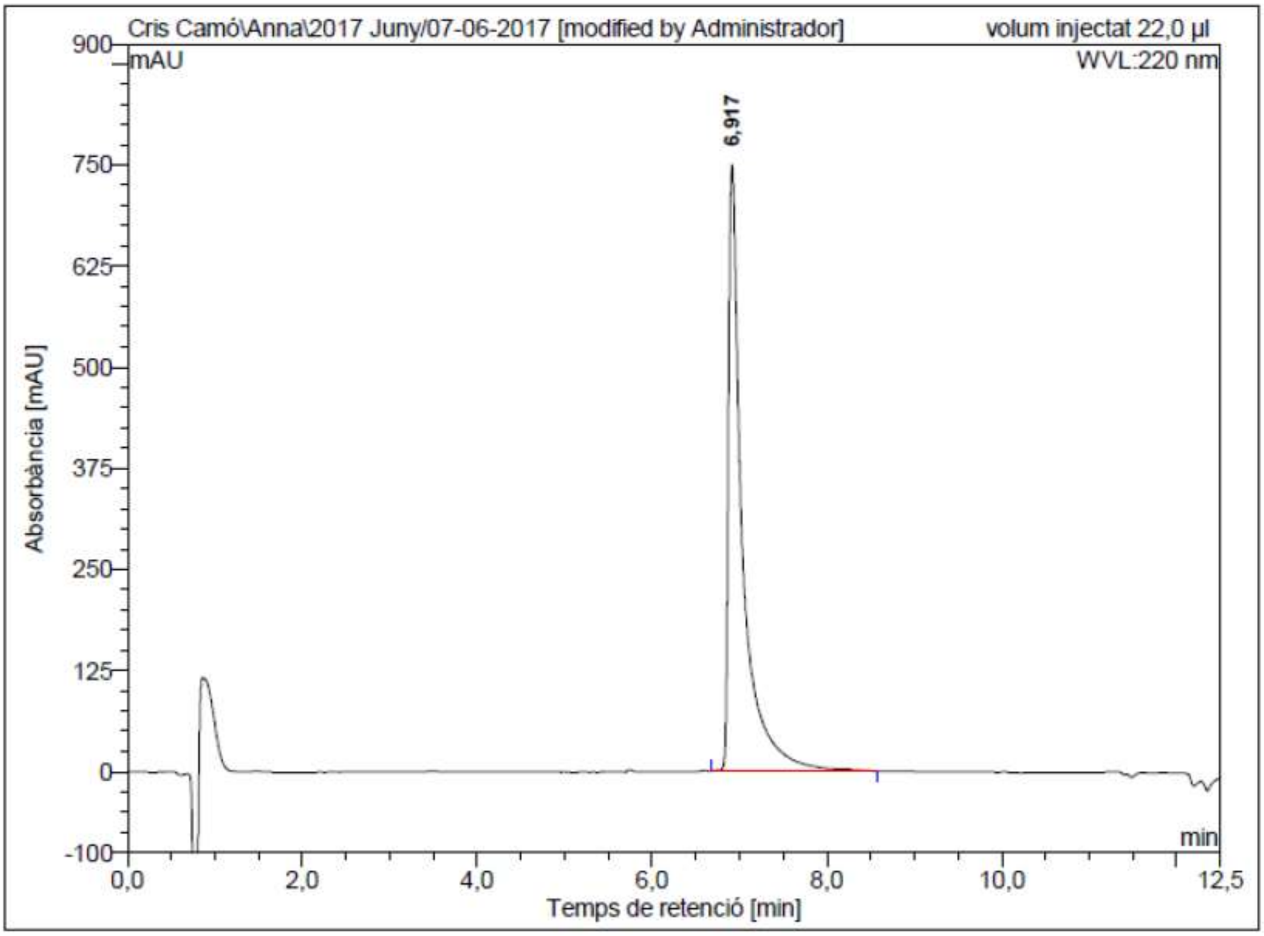

No.	Temps retenció min	alçada mAU	Area mAU*min	Area relativa %
1	6,92	749,433	139,866	100,00
Total:		749,433	139,866	100,00

ESI-MS (*m/z*)

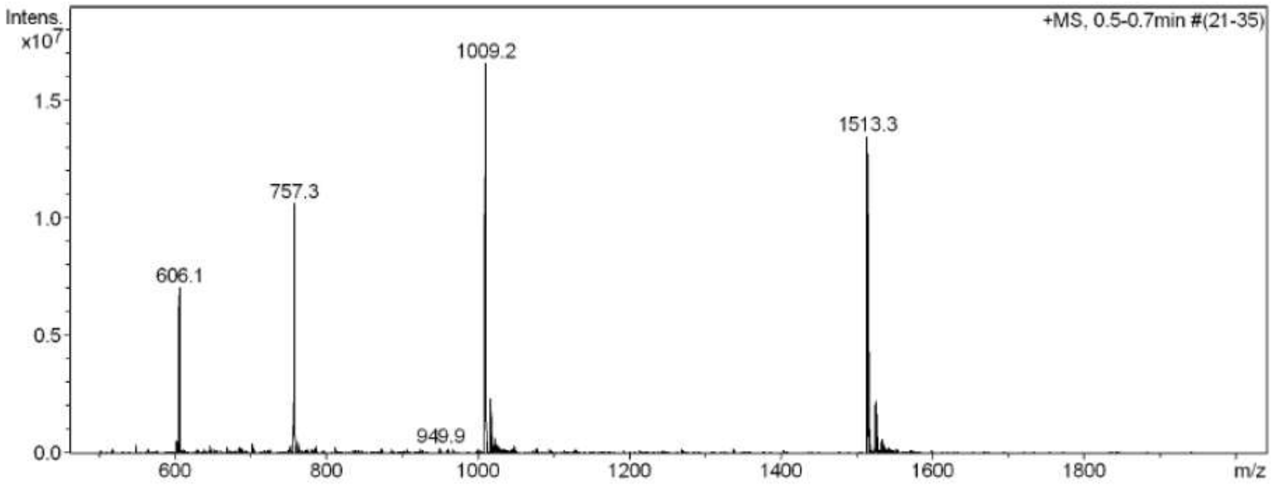

HRMS (*m/z*)

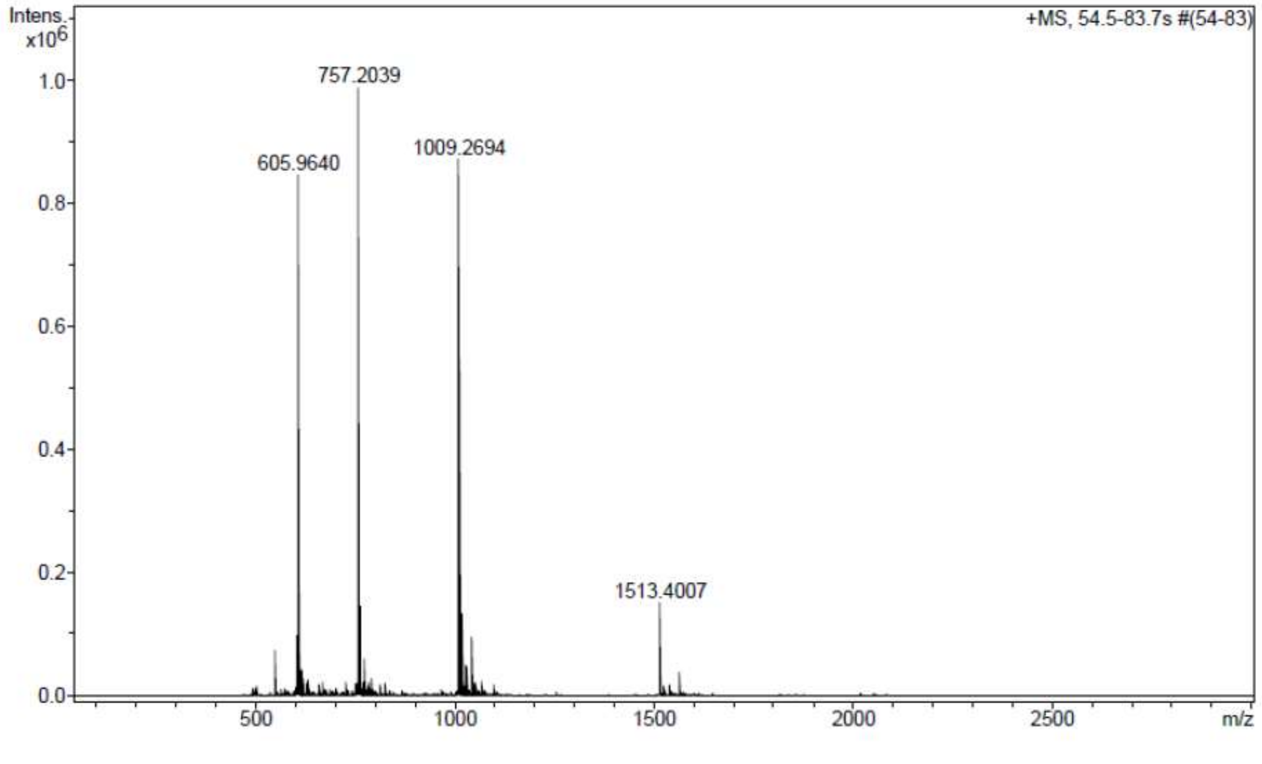

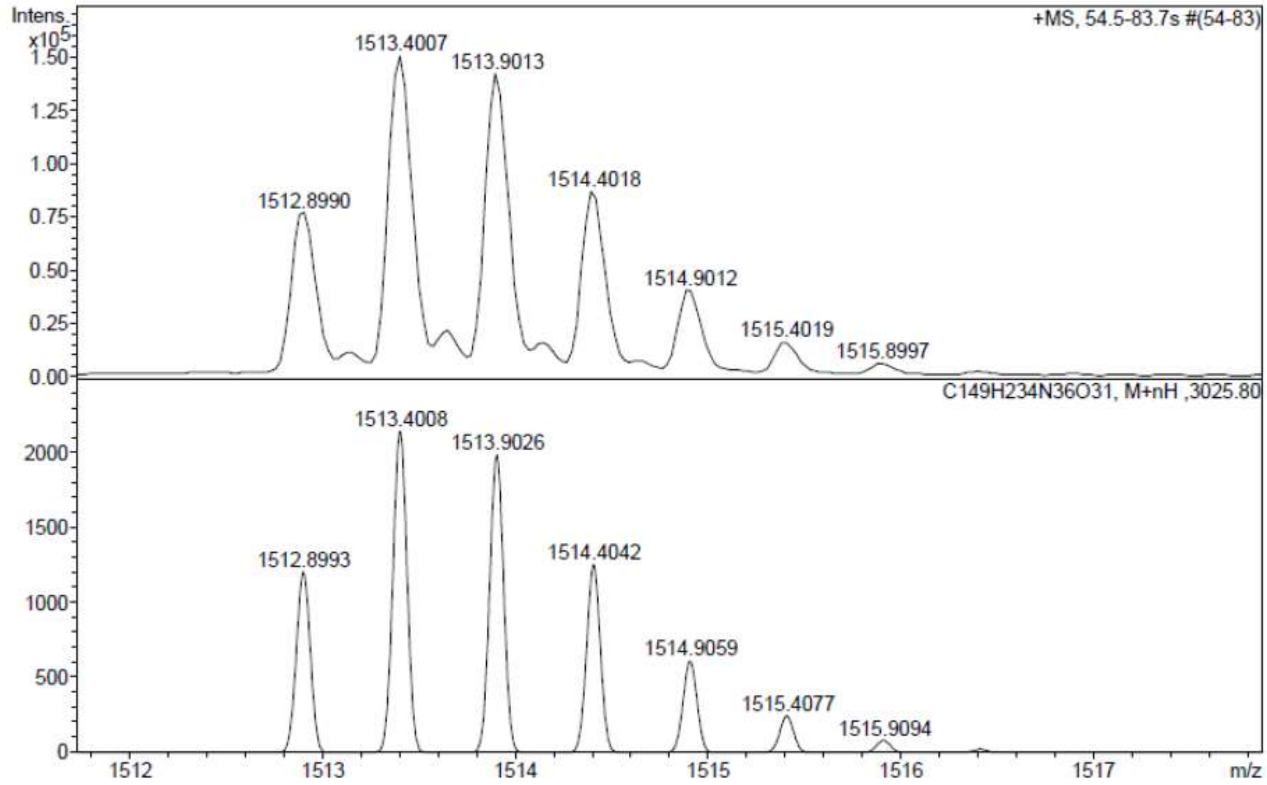

Intens. x10^5
+MS, 54.5-83.7s #(54-83)
1513.4007
1513.9013
1514.4018
1512.8990
1514.9012
1515.4019
1515.8997
C149H234N36O31, M+nH ,3025.80
1513.4008
1513.9026
1514.4042
1512.8993
1514.9059
1515.4077
1515.9094
m/z

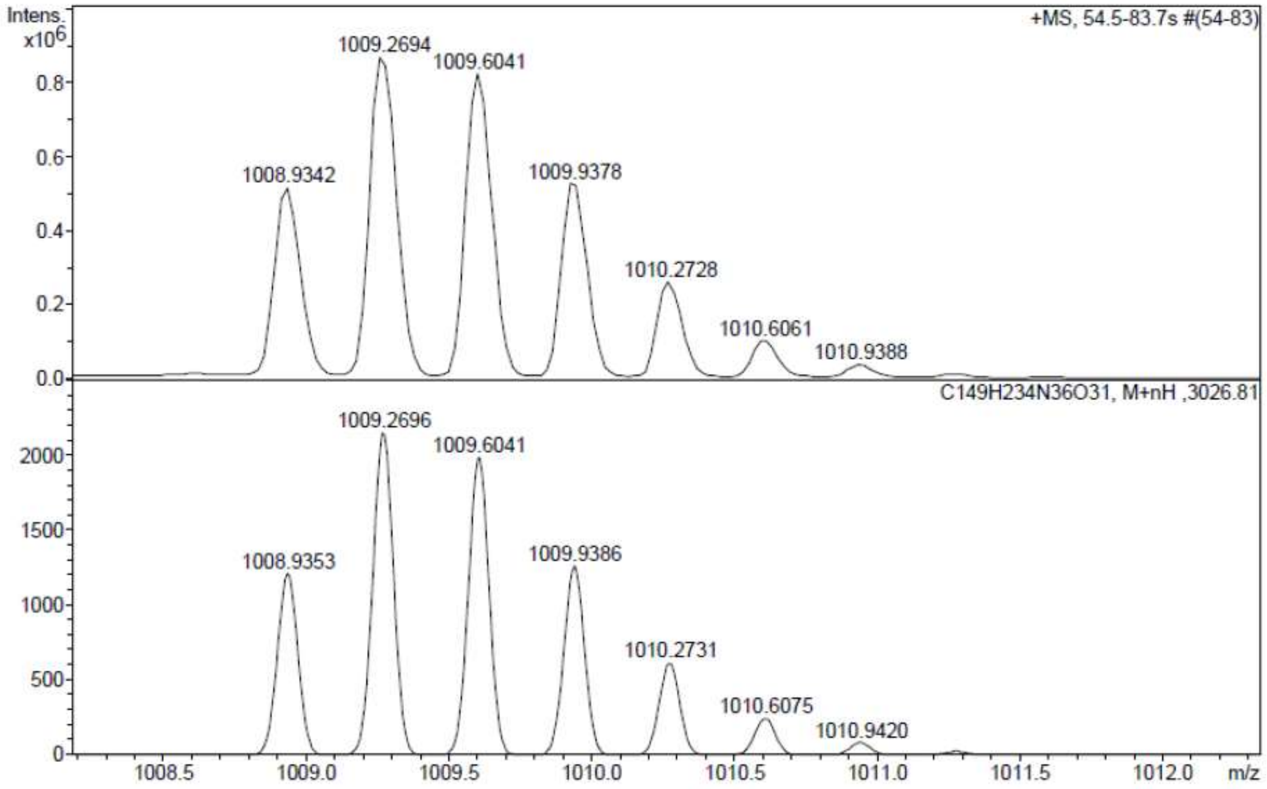

Intens. x10^6
+MS, 54.5-83.7s #(54-83)
1009.2694
1009.6041
1008.9342
1009.9378
1010.2728
1010.6061
1010.9388
C149H234N36O31, M+nH ,3026.81
1009.2696
1009.6041
1008.9353
1009.9386
1010.2731
1010.6075
1010.9420
m/z

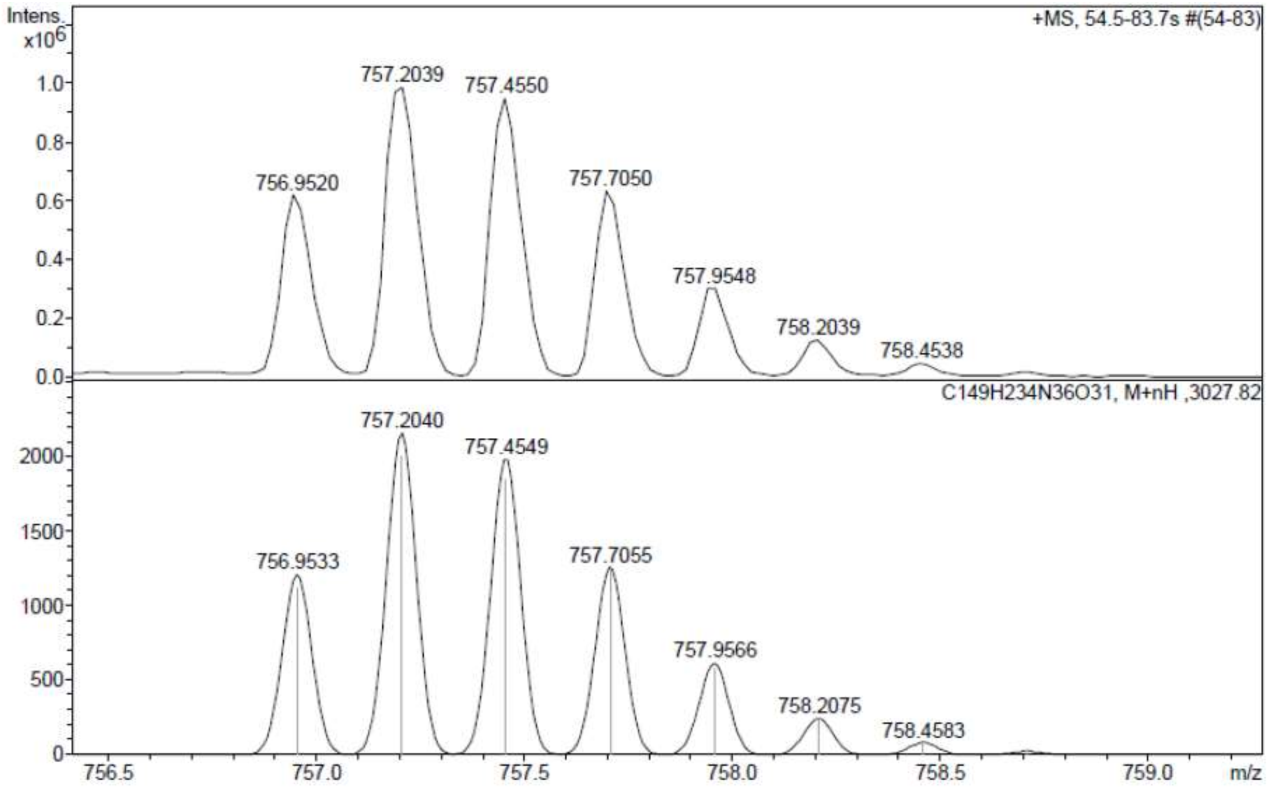
Intens.
x10^6
+MS, 54.5-83.7s #(54-83)
756.9520
757.2039
757.4550
757.7050
757.9548
758.2039
758.4538
C149H234N36O31, M+nH ,3027.82
756.9533
757.2040
757.4549
757.7055
757.9566
758.2075
758.4583
m/z

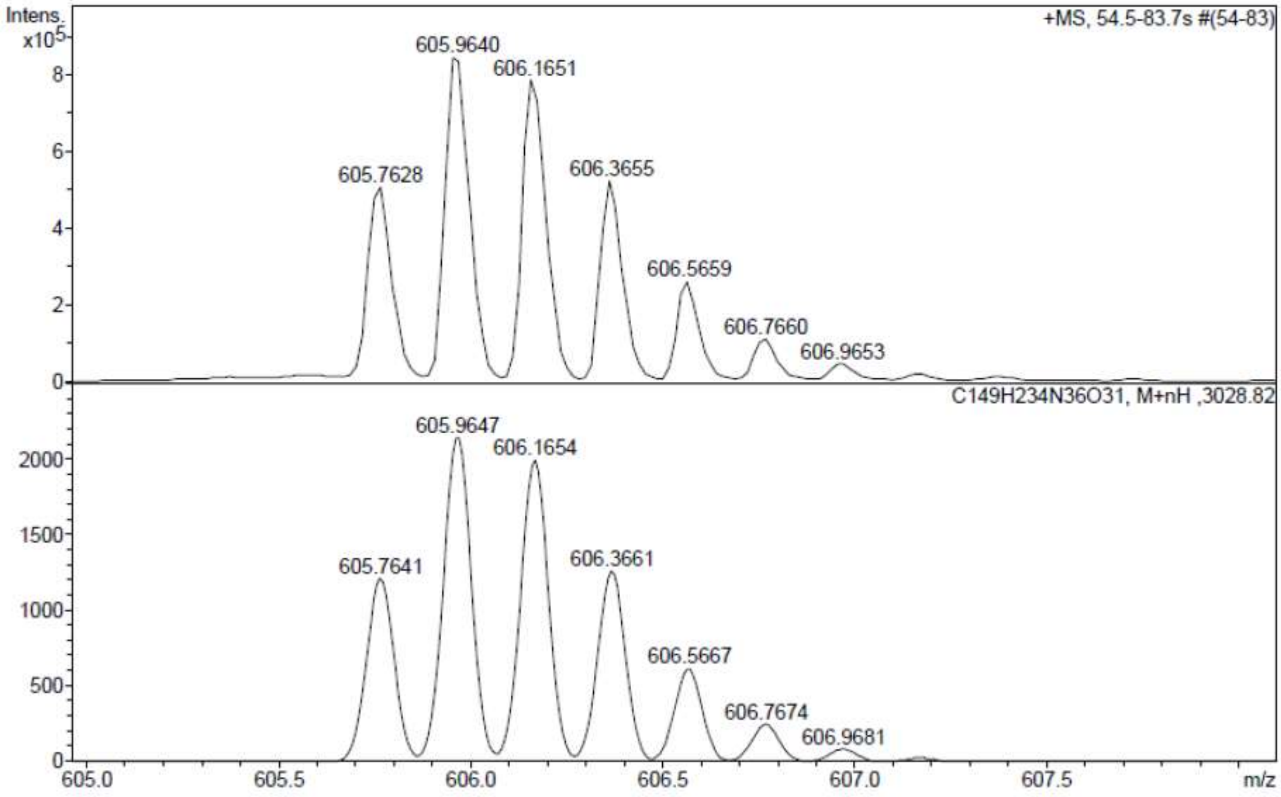
Intens.
x10^5
+MS, 54.5-83.7s #(54-83)
605.7628
605.9640
606.1651
606.3655
606.5659
606.7660
606.9653
C149H234N36O31, M+nH ,3028.82
605.7641
605.9647
606.1654
606.3661
606.5667
606.7674
606.9681
m/z

Val-Trp-Asn-Gln-Pro-Val-Arg-Gly-Phe-Lys-Val-Tyr-Glu-Lys-Lys-Val-Val-Phe-Trp-Val-Lys-Phe-Lys-NH_2 (Pep13-KSLW)

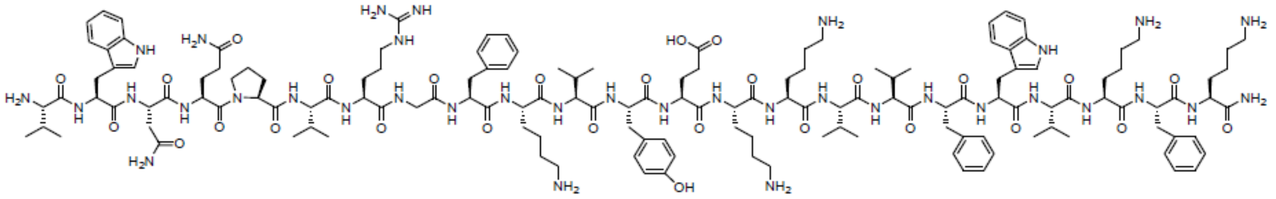

HPLC (λ=220 nm)

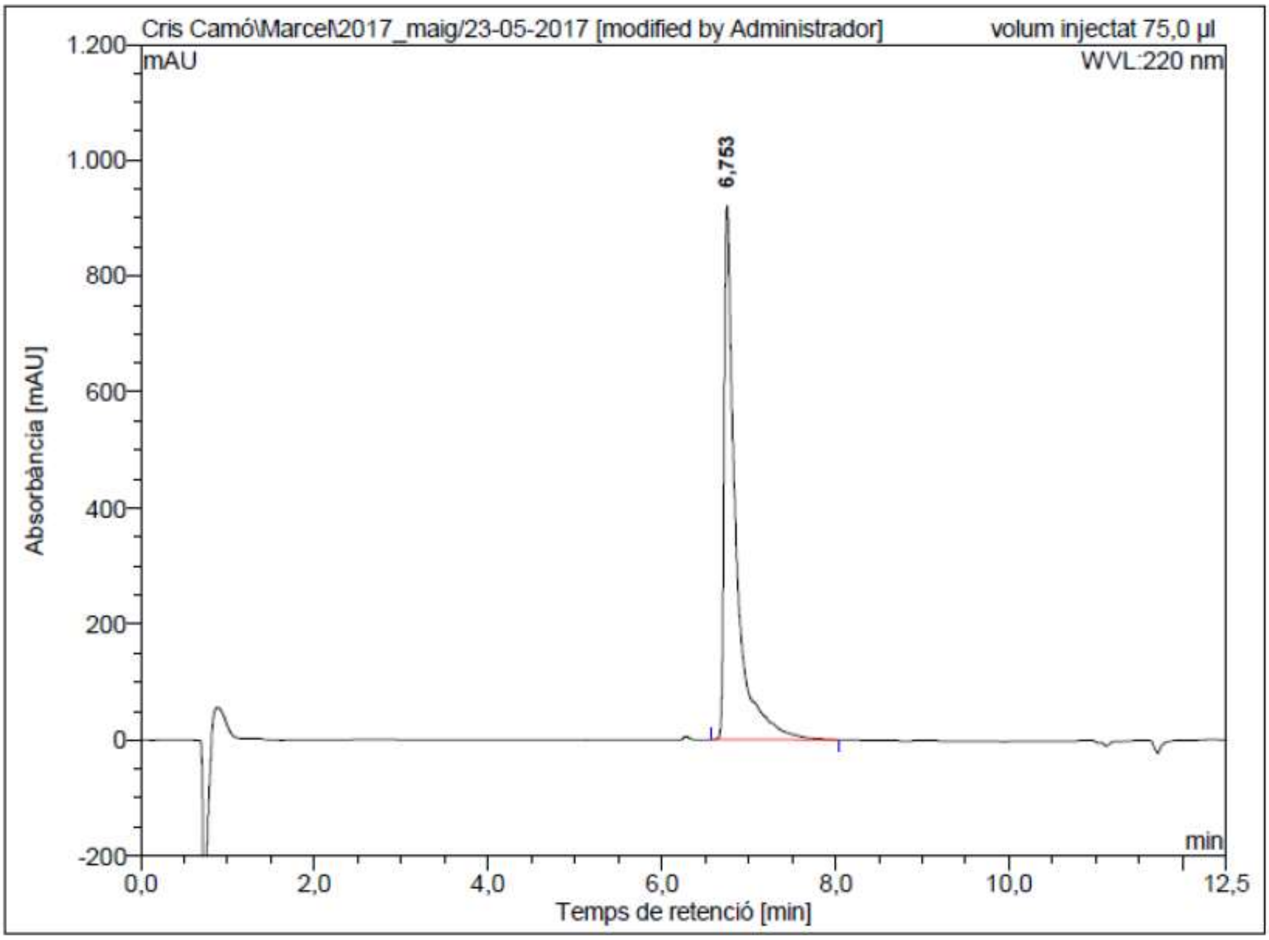

No.	Temps retenció min	alçada mAU	Area mAU*min	Area relativa %
1	6,75	920,149	143,001	100,00
Total:		920,149	143,001	100,00

ESI-MS (*m/z*)

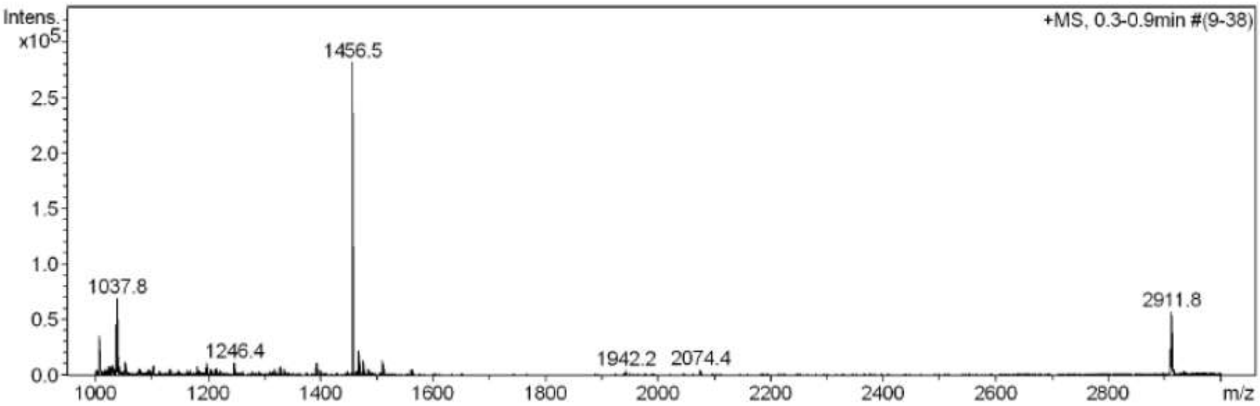

HRMS (*m/z*)

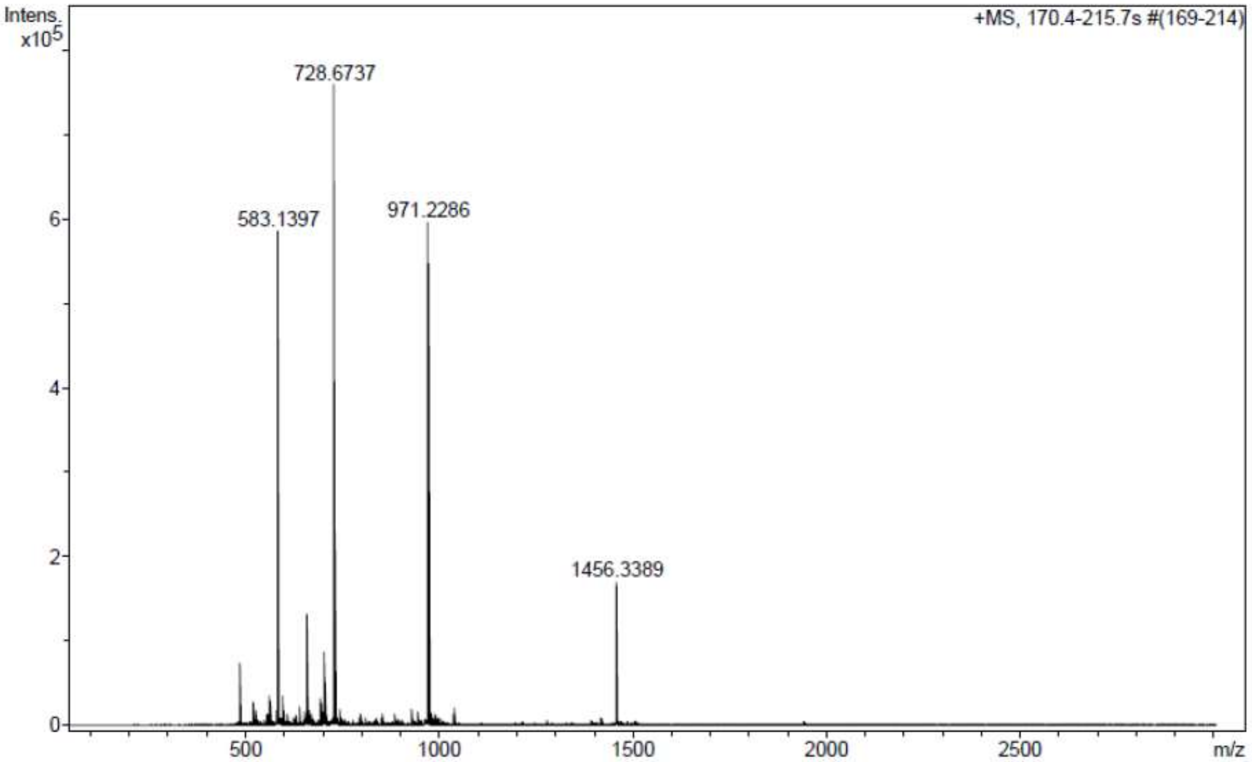

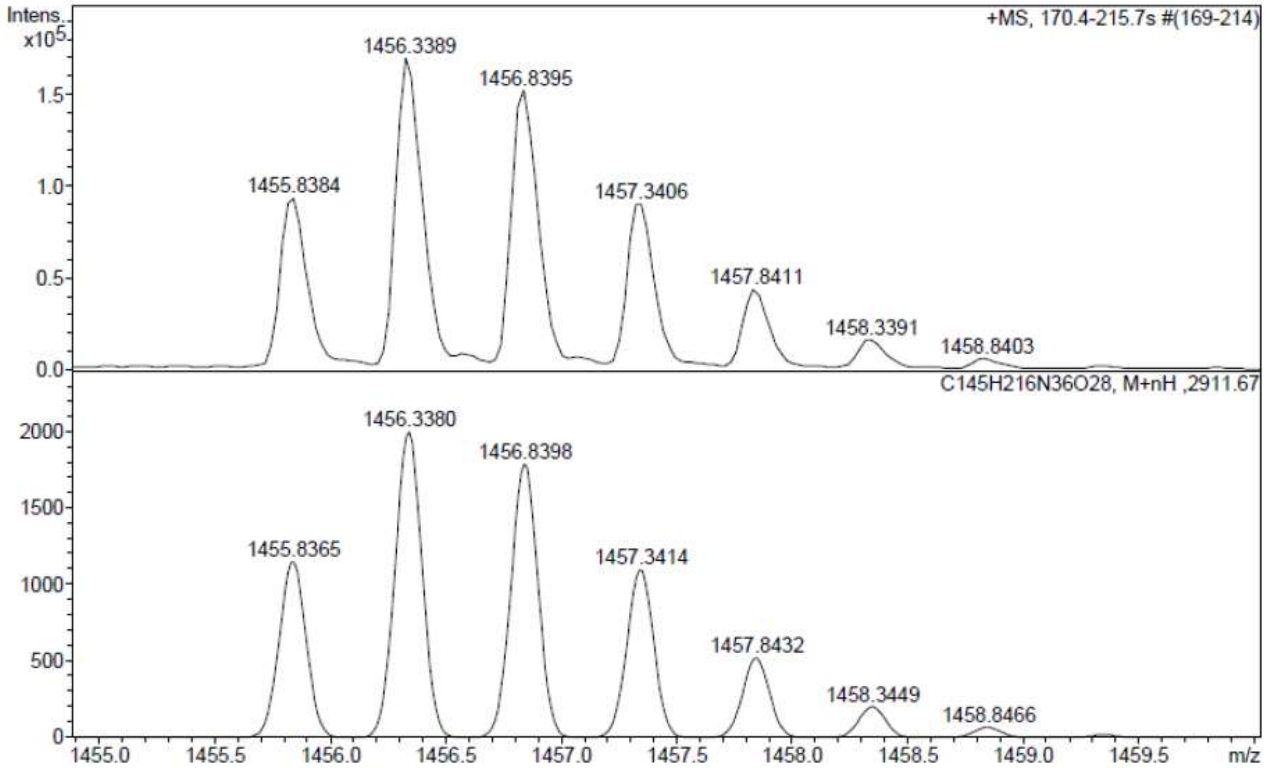
Intens.
x10^5
+MS, 170.4-215.7s #(169-214)
1456.3389
1456.8395
1455.8384
1457.3406
1457.8411
1458.3391
1458.8403
C145H216N36O28, M+nH ,2911.67
1456.3380
1456.8398
1455.8365
1457.3414
1457.8432
1458.3449
1458.8466
m/z

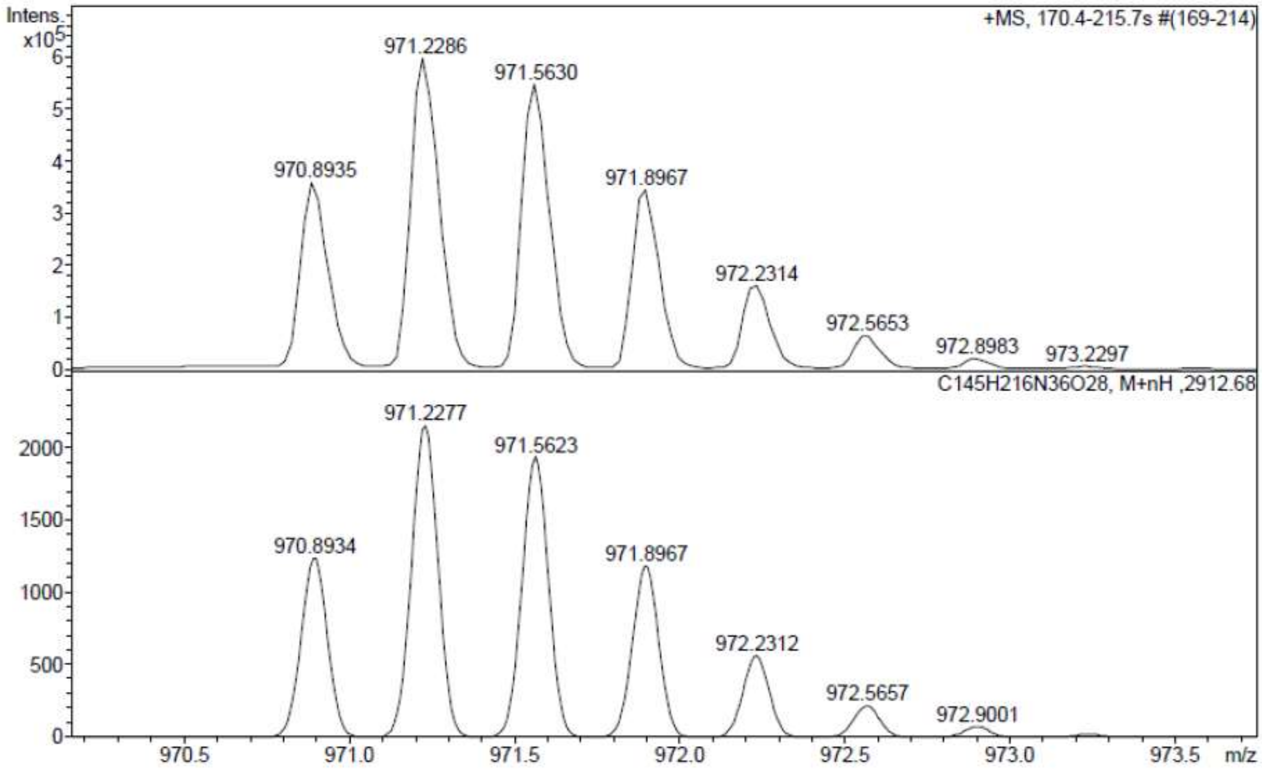
Intens.
x10^5
+MS, 170.4-215.7s #(169-214)
971.2286
971.5630
970.8935
971.8967
972.2314
972.5653
972.8983
973.2297
C145H216N36O28, M+nH ,2912.68
971.2277
971.5623
970.8934
971.8967
972.2312
972.5657
972.9001
m/z

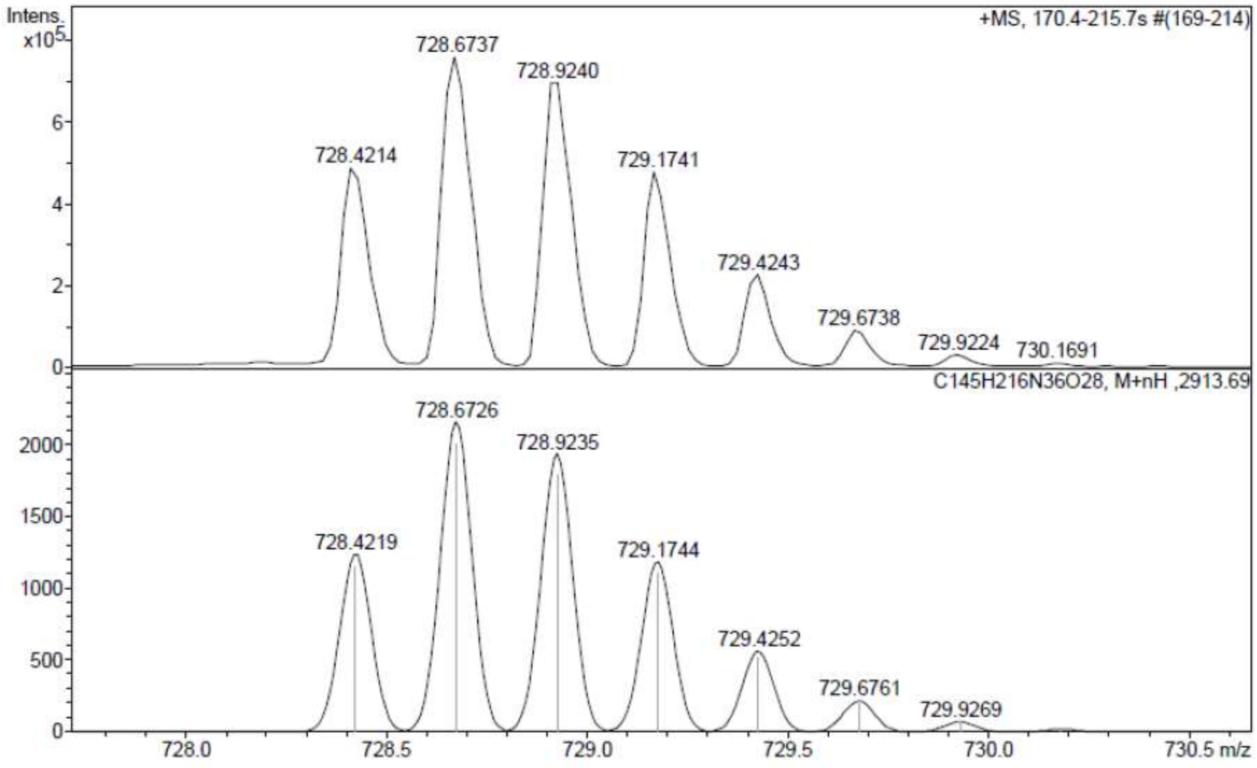
Intens.
x10^5
+MS, 170.4-215.7s #(169-214)
728.6737
728.9240
728.4214
729.1741
729.4243
729.6738
729.9224
730.1691
C145H216N36O28, M+nH ,2913.69
728.6726
728.9235
728.4219
729.1744
729.4252
729.6761
729.9269
728.0
728.5
729.0
729.5
730.0
730.5 m/z

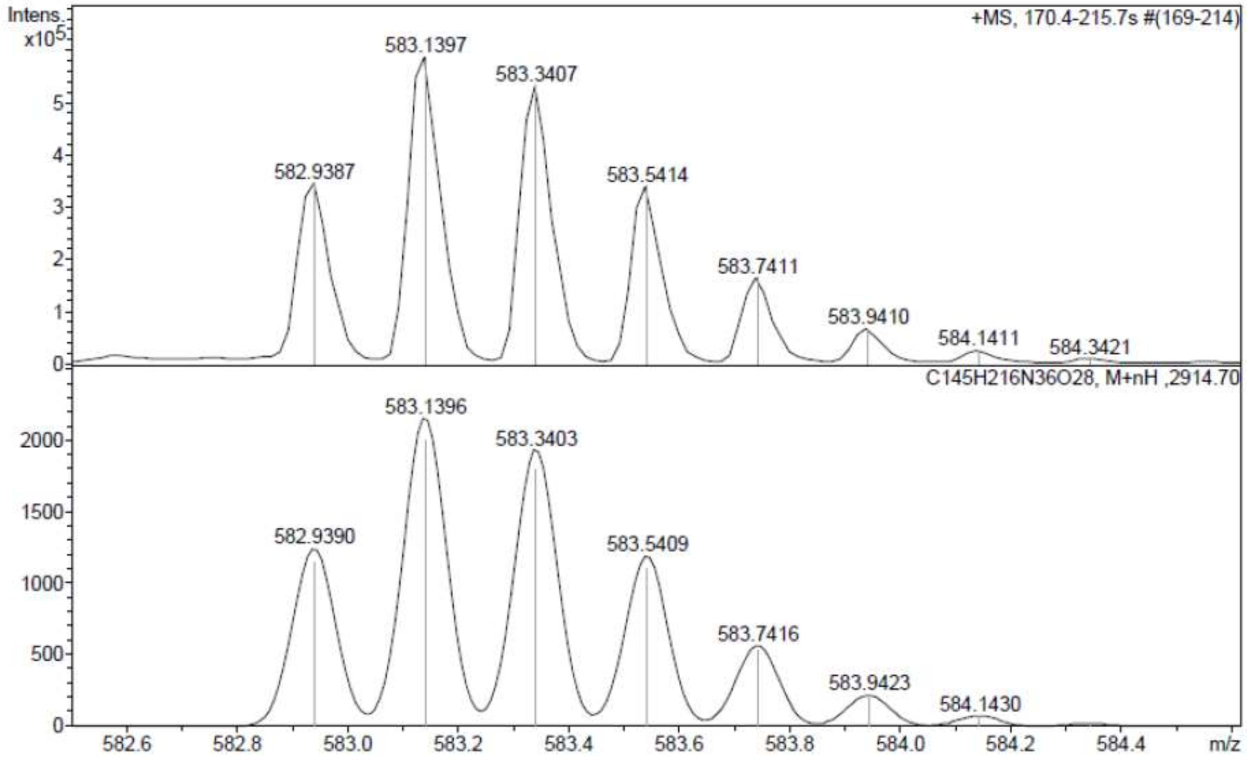
Intens.
x10^5
+MS, 170.4-215.7s #(169-214)
583.1397
583.3407
582.9387
583.5414
583.7411
583.9410
584.1411
584.3421
C145H216N36O28, M+nH ,2914.70
583.1396
583.3403
582.9390
583.5409
583.7416
583.9423
584.1430
582.6
582.8
583.0
583.2
583.4
583.6
583.8
584.0
584.2
584.4
m/z

Lys-Lys-Val-Val-Phe-Trp-Val-Lys-Phe-Lys-Val-Trp-Asn-Gln-Pro-Val-Arg-Gly-Phe-Lys-Val-Tyr-Glu-OH (KSLW-Pep13)

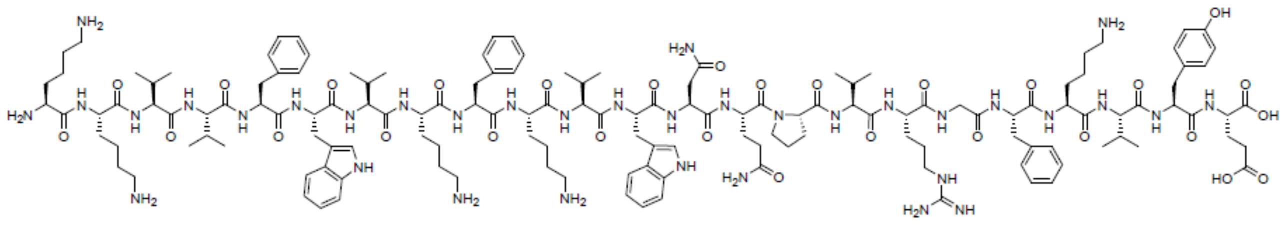

HPLC (λ=220 nm)

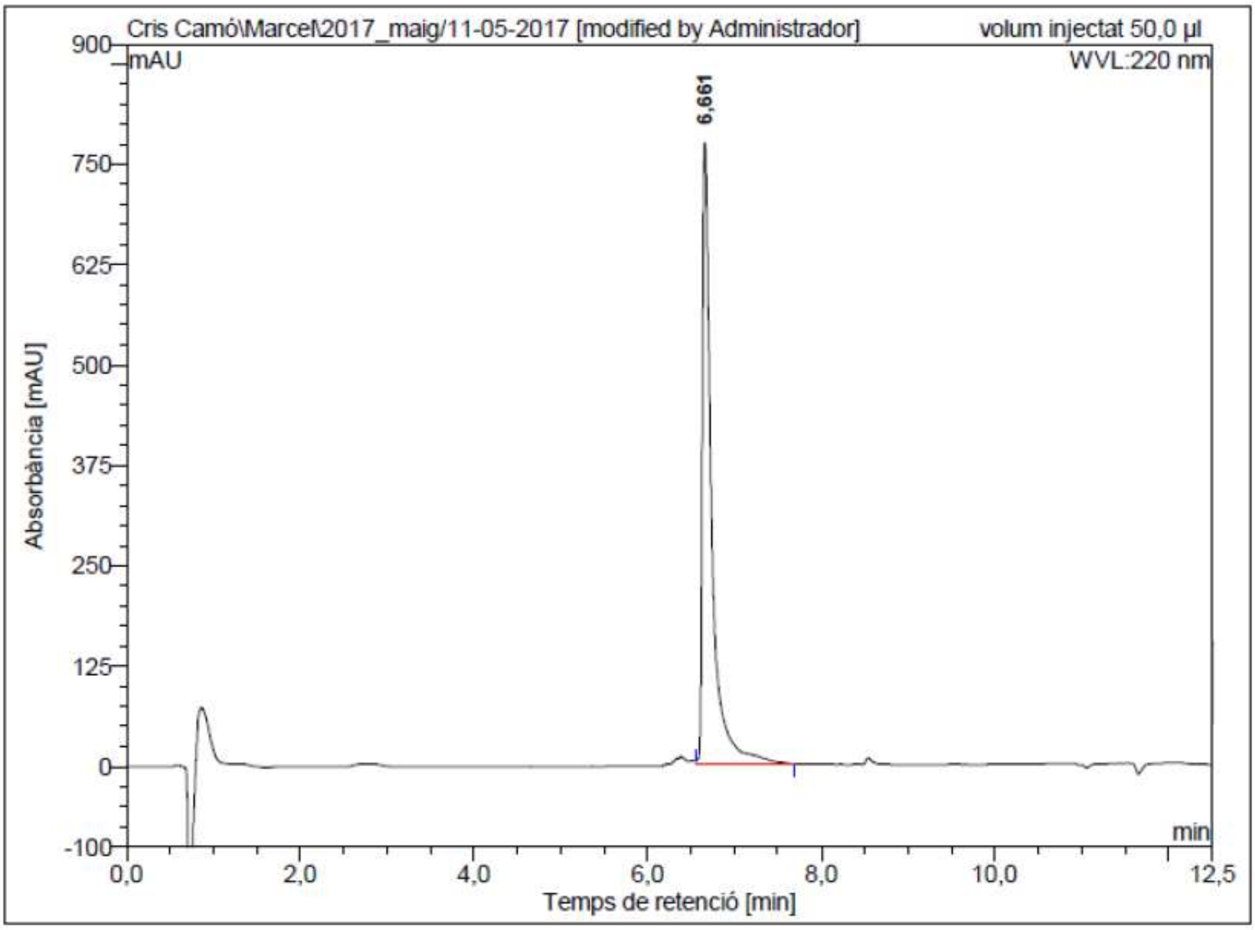

No.	Temps retenció<br>min	alçada<br>mAU	Area<br>mAU*min	Area relativa<br>%
1	6,66	774,041	97,191	100,00
Total:		774,041	97,191	100,00

ESI-MS (*m/z*)

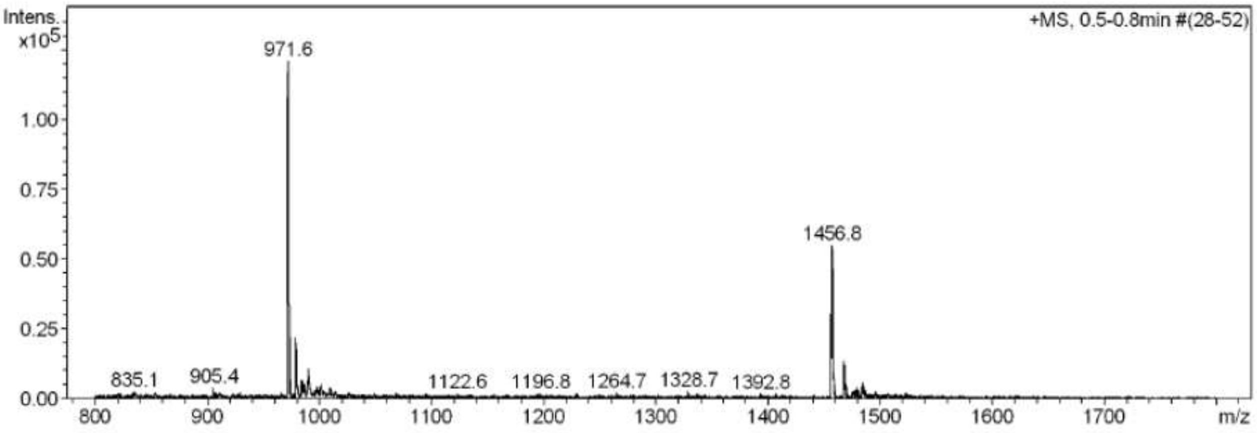

HRMS (*m/z*)

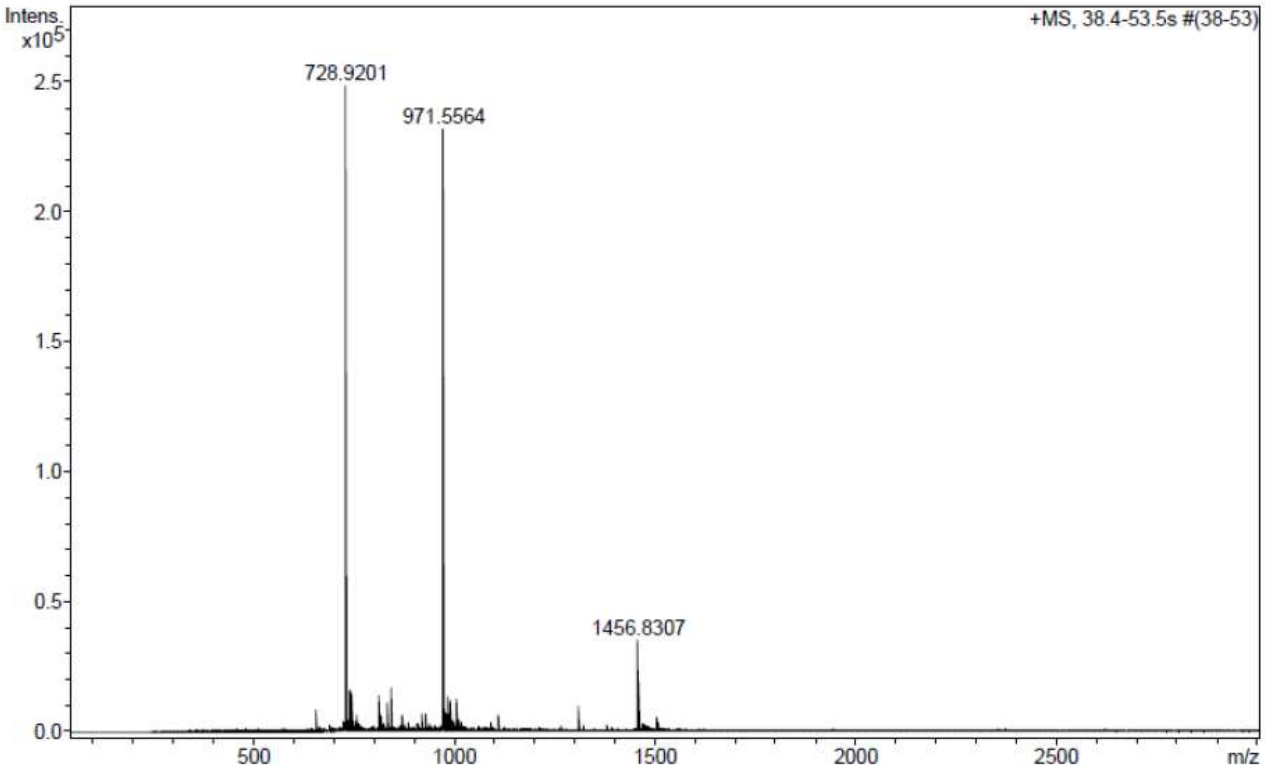

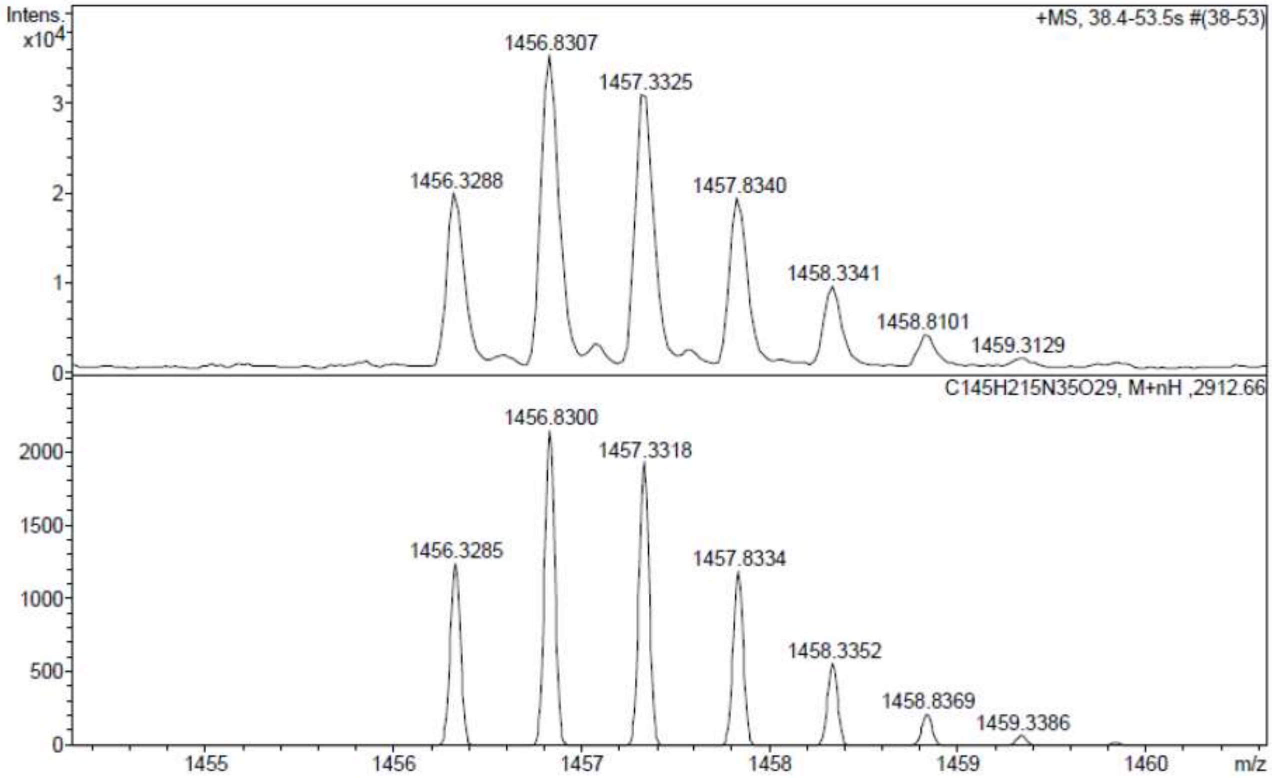
Intens. x10^4
+MS, 38.4-53.5s #(38-53)
1456.8307
1457.3325
1456.3288
1457.8340
1458.3341
1458.8101
1459.3129
C145H215N35O29, M+nH ,2912.66
1456.8300
1457.3318
1456.3285
1457.8334
1458.3352
1458.8369
1459.3386
m/z

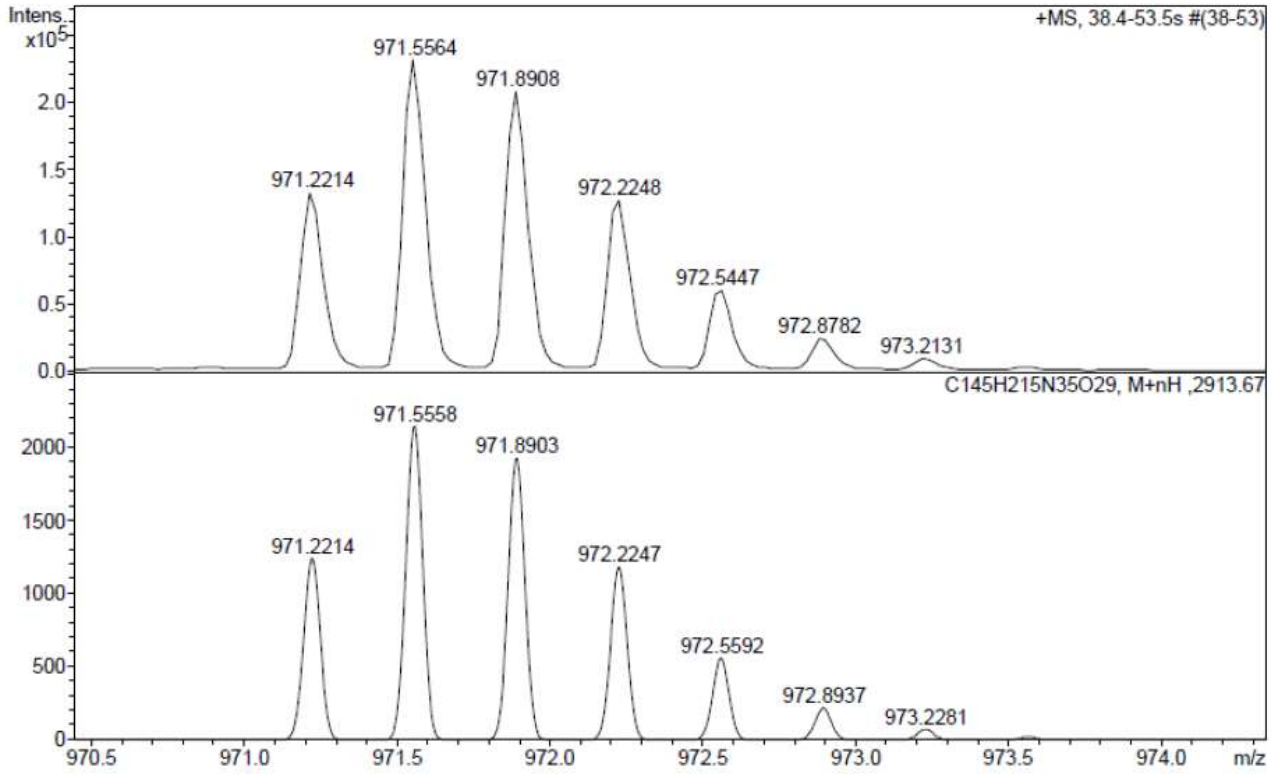
Intens. x10^5
+MS, 38.4-53.5s #(38-53)
971.5564
971.8908
971.2214
972.2248
972.5447
972.8782
973.2131
C145H215N35O29, M+nH ,2913.67
971.5558
971.8903
971.2214
972.2247
972.5592
972.8937
973.2281
m/z

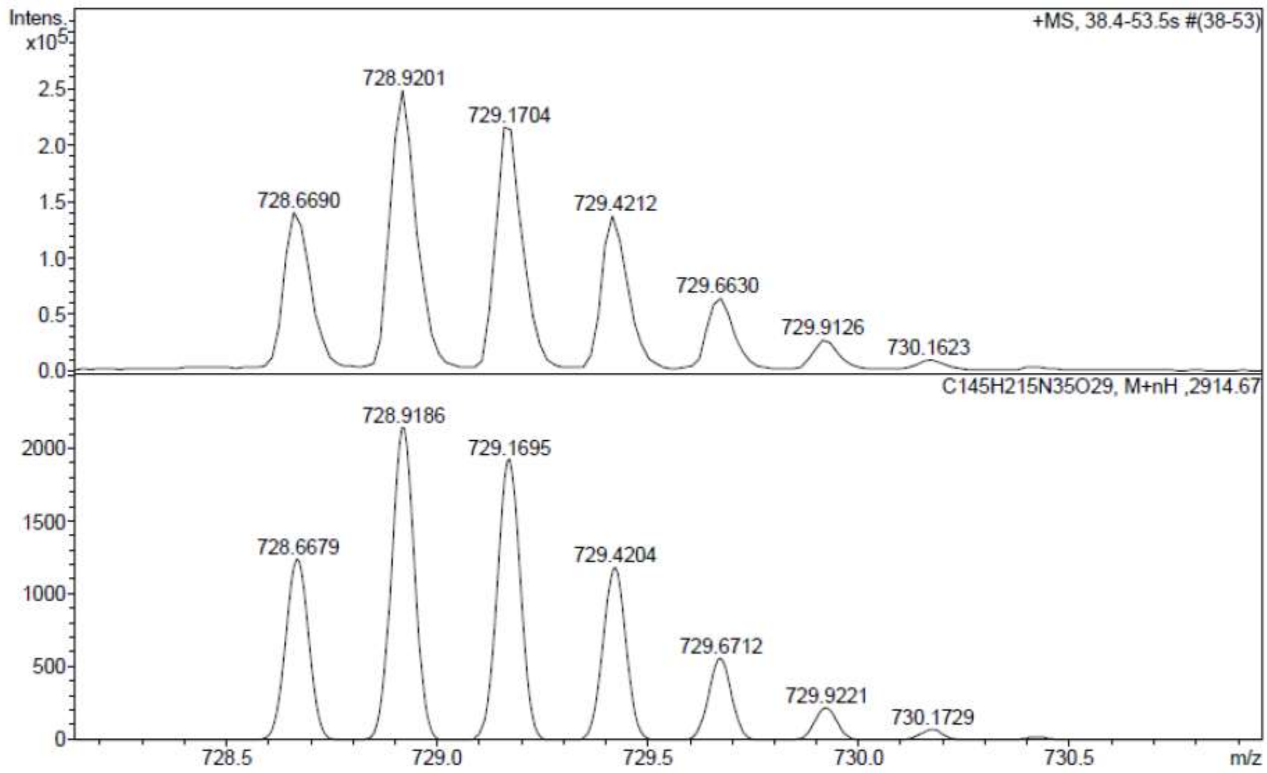
Intens.
x10^5
+MS, 38.4-53.5s #(38-53)
728.6690
728.9201
729.1704
729.4212
729.6630
729.9126
730.1623
C145H215N35O29, M+nH ,2914.67
728.6679
728.9186
729.1695
729.4204
729.6712
729.9221
730.1729
m/z

Ac-Tyr-Gly-Ile-His-Thr-His-Lys-Lys-Leu-D-Phe-Lys-Lys-Ile-Leu-Lys-Lys(COC_3H_7)-Leu-NH_2 (PIP1-BP475)

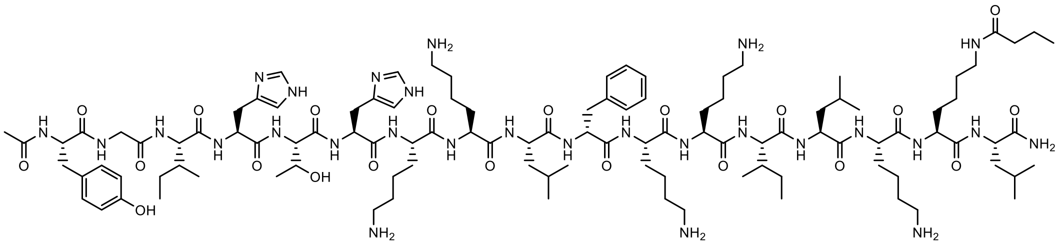

HPLC (λ=220 nm)

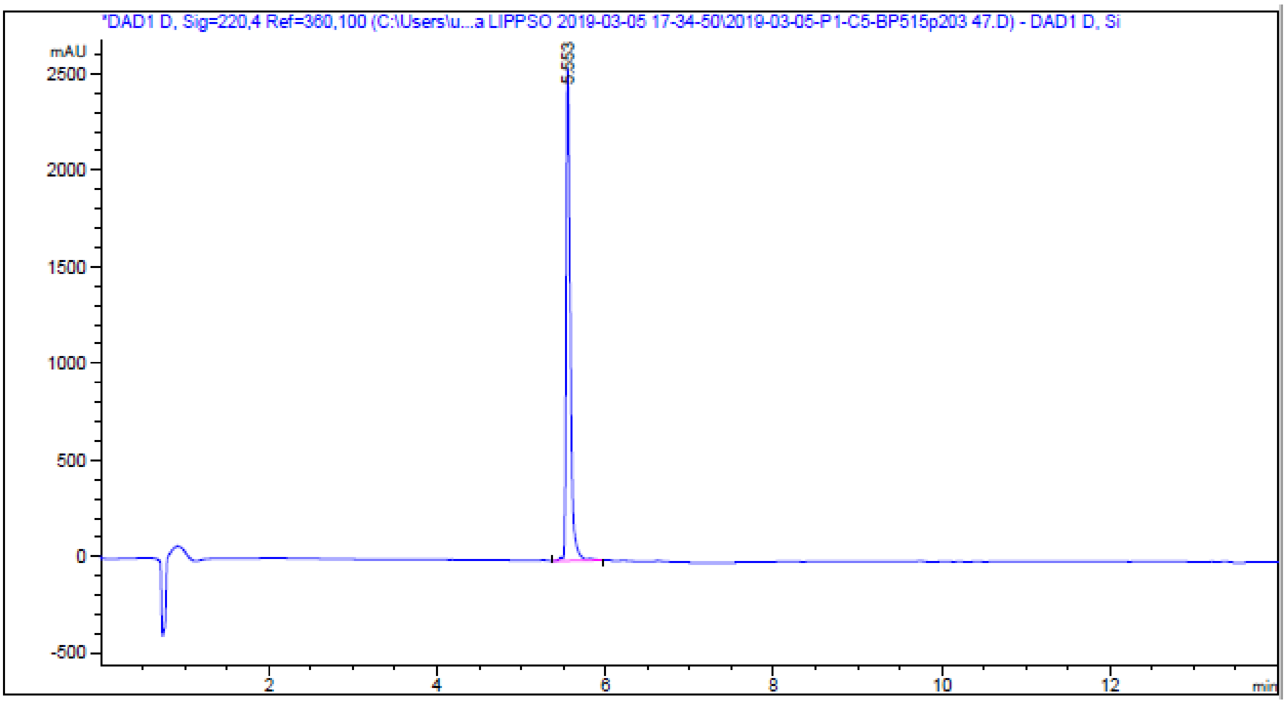

Peak #	RetTime [min]	Type	Width [min]	Area [mAU*s]	Height [mAU]	Area %
1	5.553	VV R	0.0551	9212.02441	2550.51538	100.0000

Totals : 9212.02441 2550.51538

ESI-MS (*m/z*)

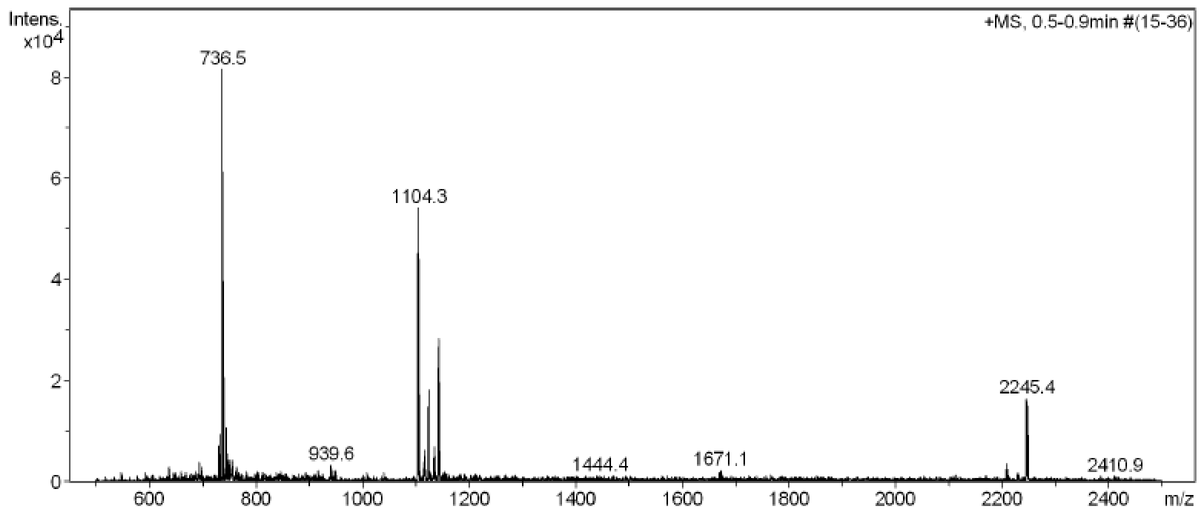

HRMS (*m/z*)

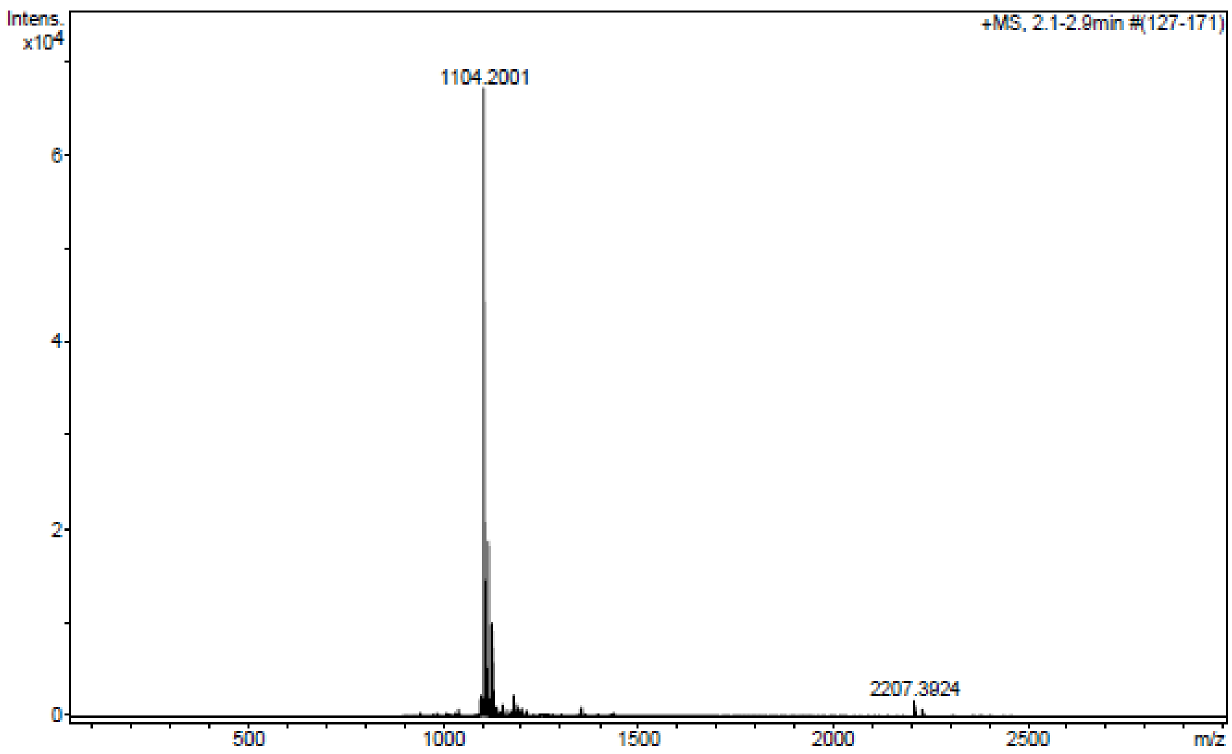

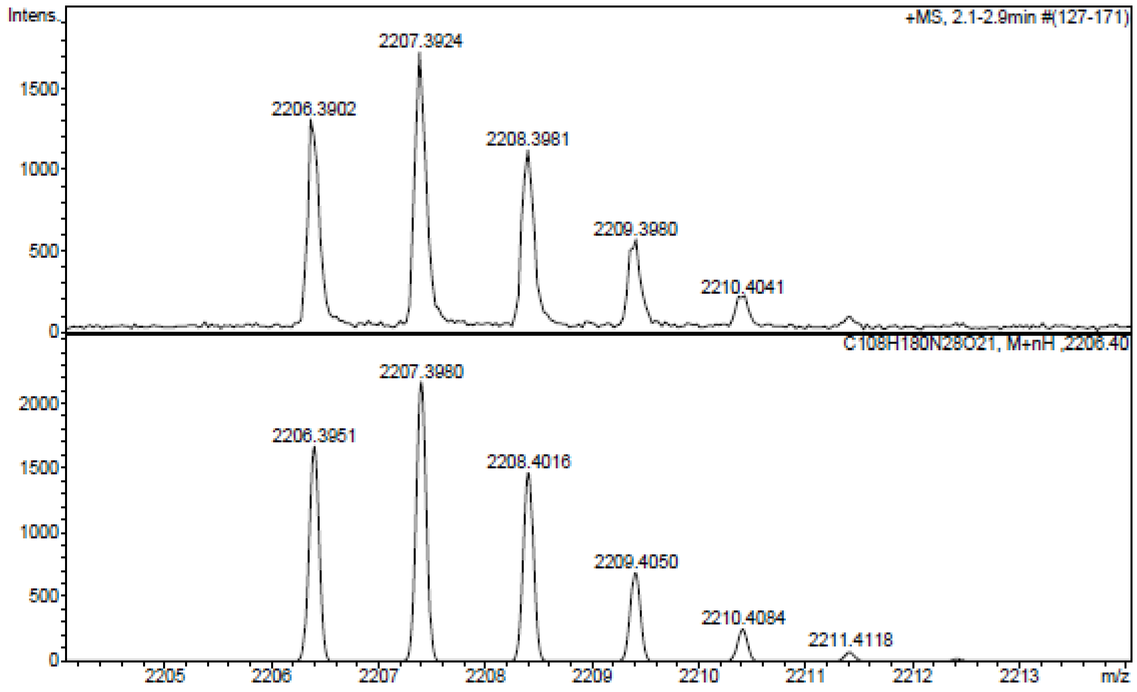
Intens.
+MS, 2.1-2.9min #(127-171)
2207.3924
2206.3902
2208.3981
2209.3980
2210.4041
C108H180N28O21, M+nH ,2206.40
2207.3980
2206.3951
2208.4016
2209.4050
2210.4084
2211.4118
m/z

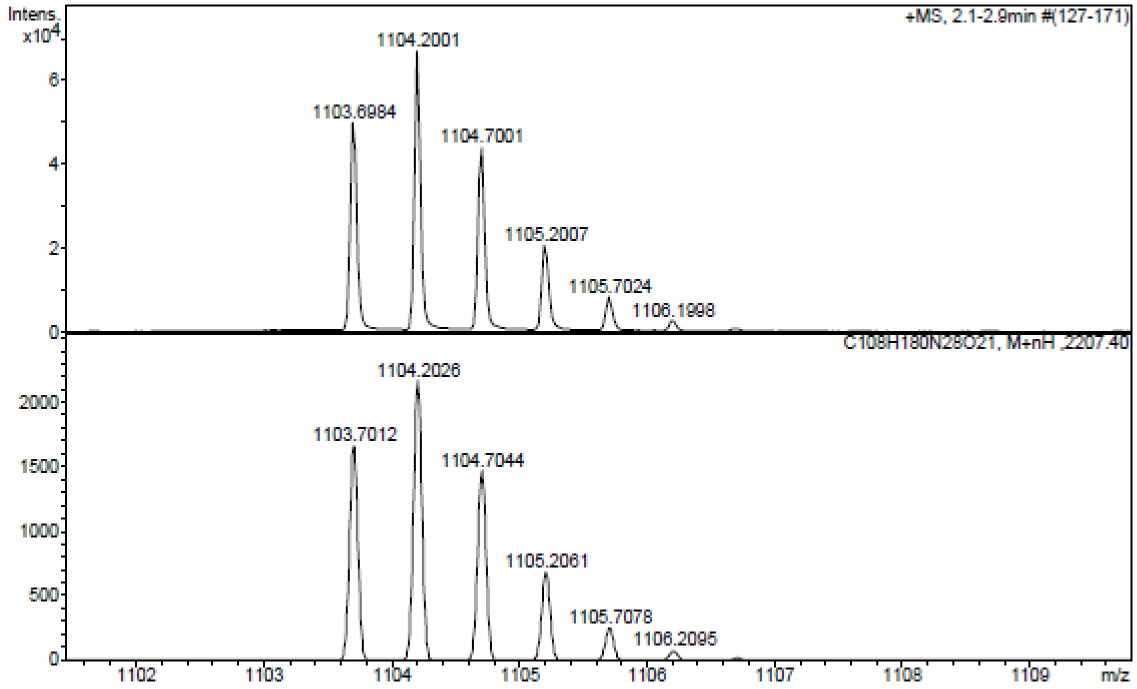
Intens.
x10^4
+MS, 2.1-2.9min #(127-171)
1104.2001
1103.6984
1104.7001
1105.2007
1105.7024
1106.1998
C108H180N28O21, M+nH ,2207.40
1104.2026
1103.7012
1104.7044
1105.2061
1105.7078
1106.2095
m/z

Ac-Lys-Lys-Leu-D-Phe-Lys-Lys-Ile-Leu-Lys-Lys(COC_3H_7)-Leu-Tyr-Gly-Ile-His-Thr-His-NH_2 (BP475-PIP1)

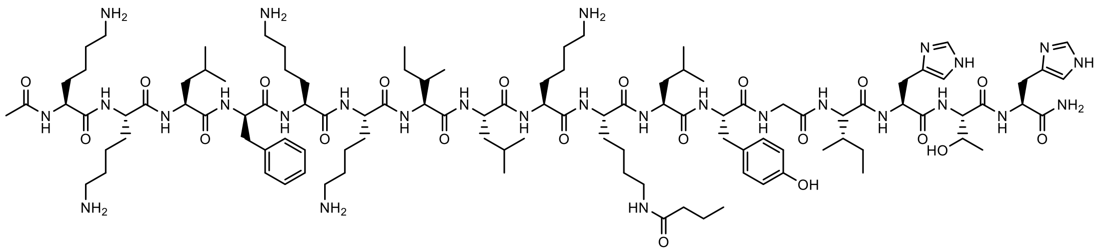

HPLC (λ=220 nm)

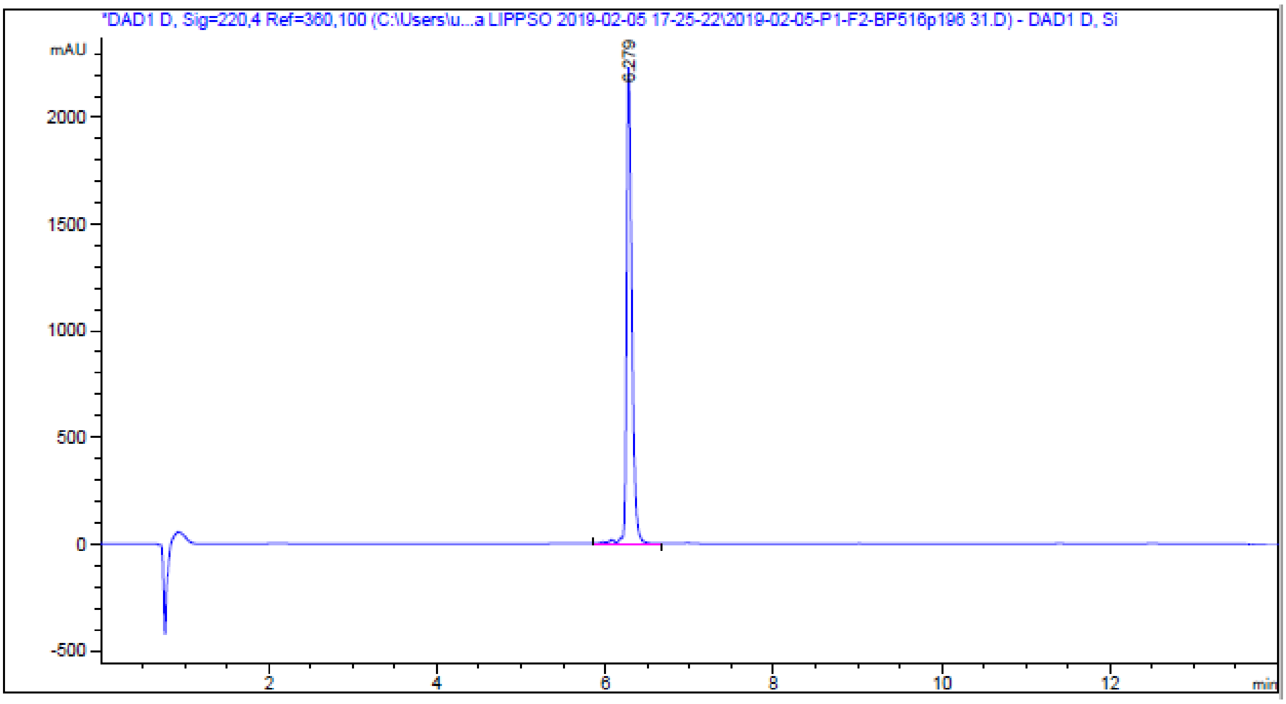

Peak #	RetTime [min]	Type	Width [min]	Area [mAU*s]	Height [mAU]	Area %
1	6.279	VB R	0.0696	1.02472e4	2232.46753	100.0000

Totals : 1.02472e4 2232.46753

ESI-MS (*m/z*)

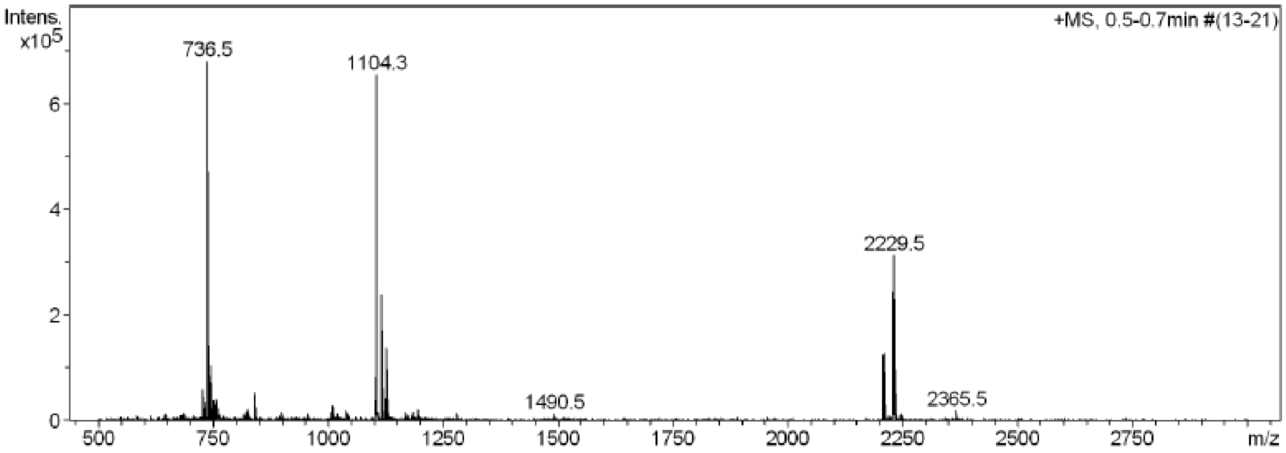

HRMS (*m/z*)

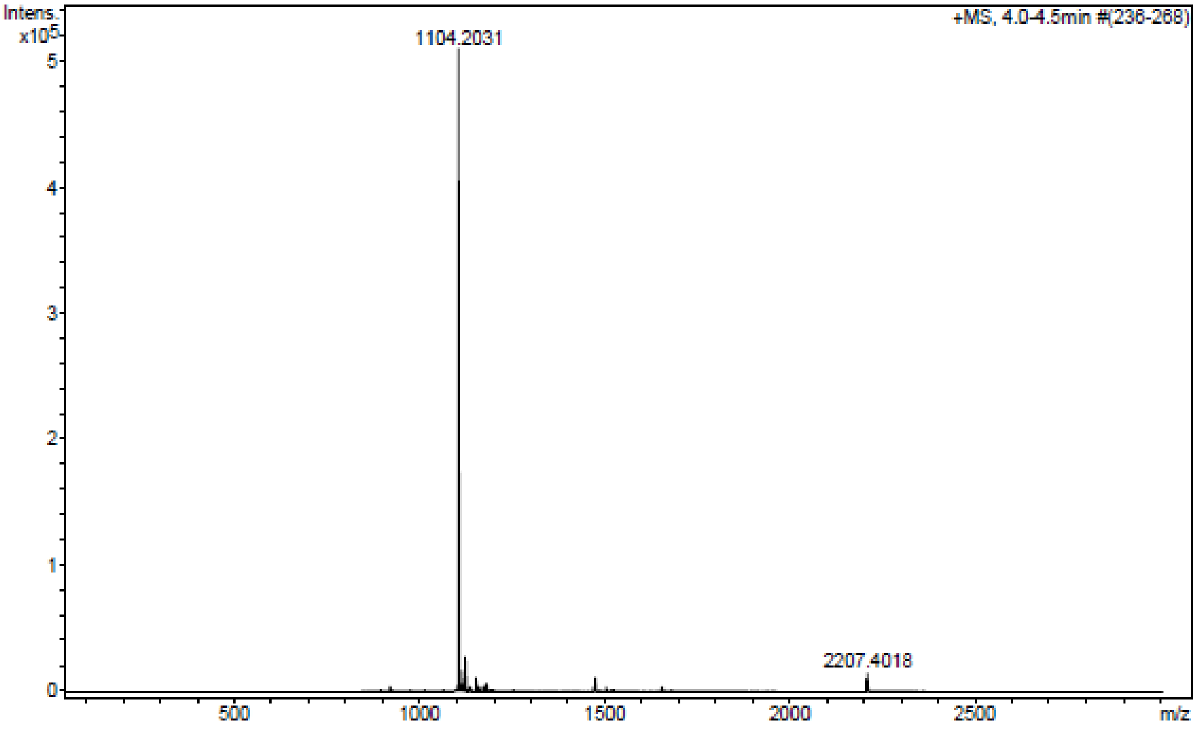

)

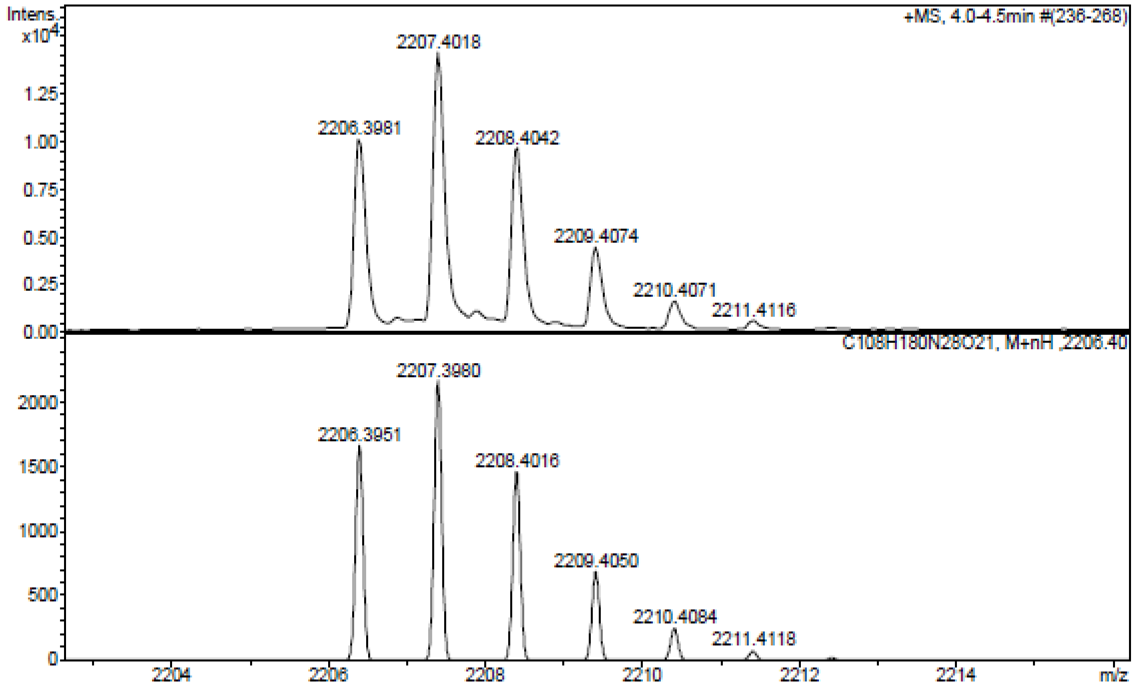
Intens. x10^4
+MS, 4.0-4.5min #(236-268)
2207.4018
2206.3981
2208.4042
2209.4074
2210.4071
2211.4116
C108H180N28O21, M+nH ,2206.40
2207.3980
2206.3951
2208.4016
2209.4050
2210.4084
2211.4118
m/z

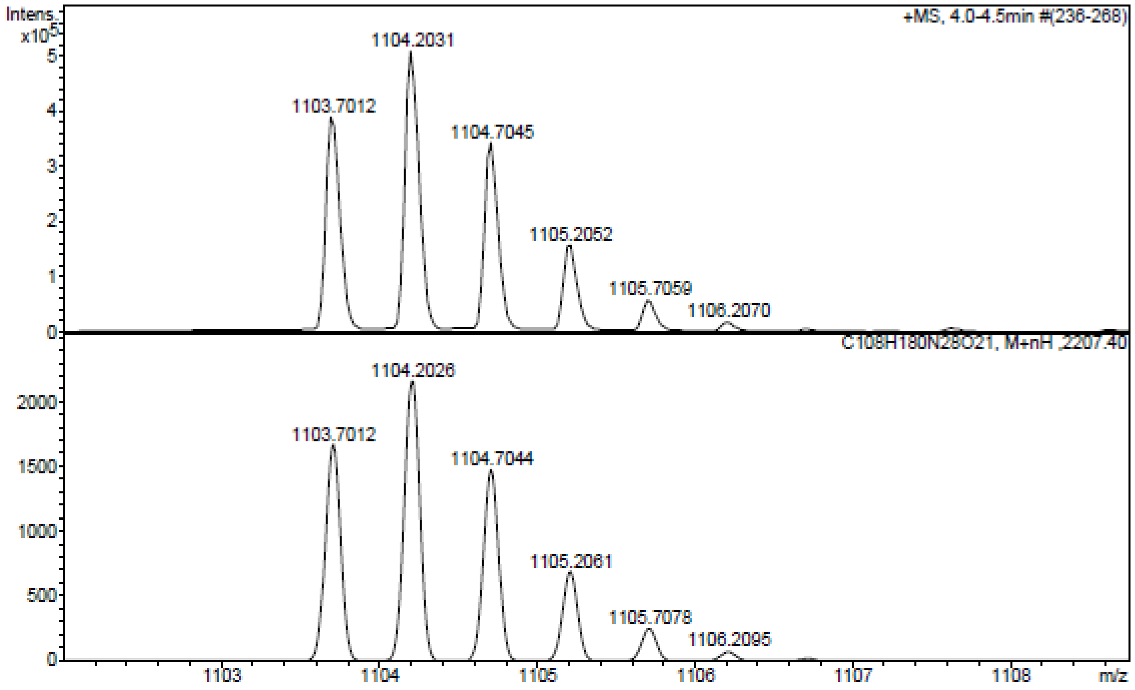
Intens. x10^5
+MS, 4.0-4.5min #(236-268)
1104.2031
1103.7012
1104.7045
1105.2052
1105.7059
1106.2070
C108H180N28O21, M+nH ,2207.40
1104.2026
1103.7012
1104.7044
1105.2061
1105.7078
1106.2095
m/z

Tyr-Gly-Ile-His-Thr-His-Lys-Lys-Val-Val-Phe-Trp-Val-Lys-Phe-Lys-NH2 (PIP1-KSLW)

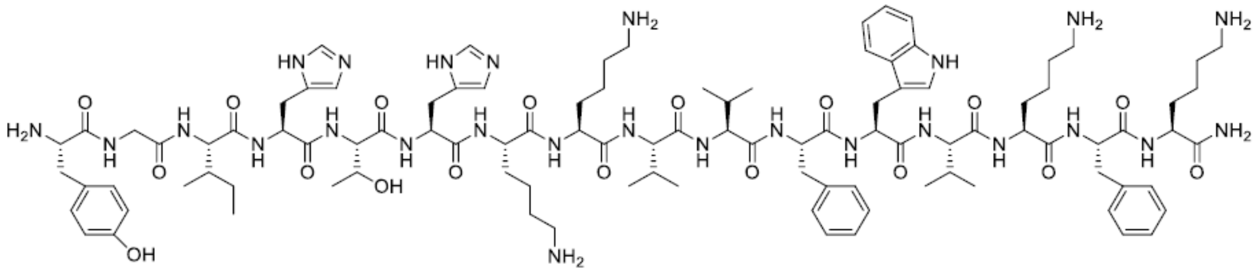

HPLC (λ=220 nm)

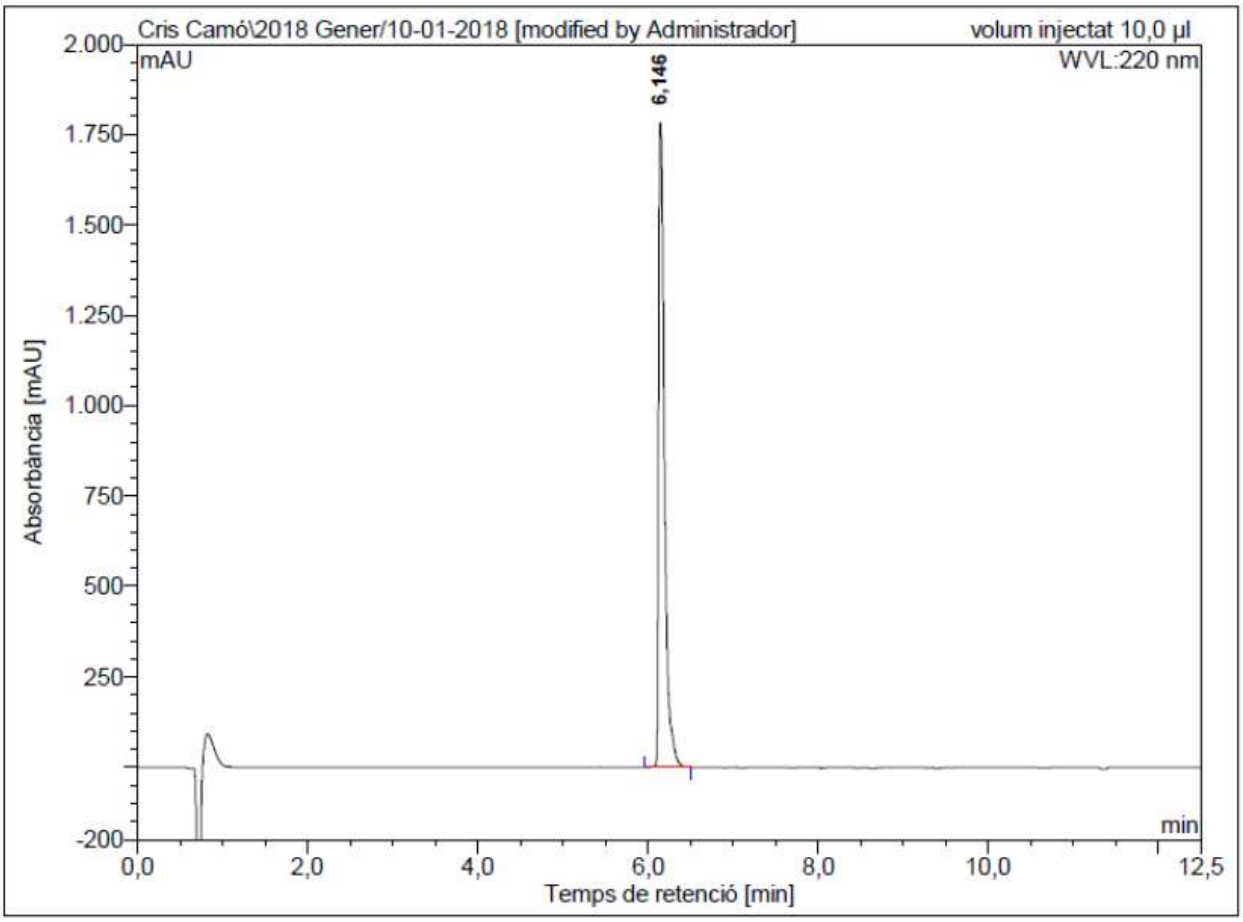

No.	Temps retenció min	alçada mAU	Area mAU*min	Area relativa %
1	6,15	1783,581	144,389	100,00
Total:		1783,581	144,389	100,00

ESI-MS (*m/z*)

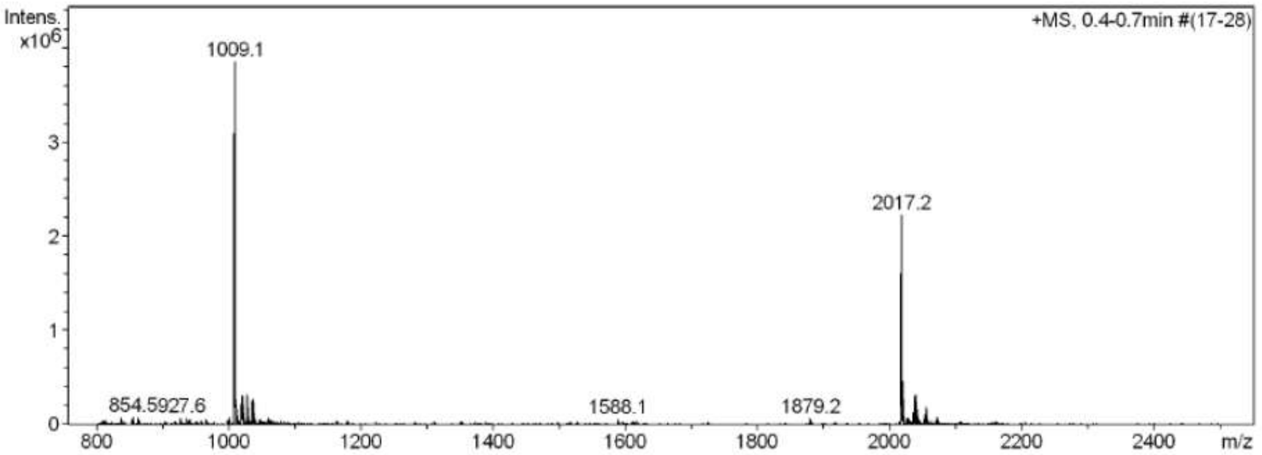

HRMS (*m/z*)

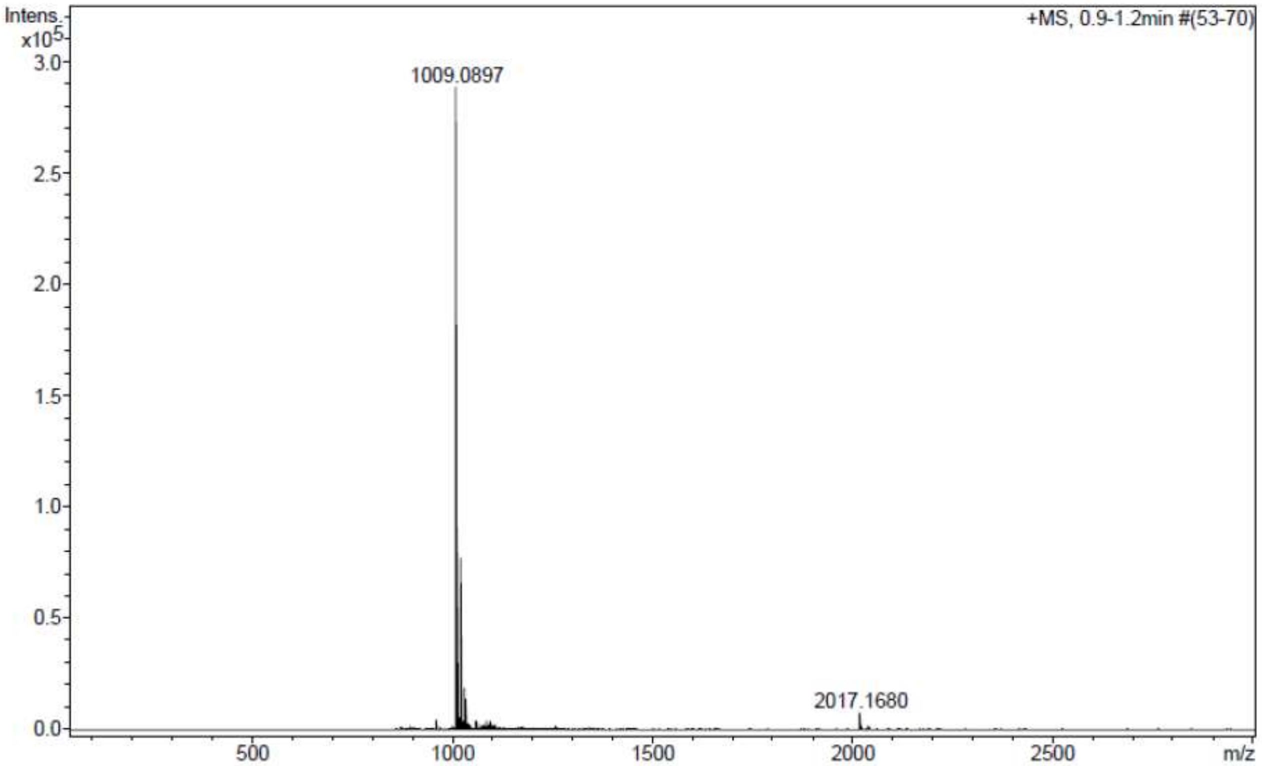

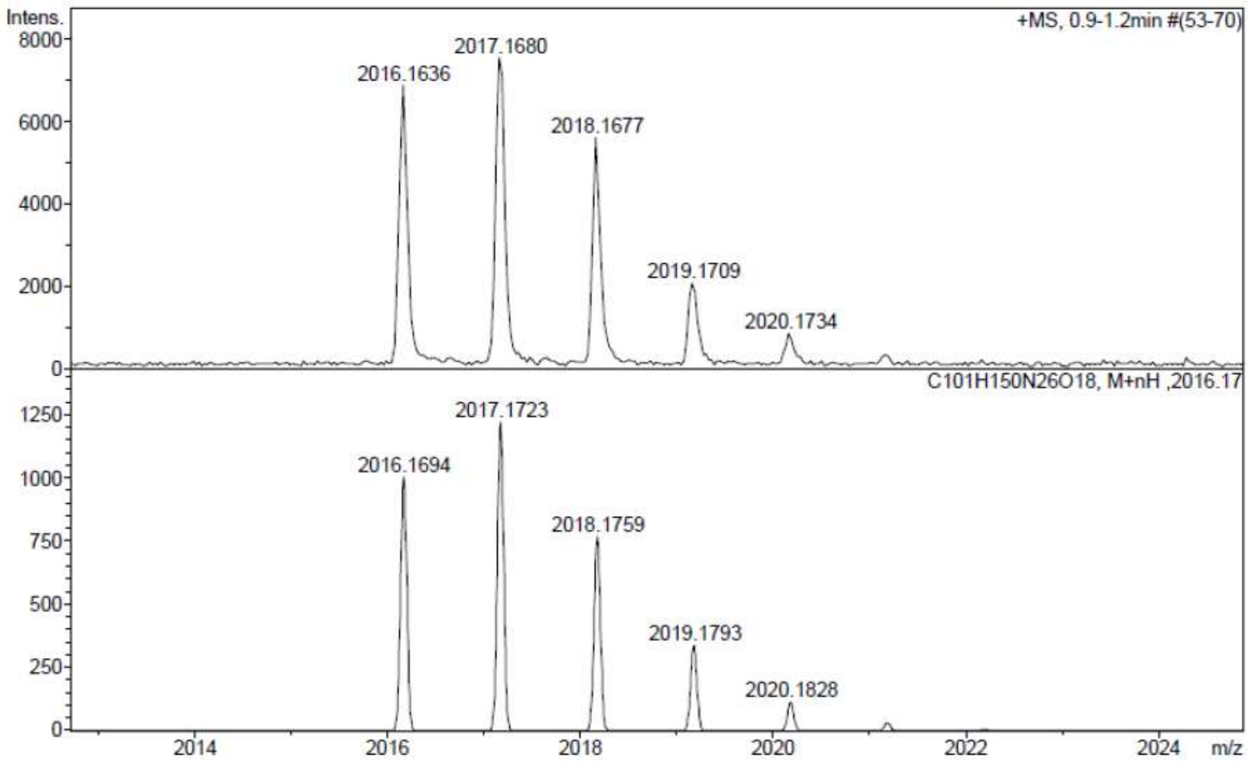
Intens.
+MS, 0.9-1.2min #(53-70)
2016.1636
2017.1680
2018.1677
2019.1709
2020.1734
C101H150N26O18, M+nH ,2016.17
2016.1694
2017.1723
2018.1759
2019.1793
2020.1828
m/z

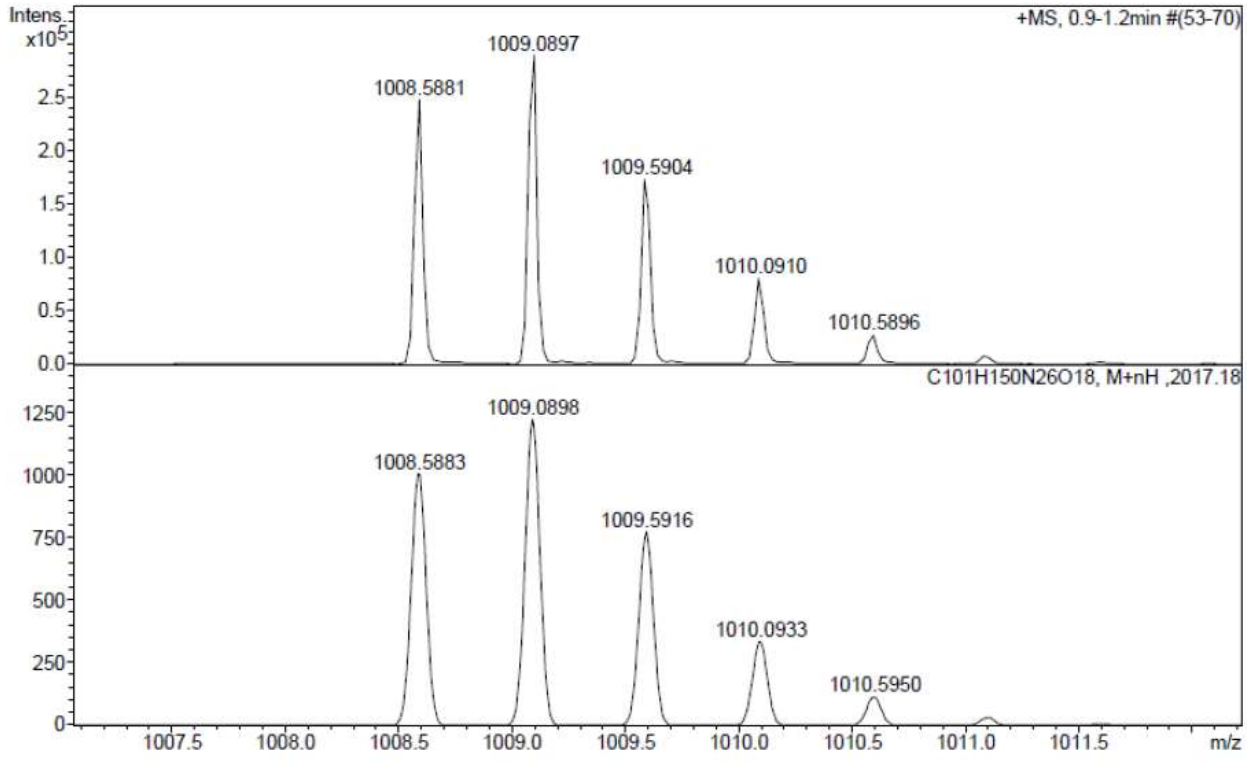
Intens.
x10^5
+MS, 0.9-1.2min #(53-70)
1008.5881
1009.0897
1009.5904
1010.0910
1010.5896
C101H150N26O18, M+nH ,2017.18
1008.5883
1009.0898
1009.5916
1010.0933
1010.5950
m/z

Lys-Lys-Val-Val-Phe-Trp-Val-Lys-Phe-Lys-Tyr-Gly-Ile-His-Thr-His-NH_2 (KSLW-Pip1)

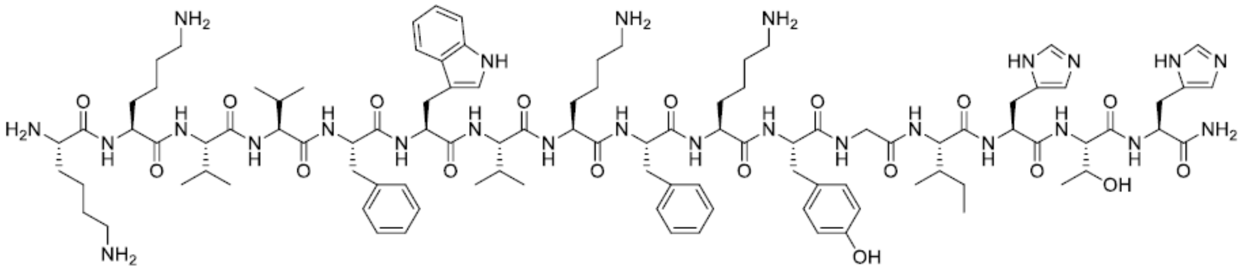

HPLC (λ=220 nm)

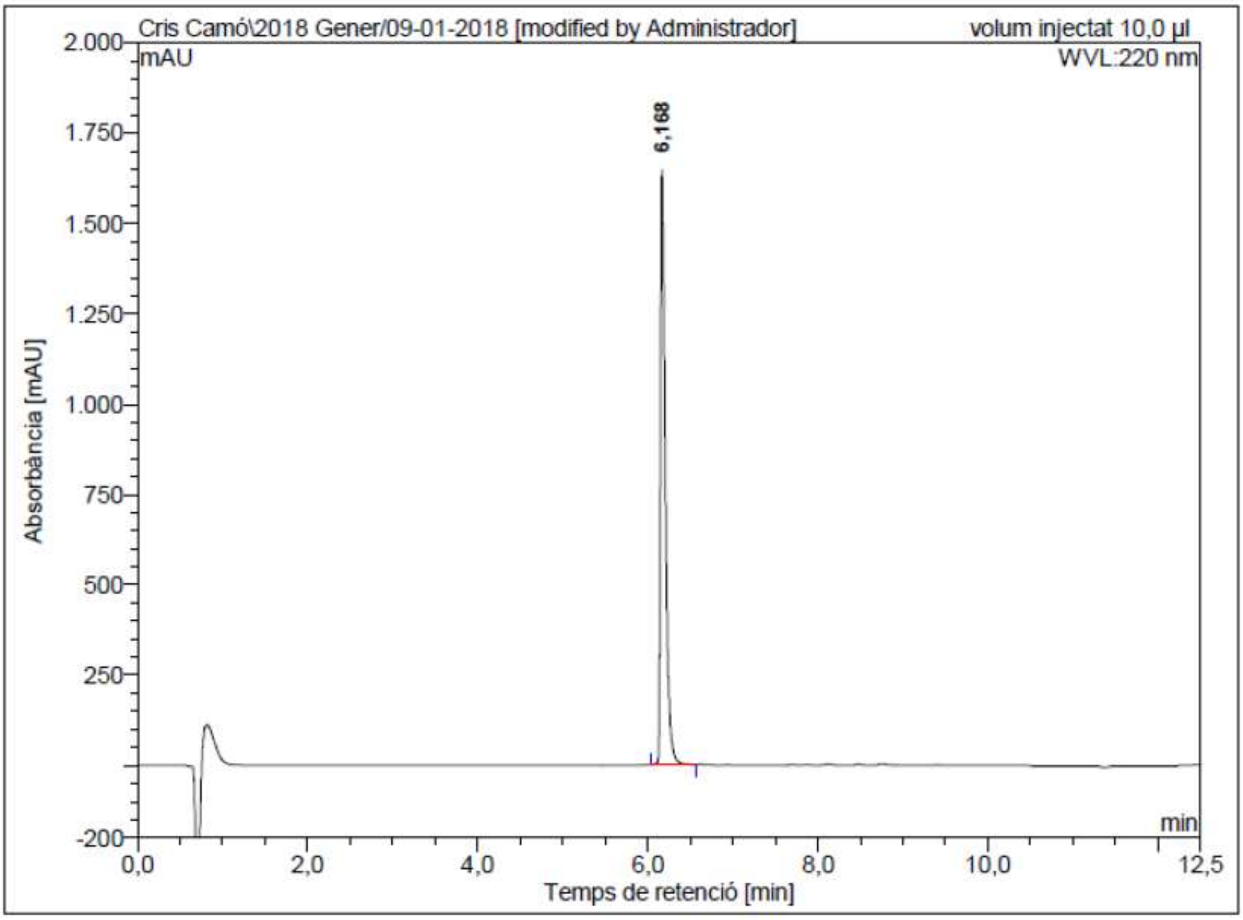

No.	Temps retenció min	alçada mAU	Area mAU*min	Area relativa %
1	6,17	1646,510	108,977	100,00
Total:		1646,510	108,977	100,00

ESI-MS (*m/z*)

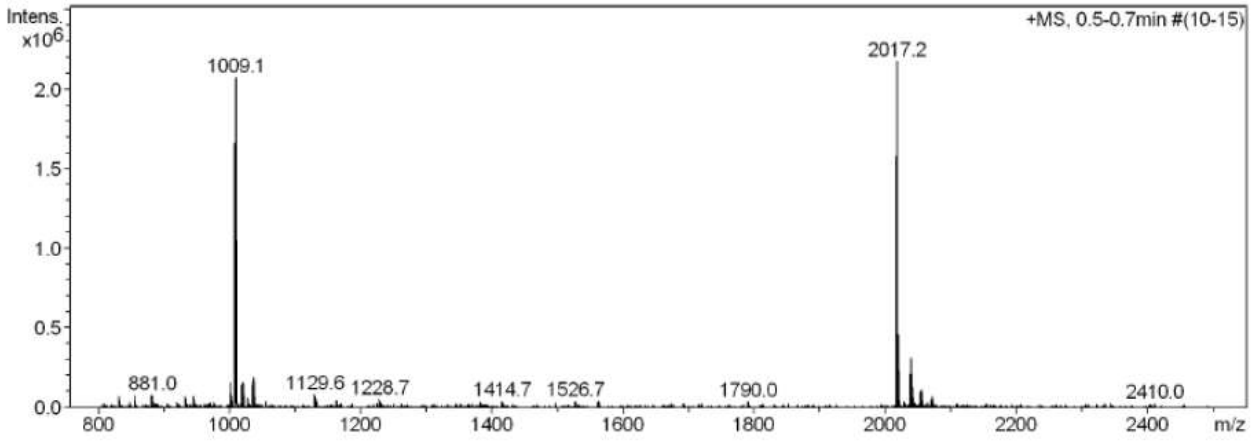

HRMS (*m/z*)

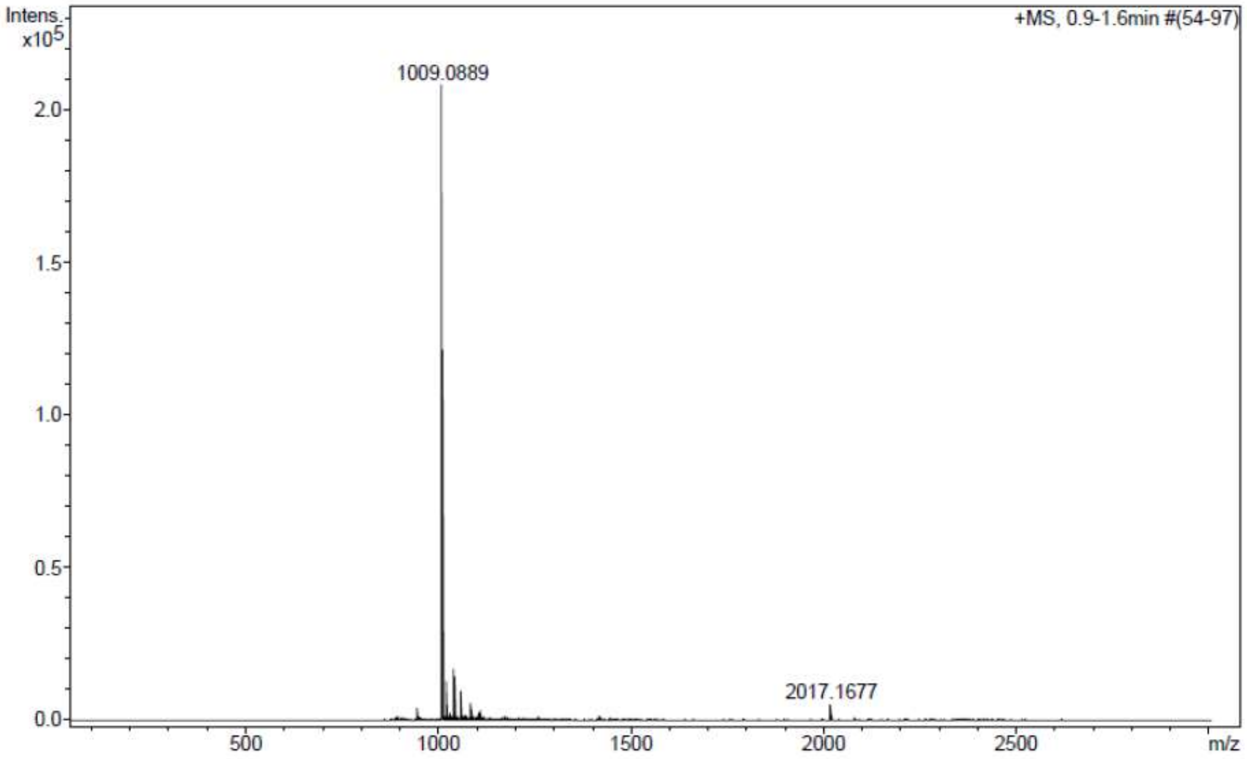

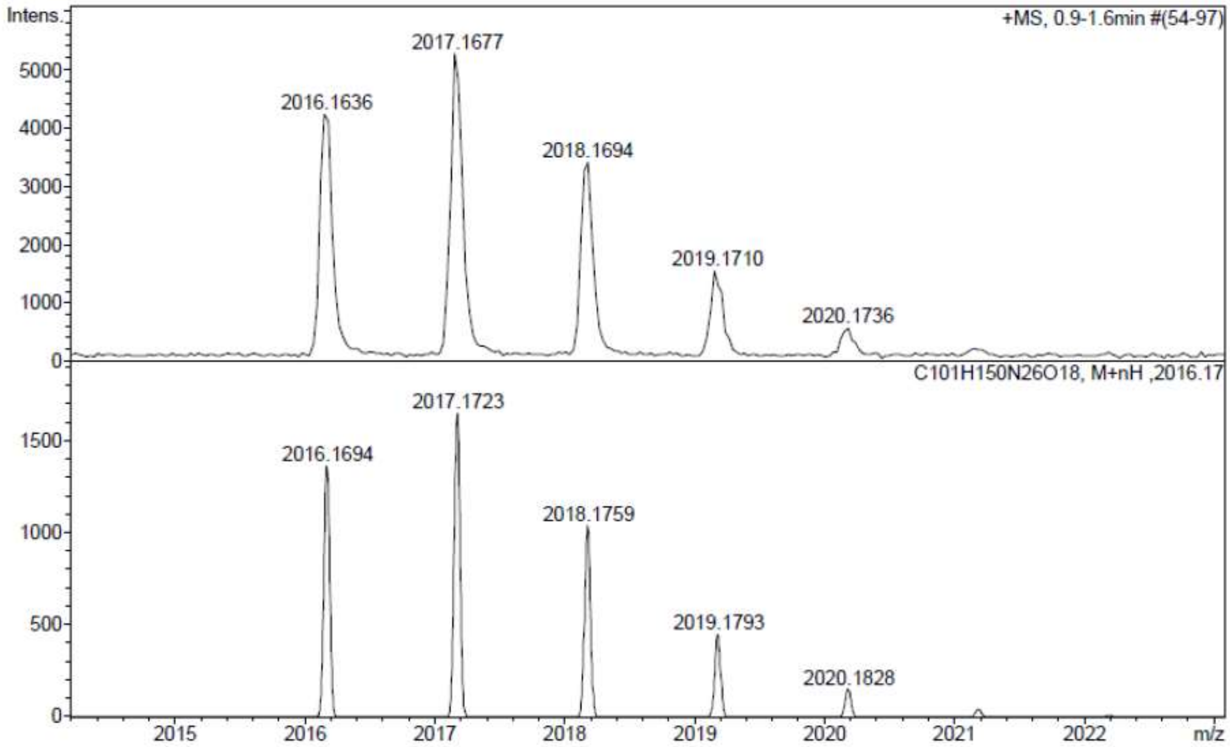
Intens.
+MS, 0.9-1.6min #(54-97)
2016.1636
2017.1677
2018.1694
2019.1710
2020.1736
C101H150N26O18, M+nH ,2016.17
2016.1694
2017.1723
2018.1759
2019.1793
2020.1828
m/z

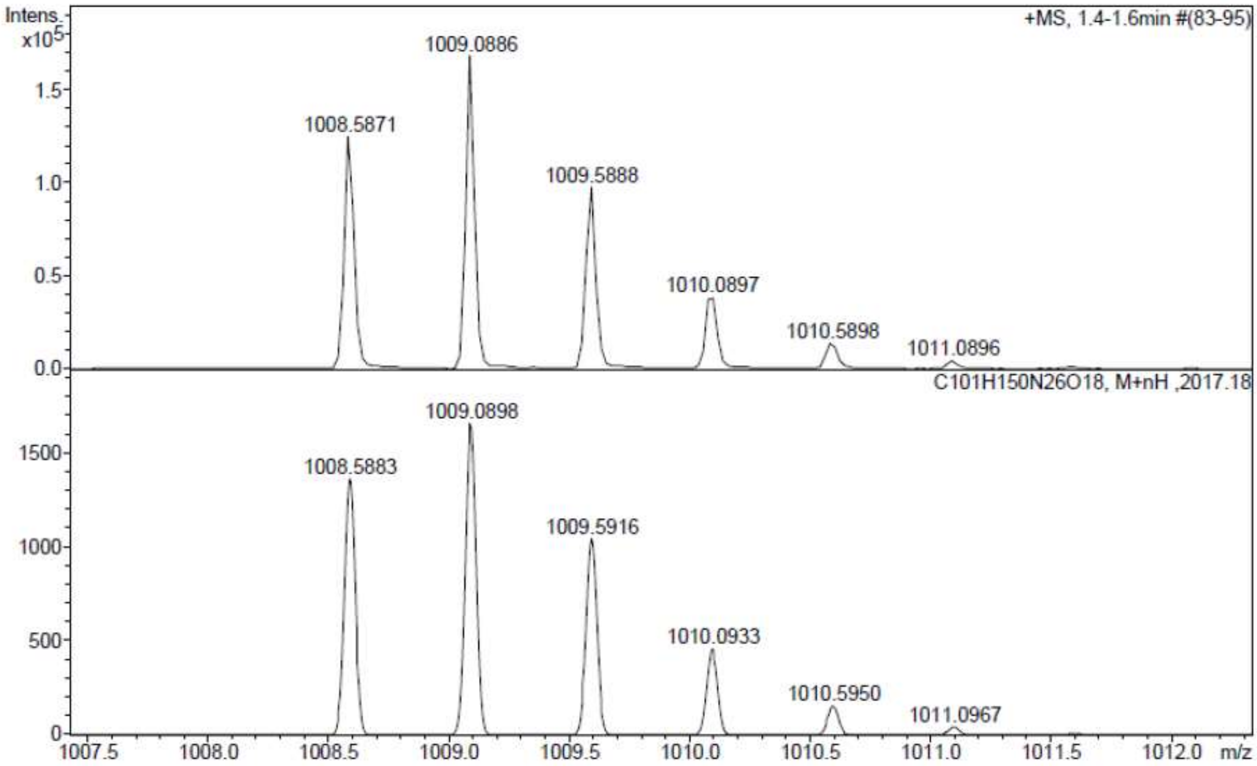
Intens.
x10^5
+MS, 1.4-1.6min #(83-95)
1008.5871
1009.0886
1009.5888
1010.0897
1010.5898
1011.0896
C101H150N26O18, M+nH ,2017.18
1008.5883
1009.0898
1009.5916
1010.0933
1010.5950
1011.0967
m/z

4. α-Helical wheel projections of peptide conjugates

flg15-BP16

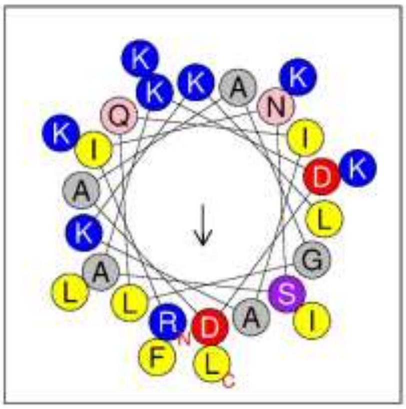

BP16-flg15

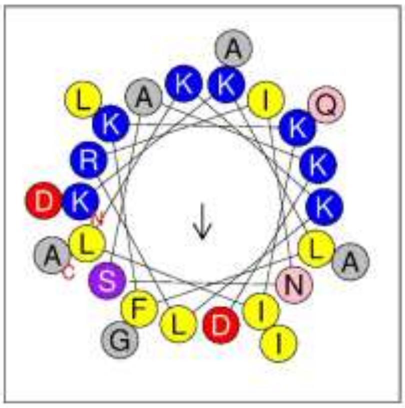

flg15-BP100

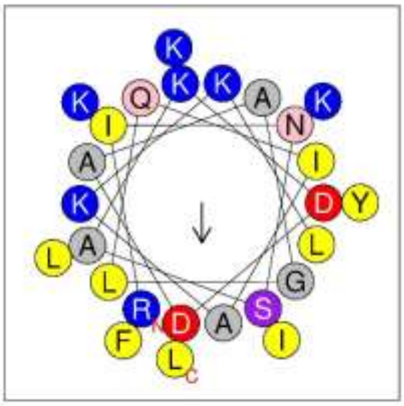

BP100-flg15

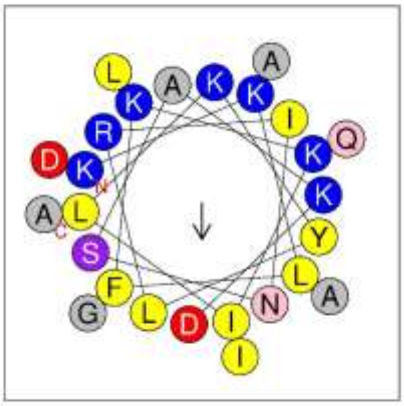

flg15-BP387

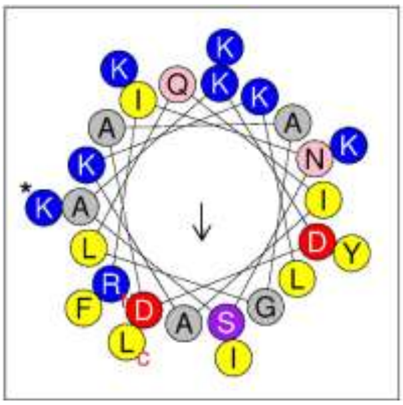

BP387-flg15

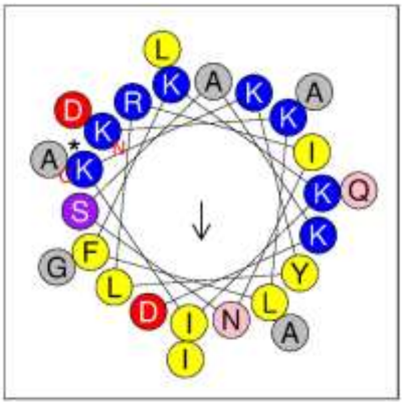

*This Lys residue corresponds to Lys(COC_3H_7)

flg15-BP475

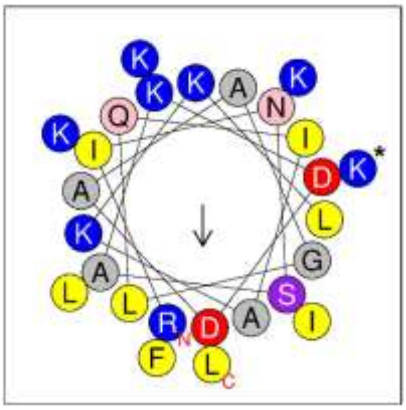

BP475-flg15

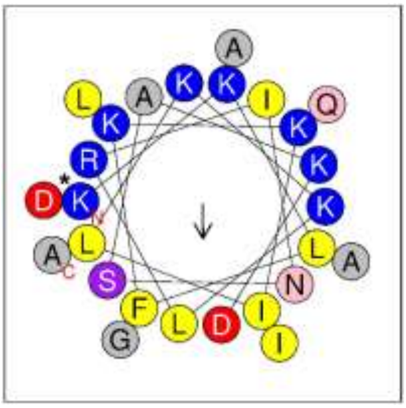

*This Lys residue corresponds to Lys(COC_3H_7). The Phe is a D-Phe

flg15-KSLW

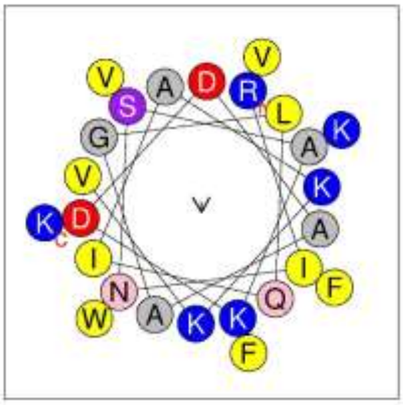

KSLW-flg15

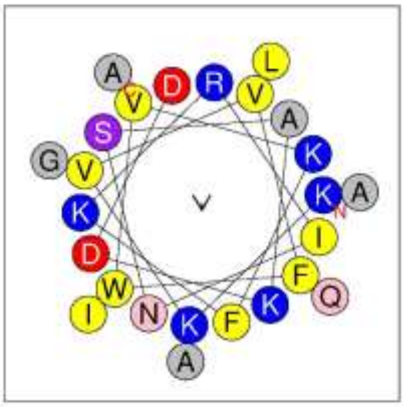

BP13-BP16

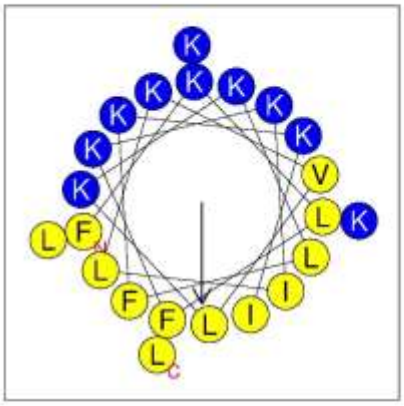

BP16-BP13

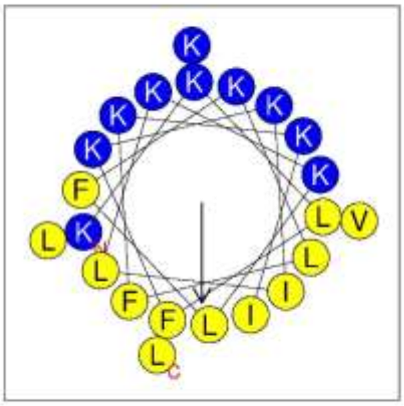

BP13-BP100

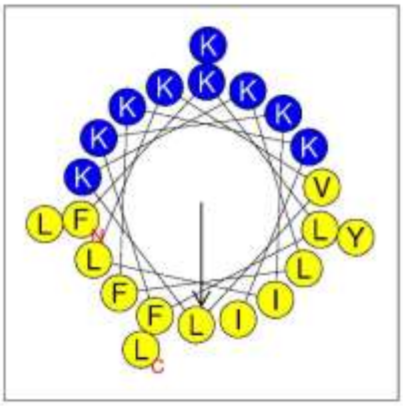

BP100-BP13

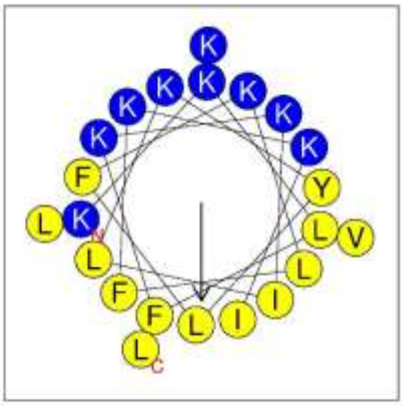

BP13-BP143

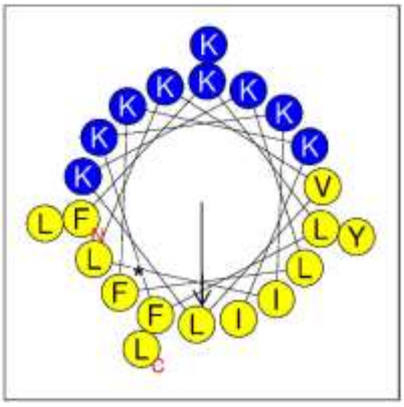

BP143-BP13

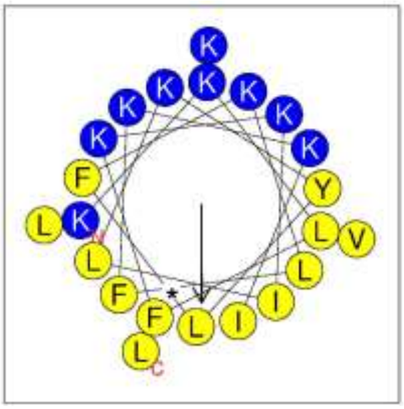

*The Phe is a D-Phe

BP13-KSLW

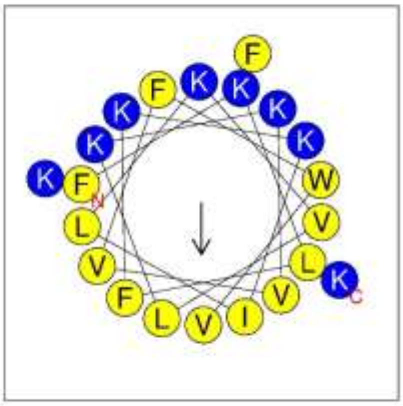

KSLW-BP13

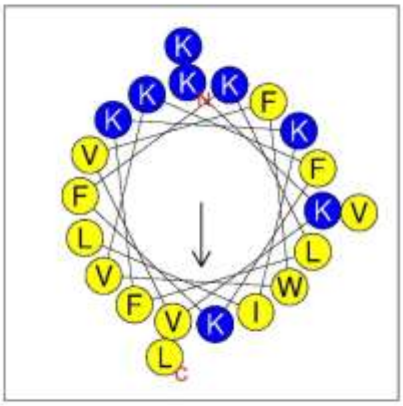

Pep13-BP16

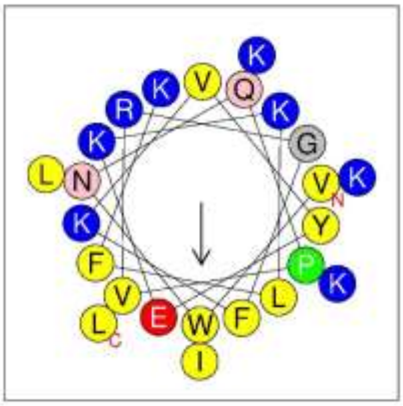

BP16-Pep13

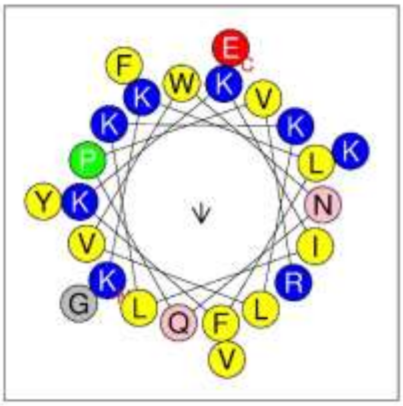

Pep13-BP100

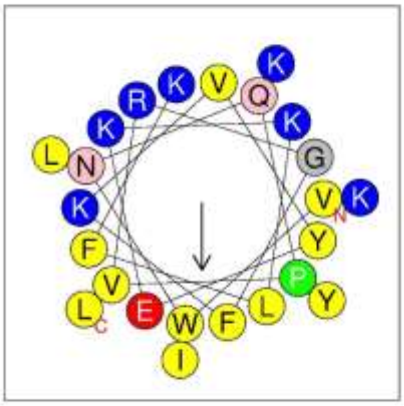

BP100-Pep13

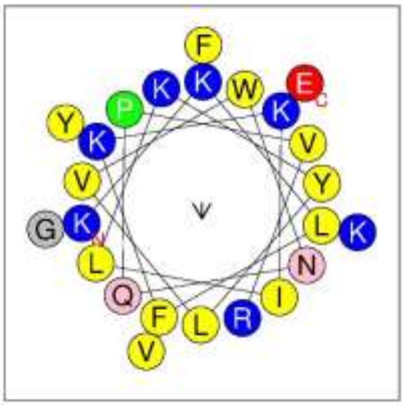

Pep13-BP143

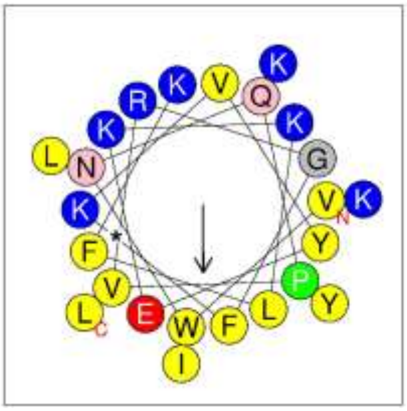

BP143-Pep13

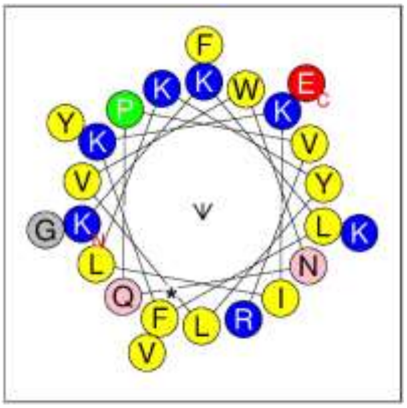

*The Phe is a D-Phe

Pep13-KSLW

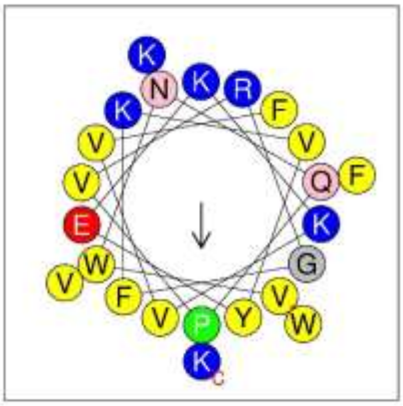

KSLW-Pep13

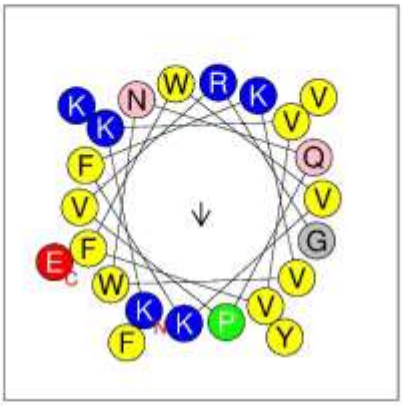

PIP1-BP475

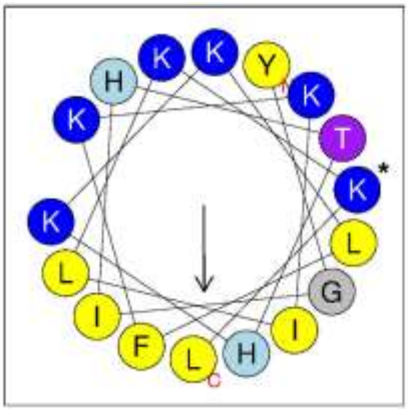

BP475-PIP1

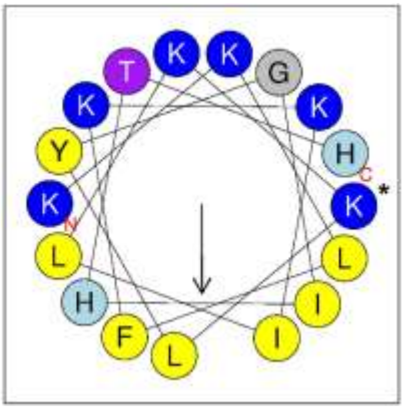

*It corresponds to Lys(COC_3H_7). The Phe is a D-Phe

PIP1-KSLW

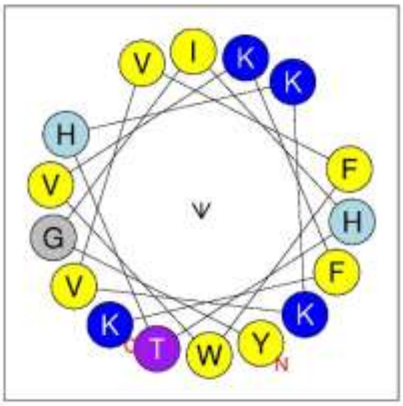

KSLW-PIP1

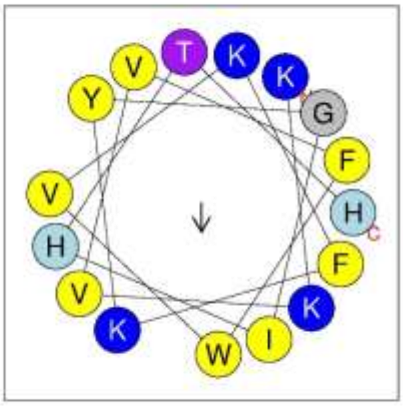

5. Structural characterization of flg15-BP475 by NMR

Table E. ^{1}H, ^{13}C and ^{15}N chemical shifts (ppm) of **flg15-BP475**

Residue	α-C*H*	α-*C*H	β-C*H*	β-*C*H	CO-*N*H	CO-N*H*
**Arg-1**	4.337	56.26	1.783	30.95	125.68	8.019
**Ile-2**	4.178	61.31	1.870	38.98	120.38	7.925
**Asn-3**	4.753	53.43	2.826	38.92	121.64	8.316
**Ser-4**	4.450	58.58	3.910	64.04	115.90	8.148
**Ala-5**	4.322	53.42	1.441	18.82	125.24	8.183
**Lys-6**	4.242	57.22	1.823	32.81	118.36	7.984
**Asp-7**	4.645	54.50	2.818	38.90	119.33	8.027
**Asp-8**	4.611	54.76	2.797	40.04	120.22	8.163
**Ala-9**	4.198	54.26	1.475	18.23	123.09	8.120
**Ala-10**	4.144	54.85	1.468	18.13	121.01	8.050
**Gly-11**	3.861	46.81	-	-	-	8.163
**Leu-12**	4.174	57.40	1.665	42.01	120.78	7.781
**Gln-13**	4.044	58.79	2.180	28.30	116.82	7.839
**Ile-14**	3.724	64.54	1.949	37.90	119.08	7.808
**Ala-15**	3.450	55.36	1.356	17.74	122.58	7.956
**Lys-16**	3.892	59.40	1.859	32.69	114.93	7.863
**Lys-17**	4.161	58.75	1.909	33.04	117.74	7.601
**Leu-18**	4.204	57.33	1.803	43.05	119.43	8.327
**D-Phe-19**	4.144	58.81	3.126	37.65	117.32	8.363
**Lys-20**	3.856	59.53	1.807	32.45	119.52	7.773
**Lys-21**	4.028	59.47	1.981	32.57	118.43	7.714
**Ile-22**	3.835	64.21	2.086	37.73	117.92	8.034
**Leu-23**	4.053	57.98	1.803	41.49	119.08	7.999
**Lys-24**	4.093	58.41	1.957	32.19	117.46	7.749
**Lys-25 (COC_3H_8)**	4.116	58.61	1.803	33.07	118.83	7.683
**Leu-26**	4.194	55.84	1.576	42.66	118.53	7.867

[1]**H-NMR** (400 MHz, Phosphate buffer 20 mM, pH = 6.5 H_2O/D_2O 90:10 + 30% TFE-d[3], 313.0 K), δ (ppm)

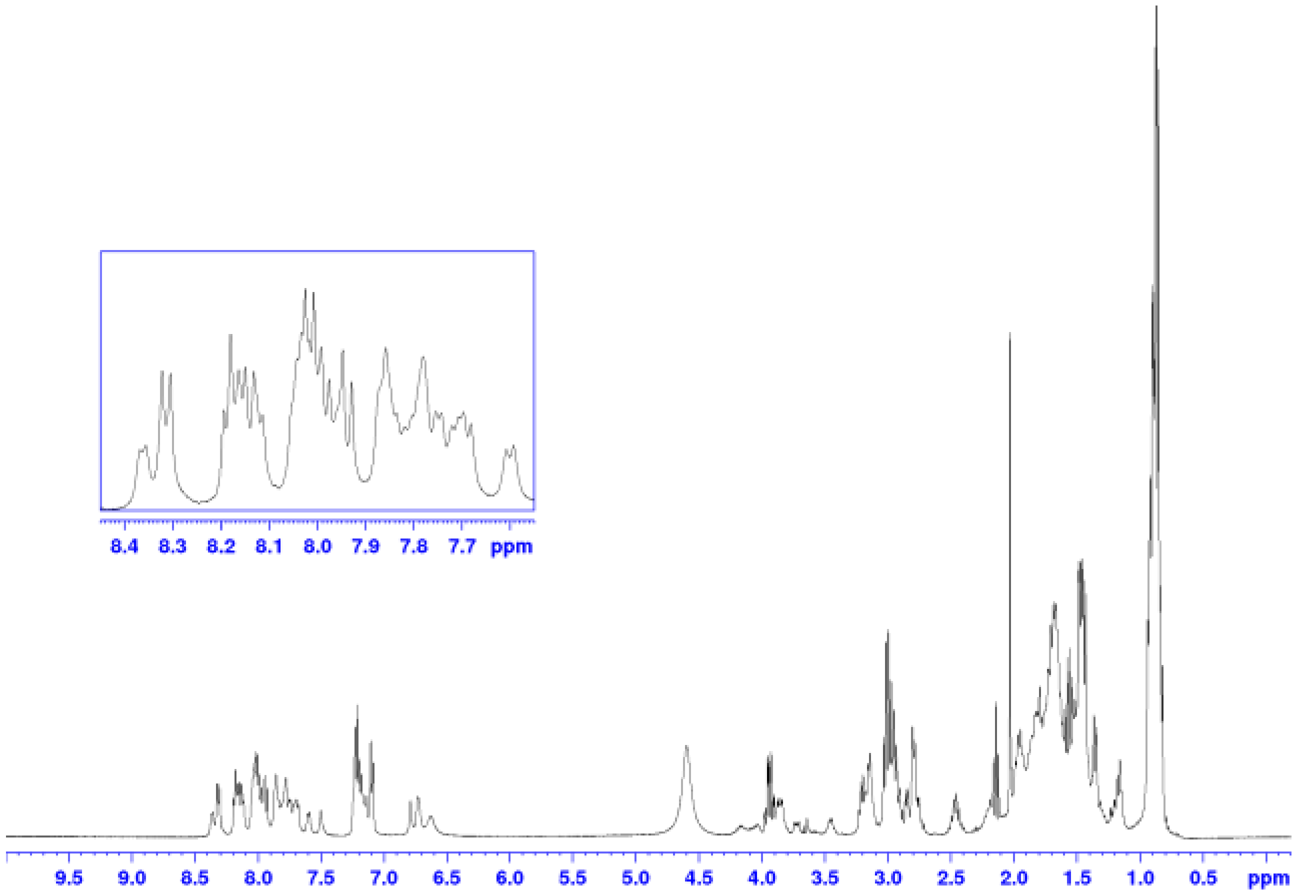

TOCSY 1H-1H, δ (ppm)

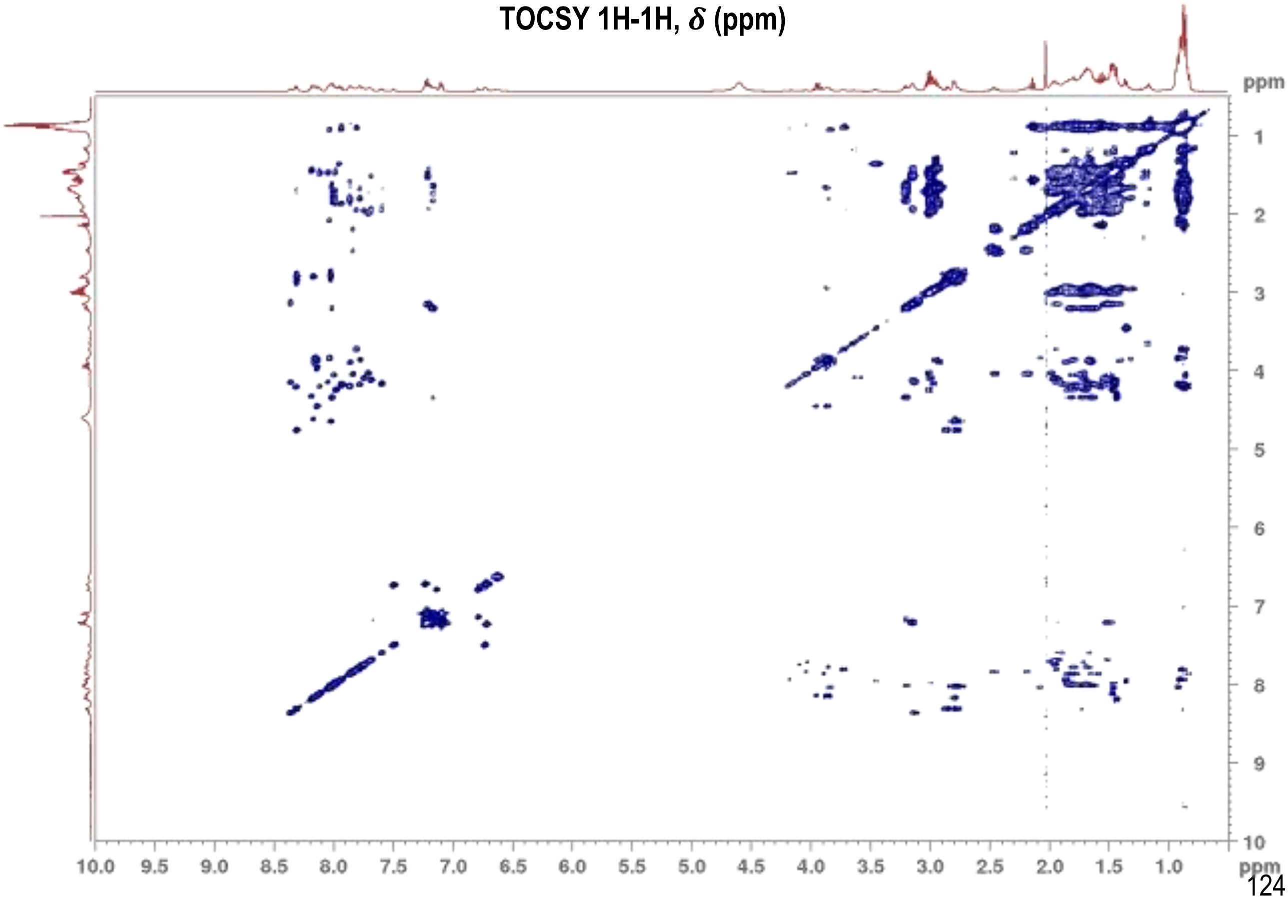

Fingerprint region TOCSY (blue signals) and NOESY (red signals)

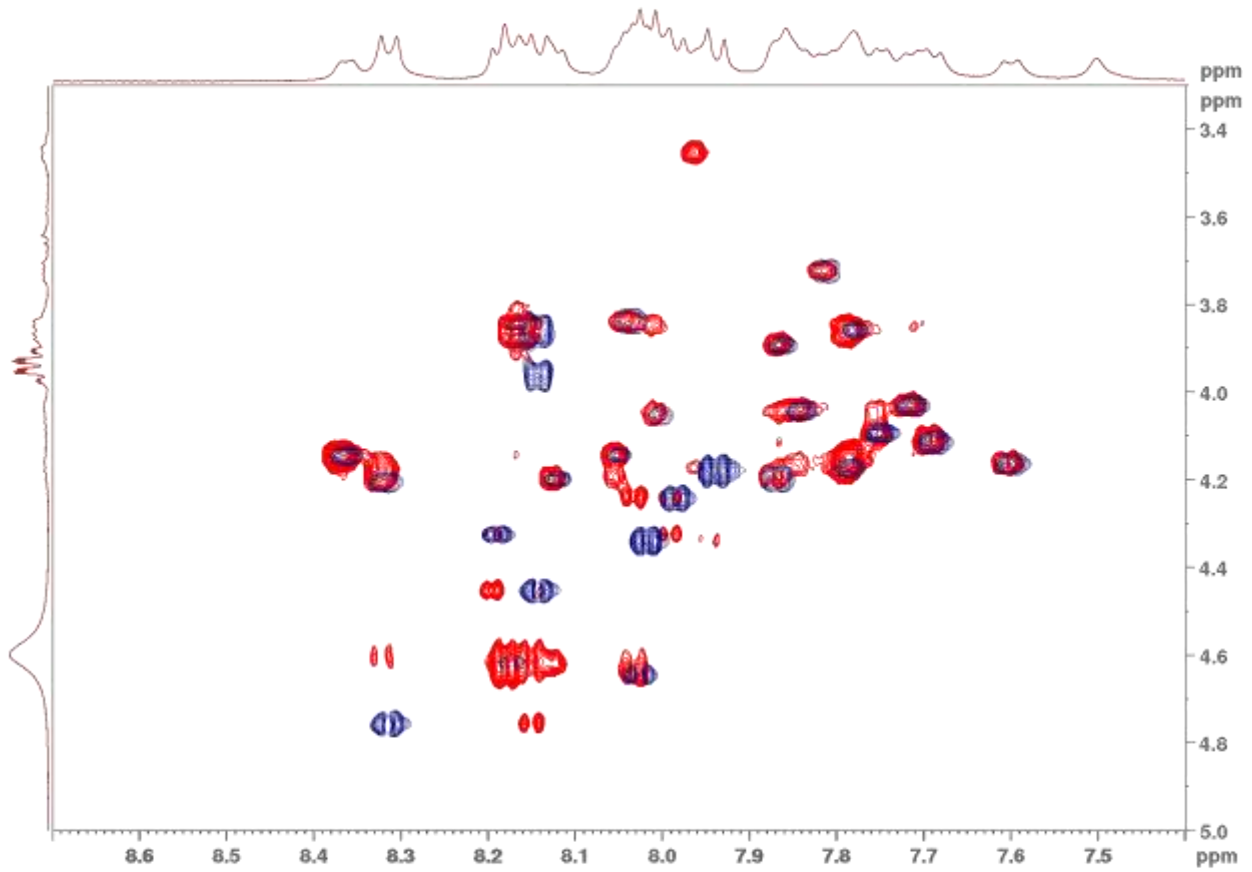

NOESY ^1H-^1H, δ (ppm)

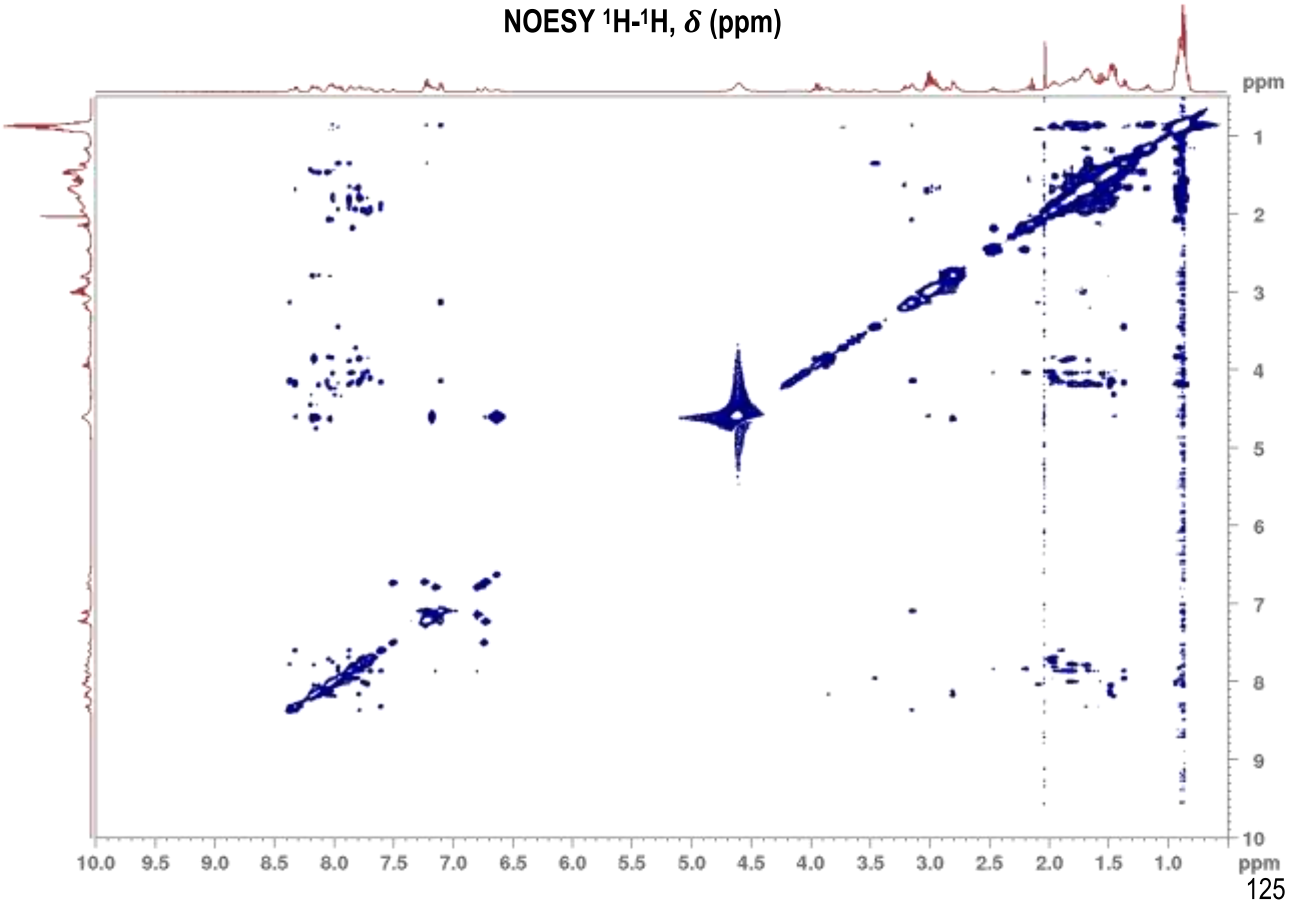

Multiplicity-edited HSQC ^{1}H-^{13}C, δ (ppm)

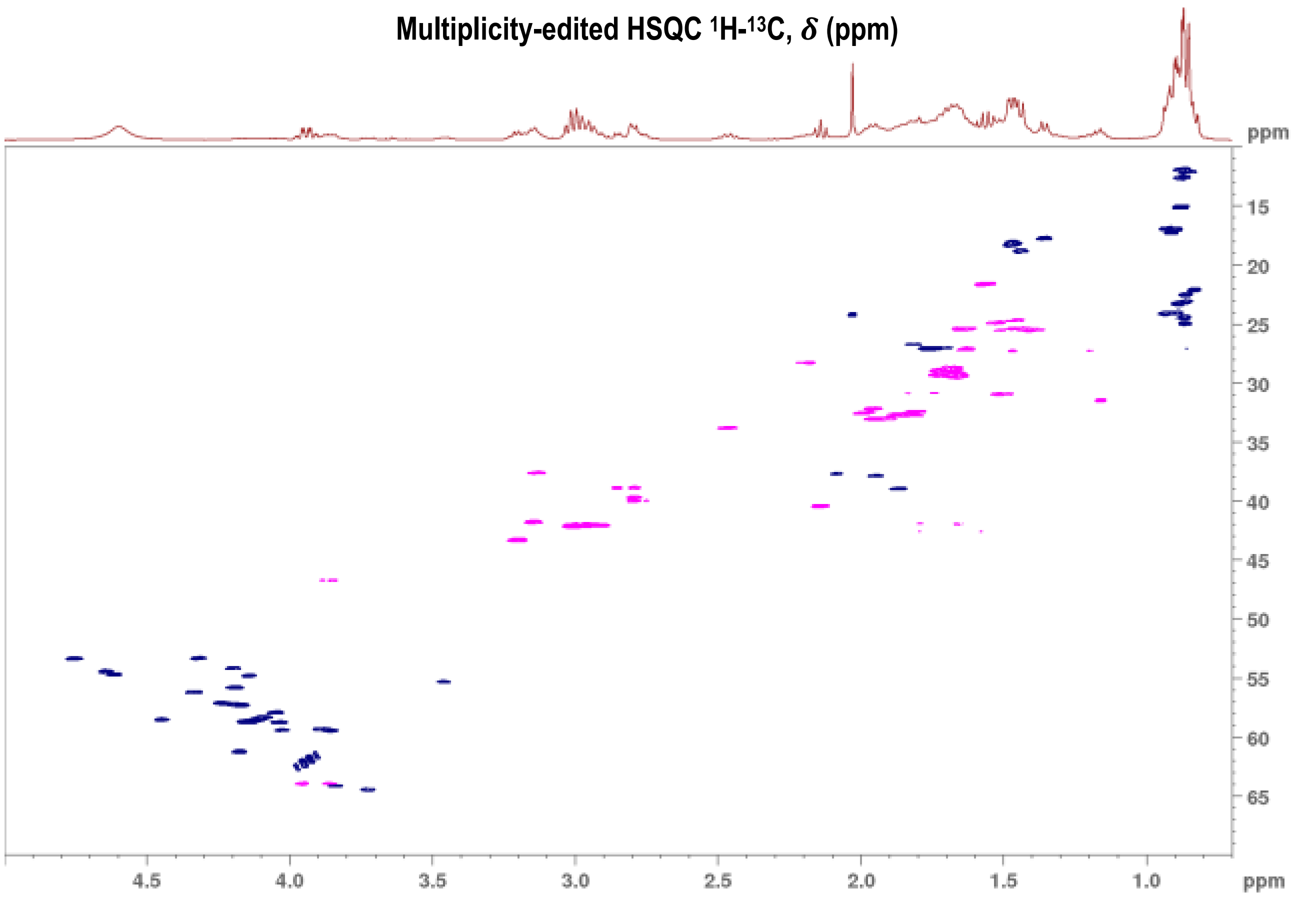

HSQC-TOCSY ^{1}H-^{13}C, δ (ppm)

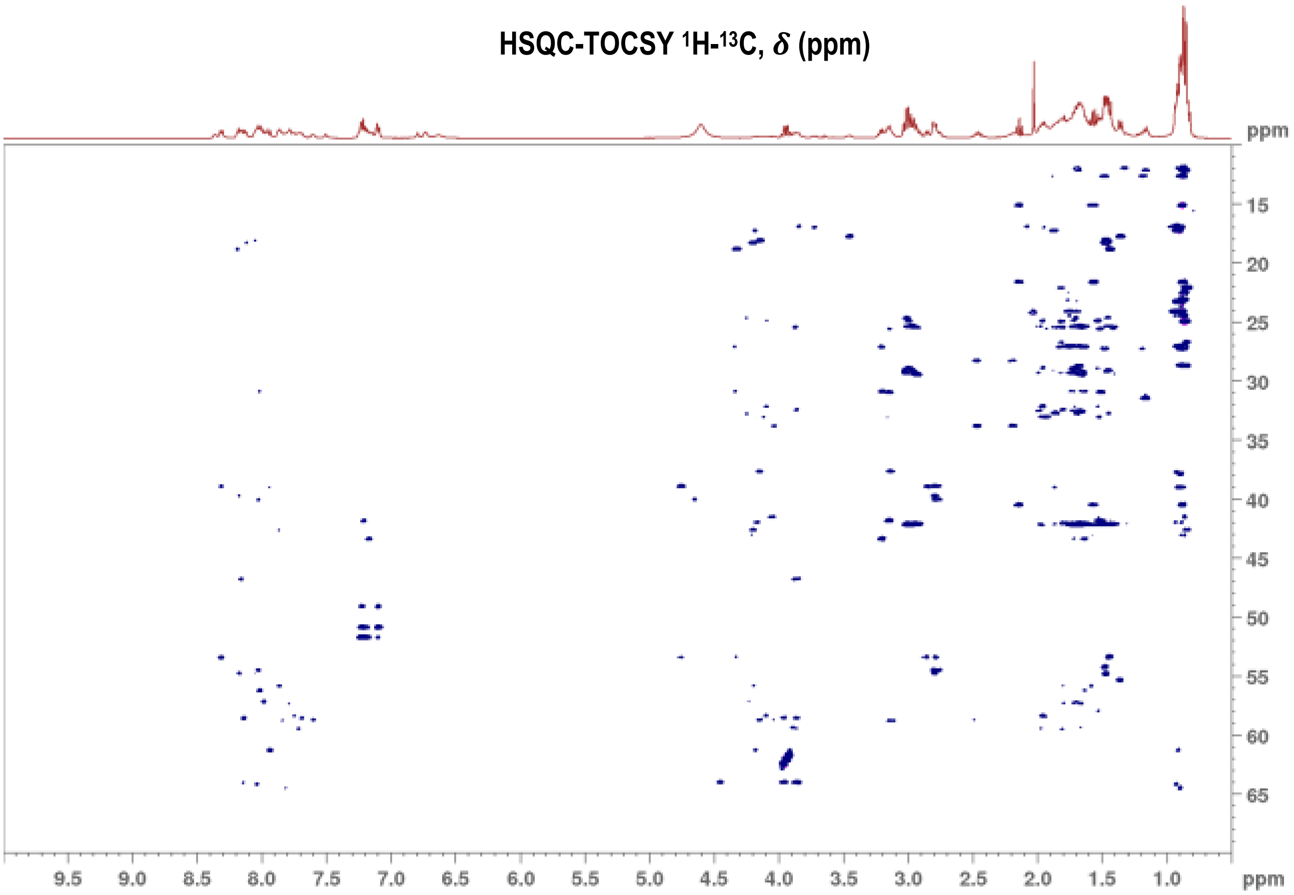

HSQC ^{1}H-^{15}N, δ (ppm)

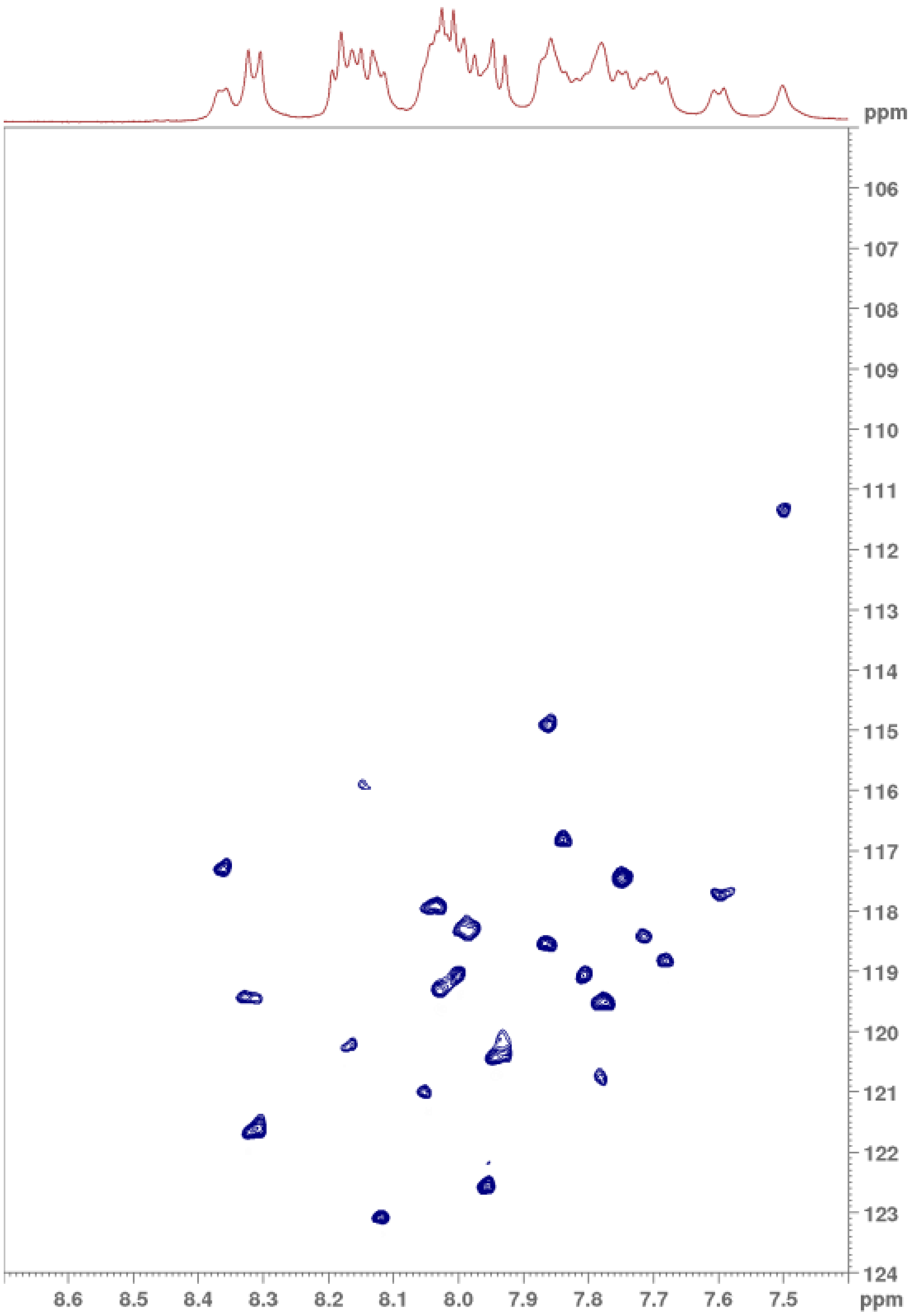

Supplement: Supplemental file 1 — Supplemental information on biological activity, synthesis, HPLC, ESI-MS, HRMS, and NMR. Download aem.00574-22-s0001.pdf, PDF file, 5.2 MB [file aem.00574-22-s0001.pdf]
